# Malignant stroma increases luminal breast cancer cell proliferation and angiogenesis through platelet-derived growth factor signaling

**DOI:** 10.1186/1471-2407-14-735

**Published:** 2014-10-01

**Authors:** Mauricio P Pinto, Wendy W Dye, Britta M Jacobsen, Kathryn B Horwitz

**Affiliations:** Departments of Medicine, Mail Stop 8106, 12801 East 17th Avenue, Aurora, CO 80045 USA; Division of Endocrinology, Metabolism & Diabetes and Pathology, University of Colorado, Anschutz Medical Campus, Aurora, Colorado USA; Universidad Andres Bello, Facultad de Medicina, Center for Integrative Medicine and Innovative Science (CIMIS), Echaurren 183, Santiago, 8370071 Chile

**Keywords:** Stroma, Proliferation, Angiogenesis, Breast cancer

## Abstract

**Background:**

Luminal, estrogen receptor-positive breast cancers represent more than 70% of cases. Despite initial good prognoses one third of Luminal cancers eventually recur locally or at distant sites and exhibit hormone resistance. Here we demonstrate that factors elaborated by malignant stromal cells can induce Luminal tumor cells proliferation and promote angiogenesis and hormone independence. We recently isolated a malignant mouse mammary gland stromal cell line named BJ3Z that increases proliferation and angiogenesis in estrogen-free xenografted Luminal MCF-7 breast cancer cells.

**Methods:**

BJ3Z and Normal mouse mammary Fibroblasts (NMFs) were expression profiled using microarray assays. Messenger RNA levels were confirmed by RT-PCR and by immunohistochemistry (IHC). Breast cancer MCF-7, BT-474, BT-20 and MDA-MB-231cell lines and stromal BJ3Z and NMFs were grown for *in vitro* assays: breast cancer cell lines were treated with stromal cells conditioned media, for three-dimensional (3D) mono and co-cultures in Matrigel, proliferation was measured by Bromo-deoxyuridine (BrdU) incorporation using IHC. Tubule formation *in vitro*, a proxy for angiogenesis, was assessed using 3D cultured Human Umbilical cord Vascular Endothelial Cells (HUVEC).

**Results:**

We show that under estrogen-free conditions, BJ3Z cells but not NMFs increase proliferation of co-cultured Luminal but not basal-like human breast cancer cells in 2D or as 3D Matrigel colonies. Gene expression profiling, RT-PCR analysis and IHC of colony-derived BJ3Z cells and NMFs shows that Platelet Derived Growth Factor ligands (PDGF-A and -B) are elaborated by BJ3Z cells but not NMFs; while PDGF receptors are present on NMFs but not BJ3Z cells. As a result, in colony co-culture assays, BJ3Z cells but not NMFs increase MCF-7 cell proliferation. This can be mimicked by direct addition of PDGF-BB, and blocked by the PDGF receptor inhibitor Imatinib Mesylate. Both normal and malignant stromal cells enhance angiogenesis in an *in vitro* model. This effect is also due to PDGF and is suppressed by Imatinib.

**Conclusions:**

We provide evidence that Luminal breast cancer cells can be targeted by the PDGF signaling pathway leading to estrogen-independent proliferation and angiogenesis. We speculate that stroma-directed therapies, including anti-PDGFR agents like Imatinib, may be useful in combination with other therapies for treatment of luminal cancers.

**Electronic supplementary material:**

The online version of this article (doi:10.1186/1471-2407-14-735) contains supplementary material, which is available to authorized users.

## Background

Human breast cancer is a heterogeneous disease. Using expression profiling Perou *et al.* [1] described several molecular subtypes among which are three main categories: Luminal cancers that express Estrogen (ER) and/or Progesterone (PR) Receptors but lack Human Epidermal Growth Factor Receptor-2 (HER2); HER2-overexpressing cancers; and basal-like cancers that lack ER, PR and HER2. Luminal cancers are by far the most common (>70% of cases) and have the best prognosis among the three major subtypes [2]. Clinically Luminal cancers are treated with endocrine therapies like fulvestrant or tamoxifen that target ER or aromatase inhibitors that suppress estrogen production. Despite the initial effectiveness of these therapies, approximately one-third of Luminal cancers acquire hormone resistance, enhancing their aggressiveness and leading to local recurrence or distant metastases [3]. Several mechanisms have been proposed for growth resumption associated with loss of regulation by estrogens [4]. Here we address the role of the tumor microenvironment.

The microenvironment, including peritumoral stroma that surrounds malignant epithelial cells, plays a major role in cancer progression and metastasis [5]. Carcinoma Associated Fibroblasts (CAFs) are the most abundant cells in such stroma and secrete a variety of factors that encourage tumor progression [6]. In xenografted human Luminal MCF-7 cells, CAFs enhance tumor growth and metastasis and promote angiogenesis by recruiting endothelial progenitor cells. Other studies suggest that CAFs play a critical role in hormone resistance. For example, co-culture of tamoxifen-sensitive cells with CAFs significantly decreases their sensitivity to tamoxifen while activating MAPK and AKT pathways [7]. In MCF-7 cells, CAFs can also induce tamoxifen and fulvestrant resistance by altering mitochondrial functions [8]. Thus the effects of peritumoral stromal cells in breast cancers are complex and effective therapeutic targets for hormone resistance generated by the proximity of such cells may delay disease recurrence.

Growth factors are important cancer regulators. Platelet-Derived Growth Factors (PDGF A, B, C and D) belong to a four-member family of factors that activate two tyrosine kinase receptors PDGFR-α and PDGFR-β [9–11]. PDGF was identified 40 years ago [12] as a constituent of whole blood serum and it is synthesized by many different cell types, including fibroblasts [13]. PDGF is a mitogen, but it also has significant angiogenic effects on endothelial cells. In fact, PDGF and Vascular Endothelial Growth Factor (VEGF) are structurally and functionally related and are conserved throughout the animal kingdom [10].

PDGF signaling is altered or overactive in many malignancies [9, 14–16]. Therapies targeting the PDGFR pathway reduce tumor growth in prostate [17], endometrial [18], pancreatic [19] and lung [20] cancers, as well as in osteosarcomas [21]. In breast cancers, TGF-β initiates an autocrine PDGF/PDGFR signaling loop critical for epithelial-to-mesenchymal transition and metastasis [22]. *In vitro*, PDGF-BB increases proliferation of breast cancer cells that can be inhibited by drugs targeting the PDGF pathway [23]. PDGF also plays a major role in initiating the “desmoplastic response” of breast cancers [24, 25]. Lastly, patients with recurrent disease have elevated circulating PDGF levels, suggesting that it may serve as a recurrence marker [26].

We have developed a model to study the effects of peritumoral stroma *in vitro*. We previously isolated and cultured malignant mouse mammary gland stromal cells we called BJ3Z cells [27]. They are derived from normal mouse fibroblasts that were transformed by proximity to human breast cancer cells grown *in vivo* as xenografts in immuno-compromised mice. BJ3Z cells are tumorigenic when injected into mice and enhance angiogenesis and proliferation of co-injected human MCF-7 cells [28]. Here we address *in vitro* mechanisms by which BJ3Z cells control growth and aggressiveness of human breast cancer cells using normal mammary gland fibroblasts (NMFs) as controls. We find that unlike NMFs, BJ3Z cells enhance proliferation of co-cultured Luminal but not basal-like breast cancer cells. Gene expression profiling shows that malignant BJ3Z cells overexpress PDGF ligands. We demonstrate that PDGF increases proliferation of Luminal breast cancer cells in the absence of estrogens. PDGF also stimulates angiogenesis in an *in vitro* model. Both effects can be prevented by Imatinib Mesylate; a potent PDGF receptor kinase inhibitor. Our studies suggest that stroma-directed therapies including anti-PDGFR agents may be useful in combination therapies for Luminal cancers.

## Methods

### Ethics statement

This study did not involve human subjects or clinical materials. The human breast cancer cell lines are commercially available. The research was approved by University of Colorado institutional review committees and granting agencies.

### Cell lines

MCF-7 human breast cancer cells were obtained from the Michigan Cancer Foundation; BT-474, MDA-MB-231, BT-20 and Human Umbilical Cord Vascular Endothelial Cells (HUVEC) were from the ATCC (Manassas VA). Transformed mouse mammary stromal cells (BJ3Z) were developed in our laboratory [27, 29]; normal mouse mammary fibroblasts (NMF) were a kind gift of L. Wakefield (NCI) [27, 29]. All cell lines were authenticated by Single Tandem Repeat analysis at the CU Cancer Center Sequencing Core and were mycoplasma-free. Cells were routinely passaged in minimum essential medium (MEM; Invitrogen, Carlsbad CA) containing 5% fetal calf serum (FCS; HyClone, Logan UT). For estrogen-free conditions the medium was phenol red-free and the serum was stripped of endogenous hormones by two incubations with dextran-coated charcoal (DCC). HUVEC cells were grown in F-12 K medium (ATCC) supplemented with 0.1 mg/ml heparin, 0.05 mg/ml endothelial cell growth supplement (ECGS; Cat N. 356006 BD Biosciences, Bedford, MA) and 10% FCS.

### BrdU and phosphohistone H3 assays

5-bromo-2'-deoxyuridine (BrdU or BrdUrd) incorporation in MCF-7 and BT-474 cells was calculated by dual staining with human CK18 (rabbit polyclonal AP1021; Calbiochem, La Jolla CA) and BrdU (mouse monoclonal #347580; Becton-Dickinson, San Jose CA), followed by red Alexa-555 goat anti-rabbit and green Alexa-488 goat anti-mouse antibodies (Invitrogen). Basal MDA-MB-231 and BT-20 were stained for human CD44 (rabbit monoclonal 1998–1; Epitomics) or CK5 (rabbit monoclonal 2290–1; Epitomics) instead of CK18. For cells grown in conditioned media, BrdU quantitation was performed by immunocytochemistry (ICC) using Image J software. For 3D cultures immunohistochemistry (IHC) was used. Total cells were quantified by counterstaining with blue fluorescent 4’-6-diamidino-2-phenylindole (DAPI). Antibody against phosphorylated Histone H3 (Rabbit pAb Millipore # 06–570) was used for IHC as described [30].

Proliferation rates were calculated by the ratio of BrdU + nuclei (green) to DAPI + nuclei (blue) in CK18+, CD44+ or CK5+ cells (red) using Image Pro 4.5 software (Media Cybernetics). Quantification of BrdU incorporation and phosphorylated Histone H3 assays were performed in a minimum of five different fields from three independent experiments.

### Conditioned media

For conditioned media, stock 5% FCS-containing MEM was removed from BJ3Z cells or NMFs growing in T-75 flasks at 70-80% confluence, and replaced with phenol red-free medium containing 5% DCC-stripped FCS for 24 h. Media from these cells were collected, filtered and added to breast cancer cells.

### 3D colonies

3D culture was performed as described [30]. Briefly, cells were trypsinized and resuspended in phenol red-free MEM containing 5% DCC-stripped serum. Breast cancer cells (10,000-50,000) and/or BJ3Z or NMF cells (50,000) were layered on Growth Factor Reduced (GFR)-phenol red-free Matrigel (hereafter called Matrigel) and cultures were maintained for ~7 days, adding fresh medium every 2 or 3 days. To calculate proliferation indices, 7 day-old colonies were incubated 1 hr. with 0.25 mg/ml BrdU in 5% phenol red-free, DCC-stripped serum.

### Expression profiling

Briefly, triplicate independent sets of mouse NMF and BJ3Z cells were grown in Matrigel as 3D colonies [30]. On day 4, colonies were incubated 5–10 min in dispase (Cat # 354235; BD Biosciences), cells were pelleted, resuspended in RLT buffer (QIAgen, Germany), homogenized (QIAshredder™; Cat # 79654, QIAgen) and RNA was extracted (RNEasy mini-kit™; Cat # 74104; QIAgen). Microarray analyses were performed at the University of Colorado Microarray Core using Affymetrix mouse gene ST 1.0 chips. Data were analyzed with Partek Genomics Suite Software 6.6 (Partek Ltd.). Raw data were normalized and analyzed to obtain significant differences by comparing NMF versus BJ3Z using *t*-test of unequal variances. The results were organized by p values (false discovery rates, FDR) of 0.05 and 0.01. This generated lists of 5,049 and 234 probesets that were significantly and differentially up- or down-regulated in NMF *vs.* BJ3Z cells respectively (Additional file 1: Table S1).

### Semi-quantitative RT-PCR

Briefly, total RNA samples (1–2 μg) were reverse transcribed using SuperScript II RT (Invitrogen), and cDNA samples were amplified by PCR using specific primers against FGF-5, IGF2BP3, IGF2BP1, PDGF-A, PDGF-B, DAPK-1, Caveolin-1, TGF-β1, TGF-β2, PDGFR-α and PDGFR-β (primer sequences are summarized in Additional file 1: Table S1); β-actin was used as a loading control. In all cases a PCR cycle versus intensity curve was obtained to confirm that amplified PCR products were in the linear area of the curve.

### PDGFR inhibition and recombinant PDGF

The PDGFR inhibitor Imatinib as its mesylate salt (IM; also Gleevec™, Glivec or STI571), was kindly provided by Novartis Pharma AG (Switzerland). Cells were incubated with 10 μM IM in sterile water for 6 h (tubule formation assay) or 48 h (3D proliferation assay). 3D colonies of MCF-7 cells were treated 48 h with 30 ng/ml recombinant human PDGF-BB (Cat N. 14-8501-80; eBioscience, San Diego, CA) [31].

### IHC for PDGFs & PDGFRs

IHCs were performed on paraffin sections (4–5 μm) of NMF and BJ3Z cells cultured in 3D colonies as described [30]. They used antibodies directed against PDGF-A (sc-7958; rabbit polyclonal, Santa Cruz), PDGF-B (sc-7878; rabbit polyclonal, Santa Cruz), PDGFR-α (AF1062; goat polyclonal, R&D systems) and PDGFR-β (AF1042; goat polyclonal, R&D systems). Briefly, sections were deparaffinized and antigen retrieval was performed in a pressure cooker (Bio-care Medical) at 20 psi for 5 min in citrate buffer (10 mM sodium citrate, 0.05% Tween-20, pH 6.0). Sections were blocked 30 min with 10% normal goat serum and primary antibodies were applied for 1 hr. Fluorescent secondary antibodies were: Alexa fluor 555 (red) donkey anti-goat IgG (1:300) and Alexa fluor 488 (green) goat anti- rabbit IgG (1:400; both Invitrogen). Cell nuclei were counterstained with 4-6-Diamidino-2-phenylindole (DAPI). Fluorescent images were obtained using a Nikon Eclipse E600 fluorescent microscope coupled to a RGB-MSC micro color camera, and Image Pro Plus software version 4.5 (Media Cybernetics, Silver Spring, MD).

### *In vitro*tubule formation

HUVEC cells were counted, pelleted and resuspended in phenol red-free medium containing 5% DCC-stripped serum; in F-12 K (a positive control containing Endothelial Cell Growth Supplement, BD Biosciences #356006); or in 48 hr-conditioned media from NMFs or BJ3Z cells grown in phenol red-free medium and 5% DCC-stripped serum. HUVEC were seeded in duplicate into 8-well chambers (40,000 cells per well) pre-coated with Matrigel. Cells were incubated for 24 h, with images captured every 2 hours. Cells were photographed in a Nikon Eclipse Ti microscope coupled to a Nikon digital camera DS-Qi1Mc. Images were analyzed and quantified in NIS-Elements AR software version 3.2 (Nikon Corp.). For quantification, the lengths of ten tubular structures/field were measured in duplicate per condition at 6 h of incubation. The experiment was repeated three independent times with similar results.

## Results

### Malignant BJ3Z stromal cells but not normal mouse mammary fibroblasts (NMFs) enhance proliferation of Luminal and HER2-positive breast cancer cells

BJ3Z are pure malignant mouse mammary stromal cells that arose after fusion of normal mouse stromal cells with human breast cancer cells xenografted into mammary glands [27]. When originally isolated, the cells contained both human and mouse chromosomes. However, after long-term culture all contaminating human chromosomes were lost as demonstrated by karyotype, single tandem repeat, and fluorescent *in situ* hybridization analyses [27]. The pure mouse malignant stromal cell line was called BJ3Z. The cells express the classical markers of activated fibroblasts when cultured in 3D [28] and have low proliferation rates. They were therefore used as a model of malignant stromal cells. In ovariectomized (ovx’d) immuno-compromised mice, BJ3Z cells are tumorigenic alone. In xenografts, BJ3Z cells enhance growth and angiogenesis of co-injected estrogen-dependent human MCF-7 breast cancer cells [28]. NMFs lack these properties.

**Figure 1:**
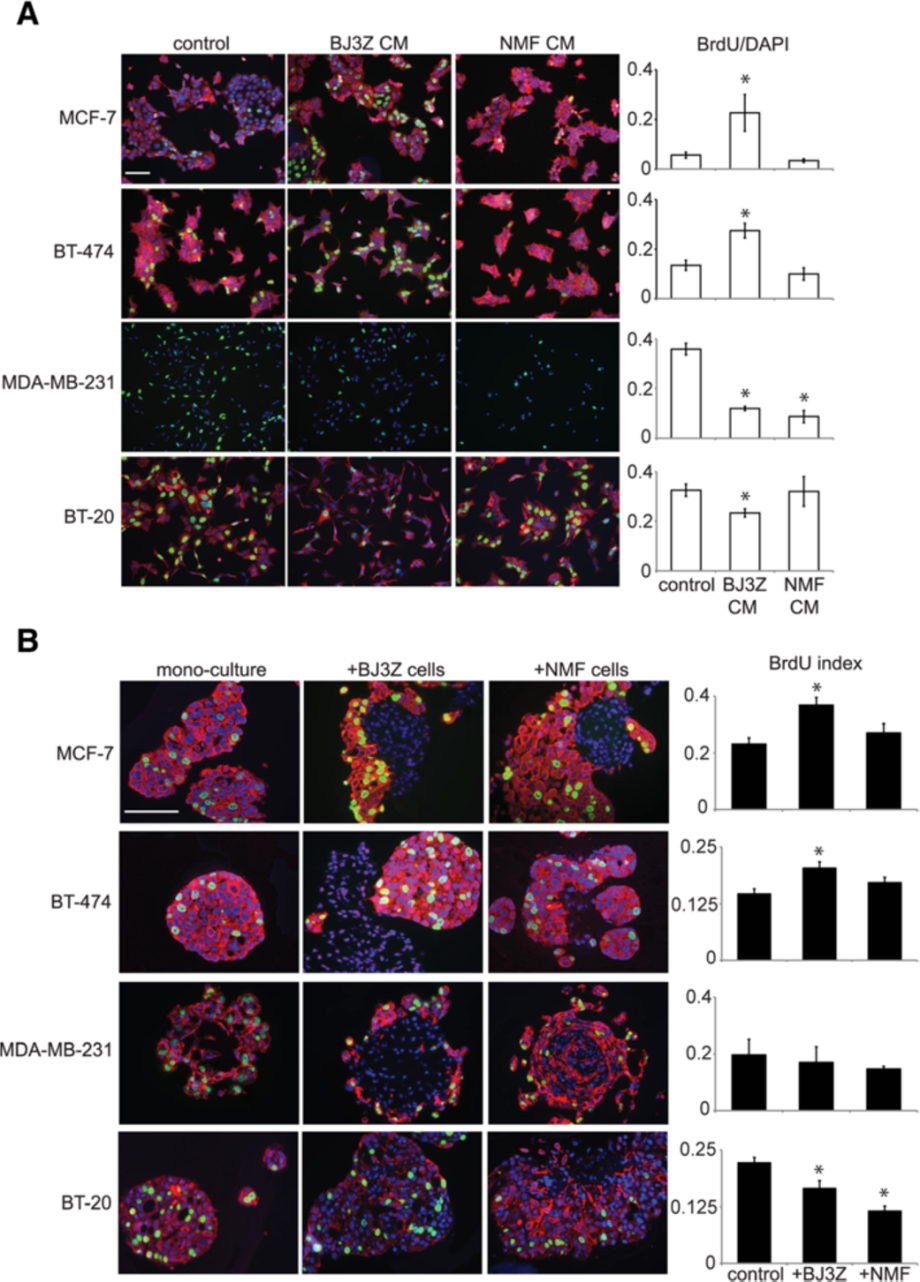
**Malignant stroma regulates proliferation of breast cancer cells** ***in vitro*** **. A**. MCF-7, BT-474, MDA-MB-231 and BT-20 breast cancer cells growing as monocultures in 2D on plastic, were incubated with control media, or media conditioned (CM) by BJ3Z or NMF stromal-like cells. Proliferation of the breast cancer cells was quantified by immunocytochemistry (ICC) for percent BrdU incorporation (green nuclei) compared to total cells (DAPI; blue), n = 3. Breast cancer cells were counterstained by ICC for Cytokeratin-18 (CK18, red). Bar: 100 μm. **B**. The same breast cancer cell lines were cultured in 3D on Matrigel and grown as colonies in monoculture or co-culture with BJ3Z or NMF cells. Colonies were stained by immunohistochemistry (IHC). Proliferation was quantified by counting BrdU incorporation (green nuclei) compared to total cells (DAPI, blue). To visualize the breast cancer cells they were counterstained with markers previously determined to be appropriate for each: CK18 (red) for MCF-7 and BT-474; CD44 (red) for MDA-MB-231; CK5 (red) for BT-20, n = 3. Bar: 100 μm *p < 0,05 by ANOVA followed by Tukey’s post-test.

Luminal breast cancer cells usually require estrogens for growth. To explain the non-estrogenic *in vivo* growth-promoting and angiogenic effects of BJ3Z cells on Luminal cells, BJ3Z were compared to NMFs using *in vitro* assays. First, we evaluated if a factor secreted by BJ3Z cells preferentially affects growth of breast cancer cells. For this media conditioned by growth of BJ3Z cells or NMFs, or control cell-free medium, were filtered and re-incubated for 2 days with cultured human breast cancer cells whose media had been removed. The tested cells represent the 3 main subtypes: MCF-7, Luminal A; BT-474, Luminal B/HER2+; MDA-MB-231 and BT-20, basal-like. One hour before the end of the study cells were pulsed with BrdU then processed for ICC. Figure 1A shows the 4 breast cancer cell lines exposed to conditioned media from control, BJ3Z cells or NMFs; stained for BrdU (green), Luminal CK18 or basal CK5 (red); and counterstained with DAPI (blue). (Note that BT-20 cells express CK5, classifying them as “triple negative” within the basal-like subtype.) Data were quantified as the BrdU/DAPI ratio (Figure 1A, right). With regard to the Luminal MCF-7 and BT-474 cells, BJ3Z-conditioned medium significantly increased proliferation, while control and NMF-conditioned media had no effect. However, effects were different on the basal-like cells, where surprisingly, BJ3Z-conditioned media strongly suppressed growth.

To confirm that a BJ3Z-secreted factor had differential effects on Luminal *vs.* basal-like breast cancer cells, we designed an assay in which malignant BJ3Z cells or normal NMFs were co-cultured with the 4 human breast cancer cells and grown as 3D Matrigel colonies *in vitro.* In 3D, cell growth more closely resembles *in vivo* conditions than growth on plastic [32, 33]. Mono-culture of the breast cancer cells served as controls (Figure 1B). Colonies were pulsed 1 hr. with BrdU at the end of the study and harvested for IHC [30]. Luminal cells were detected with CK18; basal-like cells with CD44 or CK5 (both in red); all cells were stained for BrdU incorporation (green) and counterstained with DAPI (blue). Proliferation of breast cancer cells was quantified as the BrdU/DAPI ratio (Figure 1B, right). Note that the morphology of colonies differs depending on the cell types in the mixtures, requiring that cell type-specific markers be used for quantitation. Interestingly, stromal cell proliferation appears to be negligible under 1 hr. BrdU incorporation conditions (Figure 1B). Similar quiescence is observed for stromal cells in 3D monoculture. However stromal cell proliferation can be detected if longer BrdU incubation conditions are tested (data not shown).

Quantification of BrdU in the breast cancer cells demonstrates that BJ3Z cells significantly enhance growth of co-cultured MCF-7 and BT-474 cells, but fail to do so or even suppress growth of basal-like cells. Effects of NMF are not significant. This confirms in an entirely independent manner that factor(s) produced by BJ3Z cells preferentially stimulate growth of Luminal breast cancer cells.

### Expression profiling and differentially expressed factors in BJ3Z *vs.*NMF

**Figure 2:**
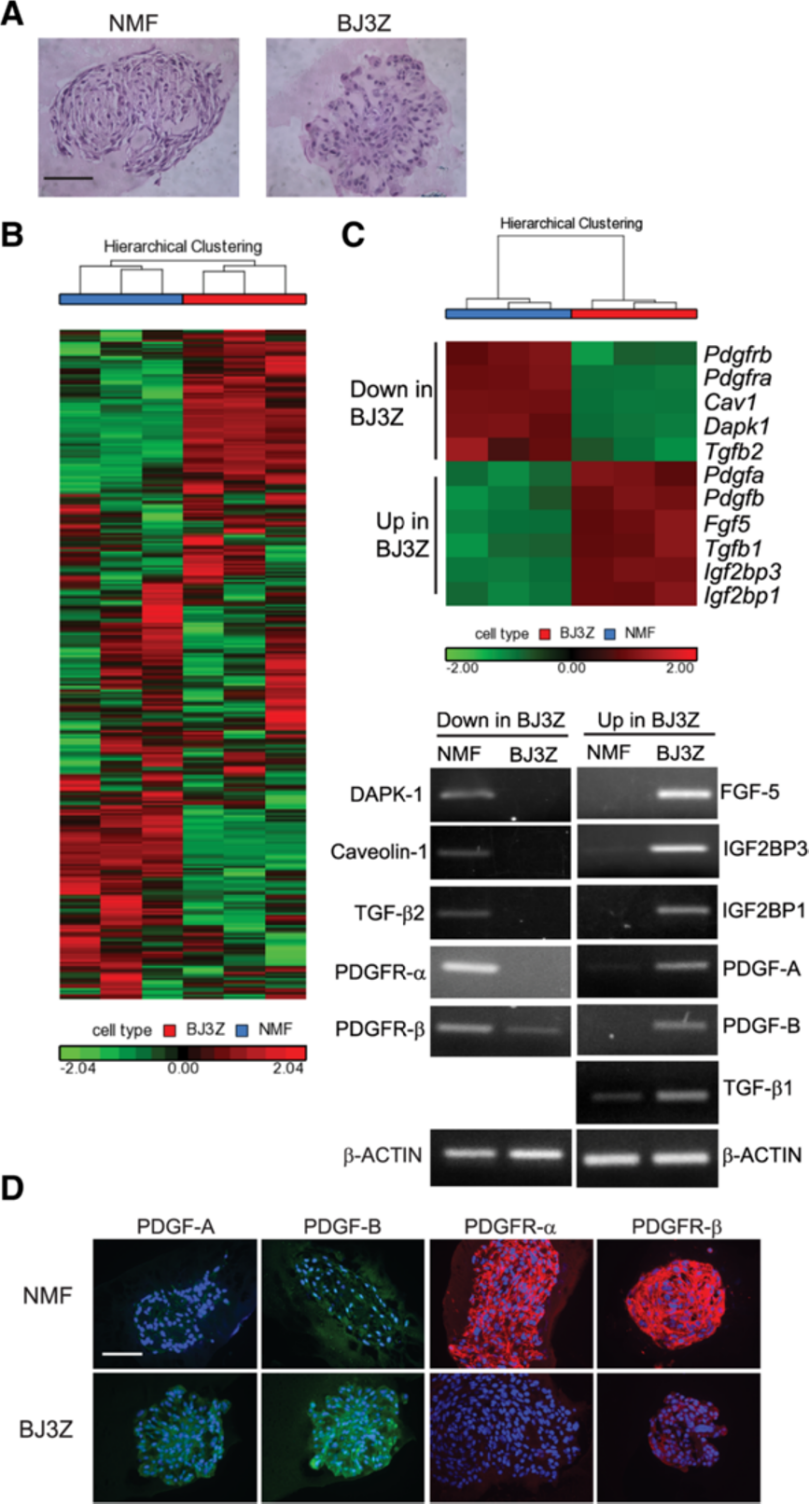
**Expression profiling of NMF and BJ3Z. A**. Hematoxylin & eosin stained clusters of NMF or BJ3Z cells cultured in Matrigel, bar: 100 μm. **B**. Hierarchical clustering of microarray data NMF (blue box) versus BJ3Z (red box), n = 3, using ANOVA p < 0.05. **C**. Sub-selected genes down (in green) or up-regulated (red) in BJ3Z cells by microarray were confirmed by semi-quantitative RT-PCR (lower panels), β-actin levels were used as loading control. **D**. NMF or BJ3Z cells in Matrigel were stained for members of the PDGF pathway: PDGF-A, PDGF-B, PDGFR-α and PDGFR-β.

To identify soluble factors elaborated by BJ3Z cells and responsible for Luminal cell proliferation, BJ3Z cells and NMFs were gene profiled after isolation from 3D colonies. Figure 2A shows that when grown in Matrigel under hormone-free conditions both BJ3Z cells and NMFs form 3D colonies that are similar in size and morphology. Cells were isolated from three independent sets of BJ3Z and NMF colonies, RNA was extracted and profiled using mouse GeneChip 1.0 ST Affymetrix arrays that probe more than 26,000 mouse transcripts. Figure 2B shows a hierarchical clustering of the triplicate sets, highlighting genes that are significantly (p < 0.05) and differentially up- (red) or down- (green) regulated in one cell type compared to the other. Differentially expressed genes were analyzed in detail and lists are available (Additional file 2: Table S2). Microarray results we confirmed on a select subset of secreted growth factors and other factors known to be commonly up- or downregulated in malignant stromal cells. These transcripts included: Death Associated Protein Kinase-1 (DAPK-1), Fibroblast Growth Factor-5 (FGF-5), Caveolin-1, Insulin-like Growth Factor-2 Binding Protein-1 and -3 (IGF2BP-1, IGF2BP-3), Transforming Growth Factor beta-1 and -2 (TGFβ-1, TGFβ-2), Platelet Derived Growth Factor-A and –B (PDGF-A, PDGF-B) and the PDGF Receptor-α and β (PDGFR-α, PDGFR-β). Differential expression of these transcripts in each cell type was confirmed by RT-PCR using β-actin as a loading control (Figure 2C, lower panel). Interestingly, the two main ligands of the PDGF pathway, PDGF-A and PDGF-B, which regulate cell growth and angiogenesis [13], are upregulated in BJ3Z cells while the two PDGF receptors, PDGFR-α and PDGFR-β are upregulated in NMFs. To confirm that transcript expression was reflected in protein levels, expression of the PDGF pathway members were analyzed by IHC (Figure 2D). This confirms that NMFs express the receptors but not the ligands; while BJ3Z cells express the ligands but not the receptors.

**Figure 3:**
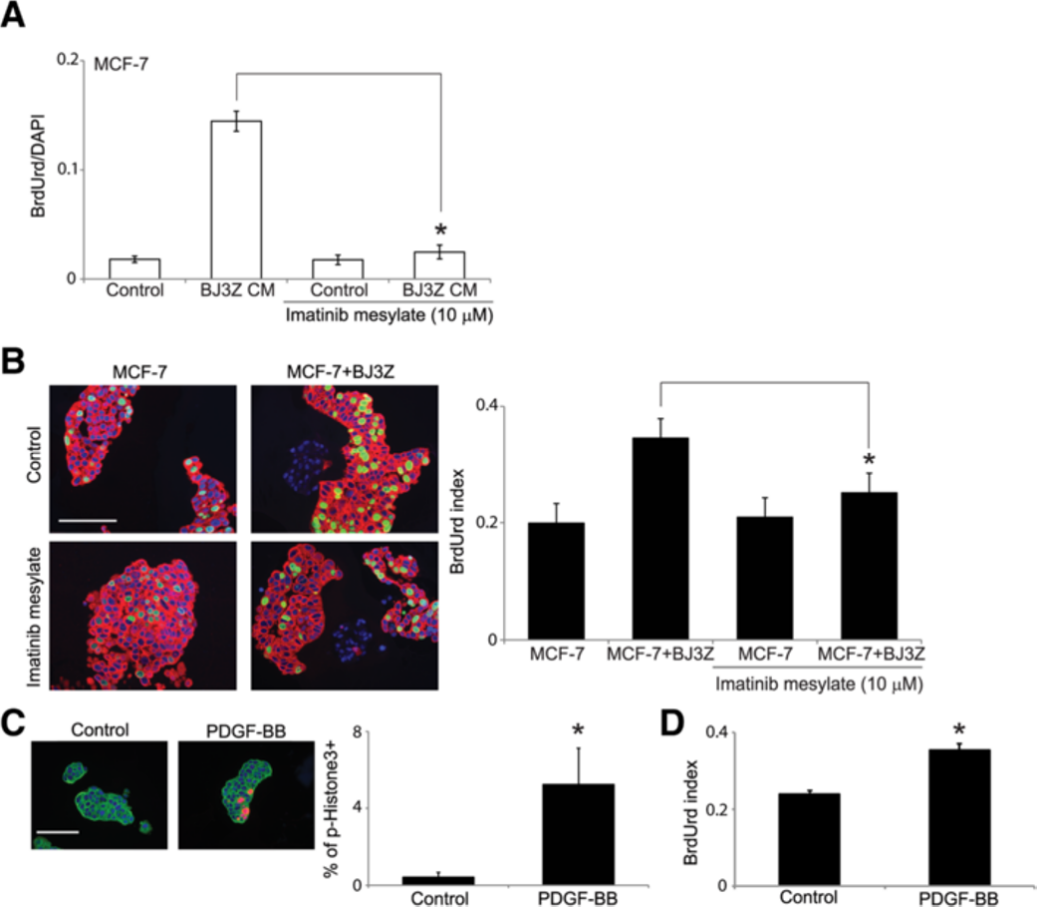
**The PDGF pathway regulates proliferation of Luminal MCF-7 cells** ***in vitro*** **. A**. MCF-7 cells in 2D on plastic were incubated with control or BJ3Z cell-conditioned media (CM) in the absence or presence of Imatinib Mesylate (IM). Percent of cells incorporating BrdU were compared to total cells (DAPI) to quantify proliferation; n = 3, *p < 0,05 by ANOVA followed by Tukey’s post-test. **B**. MCF-7 cells in 3D Matrigel monoculture, or co-cultured with BJ3Z cells, were treated with IM for 48 hr. BrdU incorporation (green nuclei) into MCF-7 cells was used to quantify proliferation compared to total cells (DAPI; blue nuclei). Cytokeratin-18 (red)-positivity mark the Luminal MCF-7 cells; BJ3Z cells are CK18-negative; n = 3; bar denotes 100 μm. Quantification of data is on the right with *p < 0,05 by ANOVA followed by Tukey’s post-test. **C.** MCF-7 cells in 3D Matrigel colonies were treated with recombinant PDGF-BB, 30 ng/ml for 48 hr. Phosphorylated-Histone H3 expression was quantified by IHC, n = 3, bar: 100 μm. *p < 0,05 by Student’s *t*-test. **D** Proliferation in PDGF-BB treated MCF-7 cells was quantified by BrdU incorporation, n = 3, *p < 0,05 by Student’s *t*-test.

Since expression profiling, RT-PCR and IHC demonstrated that PDGF ligands are produced by BJ3Z cells (Figure 2C and D), we evaluated the role of PDGF on Luminal cell proliferation (Figure 3). First we asked whether the Luminal-cell stimulatory factor(s) elaborated in BJ3Z-conditioned media involves PDGF by adding Imatinib Mesylate (IM), a potent tyrosine kinase inhibitor of PDGFRs. As shown in Figure 3A, on plastic, conditioned medium from BJ3Z cells increases proliferation of MCF-7 cells. Concomitant incubation of MCF-7 cells with BJ3Z-conditioned medium plus IM completely prevents the increased proliferation. IM was also able to block the direct effects of BJ3Z cells in 3D cultures. For this, MCF-7 cells were grown in 3D Matrigel cultures alone, or in co-culture with BJ3Z cells in the absence or presence of IM. BJ3Z cells increased proliferation of MCF-7 cells; and this effect was blocked by IM in a statistically significant manner (Figure 3B). Thus two different experimental models demonstrate that the proliferative effects of BJ3Z cells on MCF-7 cells are mediated by PDGF and can be suppressed by IM, presumably *via* PDGFR inhibition. The direct role of PDGF in promoting breast cancer cell growth was confirmed by treating MCF-7 cells grown in 3D-cultures with PDGF-BB; a ligand that binds all PDGFRs. PDGF-BB caused a significant increase in MCF-7 cell mitosis as measured by phosphorylation of Histone H3 (pHH3, Figure 3C). Additional studies probing BrdU incorporation into PDGF-BB-treated MCF-7 cells yielded similar results (Figure 3D). Thus, it is clear that PDGF secreted by malignant BJ3Z stromal cells increase proliferation of neighboring Luminal MCF-7 breast cancer cells *via* a pathway that does not involve estrogens.

### Tubule formation *in vitro*and the PDGF pathway

**Figure 4:**
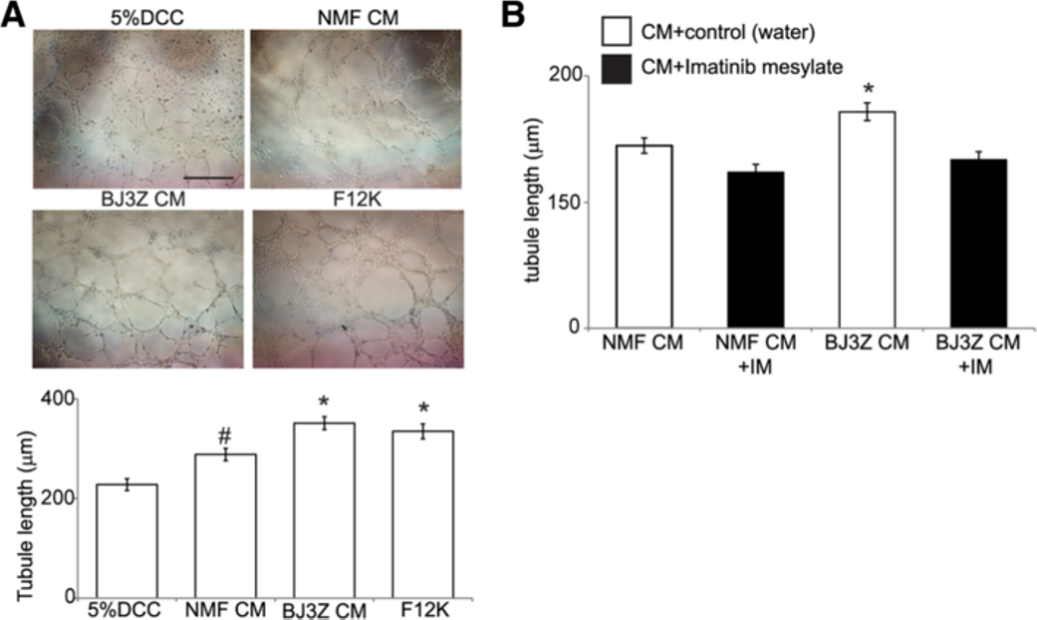
**The PDGF pathway regulates angiogenesis of HUVEC** ***in vitro*** **. A**. HUVEC were incubated with control (5 % DCC medium) or conditioned media (CM) obtained from either NMF or BJ3Z cells, F-12 K supplement was also used as a positive angiogenesis control; bar 500 μm. Tubule length quantification is shown on the lower panel, n = 3, * and # p < 0,05 by ANOVA followed by Tukey’s post-test versus control. **B** Imatinib mesylate (IM, black bars) was added to HUVEC; n = 3, *p < 0,05 by Student’s *t*-test versus control.

Besides being a potent mitogen, PDGF also induces angiogenesis [13]. Previously, we demonstrated that BJ3Z cells enhance angiogenesis *in vivo* [28]. Here we assess a possible mechanism by comparing the *in vitro* tubule formation properties of BJ3Z cells *vs.* NMFs using Human Umbilical Cord Vascular Endothelial Cells (HUVEC) [34, 35]. For this, HUVEC cells were seeded into chambers pre-coated with phenol red-free/growth factor reduced Matrigel and incubated with non-conditioned control medium, BJ3Z cell or NMF-conditioned media, or F12K medium. Chambers were photographed and the lengths of ten tubular structures/field in three independent experiments were measured per condition after 6 h of incubation. Compared to control medium, media conditioned by BJ3Z cells or NMFs induced a statistically significant increase in formation of HUVEC tubular structures (Figure 4A top). Quantification of the data (Figure 4A, bottom) showed that NMF-conditioned medium significantly increased tubule formation compared to control medium. BJ3Z-conditioned medium caused a significant further increase in tubule formation compared to either control or NMF-conditioned media. Tubule formation levels obtained with BJ3Z-conditioned medium were similar to ones induced by F-12 K – a supplement that contains pro-angiogenic factors. Finally, to test the influence of the PDGF pathway on tubule formation, BJ3Z cell- and NMF-conditioned media were co-incubated with HUVEC cells together with IM. Figure 4B shows that addition of IM blocked the increased tubule formation produced by BJ3Z- and NMF-conditioned media. We conclude that stromal elements, and in particular malignant stormal cells, not only influence proliferation of Luminal breast cancer cells, but also enhance tubule formation *in vitro*, an indicator of angiogenesis, *via* PDGF signaling.

## Discussion

### Malignant stroma

Luminal tumors represent the great majority of breast cancer cases. These malignancies of mammary gland epithelia are commonly treated with therapies that target ER, such as tamoxifen or fulvestrant, or therapies that target E biosynthesis, such as aromatase inhibitors. Despite their initial good prognosis and responses to these therapies, approximately one-third of Luminal cancers become resistant to therapies and recur either locally or at metastatic sites [3]. However, malignant epithelial cells do not exist in isolation within primary tissues or in recurrent or metastatic tissues. In all cases, the malignant epithelial cells are surrounded by and embedded in local tissue parenchyma. In normal tissues the cellular compartment located beneath the epithelium is the stroma, whose predominant cells type is the fibroblast. Stromal fibroblasts play a key role in a wide variety of normal biological processes. Additionally, multiple studies have shown that in cancers, carcinoma associated fibroblasts (CAFs; or “malignant stroma”) modify the behavior of the surrounding malignant epithelial cells by nurturing their growth and supporting their progression, invasion and metastasis [36–38]. CAFs do so by secreting a variety of growth factors, chemokines and proteases [39]. In breast cancers, malignant stroma also appears to play a role in resistance to therapies and tumor recurrence [7, 8]

### Proliferation

We previously demonstrated that a mouse mammary gland malignant stromal cell line that we derived and termed BJ3Z, enhances the *in vivo* proliferation of Luminal MCF-7 breast cancer cells [28] in the absence of estrogen supplementation. This is critical because Luminal tumors are classically thought to be estrogen-dependent. Our studies suggested that malignant stroma can replace estrogens as the proliferative agents. In the present studies we address mechanisms by which malignant stroma might do so. Importantly, as controls for the malignant BJ3Z mouse mammary stromal cells, we used normal mouse mammary fibroblasts (NMFs). Our *in vitro* results show that in the absence of estrogens, proliferation of Luminal MCF-7 cells is enhanced by BJ3Z cells but not by NMFs. Interestingly this proliferative effect is restricted to Luminal cells, since proliferation of basal-like MDA-MB-231 and BT-20 cells is unaffected by BJ3Z cells (Figure 1). A study using human reduction mammary fibroblasts immortalized with h-TERT also found increased levels of proliferation restricted to the Luminal breast cancer cell subtype [40].

Our results demonstrate that the increased Luminal breast cancer cell proliferation is dependent on PDGF ligands secreted by the malignant stromal cells. Since the effects of PDGF are observed *in vitro*, we speculate that this represents paracrine signaling that targets PDGF receptors present on the breast cancer cells. Since PDGF receptors are expressed on breast cancers [41] and breast cancer cell lines including MCF-7 cells [42] this is a likely mechanism for the proliferative effect we observe. The PDGF pathway has been implicated in proliferation and angiogenesis in many malignancies. Indeed, PDGF and/or PDGF receptors are expressed in a variety of malignancies including melanoma [43] colorectal [44], lung [45], colon [46], cervical [47] and breast cancer [48]; in all cases PDGF signaling plays a key role in tumor growth and progression.

Breast cancer patients have higher levels of PDGF and VEGF in both tumors and sera compared to normal controls [49]. PDGF-B expression predicts micrometastases in peripheral blood, bone marrow and sentinel lymph nodes of breast cancer patients [50], and plays a key role in initiating the desmoplastic response [24, 25]. A recent study suggests that the PDGF pathway also plays a key role in therapeutic resistance. Proliferation of estrogen-deprived MCF-7 cells can be restored by overexpressing PDGFR-β. Additionally, ER + breast tumors of menopausal women undergoing neoadjuvant therapy with an aromatase inhibitor exhibit increased PDGFR-β expression [51]. These data suggest that the PDGF pathway may be upregulated in hormone-resistant Luminal tumors making it a potential target for reversing resistance.

### Angiogenesis

Solid tumors require blood vessels to grow beyond a few millimeters in diameter. Angiogenesis is the mechanism by which these new blood vessels are generated and it is therefore one of the “hallmarks of cancer” [52, 53]. CAFs are the main source of VEGF; a potent morphogen for endothelial cells and the classical factor implicated in angiogenesis [54]. VEGF also increases vascular leaking and is a chemoattractant for circulating endothelial progenitor cells [55]. Orimo and colleagues [38] showed that co-injection of human CAFs and Ras-mutant MCF-7 cells generated highly vascularized tumors and proposed a mechanism involving secretion of Stromal-Derived Factor-1 (SDF-1) by CAFs into the bloodstream and mobilization and recruitment of circulating endothelial progenitor cells to form new blood vessels within tumors. We previously reported that BJ3Z cells enhance angiogenesis when co-injected with MCF-7 cells and that BJ3Z cells express SDF-1 in 3D cultures and tumors [28]. We therefore speculated that SDF-1 was the angiogenic factor. Here we show additionally, that BJ3Z-conditioned media enhance tubule formation of HUVEC cells in a PDGF-dependent manner. Thus it appears that at least two factors secreted by BJ3Z cells, and perhaps more, can induce angiogenesis; and highlights the complexity of this process. PDGF-induced angiogenesis has been described. Overexpression of PDGF-B in keratinocytes [56] and gliomas [57] increases angiogenesis. In gliomas, PDGF-B stimulates VEGF, which enhances proliferation of endothelial cells and the recruitment of vascular smooth muscle cells (pericytes) to form new blood vessels. It is possible that both paracrine and autocrine loops can explain the PDGF effects we observe, since Luminal cells also secrete VEGF [58]. Additionally, during angiogenesis, the PDGF pathway regulates recruitment of pericytes into newly formed vessels, and endothelial cell-derived PDGF-B stimulates their proliferation and migration *via* PDGFRβ [59, 60]. During angiogenesis, pericyte recruitment stabilizes immature endothelial tubes in a process that also requires PDGF-B [61, 62]. Conversely PDGFR blockade causes pericyte detachment and blood vessel regression thereby decreasing tumor growth [63–65]. Therefore there is the possibility that key targets of stromal PDGF are perivascular smooth muscle cells.

### Targeting PDGF in breast cancers

IM is a tyrosine kinase inhibitor originally developed as an inhibitor of the BCR-ABL kinase which causes chronic myeloid leukemia (CML). It has been successfully used for the treatment of CML, as well as gastrointestinal stromal tumors, which are typically driven by gain-of-function mutations in the KIT tyrosine kinase receptor [66]. IM also targets PDGF receptors [67] and recent studies have evaluated its use in breast cancers. These show that IM inhibits breast cancer cell proliferation by inhibiting telomerase activity [68]; inhibits phosphorylation of PDGFR-β in MDA-MB-231 breast cancer cells [69]; and increases sensitivity of breast cancer cell lines to radiotherapy [31] *in vitro*. In mice, IM suppresses orthotopic MDA-MB-231 tumor growth [69] and decreases osteolytic lesions and metastatic tumor burden generated by these cells. In combination with Paclitaxel, IM inhibits growth, PDGFR phosphorylation, cell proliferation, and tumor microvessel density of MDA-MB-435 metastases in bone [70]. As a result, IM has been evaluated for use in metastatic breast cancer patients. In a phase II trial IM in combination with Capecitabine in 19 such patients (68% ER + and/or PR+) was well tolerated, but response rates were not different than with Capecitabine alone [71]. A second phase II trial evaluated IM in combination with Docetaxel in 37 patients with metastatic disease (49% ER+). This study reported poor tolerance and low objective response with the combinations, compared to Docetaxel alone [72]. It is likely that anti-PDGF receptor therapies, like all targeted therapies, will require that patients be selected on the basis of clearly defined criteria, including the presence of PDGR receptors on their tumor cells. Moreover, our studies suggest that such drugs may be especially useful as second-line therapy in Luminal disease with demonstrated hormone resistance.

## Conclusions

Malignant stromal cells enhance proliferation of human Luminal breast cancer cells in the absence of estrogens. They also stimulate *in vitro* tubule formation; a marker of angiogenesis. Both mechanisms could be linked to endocrine therapy resistance. We speculate that stroma-directed therapies, including anti-PDGFR agents like Imatinib, may be useful in combination with other therapies for treatment of Luminal cancers.

## Electronic supplementary material

Additional file 1: Table S1: RT-PCR primers. Description of data: Table summarizes the sequence of all RT-PCR primers used in this study. (DOC 41 KB)

Additional file 2: Table S2: Differentially expressed genes BJ3Z vs NMF (FDR < 0.05). Description of data: Table summarizes the differentially expressed genes when comparing normal mammary fibroblasts (NMFs) versus malignant BJ3Z stromal cells using a false discovery rate 0.05. (PDF 549 KB)
