# Supplementary material for: Malignant stroma increases luminal breast cancer cell proliferation and angiogenesis through platelet-derived growth factor signaling
Source: BMC Cancer. 2014 Oct 1;14:735. doi: 10.1186/1471-2407-14-735 (PMC4190420; doi:10.1186/1471-2407-14-735)
Supplement: Supplementary file 1 — Additional file 1: Table S1: RT-PCR primers. Description of data: Table summarizes the sequence of all RT-PCR primers used in this study. (DOC 41 KB) [file 12885_2014_4913_MOESM1_ESM.doc]

Supplementary table 1. RT-PCR primers

| Symbol | orientation | Sequence (5’-3’) |
| --- | --- | --- |
| PDGFA | Forward | 5’-agcgactcttggagatagactcc-3’ |
|  | Reverse | 5’-gctggtgttacaacagccagtg-3’ |
| PDGFB | Forward | 5’-tgacgttctggccaatgtaggt-3’ |
|  | Reverse | 5’-ccatcgggtagaaccgcaaaag-3’ |
| PDGFRα | Forward | 5’-actggaagcttggggcttactt-3’ |
|  | Reverse | 5’-cagctcacttcactctccccaa-3’ |
| PDGFRβ | Forward | 5’-gctgttagtggtgaaggtaggc-3’ |
|  | Reverse | 5’-tggtaggtacacacttcaggca-3’ |
| Caveolin-1 | Forward | 5’-cttgaggtaatgttcttgctgg-3’ |
|  | Reverse | 5’-acagacatgtcttggaacacacag-3’ |
| IGF2BP1 | Forward | 5’-tagtgccaagagaccagacc-3’ |
|  | Reverse | 5’-gagatcaggattcctcactgg-3’ |
| IGF2BP3 | Forward | 5’-acacacttgagatcagaccatac-3’ |
|  | Reverse | 5’-aagtccgatgtttccacagtctg-3’ |
| FGF-5 | Forward | 5’-ccagtgaagtacagactgaag-3’ |
|  | Reverse | 5’-ctcggtgatctgaagagatgag-3’ |
| TGF-β1 | Forward | 5’-gactctccacctgcaagac-3’ |
|  | Reverse | 5’-catagatggcgttgttgcg-3’ |
| TGF-β2 | Forward | 5’-ctacatcgatagcaaggttgtg-3’ |
|  | Reverse | 5’-ctggactgttgtgactccag-3’ |
| DAPK-1 | Forward | 5’-gacggtgaagcactacctgag-3’ |
|  | Reverse | 5’-agactcaggtctgacgcatg-3’ |
| β-Actin | Forward | 5’-accaactgggacgatatggagaaga-3’ |
|  | Reverse | 5’-tacgaccagaggcatacagggacaa-3’ |
