# Supplementary material for: Malignant stroma increases luminal breast cancer cell proliferation and angiogenesis through platelet-derived growth factor signaling
Source: BMC Cancer. 2014 Oct 1;14:735. doi: 10.1186/1471-2407-14-735 (PMC4190420; doi:10.1186/1471-2407-14-735)
Supplement: Supplementary file 2 — Additional file 2: Table S2: Differentially expressed genes BJ3Z vs NMF (FDR < 0.05). Description of data: Table summarizes the differentially expressed genes when comparing normal mammary fibroblasts (NMFs) versus malignant BJ3Z stromal cells using a false discovery rate 0.05. (PDF 549 KB) [file 12885_2014_4913_MOESM2_ESM.pdf]

Supplementary Table 2: Differentially expressed genes BJ3Z vs NMF (FDR<0.05)

Gene symbol	RefSeq	p-value	t	Mean (BJ3Z)	Mean (NMF)	MeanRatio (BJ3Z/NMF)	MeanDiff (BJ3Z-NMF)	FoldChange (BJ3Z/NMF)
Gm773	BC147685	7.26E-06	30.0968	9.88618	3.37658	91.114	6.5096	91.114
Msln	NM_018857	2.25E-06	40.357	11.2595	5.08913	72.022	6.17037	72.022
Xist	NR_001463	1.31E-06	46.2043	9.42412	3.33347	68.1503	6.09065	68.1503
Ott	NM_011022	2.19E-06	40.6338	9.46352	3.58117	58.988	5.88235	58.988
Ott	NM_011022	1.51E-06	44.5813	9.47381	3.59315	58.9189	5.88066	58.9189
Gm15107	NM_001081648	3.41E-06	36.3759	9.47436	3.59908	58.6994	5.87527	58.6994
Nes	NM_016701	2.45E-06	39.5268	9.88876	4.02924	58.0618	5.85952	58.0618
Luzp4	NM_001114383	9.36E-07	50.2852	9.41785	3.60756	56.114	5.81029	56.114
Ott	NM_011022	1.76E-06	42.9548	9.37662	3.59285	55.0917	5.78376	55.0917
Ott	NM_011022	2.53E-06	39.2134	9.36843	3.70373	50.7276	5.6647	50.7276
Ott	NM_011022	2.53E-06	39.2134	9.36843	3.70373	50.7276	5.6647	50.7276
Gatm	NM_025961	5.68E-06	32	9.6378	4.05152	48.044	5.58628	48.044
Ott	NM_011022	3.22E-06	36.8882	9.32421	3.73869	48.0187	5.58552	48.0187
Ott	NM_011022	2.09E-06	41.1301	9.17178	3.67726	45.0831	5.49452	45.0831
Blnk	NM_008528	1.16E-05	26.7438	9.54217	4.05566	44.8337	5.48651	44.8337
Ott	NM_011022	2.89E-06	37.9285	9.17135	3.69852	44.4106	5.47283	44.4106
Ott	NM_011022	2.15E-06	40.8193	8.96061	3.57299	41.8634	5.38762	41.8634
Aspa	NM_023113	4.12E-05	19.4451	9.30055	4.15038	35.5105	5.15017	35.5105
Cyp2c55	NM_028089	4.64E-06	33.6813	8.71073	3.59462	34.6818	5.11611	34.6818
Cbr2	NM_007621	0.000518	10.2126	10.6068	5.62802	31.5325	4.97877	31.5325
Gm5930	ENSMUST00000111864	4.93E-05	18.5856	8.07048	3.18053	29.6498	4.88995	29.6498
Gm7609	NM_001081746	3.86E-06	35.2581	9.20729	4.50251	26.0784	4.70478	26.0784
Itga3	NM_013565	9.01E-06	28.5052	9.58377	4.89606	25.7715	4.68771	25.7715
Cfh	NM_009888	0.001273	8.08243	10.8113	6.19881	24.463	4.61253	24.463
Gm7609	NM_001081746	1.13E-05	26.932	8.89621	4.37034	23.0367	4.52587	23.0367
Gm5622	NM_001013816	4.25E-05	19.2926	7.96786	3.52293	21.7801	4.44494	21.7801
Sox2	NM_011443	4.52E-05	19.0026	8.84674	4.40212	21.7753	4.44462	21.7753

Ces1f	NM_144930	5.42E-05	18.1457	8.24545	3.83288	21.2969	4.41257	21.2969
2610305D13Rik	NM_145078	2.48E-05	22.0919	7.18163	2.80971	20.7051	4.37191	20.7051
Aim2	NM_001013779	5.19E-05	18.3524	8.0953	3.74923	20.3375	4.34607	20.3375
	---	1.15E-05	26.7948	9.0128	4.68955	20.0183	4.32325	20.0183
Gm5622	NM_001013816	0.000124	14.7044	7.86654	3.56164	19.7653	4.3049	19.7653
Herc3	NM_028705	1.43E-05	25.3822	9.42444	5.21771	18.4652	4.20673	18.4652
Rex2	NM_009051	3.09E-06	37.2977	9.20052	5.03243	17.9771	4.16809	17.9771
Csprs	NM_033616	2.58E-05	21.8771	8.4457	4.2779	17.9735	4.1678	17.9735
Klhl30	NM_027551	8.10E-05	16.3944	8.89642	4.74162	17.8123	4.1548	17.8123
Crmp1	NM_007765	2.59E-05	21.8561	8.97037	4.85144	17.3748	4.11893	17.3748
Tspan15	NM_197996	8.95E-06	28.5598	9.02092	4.91402	17.2307	4.10691	17.2307
Cadm1	NM_207675	5.92E-05	17.7498	9.00621	4.9009	17.2115	4.1053	17.2115
Gbp9	NM_172777	0.000871	8.92543	8.98347	4.8889	17.084	4.09457	17.084
Csprs	NM_033616	1.74E-05	24.1711	8.25286	4.18929	16.7208	4.06357	16.7208
Prune2	NM_181348	9.95E-06	27.8036	8.54078	4.55915	15.7976	3.98163	15.7976
Samd12	NM_177225	4.45E-05	19.0717	8.72975	4.77083	15.5508	3.95892	15.5508
Xlr	NM_011725	0.000522	10.1935	7.00046	3.04832	15.478	3.95215	15.478
Slxl1	NM_029181	6.27E-05	17.4927	7.13373	3.20701	15.2076	3.92672	15.2076
Rgs17	NM_001161822	5.44E-05	18.128	8.27048	4.38765	14.7519	3.88283	14.7519
Gcnt1	NM_173442	0.000161	13.77	8.51572	4.63328	14.748	3.88244	14.748
Plscr2	NM_001195084	0.000103	15.4194	7.75378	3.88071	14.6524	3.87307	14.6524
Itgb8	NM_177290	5.31E-05	18.2408	8.20808	4.33858	14.6162	3.8695	14.6162
Fam134b	NM_001034851	1.46E-05	25.2417	9.24649	5.46098	13.7896	3.78551	13.7896
Itga6	NM_008397	2.45E-05	22.1714	8.27407	4.50118	13.6695	3.77289	13.6695
Vmn2r50	ENSMUST00000086298	4.67E-05	18.8423	8.48883	4.72481	13.5857	3.76402	13.5857
3830403N18Rik	NM_027510	0.000306	11.693	6.79949	3.03766	13.5651	3.76183	13.5651
Rims2	NM_053271	5.73E-06	31.941	8.22409	4.47648	13.4321	3.74761	13.4321
Gm10058	NM_001109969	0.000116	14.9692	7.31564	3.60406	13.1008	3.71158	13.1008
Scrn1	NM_027268	0.000174	13.5045	10.1916	6.48139	13.0882	3.71019	13.0882
Mustn1	NM_181390	0.001386	7.90419	10.8906	7.19837	12.9266	3.69227	12.9266

Pde3b	NM_011055	2.12E-05	22.9942	9.13237	5.45117	12.8278	3.68121	12.8278
Zfp772	BC023179	1.59E-05	24.7151	8.36801	4.69233	12.7787	3.67567	12.7787
Crabp2	NM_007759	0.000299	11.7624	9.62704	5.95321	12.7624	3.67383	12.7624
Tmem74	NM_175502	9.71E-06	27.979	7.51722	3.86118	12.606	3.65603	12.606
Epb4.1l4a	NM_013512	1.48E-06	44.853	8.28537	4.632	12.5826	3.65336	12.5826
Gm10058	NM_001109969	8.92E-05	15.9995	7.6614	4.0104	12.562	3.651	12.562
Gm10058	NM_001109969	8.92E-05	15.9995	7.6614	4.0104	12.562	3.651	12.562
Lama5	NM_001081171	4.78E-07	59.4823	9.19832	5.58398	12.2469	3.61434	12.2469
Gabrb3	NM_008071	2.02E-05	23.2854	7.90799	4.2984	12.2066	3.60959	12.2066
C130026I21Rik	NM_175219	3.93E-05	19.6803	7.93363	4.32669	12.1842	3.60694	12.1842
4930528F23Rik	NM_029197	0.002387	6.84257	6.39718	2.80106	12.0931	3.59611	12.0931
Pax3	NM_008781	8.24E-06	29.1586	8.60785	5.03846	11.8712	3.56939	11.8712
Gm10058	NM_001109969	8.50E-05	16.1982	7.59464	4.03725	11.7729	3.55739	11.7729
Gm10058	NM_001109969	8.50E-05	16.1982	7.59464	4.03725	11.7729	3.55739	11.7729
Col18a1	NM_009929	7.89E-07	52.4736	9.86335	6.30602	11.7723	3.55732	11.7723
Osbpl3	NM_027881	0.000179	13.4158	9.45104	5.91173	11.6263	3.53931	11.6263
Adh7	NM_009626	0.000641	9.66601	11.5288	8.00619	11.4921	3.52257	11.4921
Aldh3a1	NM_007436	0.001271	8.08537	12.0718	8.55346	11.4585	3.51835	11.4585
Tinagl1	NM_023476	9.61E-06	28.0492	9.76805	6.27067	11.2932	3.49738	11.2932
Kcnn4	NM_008433	2.52E-05	22.0237	8.87866	5.39019	11.2237	3.48847	11.2237
Pde1b	NM_008800	0.000344	11.3429	9.15644	5.67831	11.1435	3.47813	11.1435
Cdh6	NM_007666	0.000118	14.8972	7.9585	4.48323	11.1214	3.47527	11.1214
Prune2	NM_181348	3.42E-05	20.3862	8.44423	4.96968	11.1159	3.47456	11.1159
Cdh26	NM_198656	0.00067	9.5541	7.64636	4.17323	11.1049	3.47312	11.1049
Gm10058	NM_001109969	8.64E-05	16.1306	7.6025	4.138	11.0387	3.4645	11.0387
Gm10058	NM_001109969	8.64E-05	16.1306	7.6025	4.138	11.0387	3.4645	11.0387
Gm10058	NM_001109969	8.64E-05	16.1306	7.6025	4.138	11.0387	3.4645	11.0387
Gm10058	NM_001109969	8.64E-05	16.1306	7.6025	4.138	11.0387	3.4645	11.0387
Gm10058	NM_001109969	8.64E-05	16.1306	7.6025	4.138	11.0387	3.4645	11.0387
	---	0.00257	6.70825	7.17055	3.70867	11.0187	3.46188	11.0187
Igf2bp3	NM_023670	2.58E-06	38.9942	7.94166	4.48535	10.9762	3.4563	10.9762

2310002L13Rik	ENSMUST00000025390	6.86E-05	17.1019	7.00606	3.5627	10.8782	3.44336	10.8782
Ralgps2	NM_001159965	6.71E-07	54.6462	7.76347	4.3604	10.5785	3.40307	10.5785
Ctsc	NM_009982	0.002978	6.44768	9.34183	5.941	10.5622	3.40083	10.5622
Pip5k1b	NM_008846	4.51E-05	19.0066	7.52129	4.1604	10.2737	3.36089	10.2737
1700008I05Rik	NM_027952	0.000352	11.2789	6.83255	3.47929	10.2195	3.35326	10.2195
	---	0.001257	8.11046	7.0736	3.72387	10.1945	3.34972	10.1945
Igf2bp1	NM_009951	5.20E-05	18.3369	7.83081	4.51089	9.98609	3.31992	9.98609
Mtap2	NM_001039934	7.42E-06	29.9321	7.54563	4.27095	9.67779	3.27468	9.67779
Cmbl	NM_181588	4.27E-05	19.2768	7.36176	4.09317	9.63704	3.26859	9.63704
2810047C21Rik1	NR_015598	0.000203	12.9893	7.94242	4.67424	9.63435	3.26819	9.63435
	---	0.002124	7.05922	6.20282	2.95435	9.5036	3.24847	9.5036
Col8a1	NM_007739	2.90E-06	37.8725	9.25361	6.00692	9.49188	3.24669	9.49188
Snora2b	NR_034052	0.00044	10.6511	7.88319	4.64326	9.44749	3.23993	9.44749
Hpse	NM_152803	8.38E-05	16.2558	9.32975	6.10022	9.37965	3.22953	9.37965
AU021092	NM_001033220	0.000113	15.083	7.41454	4.18775	9.36182	3.22679	9.36182
Ces1g	NM_021456	1.40E-07	80.898	7.54618	4.33586	9.25557	3.21032	9.25557
Prrg4	NM_178695	0.006732	5.1527	7.43166	4.22827	9.2112	3.20339	9.2112
1700049E17Rik1	ENSMUST00000095985	0.000745	9.29513	6.90163	3.71056	9.13289	3.19107	9.13289
4930503E14Rik	NM_029131	3.42E-05	20.3817	7.44721	4.27186	9.03393	3.17535	9.03393
Bcar3	NM_013867	7.71E-06	29.6472	8.31054	5.14405	8.9786	3.16649	8.9786
Gm5169	NM_001040669	0.000165	13.6876	6.93	3.76464	8.97153	3.16535	8.97153
Arhgef33	NM_001145452	0.000114	15.0485	7.2644	4.1261	8.80484	3.1383	8.80484
Csprs	NM_033616	0.00012	14.8387	8.48902	5.36229	8.73451	3.12673	8.73451
Cav2	NM_016900	8.70E-06	28.7592	9.8073	6.684	8.71376	3.12329	8.71376
Pdgfb	NM_011057	0.000273	12.0379	8.47619	5.35867	8.67895	3.11752	8.67895
Scx	NM_198885	0.000104	15.3761	9.882	6.78575	8.5519	3.09625	8.5519
Ttll7	NM_027594	1.67E-05	24.4276	8.2177	5.12457	8.53349	3.09314	8.53349
Pcolce2	NM_029620	0.000263	12.1507	8.21413	5.12286	8.52246	3.09127	8.52246
Eps8l2	NM_133191	4.61E-06	33.7248	8.83185	5.7467	8.48643	3.08516	8.48643
Pla2g7	NM_013737	0.000147	14.103	8.55077	5.48431	8.37717	3.06646	8.37717

Robo2	NM_175549	0.000682	9.51063	8.47502	5.41927	8.3152	3.05575	8.3152
	---	3.11E-06	37.2258	9.79592	6.74405	8.29284	3.05187	8.29284
Ppm1e	NM_177167	8.59E-06	28.8487	7.92428	4.87456	8.2805	3.04972	8.2805
Rundc3a	NM_016759	0.000233	12.5335	9.1745	6.12805	8.26173	3.04644	8.26173
Eya1	NM_010164	0.000151	14.0032	7.22708	4.21701	8.05605	3.01007	8.05605
Afap1l2	NM_146102	0.000716	9.3924	9.35565	6.36283	7.96027	2.99282	7.96027
Pla2g5	NM_001122954	0.000312	11.6347	8.27086	5.28135	7.94205	2.98951	7.94205
A630033H20Rik	NM_175442	0.000146	14.1317	6.50303	3.51718	7.92187	2.98584	7.92187
Gprc5a	NM_181444	0.002021	7.1531	7.61784	4.64813	7.83376	2.96971	7.83376
Slc22a23	NM_001033167	0.000149	14.0524	8.11479	5.15942	7.75633	2.95537	7.75633
Lef1	NM_010703	0.000203	12.9877	8.56944	5.61479	7.75245	2.95465	7.75245
Foxd1	NM_008242	4.28E-07	61.1549	7.7945	4.84006	7.75128	2.95443	7.75128
Murc	NM_026509	2.08E-06	41.1552	7.89902	4.95638	7.68819	2.94264	7.68819
1700049E17Rik1	ENSMUST00000100701	0.000825	9.05221	6.70439	3.76956	7.64668	2.93483	7.64668
Lgr6	NM_001033409	7.22E-05	16.8803	7.98365	5.04889	7.6463	2.93476	7.6463
Arl4d	NM_025404	0.000174	13.5089	8.80729	5.89451	7.53069	2.91278	7.53069
	---	0.003956	5.96949	6.58083	3.67398	7.4998	2.90685	7.4998
Naip2	NM_010872	0.000436	10.6777	7.60484	4.69851	7.49705	2.90632	7.49705
Apol9b	NM_173743	0.000308	11.6708	8.40558	5.51849	7.39774	2.88708	7.39774
Gbp4	NM_008620	2.80E-05	21.4339	7.99935	5.11368	7.39048	2.88567	7.39048
Fgf5	NM_010203	7.48E-05	16.7314	7.89545	5.03552	7.25983	2.85994	7.25983
Atp6v0e2	NM_133764	3.42E-05	20.386	9.29258	6.45175	7.1643	2.84083	7.1643
Nuak2	NM_001195025	3.19E-05	20.7418	8.16903	5.33029	7.15394	2.83874	7.15394
Exoc6	NM_175353	0.000128	14.6054	7.99515	5.16016	7.13537	2.83499	7.13537
Il18rap	NM_010553	0.000124	14.7156	7.90669	5.08094	7.08985	2.82575	7.08985
Kcnk5	NM_021542	0.000642	9.6623	7.41828	4.59859	7.0601	2.81969	7.0601
Tlr6	NM_011604	0.000181	13.3671	7.3363	4.52857	7.0018	2.80773	7.0018
Gbp8	NM_029509	0.000611	9.7853	6.90935	4.11941	6.91603	2.78995	6.91603
Arhgef12	NM_027144	0.000129	14.5816	7.02244	4.23265	6.91526	2.78978	6.91526
Chd7	NM_001081417	0.000468	10.4854	6.3898	3.60935	6.87067	2.78045	6.87067

Atp8a1	NM_001038999	4.50E-05	19.0215	7.15747	4.3863	6.82659	2.77117	6.82659
Sp140	NM_001013817	0.000353	11.2683	7.62579	4.85913	6.80532	2.76666	6.80532
2810030E01Rik	NM_028317	0.000843	9.00252	7.50424	4.74403	6.77497	2.76022	6.77497
Dusp18	NM_173745	0.000291	11.842	8.79213	6.04088	6.733	2.75125	6.733
Megf10	NM_001001979	0.000167	13.6365	8.41095	5.66181	6.7232	2.74915	6.7232
Ephb6	NM_001146351	5.51E-05	18.0741	9.04277	6.29476	6.7179	2.74801	6.7179
4930503E14Rik	NM_029131	0.001843	7.33035	6.51227	3.76651	6.70743	2.74576	6.70743
Spag1	NM_012031	4.38E-05	19.148	7.2208	4.47578	6.704	2.74502	6.704
	---	5.78E-05	17.8551	7.9998	5.26235	6.66888	2.73744	6.66888
4930420K17Rik	BC147127	0.001254	8.11502	8.05364	5.31889	6.65641	2.73474	6.65641
Slc25a13	NM_015829	5.05E-05	18.4727	8.73992	6.01715	6.60142	2.72278	6.60142
	---	0.006426	5.22028	6.029	3.31024	6.5831	2.71877	6.5831
Tdrkh	NM_028307	0.001037	8.52941	7.23563	4.52313	6.55455	2.7125	6.55455
A530040E14Rik	ENSMUST0000009686 2	0.004962	5.60948	6.89193	4.19188	6.49826	2.70005	6.49826
A530040E14Rik	ENSMUST0000009686 2	0.004962	5.60948	6.89193	4.19188	6.49826	2.70005	6.49826
	---	0.0045	5.76235	7.28849	4.60234	6.43594	2.68615	6.43594
Prss23	NM_029614	4.65E-05	18.8692	9.44353	6.76432	6.40506	2.67921	6.40506
Nlrp4f	NM_175290	0.001482	7.76568	5.7842	3.10783	6.39245	2.67637	6.39245
Pkp2	NM_026163	0.000795	9.14073	7.53543	4.86623	6.36075	2.6692	6.36075
Ttll7	NM_027594	3.26E-05	20.6347	8.62601	5.96229	6.33663	2.66372	6.33663
Tcfap2a	NM_011547	0.000363	11.1875	7.00842	4.34535	6.33383	2.66308	6.33383
Slc16a13	NM_172371	5.83E-06	31.803	9.16736	6.51138	6.30277	2.65599	6.30277
Abcb1b	NM_011075	0.002497	6.76075	8.70878	6.05608	6.28844	2.6527	6.28844
Plekha1	NM_133942	7.68E-06	29.6782	10.6351	7.98282	6.28653	2.65226	6.28653
Avpi1	NM_027106	2.17E-05	22.8529	8.73532	6.08521	6.27714	2.65011	6.27714
A530040E14Rik	ENSMUST0000009686 2	0.004835	5.64962	6.92853	4.28352	6.25501	2.64501	6.25501
A530040E14Rik	ENSMUST0000009686 2	0.004835	5.64962	6.92853	4.28352	6.25501	2.64501	6.25501
Rragb	NM_001004154	0.001231	8.15507	7.20288	4.57657	6.17447	2.62631	6.17447
Gpc1	NM_016696	0.001192	8.22354	10.068	7.44414	6.16402	2.62387	6.16402

Dlx1	NM_010053	0.000914	8.81353	7.95339	5.33908	6.12328	2.61431	6.12328
Fads3	NM_021890	0.000536	10.1236	10.352	7.74333	6.09932	2.60865	6.09932
Vtcn1	NM_178594	4.44E-06	34.0472	7.4993	4.91132	6.01255	2.58798	6.01255
Crip2	NM_024223	0.001913	7.25806	10.2258	7.6379	6.01204	2.58785	6.01204
Fam105a	BC052328	1.93E-06	41.9502	6.97742	4.39625	5.98426	2.58117	5.98426
Mapk12	NM_013871	8.57E-05	16.1633	8.81897	6.24171	5.96806	2.57726	5.96806
Chd7	NM_001081417	7.96E-05	16.4665	6.10792	3.53147	5.96474	2.57646	5.96474
Jup	NM_010593	0.000555	10.0329	9.56413	6.99687	5.92684	2.56726	5.92684
Ccnd1	NM_007631	0.003135	6.35877	10.4658	7.90302	5.90847	2.56278	5.90847
Napsa	NM_008437	0.000326	11.5022	7.16104	4.60267	5.89041	2.55837	5.89041
Als2cl	NM_001146059	0.00019	13.2095	7.32742	4.77575	5.86312	2.55167	5.86312
Insig1	NM_153526	0.00033	11.4636	11.1073	8.57115	5.80032	2.53613	5.80032
G930009F23Rik	AK145170	2.75E-05	21.5323	7.01399	4.47929	5.79458	2.5347	5.79458
Trp53i11	NM_001025246	0.000202	13.0035	8.43368	5.90092	5.78678	2.53276	5.78678
Gm904	BC147620	6.80E-05	17.1362	6.32437	3.80973	5.71456	2.51464	5.71456
Lyz2	NM_017372	0.000608	9.8002	6.41893	3.91663	5.66588	2.5023	5.66588
Ctnna2	NM_001109764	8.98E-05	15.9715	7.14928	4.66306	5.60309	2.48622	5.60309
Nefm	NM_008691	0.001203	8.20386	6.87172	4.39788	5.55521	2.47384	5.55521
Pde2a	NM_001143848	8.25E-06	29.1487	7.86091	5.38874	5.54879	2.47217	5.54879
Chd7	NM_001081417	2.86E-05	21.3235	6.71919	4.2477	5.54615	2.47149	5.54615
	---	0.003275	6.28393	5.95576	3.48443	5.54555	2.47133	5.54555
A530032D15Rik	NM_213615	1.70E-05	24.2939	9.08945	6.62191	5.53098	2.46754	5.53098
Hspa4l	NM_011020	0.000306	11.6912	9.55142	7.08397	5.53067	2.46745	5.53067
	---	0.005741	5.3871	6.72613	4.25907	5.52919	2.46707	5.52919
	---	0.002714	6.61079	6.14353	3.68561	5.49422	2.45791	5.49422
Slc24a3	NM_053195	0.002007	7.16614	8.01753	5.56125	5.488	2.45628	5.488
	---	0.000678	9.52552	8.69771	6.24453	5.47623	2.45318	5.47623
C630004H02Rik	BC024617	0.000178	13.4188	7.99469	5.5471	5.45507	2.4476	5.45507
Bpgm	NM_007563	0.000201	13.0242	9.15729	6.71448	5.43699	2.44281	5.43699
Gm5168	NM_001025607	6.15E-05	17.5809	6.15365	3.72571	5.38123	2.42794	5.38123
	---	0.001773	7.40641	8.18135	5.76151	5.35113	2.41984	5.35113

Gpr30	NM_029771	0.001831	7.34336	6.95409	4.53643	5.34301	2.41765	5.34301
Klhdc8a	NM_144810	0.001548	7.67734	6.86279	4.45609	5.3026	2.4067	5.3026
Epha2	NM_010139	0.003414	6.21372	8.79294	6.41373	5.20251	2.37921	5.20251
Spata13	NM_001033272	1.64E-05	24.5236	6.5225	4.14744	5.18756	2.37506	5.18756
	---	0.006712	5.15689	8.42929	6.05776	5.17488	2.37153	5.17488
Lfng	NM_008494	0.000432	10.703	7.33856	4.96843	5.16989	2.37013	5.16989
Sema3b	NM_001042779	0.000216	12.7809	8.03634	5.67324	5.14473	2.3631	5.14473
Nr3c2	NM_001083906	0.001284	8.06517	6.20495	3.84984	5.11632	2.35511	5.11632
Mllt3	NM_027326	0.000174	13.5098	8.21251	5.86396	5.0931	2.34854	5.0931
Scube3	NM_001004366	0.000205	12.9584	7.36571	5.01941	5.08518	2.3463	5.08518
Gm13235	ENSMUST0000010573<br>6	0.001138	8.3248	6.60463	4.26253	5.07041	2.3421	5.07041
Gm13235	ENSMUST0000010573<br>6	0.001138	8.3248	6.60463	4.26253	5.07041	2.3421	5.07041
Epb4.1l5	NM_145506	0.000409	10.8538	7.7065	5.37445	5.0352	2.33205	5.0352
Nhedc1	NM_028946	0.00029	11.853	5.7887	3.47035	4.98762	2.31835	4.98762
Mmp28	NM_080453	0.000128	14.6011	8.38212	6.06529	4.98236	2.31683	4.98236
	---	0.004623	5.7199	6.77179	4.45497	4.98232	2.31682	4.98232
Swap70	NM_009302	0.000304	11.7114	9.11374	6.79702	4.98197	2.31672	4.98197
Vdr	NM_009504	0.003733	6.06463	8.56914	6.25698	4.96625	2.31216	4.96625
Apol9a	NM_173786	0.00067	9.55677	7.65576	5.34615	4.9575	2.30961	4.9575
Chd7	NM_001081417	0.000145	14.1431	6.40574	4.10099	4.94079	2.30474	4.94079
Naip5	NM_010870	0.002345	6.87484	5.75136	3.44666	4.94065	2.3047	4.94065
Cp	NM_001042611	0.001951	7.22078	11.9197	9.62505	4.90644	2.29468	4.90644
Kif21a	NM_001109040	1.66E-05	24.4369	8.95372	6.665	4.88622	2.28872	4.88622
	---	0.00013	14.5408	8.4954	6.20676	4.88595	2.28864	4.88595
Anxa3	NM_013470	0.00264	6.66007	9.9935	7.7051	4.88515	2.2884	4.88515
Sp140	NM_001013817	0.0011	8.39867	6.73423	4.44598	4.88462	2.28825	4.88462
	---	0.00101	8.58848	5.65484	3.36801	4.87983	2.28683	4.87983
Aif1l	NM_145144	6.60E-05	17.2679	8.10346	5.84128	4.79715	2.26218	4.79715
Zdhhc2	NM_178395	0.002608	6.68195	7.87651	5.61761	4.78629	2.25891	4.78629
Cdc42ep1	NM_027219	0.000346	11.3318	8.19183	5.93666	4.77389	2.25517	4.77389

Slc22a4	NM_019687	0.003537	6.15406	8.1964	5.94188	4.77178	2.25453	4.77178
Pthlh	NM_008970	0.00106	8.48105	7.87683	5.63125	4.74229	2.24558	4.74229
Cdkn2b	NM_007670	0.001711	7.47691	9.03278	6.79222	4.72583	2.24057	4.72583
Chd7	NM_001081417	4.55E-05	18.9687	5.71658	3.47897	4.71617	2.23761	4.71617
Pdlim1	NM_016861	0.001514	7.72191	9.85221	7.61609	4.71127	2.23612	4.71127
Tiparp	NM_178892	0.000353	11.2682	10.229	7.99501	4.70444	2.23402	4.70444
Mobkl2b	ENSMUST00000102975	0.000126	14.6627	6.24534	4.01989	4.67659	2.22546	4.67659
Ptprf	NM_011213	0.000383	11.0381	8.53166	6.32116	4.62838	2.21051	4.62838
	---	0.002224	6.97314	6.88263	4.67317	4.62502	2.20946	4.62502
Naip6	NM_010871	0.002761	6.5805	5.25086	3.04296	4.62002	2.2079	4.62002
Cdca7l	NM_146040	0.000109	15.21	8.32848	6.12403	4.60899	2.20445	4.60899
H2-gs10	NM_001143689	0.000337	11.4066	7.33461	5.15119	4.54231	2.18343	4.54231
Fbxo36	NM_025386	6.84E-05	17.114	9.41133	7.23143	4.53122	2.1799	4.53122
Gjb4	NM_008127	2.37E-05	22.3597	6.94069	4.76524	4.51724	2.17544	4.51724
Hap1	NM_010404	0.001995	7.17842	7.04902	4.88285	4.4883	2.16617	4.4883
Ptpn22	NM_008979	0.00069	9.4828	5.8997	3.74009	4.46793	2.15961	4.46793
Rab3ip	NM_001003950	3.15E-06	37.0977	8.77532	6.61808	4.4606	2.15724	4.4606
Efna1	NM_010107	0.001682	7.51022	8.07286	5.91743	4.45503	2.15544	4.45503
Arhgap18	NM_176837	0.000801	9.12313	8.67731	6.5238	4.44907	2.1535	4.44907
Cd59a	NM_001111060	0.000163	13.7219	7.59109	5.44619	4.42263	2.1449	4.42263
Ncf4	NM_008677	0.000408	10.8606	6.73002	4.58548	4.42149	2.14453	4.42149
Syt7	NM_173068	0.000262	12.1657	7.78725	5.64509	4.41423	2.14216	4.41423
Rap1gap2	NM_001015046	0.000885	8.89028	7.7131	5.57172	4.41182	2.14137	4.41182
Mageb1	NM_010759	0.000371	11.1264	5.89381	3.75806	4.39465	2.13575	4.39465
Mageb1	NM_010759	0.000371	11.1264	5.89381	3.75806	4.39465	2.13575	4.39465
Slc25a23	NM_025877	1.84E-06	42.4276	7.26616	5.13067	4.39386	2.13549	4.39386
Cdkn2aipnl	NM_029976	0.000443	10.6326	9.43276	7.30229	4.37861	2.13047	4.37861
Metrn	NM_133719	0.000174	13.5038	8.35931	6.23109	4.37178	2.12822	4.37178
Zfp600	NM_001177546	0.001929	7.24274	4.68201	2.5539	4.37144	2.12811	4.37144
Sp110	NM_175397	0.000361	11.2076	7.5949	5.46724	4.37009	2.12766	4.37009

B3gnt2	NM_016888	0.000848	8.98863	8.43974	6.32145	4.34177	2.11828	4.34177
Gm10058	NM_001109969	0.000281	11.9482	5.42016	3.31923	4.28989	2.10094	4.28989
Gm13242	NM_001103158	0.001465	7.79013	5.32954	3.22881	4.28927	2.10073	4.28927
Pcdh17	NM_001013753	0.001925	7.24633	7.43555	5.33873	4.27767	2.09682	4.27767
Plin4	NM_020568	0.000493	10.3443	7.33268	5.24151	4.26096	2.09118	4.26096
Zdhhc16	NM_023740	5.36E-06	32.4787	9.28105	7.19222	4.25402	2.08883	4.25402
Rundc3b	NM_198620	0.000758	9.2546	7.4236	5.33794	4.24468	2.08566	4.24468
Lctl	NM_145835	0.00067	9.55643	6.36948	4.29275	4.2185	2.07673	4.2185
Acy3	NM_027857	0.000246	12.3644	7.57536	5.49872	4.21822	2.07663	4.21822
Plcd3	NM_152813	0.000402	10.8983	8.09768	6.02894	4.19521	2.06874	4.19521
Gm10058	NM_001109969	0.002278	6.92837	5.94675	3.88241	4.18244	2.06434	4.18244
Arhgap22	NM_153800	0.000189	13.2287	7.78292	5.71949	4.1798	2.06343	4.1798
Gstm7	NM_026672	0.000483	10.3967	7.60177	5.54286	4.1667	2.05891	4.1667
Rassf7	NM_025886	0.005126	5.55926	7.29814	5.24171	4.15957	2.05644	4.15957
Mmp9	NM_013599	0.000879	8.90549	7.96967	5.91401	4.15733	2.05566	4.15733
Glcci1	NM_133236	0.000733	9.33544	8.41651	6.36175	4.15474	2.05476	4.15474
Ahr	NM_013464	0.006385	5.2298	8.98699	6.93707	4.14085	2.04993	4.14085
Fnbp1l	NM_001114665	0.000305	11.6971	9.51258	7.47719	4.09933	2.03539	4.09933
P2ry14	NM_133200	0.00369	6.08364	7.3866	5.35489	4.0889	2.03171	4.0889
Rin1	NM_145495	0.000549	10.0624	8.54671	6.52431	4.06257	2.02239	4.06257
Klf5	NM_009769	0.000416	10.8038	8.49884	6.47689	4.06133	2.02195	4.06133
Chd7	NM_001081417	6.63E-06	30.7922	5.47896	3.45843	4.05735	2.02054	4.05735
Lphn3	NM_198702	0.00088	8.90276	7.14228	5.12362	4.05206	2.01865	4.05206
Cyp7a1	NM_007824	0.001562	7.65857	5.44711	3.43601	4.03089	2.0111	4.03089
Gm5665	BC032201	0.000944	8.74027	5.38205	3.37228	4.02719	2.00977	4.02719
Galnt10	NM_134189	7.10E-06	30.2643	8.80966	6.80516	4.01248	2.00449	4.01248
	---	0.004078	5.91994	9.2217	7.22791	3.98284	1.9938	3.98284
Edil3	NM_001037987	0.000798	9.13172	8.48844	6.49629	3.97829	1.99215	3.97829
St3gal5	NM_011375	2.10E-05	23.0583	10.5206	8.53062	3.97225	1.98996	3.97225
	---	0.001826	7.3487	8.82841	6.84321	3.95918	1.9852	3.95918
Met	NM_008591	0.003377	6.23182	9.60659	7.62522	3.94868	1.98137	3.94868

Lgals2	NM_025622	0.000121	14.8234	6.14505	4.16448	3.94649	1.98057	3.94649
Tjp1	NM_009386	7.86E-05	16.5203	10.6055	8.62578	3.94406	1.97968	3.94406
Gm14005	NR_028590	0.00099	8.63277	7.22074	5.24314	3.93837	1.9776	3.93837
Rrad	NM_019662	0.002975	6.44947	8.44517	6.46771	3.93801	1.97747	3.93801
Tmeff2	NM_019790	0.000131	14.5277	7.6606	5.68386	3.93601	1.97673	3.93601
Rasgef1a	NM_027526	5.04E-05	18.4885	6.82741	4.85171	3.93318	1.9757	3.93318
Macrod2	NM_001013802	2.07E-05	23.1296	7.38871	5.41512	3.92742	1.97358	3.92742
Pdlim1	NM_016861	0.001379	7.91514	9.68864	7.71508	3.92736	1.97356	3.92736
Mfap3l	NM_027756	0.000583	9.90392	7.85907	5.88627	3.92529	1.9728	3.92529
Bhlhe41	NM_024469	0.003605	6.12248	7.88281	5.91064	3.92356	1.97216	3.92356
	---	0.000399	10.9258	5.09478	3.13353	3.89398	1.96124	3.89398
Hagh	NM_024284	9.18E-05	15.8844	10.2306	8.27288	3.88447	1.95772	3.88447
Tnnt2	NM_001130174	0.000177	13.4439	7.45335	5.49982	3.87322	1.95353	3.87322
Mfsd6	NM_133829	0.000119	14.8809	6.36177	4.41113	3.86546	1.95064	3.86546
Chd7	NM_001081417	0.002221	6.97535	6.39068	4.44205	3.86009	1.94863	3.86009
Ogdhl	NM_001081130	8.40E-05	16.2465	8.25315	6.30705	3.85331	1.9461	3.85331
Itgb7	NM_013566	0.000314	11.613	7.17353	5.23651	3.82914	1.93702	3.82914
Dysf	NM_021469	0.000598	9.83876	8.58829	6.65518	3.81878	1.93311	3.81878
	---	0.004703	5.69299	7.47136	5.53848	3.81818	1.93288	3.81818
Fmnl3	NM_011711	3.77E-05	19.8953	9.43468	7.5027	3.81577	1.93197	3.81577
Chd7	NM_001081417	0.002717	6.60883	5.98691	4.05989	3.80268	1.92702	3.80268
	---	0.000169	13.5995	8.71712	6.7909	3.80058	1.92622	3.80058
Tuft1	NM_011656	0.000666	9.56956	8.84488	6.92649	3.78	1.91839	3.78
Gm14137	NM_001039223	0.000299	11.7617	6.35147	4.4403	3.76117	1.91118	3.76117
Tpd52	NM_009412	0.001928	7.24352	7.76494	5.85712	3.7524	1.90782	3.7524
Mobkl2b	NM_178061	0.00019	13.2078	7.08303	5.17575	3.75102	1.90728	3.75102
	---	0.00174	7.44316	9.43965	7.53739	3.73797	1.90226	3.73797
Tmem38a	NM_144534	7.48E-05	16.7273	7.13299	5.23309	3.73187	1.8999	3.73187
Gimap9	NM_174960	0.000474	10.4512	5.64865	3.74949	3.72997	1.89916	3.72997
Ecscr	NM_001033141	0.001438	7.82821	7.73126	5.84244	3.70333	1.88882	3.70333
Il12rb1	NM_008353	0.000608	9.79723	7.25787	5.37488	3.68839	1.88299	3.68839

	---	0.006392	5.2282	9.13369	7.25261	3.6835	1.88108	3.6835
Jub	NM_010590	0.000857	8.96307	8.41087	6.53033	3.68212	1.88054	3.68212
Nupr1	NM_019738	0.001107	8.38517	11.5231	9.6445	3.67705	1.87855	3.67705
Macrod2	NM_001013802	0.004159	5.88842	5.122	3.24764	3.6664	1.87436	3.6664
Cadm4	NM_153112	0.000153	13.9471	7.04721	5.17512	3.6606	1.87208	3.6606
Pipox	NM_008952	0.000768	9.22344	6.67129	4.79926	3.66048	1.87203	3.66048
Fbxl2	NM_178624	0.001761	7.4199	8.24153	6.37475	3.64717	1.86678	3.64717
Bicd1	NM_009753	6.54E-05	17.3053	6.51708	4.65375	3.63846	1.86333	3.63846
Ivd	NM_019826	7.11E-05	16.944	9.8438	7.98501	3.62703	1.85879	3.62703
Macrod2	NM_001013802	0.005859	5.35676	5.14675	3.29324	3.61379	1.85351	3.61379
Scara5	NM_028903	0.000103	15.4171	8.52678	6.67977	3.59753	1.84701	3.59753
Nlk	NM_008702	7.78E-05	16.5662	8.93106	7.08641	3.59167	1.84465	3.59167
Llgl2	NM_145438	2.01E-05	23.3152	6.7811	4.93663	3.59119	1.84446	3.59119
Dmpk	NM_032418	0.000409	10.8513	10.1272	8.2838	3.58857	1.84341	3.58857
Sp110	NM_175397	6.82E-05	17.1228	7.0597	5.21666	3.58767	1.84305	3.58767
Slc24a6	NM_133221	0.000586	9.8924	9.06568	7.223	3.58676	1.84268	3.58676
Bid	NM_007544	0.000495	10.334	8.74417	6.90202	3.58545	1.84215	3.58545
Mcam	NM_023061	0.001448	7.81304	8.28107	6.43996	3.58283	1.8411	3.58283
Zfp239	NM_001001792	0.001157	8.28837	7.61705	5.777	3.58022	1.84005	3.58022
Mgat4a	NM_173870	0.0003	11.7494	6.44263	4.6053	3.57349	1.83733	3.57349
Nup210	NM_018815	4.81E-06	33.3767	6.77024	4.93628	3.56514	1.83396	3.56514
Car5b	NM_181315	0.000954	8.71598	9.13806	7.30496	3.56304	1.83311	3.56304
1810011O10Rik	NM_026931	0.005464	5.46177	8.96631	7.1451	3.53377	1.82121	3.53377
Mllt4	NM_010806	5.04E-05	18.4849	9.05145	7.23498	3.52218	1.81647	3.52218
Card6	NM_001163138	0.000463	10.5141	6.06658	4.25713	3.50508	1.80945	3.50508
Chd7	NM_001081417	0.000328	11.4856	6.20028	4.39177	3.5028	1.80851	3.5028
Chd7	NM_001081417	0.000292	11.8297	6.27793	4.46985	3.50176	1.80808	3.50176
Ppp1r14c	NM_133485	0.000835	9.02478	7.33283	5.52496	3.50126	1.80787	3.50126
Cspg4	NM_139001	0.00029	11.8539	7.30496	5.50066	3.49259	1.8043	3.49259
	---	0.00389	5.99669	4.94573	3.14344	3.48772	1.80229	3.48772
BC049715	NM_178776	0.00018	13.3832	6.31637	4.51805	3.47816	1.79832	3.47816

Zbp1	NM_021394	0.002079	7.09926	7.36679	5.57017	3.47405	1.79662	3.47405
Sema3f	NM_011349	0.00011	15.1642	8.22221	6.42589	3.47332	1.79632	3.47332
Eno3	NM_007933	0.000394	10.9597	8.31429	6.52062	3.46696	1.79367	3.46696
Bcl2	NM_009741	0.000249	12.324	7.67549	5.88326	3.46349	1.79223	3.46349
Dock8	NM_028785	0.000177	13.4482	6.44817	4.65824	3.45798	1.78993	3.45798
Sp110	NM_175397	0.000161	13.7686	5.12057	3.33542	3.44653	1.78515	3.44653
Sp110	NM_175397	0.000161	13.7686	5.12057	3.33542	3.44653	1.78515	3.44653
Trim30a	NM_009099	0.000395	10.9524	8.7105	6.92648	3.44385	1.78402	3.44385
Chd7	NM_001081417	0.000169	13.5995	6.10721	4.32493	3.43969	1.78228	3.43969
Magi3	NM_133853	0.000322	11.5373	8.4958	6.71616	3.43339	1.77963	3.43339
Gm5622	NM_001013816	0.002877	6.50787	5.945	4.16573	3.43252	1.77927	3.43252
Cbx5	NM_007626	4.19E-05	19.3656	9.59016	7.81147	3.43114	1.77869	3.43114
Cacnb3	NM_007581	2.69E-05	21.6624	9.42961	7.65376	3.42438	1.77584	3.42438
Slc6a6	NM_009320	0.000141	14.2412	10.8272	9.05183	3.42329	1.77538	3.42329
Cuedc1	NM_198013	0.000188	13.2348	8.46533	6.69111	3.42054	1.77422	3.42054
	---	0.00189	7.2817	8.04283	6.26977	3.41778	1.77306	3.41778
Rabl2	NM_026817	0.003254	6.29502	6.54562	4.77279	3.41723	1.77283	3.41723
Fancl	NM_025923	0.000176	13.4589	8.13482	6.3629	3.41508	1.77192	3.41508
Myo10	NM_019472	0.00098	8.65657	10.3025	8.53291	3.40947	1.76955	3.40947
Acsbg1	NM_053178	9.40E-05	15.7912	7.22736	5.45841	3.40806	1.76895	3.40806
Baalc	NM_080640	3.55E-05	20.1936	6.90909	5.1411	3.40579	1.76799	3.40579
	---	0.000587	9.88816	8.84143	7.07568	3.40052	1.76576	3.40052
Ankrd5	NM_175667	0.000149	14.0371	5.7959	4.03411	3.3912	1.7618	3.3912
Itga7	NM_008398	0.000337	11.4064	7.40257	5.64296	3.38607	1.75961	3.38607
Pcdh19	NM_001105245	0.001399	7.88539	6.57426	4.81533	3.38446	1.75893	3.38446
Ap3b2	NM_021492	0.00063	9.71083	7.02543	5.26887	3.37893	1.75656	3.37893
Elavl2	NM_207685	4.35E-05	19.1901	6.28273	4.52762	3.37551	1.7551	3.37551
Agrn	NM_021604	5.13E-05	18.4045	8.10446	6.351	3.37166	1.75346	3.37166
Nnt	NR_003544	0.000191	13.1876	10.3949	8.64188	3.37065	1.75303	3.37065
4632427E13Rik	NR_015510	4.50E-05	19.0221	8.17273	6.42087	3.36791	1.75185	3.36791
Rnls	NM_001167818	0.000579	9.92171	7.66837	5.91685	3.36712	1.75152	3.36712

Clu	NM_013492	0.000507	10.2681	7.50109	5.75059	3.36474	1.7505	3.36474
Ablim1	NM_178688	0.00057	9.96413	8.20345	6.4531	3.36439	1.75034	3.36439
Epb4.1	NM_183428	9.46E-06	28.1594	7.02028	5.27141	3.36094	1.74886	3.36094
Nfkbie	NM_008690	0.004296	5.83618	7.81748	6.07744	3.34042	1.74003	3.34042
9930111J21Rik2	NM_173434	0.000519	10.2089	6.29298	4.5535	3.33914	1.73948	3.33914
Rad18	NM_001167730	0.003207	6.3196	8.13907	6.40141	3.33494	1.73766	3.33494
Ly6c2	NM_001099217	0.006854	5.12673	8.71924	6.98284	3.33204	1.7364	3.33204
Rgnef	NM_012026	0.000528	10.1609	7.70276	5.96756	3.32926	1.7352	3.32926
Lrrk1	NM_146191	0.000282	11.9379	8.55972	6.82524	3.3276	1.73448	3.3276
Arhgdib	NM_007486	0.000694	9.47049	7.92694	6.19283	3.32674	1.73411	3.32674
Sync	NM_023485	0.001336	7.98165	8.30275	6.56944	3.32491	1.73332	3.32491
Mrps30	NM_021556	1.33E-05	25.8296	8.06324	6.33048	3.32364	1.73276	3.32364
Eml5	NM_001081191	2.67E-05	21.6928	6.37561	4.64309	3.32308	1.73252	3.32308
Fcgr4	NM_144559	0.000866	8.93945	5.88182	4.1494	3.32284	1.73242	3.32284
Tmem71	NM_172514	7.77E-05	16.5709	5.89974	4.169	3.31899	1.73074	3.31899
Pkp4	NM_026361	0.000103	15.4249	8.56145	6.83093	3.31847	1.73052	3.31847
Chek2	NM_016681	0.002842	6.5293	7.17075	5.44482	3.30794	1.72593	3.30794
Crim1	NM_015800	0.001757	7.42436	9.94915	8.22393	3.30631	1.72522	3.30631
Cdsn	NM_001008424	0.003668	6.09375	7.88054	6.16071	3.29397	1.71983	3.29397
Gsr	NM_010344	4.63E-06	33.6972	8.9227	7.20366	3.29219	1.71905	3.29219
Cdk6	NM_009873	0.001789	7.38927	8.64593	6.92758	3.29058	1.71834	3.29058
	---	0.001988	7.18505	10.9439	9.22606	3.28945	1.71785	3.28945
Tcirg1	NM_016921	0.000124	14.7337	9.96293	8.24668	3.2858	1.71624	3.2858
Akr1c13	NM_013778	0.000739	9.31492	5.60417	3.88809	3.28543	1.71608	3.28543
Ash2l	NM_011791	7.07E-06	30.2917	10.1317	8.41778	3.28055	1.71394	3.28055
Ipo13	NM_146152	7.78E-05	16.5635	8.88982	7.17972	3.27184	1.7101	3.27184
Slc2a6	NM_172659	0.000554	10.0391	7.75597	6.04587	3.27184	1.7101	3.27184
Pdzd2	NM_001081064	0.000603	9.82091	6.42246	4.71385	3.26845	1.70861	3.26845
Hdac9	NM_024124	0.000431	10.7106	6.76611	5.06131	3.25983	1.7048	3.25983
Tnfrsf12a	NM_013749	0.000543	10.0887	10.0596	8.35956	3.24913	1.70005	3.24913
Spred2	BC040462	0.00014	14.2645	9.05796	7.35801	3.24889	1.69995	3.24889

Ptpre	NM_011212	7.86E-05	16.5241	6.29663	4.59902	3.24362	1.69761	3.24362
	---	5.03E-05	18.4901	10.2864	8.5904	3.23994	1.69597	3.23994
Lmbr1l	NM_029098	0.000445	10.6198	8.48088	6.78769	3.23372	1.6932	3.23372
Tmie	NM_146260	0.000572	9.95681	6.72742	5.037	3.2275	1.69042	3.2275
Tec	NM_001113460	0.003562	6.14258	8.44438	6.75994	3.21415	1.68444	3.21415
LOC100038746	ENSMUST00000084298	7.57E-05	16.6782	8.32528	6.642	3.21157	1.68328	3.21157
Slc1a4	NM_018861	7.94E-05	16.477	10.1393	8.45736	3.2086	1.68195	3.2086
	---	0.001473	7.77876	6.59048	4.90992	3.20552	1.68056	3.20552
4930572J05Rik	NM_198607	8.43E-05	16.2332	7.4881	5.80821	3.20403	1.67989	3.20403
Tmc6	NM_145439	0.000233	12.5359	7.23469	5.55495	3.20369	1.67973	3.20369
Ppm1l	NM_178726	0.002024	7.1508	8.28389	6.60707	3.19723	1.67682	3.19723
Stk17b	NM_133810	0.000172	13.5472	10.8147	9.1447	3.18209	1.66997	3.18209
Nrp2	NM_001077403	0.003872	6.0046	9.05023	7.38072	3.18106	1.66951	3.18106
Mtbp	NM_134092	0.002863	6.51628	8.71594	7.04652	3.18085	1.66941	3.18085
Nlgn1	NM_138666	0.001203	8.20307	6.15252	4.4874	3.1714	1.66512	3.1714
Abcb1a	NM_011076	8.20E-05	16.3443	7.16939	5.50562	3.16843	1.66377	3.16843
	---	6.73E-05	17.1838	8.99121	7.3318	3.15887	1.65941	3.15887
Shc4	NM_199022	8.64E-05	16.1326	6.74109	5.08302	3.15593	1.65807	3.15593
Tarsl2	NM_172310	0.001791	7.38619	6.93618	5.28153	3.14846	1.65465	3.14846
Ngf	NM_013609	0.000343	11.3548	7.3112	5.65803	3.14525	1.65317	3.14525
Chd7	NM_001081417	0.000747	9.28951	7.20766	5.55772	3.13821	1.64994	3.13821
Sh3tc2	NM_172628	0.000959	8.70481	6.53466	4.89045	3.12578	1.64422	3.12578
Aqp5	NM_009701	0.004151	5.89132	8.58286	6.93963	3.12364	1.64323	3.12364
Pdgfa	NM_008808	0.00014	14.26	8.78014	7.13949	3.11805	1.64064	3.11805
9930111J21Rik2	NM_173434	0.000196	13.0933	6.16212	4.52192	3.1171	1.6402	3.1171
Chd7	NM_001081417	0.001057	8.48659	5.16462	3.53328	3.098	1.63134	3.098
Slc30a4	NM_011774	0.000154	13.9418	9.60296	7.97525	3.09021	1.6277	3.09021
Coro2b	NM_175484	0.000599	9.83452	6.45408	4.82871	3.08521	1.62537	3.08521
Hist2h2be	NM_178214	0.000879	8.90415	5.58497	3.96231	3.07943	1.62266	3.07943
Herc6	NM_025992	0.006101	5.29665	7.43708	5.81712	3.07366	1.61996	3.07366

Gm4340	NM_001177535	0.000664	9.57603	5.26892	3.64949	3.07253	1.61943	3.07253
Sc4mol	NM_025436	0.001744	7.43916	10.6341	9.01745	3.0666	1.61664	3.0666
A830019P07Rik	ENSMUST0000006908 4	0.001785	7.39279	5.93131	4.31621	3.06332	1.61509	3.06332
	---	0.000714	9.40032	8.87835	7.26423	3.06124	1.61411	3.06124
5730419I09Rik	NM_029081	0.000509	10.2611	8.72495	7.1184	3.04523	1.60655	3.04523
Sh3bp2	NM_001145859	0.000753	9.27077	7.57836	5.97311	3.04248	1.60525	3.04248
Pitpnm1	NM_008851	0.00019	13.2088	8.31517	6.71049	3.04128	1.60468	3.04128
Fam164c	NM_172414	0.001097	8.40359	5.12887	3.52494	3.03969	1.60392	3.03969
	---	0.001604	7.60532	6.6178	5.01498	3.03736	1.60282	3.03736
	---	0.000735	9.32808	8.21415	6.61365	3.03248	1.6005	3.03248
Carns1	NM_134148	7.31E-05	16.8258	7.08067	5.48196	3.02872	1.59871	3.02872
Itpr3	NM_080553	4.25E-05	19.3013	8.84181	7.24671	3.02116	1.5951	3.02116
Il34	NM_029646	0.006375	5.23197	6.4066	4.8143	3.01529	1.5923	3.01529
Cdkl5	NM_001024624	7.31E-05	16.8279	5.5459	3.95607	3.01014	1.58983	3.01014
2310037I24Rik	BC083325	2.82E-05	21.4	10.7677	9.17843	3.00902	1.58929	3.00902
Cdc14a	NM_001080818	0.001143	8.31485	6.86286	5.27436	3.00737	1.5885	3.00737
Rpap3	NM_028003	5.24E-05	18.3076	8.58768	6.99957	3.00655	1.58811	3.00655
Mrpl38	NM_024177	6.36E-05	17.4294	9.81445	8.22699	3.00518	1.58745	3.00518
Stk10	NM_009288	4.33E-05	19.2094	7.74444	6.15982	2.99929	1.58462	2.99929
Gm10561	ENSMUST0000009773 6	0.001527	7.70509	6.90311	5.3187	2.99885	1.58441	2.99885
Ptpn6	NM_013545	3.09E-05	20.9147	7.11393	5.52956	2.99876	1.58437	2.99876
Gsto2	NM_026619	0.001186	8.23486	7.87071	6.28704	2.99732	1.58367	2.99732
Hspa8	NM_031165	0.00024	12.4437	12.4385	10.8549	2.99723	1.58363	2.99723
Des	NM_010043	0.003528	6.15861	6.38248	4.80334	2.98792	1.57914	2.98792
Sp100	NM_013673	0.003225	6.31039	6.31224	4.7331	2.98792	1.57914	2.98792
Ezr	NM_009510	0.003649	6.10236	8.72486	7.14686	2.98556	1.578	2.98556
	---	0.001644	7.55632	9.44299	7.868	2.97933	1.57499	2.97933
Sp110	NM_175397	0.001741	7.44216	6.04114	4.46673	2.97814	1.57441	2.97814
Lamb2	NM_008483	5.87E-05	17.7844	10.8407	9.26745	2.97584	1.57329	2.97584
Nipal3	NM_028995	0.001304	8.03258	6.14278	4.57083	2.97306	1.57195	2.97306

0610011F06Rik	NM_026686	0.000408	10.8605	8.84418	7.27281	2.97188	1.57137	2.97188
Ptgds	NM_008963	0.001948	7.22377	7.18761	5.61646	2.97141	1.57115	2.97141
Pick1	NM_008837	0.000487	10.374	7.63811	6.07029	2.96455	1.56782	2.96455
2310037I24Rik	BC003301	4.71E-05	18.8078	10.8091	9.24221	2.96271	1.56692	2.96271
Hspb6	NM_001012401	0.001011	8.58511	9.14342	7.57685	2.96199	1.56657	2.96199
A630001G21Rik	NM_177055	0.001777	7.40218	6.27843	4.71273	2.96022	1.5657	2.96022
2200002D01Rik	NM_028179	7.97E-05	16.465	8.05802	6.4925	2.95985	1.56553	2.95985
Gsto1	NM_010362	5.80E-05	17.8397	10.2014	8.63696	2.95758	1.56442	2.95758
Slc29a1	NM_022880	0.000199	13.0426	9.60733	8.04342	2.95654	1.56391	2.95654
Chst1	NM_023850	0.000587	9.88801	7.63507	6.07133	2.95619	1.56374	2.95619
Ifit2	NM_008332	0.002227	6.97073	7.16104	5.5975	2.95579	1.56355	2.95579
Cdkl2	NM_016912	1.94E-05	23.5125	6.26418	4.70078	2.95548	1.56339	2.95548
Ints4	NM_027256	7.40E-05	16.7752	9.59677	8.03404	2.95411	1.56273	2.95411
Vps13a	NM_173028	0.002271	6.93431	7.82032	6.25925	2.95073	1.56107	2.95073
	---	0.00335	6.24571	6.63747	5.07646	2.95062	1.56102	2.95062
Nlrp10	NM_175532	0.004115	5.90536	5.73827	4.18052	2.94394	1.55775	2.94394
Slc12a2	NM_009194	0.002527	6.73851	9.61914	8.06186	2.943	1.55729	2.943
Arhgap19	NM_027667	0.001288	8.05865	5.96147	4.40614	2.93901	1.55533	2.93901
Fgd3	NM_015759	5.31E-05	18.2458	6.06336	4.50914	2.93676	1.55423	2.93676
Gm9958	ENSMUST00000068250	0.001526	7.70651	5.72914	4.17749	2.93152	1.55165	2.93152
Etnk1	NM_029250	0.000545	10.0788	9.68248	8.13183	2.92949	1.55065	2.92949
	---	0.000895	8.86371	10.436	8.88603	2.92816	1.54999	2.92816
Agpat9	NM_172715	0.000113	15.0687	7.50016	5.95112	2.92622	1.54904	2.92622
Fam107b	BC021353	4.67E-05	18.8393	6.58174	5.033	2.92562	1.54874	2.92562
Taf2	NM_001081288	0.002871	6.5118	9.87401	8.32544	2.92526	1.54857	2.92526
Rhox2a	NM_029203	0.003608	6.12093	6.148	4.60102	2.92205	1.54698	2.92205
Pik3cb	NM_029094	8.46E-05	16.2171	8.1153	6.56881	2.92105	1.54649	2.92105
Fads1	NM_146094	0.000458	10.5448	10.042	8.49706	2.91787	1.54492	2.91787
Plcg2	NM_172285	0.000363	11.1939	7.19954	5.65621	2.91466	1.54333	2.91466
Slc23a2	NM_018824	4.26E-05	19.2808	9.6001	8.05681	2.91459	1.54329	2.91459

Epn3	NM_027984	0.000404	10.885	7.15968	5.62052	2.90625	1.53916	2.90625
Chd3	NM_146019	5.32E-05	18.2326	9.73342	8.1951	2.90456	1.53832	2.90456
Snta1	NM_009228	0.001186	8.23482	7.88618	6.35065	2.89895	1.53553	2.89895
Tns1	NM_027884	0.001331	7.98847	10.0202	8.49135	2.88553	1.52884	2.88553
Tfb1m	NM_146074	0.007075	5.08125	7.33302	5.80466	2.88459	1.52837	2.88459
Slc9a3r1	NM_012030	0.000385	11.0218	8.61804	7.08992	2.88409	1.52812	2.88409
Aftph	NM_181411	5.06E-05	18.4677	9.51196	7.98573	2.88031	1.52622	2.88031
Gchfr	NM_177157	0.000889	8.87982	6.66588	5.13995	2.87972	1.52593	2.87972
Net1	NM_019671	0.004109	5.90787	7.92648	6.40136	2.87812	1.52513	2.87812
Zfp664	NM_001081750	0.000137	14.3614	9.48235	7.95814	2.87629	1.52421	2.87629
Myof	NM_001099634	0.000523	10.1877	10.5118	8.98843	2.87459	1.52336	2.87459
P2rx1	NM_008771	0.000118	14.8959	5.26771	3.7475	2.86832	1.52021	2.86832
	---	0.000357	11.2408	7.71572	6.19562	2.86812	1.5201	2.86812
Aplp2	NM_001102455	9.14E-05	15.902	11.9179	10.3979	2.86799	1.52004	2.86799
Sigirr	NM_023059	0.000307	11.6847	6.81465	5.29524	2.86674	1.51941	2.86674
	---	0.005834	5.36326	9.65931	8.13993	2.86669	1.51938	2.86669
Camta1	NM_001081557	0.00175	7.43259	5.11315	3.59452	2.86519	1.51863	2.86519
	---	3.19E-05	20.7491	11.0064	9.4893	2.86221	1.51713	2.86221
Specc1	NM_001029936	0.000629	9.71499	7.61334	6.09667	2.8613	1.51667	2.8613
Eral1	NM_022313	0.000225	12.6423	8.17347	6.65937	2.85621	1.5141	2.85621
	---	0.000208	12.8985	11.1762	9.66465	2.85124	1.51159	2.85124
Slc35e4	NM_153142	0.000385	11.0231	7.36315	5.85195	2.85046	1.5112	2.85046
Gxylt2	NM_198612	0.000431	10.7072	8.2722	6.7613	2.84989	1.5109	2.84989
Tctn3	NM_026260	0.002458	6.78913	7.77282	6.262	2.84972	1.51082	2.84972
Gdpd5	NM_201352	0.000444	10.627	7.27628	5.76596	2.84875	1.51033	2.84875
Chd7	NM_001081417	0.000428	10.7304	6.20962	4.70395	2.83957	1.50567	2.83957
Cux1	NM_009986	0.000721	9.37658	7.88676	6.38514	2.83161	1.50162	2.83161
Plcl1	NM_001114663	0.000296	11.7873	7.9745	6.47562	2.82623	1.49888	2.82623
	---	0.000134	14.4227	9.61277	8.11415	2.82572	1.49862	2.82572
	---	9.98E-05	15.5532	5.39408	3.89549	2.82566	1.49859	2.82566
Strbp	NM_009261	0.000292	11.8361	7.46304	5.96685	2.82096	1.49619	2.82096

Cacna1h	NM_021415	0.00078	9.18719	6.38409	4.8882	2.82039	1.4959	2.82039
	---	0.000241	12.4231	9.29797	7.80284	2.8189	1.49513	2.8189
Pcdh1	NM_029357	0.000178	13.4294	5.44766	3.9537	2.81661	1.49396	2.81661
Sord	NM_146126	0.006352	5.23726	10.6583	9.16453	2.81629	1.49379	2.81629
Shisa4	NM_175259	0.000713	9.40271	9.16471	7.67127	2.8156	1.49344	2.8156
Acot7	NM_133348	0.000698	9.45402	9.76622	8.2782	2.80505	1.48802	2.80505
Abcb6	NM_023732	0.000112	15.1153	8.35965	6.8735	2.80141	1.48615	2.80141
Zfp422	NM_026057	0.000204	12.9622	8.53312	7.04805	2.79931	1.48507	2.79931
Usp53	NM_133857	0.001128	8.34294	8.71739	7.23319	2.79763	1.48421	2.79763
	---	0.000398	10.9314	10.3898	8.90613	2.79658	1.48366	2.79658
Cd48	NM_007649	0.003024	6.42077	5.08696	3.60378	2.79563	1.48318	2.79563
Slc25a24	NM_172685	7.28E-05	16.8464	9.81996	8.34075	2.78797	1.47922	2.78797
	---	0.000134	14.419	10.1569	8.67893	2.78558	1.47798	2.78558
Rad51c	NM_053269	0.001455	7.80386	6.30861	4.83158	2.78375	1.47703	2.78375
Macrod2	NM_001013802	0.002454	6.79179	6.41612	4.94061	2.78082	1.47551	2.78082
Macrod2	NM_001013802	0.001389	7.89966	5.1162	3.6457	2.77119	1.47051	2.77119
Ppip5k1	NM_178795	1.44E-05	25.3393	7.40292	5.93441	2.76735	1.46851	2.76735
Pcdh9	NM_001081377	0.006619	5.17717	7.64325	6.17564	2.76564	1.46761	2.76564
	---	0.000175	13.4822	10.8789	9.41138	2.76548	1.46753	2.76548
Fam33a	NM_025377	0.006578	5.1863	8.67618	7.20871	2.76536	1.46747	2.76536
Adipor2	NM_197985	0.002524	6.74079	8.94078	7.47554	2.76109	1.46524	2.76109
Heatr2	NM_001081265	1.18E-05	26.6363	7.48832	6.02309	2.76108	1.46523	2.76108
Idh2	NM_173011	0.000279	11.9769	10.0964	8.63303	2.75756	1.46339	2.75756
Myo18a	NM_011586	0.00017	13.5941	7.7893	6.32743	2.75466	1.46187	2.75466
Synj2	NM_011523	3.89E-05	19.7308	8.63337	7.17216	2.75339	1.46121	2.75339
Map4k2	NM_009006	0.000428	10.7278	7.55722	6.09886	2.74797	1.45837	2.74797
	---	0.004771	5.67058	6.19466	4.7378	2.7451	1.45686	2.7451
Pcgf5	NM_029508	0.001943	7.22871	8.26675	6.81401	2.73728	1.45274	2.73728
Prss46	NM_183103	5.62E-05	17.9855	5.84643	4.39388	2.73691	1.45255	2.73691
Rftn2	NM_028713	3.58E-05	20.1471	7.99326	6.54371	2.73123	1.44955	2.73123
3110048L19Rik	NR_003549	0.003138	6.35693	7.91812	6.47144	2.7258	1.44668	2.7258

Edn1	NM_010104	0.00103	8.54345	6.17595	4.73006	2.72431	1.44589	2.72431
Dyrk3	NM_145508	0.00376	6.05265	7.12663	5.6818	2.7223	1.44483	2.7223
Sft2d2	NM_145512	0.000174	13.4955	8.73964	7.29655	2.71902	1.44309	2.71902
Sord	NM_146126	0.005851	5.35872	10.4704	9.02744	2.71876	1.44295	2.71876
Rcor2	NM_054048	0.000867	8.93657	7.28012	5.83807	2.71707	1.44205	2.71707
Vamp5	NM_016872	3.32E-05	20.5372	7.63702	6.19546	2.71613	1.44155	2.71613
	---	7.37E-05	16.7943	11.2083	9.76717	2.71539	1.44116	2.71539
Nfs1	NM_010911	0.000379	11.07	9.24685	7.80824	2.71059	1.43861	2.71059
Reep6	NM_139292	0.000691	9.47878	7.62346	6.18525	2.70983	1.4382	2.70983
Mrpl19	NM_026490	0.000233	12.5321	8.33239	6.89528	2.70777	1.43711	2.70777
Odz3	NM_011857	0.000263	12.1564	9.17189	7.73707	2.70347	1.43481	2.70347
Zfp157	NM_028130	0.004224	5.86318	8.02595	6.59137	2.70305	1.43459	2.70305
0610010O12Rik	BC028765	0.000502	10.2937	8.05893	6.62471	2.70237	1.43423	2.70237
Rgs7	NM_011880	0.00339	6.22534	4.61196	3.17811	2.70167	1.43385	2.70167
Gpr123	NM_177469	5.38E-05	18.1867	6.65523	5.22164	2.70117	1.43358	2.70117
Lcor	NM_172154	0.000201	13.009	8.58073	7.14741	2.70069	1.43333	2.70069
Rpa1	NM_001164223	0.001986	7.18698	8.62726	7.19744	2.69414	1.42983	2.69414
Scn3a	NM_018732	8.87E-05	16.0249	4.89202	3.46239	2.69377	1.42963	2.69377
Tlcd1	NM_026708	0.001083	8.43301	8.59501	7.16565	2.69327	1.42936	2.69327
Tnfrsf9	NM_011612	0.002934	6.47366	7.77629	6.34694	2.69325	1.42935	2.69325
Otud7a	NM_130880	0.000577	9.93382	5.92714	4.49941	2.69023	1.42773	2.69023
2310030G06Rik	BC027409	0.000759	9.25156	6.40078	4.97357	2.68926	1.42721	2.68926
	---	0.00424	5.85709	10.6165	9.19265	2.68301	1.42385	2.68301
Ehd4	NM_133838	0.004748	5.67815	8.15421	6.73055	2.68265	1.42366	2.68265
Hadh	NM_008212	0.000319	11.5642	11.0209	9.59775	2.68178	1.42319	2.68178
Hip1	NM_146001	0.000634	9.69307	7.86477	6.44187	2.68125	1.4229	2.68125
Nmt1	NM_008707	2.22E-05	22.7394	10.4795	9.05744	2.67977	1.42211	2.67977
Gdnf	NM_010275	0.001941	7.23039	8.3514	6.92941	2.67954	1.42199	2.67954
Glo1	NM_025374	5.06E-05	18.4666	10.102	8.68009	2.67937	1.42189	2.67937
Fahd1	NM_023480	0.001819	7.35577	6.84934	5.42831	2.67777	1.42103	2.67777
Ston2	NM_175367	0.003488	6.17756	6.83891	5.41903	2.67564	1.41988	2.67564

Cd300lb	NM_199221	0.001723	7.46325	6.66213	5.24391	2.67254	1.41821	2.67254
Ddr1	NM_007584	2.87E-05	21.3011	8.19401	6.77624	2.67173	1.41777	2.67173
Rhpn2	NM_027897	0.000691	9.47764	6.34121	4.92419	2.67034	1.41702	2.67034
Axl	NM_009465	2.36E-05	22.381	10.7282	9.31137	2.67006	1.41687	2.67006
Parvb	NM_133167	1.10E-05	27.0934	7.01545	5.59873	2.66978	1.41672	2.66978
Adssl1	NM_007421	0.004177	5.8814	7.84413	6.42874	2.66732	1.41539	2.66732
Plekhg4	NM_001081333	2.84E-05	21.3561	6.29555	4.8806	2.6665	1.41495	2.6665
Lrp11	NM_172784	0.000826	9.05118	8.50022	7.08875	2.66008	1.41147	2.66008
C1qtnf1	NM_019959	0.004062	5.9263	7.57183	6.16049	2.65985	1.41135	2.65985
Trim16	NM_053169	0.000674	9.53995	8.10094	6.69132	2.65667	1.40962	2.65667
	---	0.00221	6.98478	5.48576	4.07974	2.65004	1.40602	2.65004
Btbd9	NM_027060	1.21E-05	26.4516	8.4448	7.03915	2.64937	1.40565	2.64937
	---	0.000453	10.5717	10.9573	9.55178	2.64906	1.40548	2.64906
	---	0.002716	6.60928	8.98497	7.5797	2.64866	1.40526	2.64866
	---	8.38E-05	16.2543	11.3219	9.91706	2.64786	1.40483	2.64786
Ccnt1	NM_009833	0.000326	11.5003	9.26112	7.8573	2.64602	1.40382	2.64602
Chd7	NM_001081417	0.000409	10.8542	5.62206	4.21974	2.64326	1.40232	2.64326
Wdyhv1	NM_029734	0.000778	9.19361	8.95959	7.55769	2.6425	1.4019	2.6425
Dyrk4	NM_207210	0.002291	6.91833	5.76676	4.36566	2.64102	1.40109	2.64102
Hexim2	NM_027658	0.000545	10.0811	7.11466	5.71572	2.63708	1.39894	2.63708
Tmem62	NM_175285	0.001829	7.34574	7.41643	6.01893	2.63444	1.3975	2.63444
	---	0.00302	6.42349	8.15739	6.76125	2.63196	1.39614	2.63196
Cyfip2	NM_133769	0.000841	9.00816	6.6299	5.23379	2.6319	1.3961	2.6319
Bbs12	NM_001008502	0.003846	6.01554	6.17124	4.77591	2.63048	1.39533	2.63048
Plaur	NM_011113	0.001704	7.48456	8.75644	7.36147	2.62982	1.39496	2.62982
Stard13	NM_001163493	0.003453	6.1946	8.31442	6.91953	2.62968	1.39489	2.62968
Morc2a	NM_001159288	0.001076	8.44771	10.1125	8.71789	2.62914	1.39459	2.62914
Fam164a	NM_173181	0.001283	8.06563	9.39364	7.99975	2.62786	1.39389	2.62786
Glo1	NM_025374	5.04E-05	18.4813	10.1334	8.74217	2.62301	1.39122	2.62301
Ctsw	NM_009985	0.001256	8.11122	6.24522	4.85811	2.61555	1.38711	2.61555
	---	0.00596	5.33131	8.63363	7.24714	2.61443	1.38649	2.61443

Dagla	NM_198114	5.31E-06	32.5568	7.6151	6.2287	2.61427	1.38641	2.61427
Chd7	NM_001081417	0.000509	10.2584	5.0961	3.70977	2.61413	1.38633	2.61413
Msto1	NM_144898	0.000143	14.1887	8.24496	6.8605	2.61073	1.38445	2.61073
	---	2.31E-05	22.5007	11.7887	10.4042	2.61068	1.38443	2.61068
	---	0.005092	5.56955	4.56609	3.18303	2.60822	1.38307	2.60822
Macrod2	NM_028387	0.0007	9.44839	6.29665	4.91537	2.60499	1.38128	2.60499
Irf1	NM_008390	0.003954	5.97015	9.14553	7.7658	2.60219	1.37973	2.60219
Tra2a	AB052758	0.00155	7.67389	7.4618	6.08293	2.60064	1.37887	2.60064
Tle4	NM_011600	0.000921	8.79602	6.76819	5.39241	2.59508	1.37578	2.59508
Dhcr7	NM_007856	0.002571	6.70801	7.49529	6.11967	2.59479	1.37562	2.59479
Tmem171	NM_001025606	7.01E-05	17.0077	6.54714	5.17365	2.59097	1.37349	2.59097
Smarcd1	NM_031842	0.003273	6.28517	7.67512	6.30276	2.58894	1.37236	2.58894
Gata3	NM_008091	0.00095	8.72527	6.10841	4.73728	2.58673	1.37113	2.58673
Cpeb3	NM_198300	0.000665	9.57531	5.66125	4.29058	2.5859	1.37067	2.5859
Ces2g	NM_197999	0.002465	6.78412	7.17448	5.80634	2.58136	1.36813	2.58136
Spred2	NM_033523	0.000226	12.6341	8.53347	7.16591	2.58033	1.36756	2.58033
Ccnt2	NM_028399	0.001117	8.36454	9.42001	8.05264	2.57999	1.36737	2.57999
Chd7	NM_001081417	0.001835	7.33881	4.30329	2.93613	2.57963	1.36716	2.57963
Ankrd13c	NM_001013806	0.000436	10.6776	9.24749	7.88113	2.57818	1.36636	2.57818
Polr3h	NM_030229	0.000421	10.7728	7.27279	5.90805	2.57529	1.36473	2.57529
Tpra1	NM_011906	0.000154	13.9295	9.43662	8.07408	2.57138	1.36254	2.57138
Lrrc8b	NM_001033550	0.001535	7.69418	7.02696	5.66561	2.56926	1.36135	2.56926
Pnpla2	NR_028142	0.003622	6.11453	7.98432	6.62324	2.56878	1.36108	2.56878
D4Bwg0951e	BC030404	0.005542	5.44016	7.90317	6.54218	2.56861	1.36099	2.56861
Atp2a3	NM_016745	0.00019	13.1964	6.45744	5.09864	2.56472	1.3588	2.56472
Gm340	AK145489	0.003599	6.12522	6.92693	5.56822	2.56456	1.35871	2.56456
Tfrc	NM_011638	0.006479	5.20825	9.67404	8.31699	2.56162	1.35706	2.56162
Tbc1d4	NM_001081278	0.000118	14.8966	6.37811	5.0211	2.56154	1.35701	2.56154
Tes	NM_207176	0.002171	7.01776	10.0583	8.70348	2.55762	1.3548	2.55762
Tjap1	NM_028751	5.42E-05	18.1454	7.71092	6.35641	2.55711	1.35451	2.55711
Chd7	NM_001081417	0.001422	7.85088	6.80194	5.44795	2.55617	1.35398	2.55617

Pde8a	NM_008803	0.000328	11.485	8.32749	6.97389	2.5555	1.35361	2.5555
Parp10	NM_001163575	6.98E-05	17.0267	8.03387	6.68563	2.54602	1.34825	2.54602
Rnd1	NM_172612	0.005774	5.37853	7.17855	5.83051	2.54565	1.34804	2.54565
Hbegf	NM_010415	0.004385	5.80364	8.70168	7.35523	2.54285	1.34645	2.54285
Rasal2	NM_177644	4.52E-05	19.0021	6.82556	5.4792	2.54269	1.34636	2.54269
Syne2	NM_001005510	0.000885	8.8897	6.50179	5.15582	2.54201	1.34597	2.54201
Zfp93	NM_009567	0.002724	6.60439	7.10234	5.75793	2.53926	1.34441	2.53926
Gpr141	NM_181754	0.000889	8.87909	4.27473	2.93045	2.53904	1.34429	2.53904
Tmcc3	NM_172051	0.000182	13.3523	6.53027	5.18754	2.53632	1.34274	2.53632
Fam126a	NM_053090	0.000584	9.89955	8.89259	7.55	2.53606	1.34259	2.53606
Fryl	NM_028194	0.002042	7.13353	7.98085	6.63967	2.53358	1.34118	2.53358
Wwc2	NM_133791	8.60E-05	16.1473	8.92326	7.58286	2.53223	1.34041	2.53223
Hdgfrp3	NM_013886	0.001346	7.96565	6.81042	5.47005	2.53216	1.34037	2.53216
Cntnap4	NM_130457	0.004553	5.74396	6.11857	4.77824	2.5321	1.34034	2.5321
Fam117b	NM_001037725	0.00215	7.03656	7.98977	6.65053	2.53019	1.33924	2.53019
Trmt12	NM_026642	0.004895	5.63033	7.06402	5.72535	2.52918	1.33867	2.52918
Fam83h	NM_134087	0.000775	9.20269	6.63346	5.29629	2.52654	1.33716	2.52654
Galnt7	NM_144731	0.000204	12.9717	8.23489	6.9008	2.52117	1.33409	2.52117
Fggy	NM_001113412	0.003857	6.01092	7.06921	5.73608	2.51949	1.33313	2.51949
Slc5a8	NM_145423	0.000531	10.1458	5.90224	4.57072	2.51667	1.33152	2.51667
Clstn1	NM_023051	0.000183	13.3314	9.65759	8.32673	2.51554	1.33087	2.51554
Papolg	NM_172555	0.004139	5.89595	8.10977	6.78074	2.51235	1.32904	2.51235
Unc119	NM_011676	0.001476	7.77377	8.6326	7.30434	2.511	1.32826	2.511
	---	0.005783	5.37616	8.65222	7.3247	2.5097	1.32752	2.5097
Ctnnal1	NM_018761	0.001621	7.5843	8.22187	6.89654	2.5059	1.32533	2.5059
Pik3r3	NM_181585	0.003613	6.11856	7.91467	6.59106	2.50292	1.32362	2.50292
Gmpr	NM_025508	0.000804	9.11279	6.79989	5.47786	2.50018	1.32203	2.50018
Ddx58	NM_172689	5.35E-05	18.2089	9.09901	7.77723	2.49974	1.32178	2.49974
	---	0.005104	5.56594	8.27143	6.94978	2.49951	1.32164	2.49951
Fut8	NM_016893	0.000474	10.4485	8.18128	6.86085	2.49741	1.32043	2.49741
Pigs	NM_201406	7.16E-05	16.9155	9.10149	7.78209	2.49562	1.3194	2.49562

Krtap1-5	NM_027157	0.002291	6.91809	4.53353	3.21463	2.49476	1.3189	2.49476
Ppp1r13b	NM_011625	0.000112	15.0947	6.77982	5.46121	2.49427	1.31862	2.49427
Kbtbd8	NM_001008785	0.000236	12.4979	5.17247	3.85393	2.49414	1.31854	2.49414
Tgtp1	NM_011579	0.003764	6.0511	5.79245	4.47426	2.49353	1.31819	2.49353
Eme2	NM_001163102	0.000636	9.68455	8.31961	7.00472	2.48784	1.31489	2.48784
Marveld1	NM_183195	3.32E-05	20.5436	10.2018	8.88834	2.48542	1.31349	2.48542
Tle1	NM_011599	0.001946	7.22526	7.4122	6.09913	2.48468	1.31306	2.48468
Pus10	NM_028304	0.000512	10.2411	8.84806	7.53521	2.48431	1.31285	2.48431
Capn5	NM_007602	0.000451	10.5839	8.13731	6.82531	2.48284	1.31199	2.48284
Rad21	NM_009009	3.20E-05	20.722	10.8478	9.53605	2.4825	1.31179	2.4825
Dtna	NM_207650	0.00323	6.30767	7.14446	5.83477	2.47888	1.30969	2.47888
Nap1l3	NM_138742	0.00069	9.48394	5.35501	4.04533	2.47886	1.30968	2.47886
P2rx7	NM_011027	0.002732	6.59905	7.65232	6.34292	2.47837	1.30939	2.47837
Tmem20	NM_175507	0.000795	9.14053	7.6378	6.32939	2.47669	1.30841	2.47669
Slc4a8	NM_021530	0.000139	14.2986	6.94179	5.63431	2.47509	1.30748	2.47509
	---	0.000621	9.74437	8.30436	6.99816	2.47291	1.30621	2.47291
Gm13154	NM_001014397	0.003481	6.18116	4.11743	2.81137	2.47266	1.30606	2.47266
Madd	NM_001177721	0.001136	8.32697	6.28049	4.97492	2.47181	1.30557	2.47181
Nup155	NM_133227	0.005675	5.40445	8.96526	7.66018	2.47097	1.30508	2.47097
Aox1	NM_009676	9.03E-05	15.9496	9.54353	8.24172	2.46539	1.30181	2.46539
Gm10351	AB010332	0.000237	12.4851	5.88152	4.58135	2.46259	1.30018	2.46259
Slc12a5	NM_020333	9.87E-06	27.8624	6.42985	5.13069	2.46086	1.29916	2.46086
	---	0.003899	5.9932	6.00686	4.70773	2.4608	1.29913	2.4608
Ccdc88c	NM_026681	0.000992	8.62782	6.33919	5.04008	2.46077	1.29911	2.46077
Plekhg1	NM_001033253	0.001128	8.34356	5.34076	4.04246	2.45939	1.2983	2.45939
Snx16	NM_029068	4.20E-05	19.3538	7.79923	6.50131	2.45874	1.29792	2.45874
Rasa4	NM_133914	0.002389	6.84082	7.2585	5.96235	2.45574	1.29616	2.45574
Ccdc92	NM_144819	0.002108	7.07372	7.25266	5.95714	2.45466	1.29552	2.45466
Fryl	NM_028194	0.002357	6.86603	7.54553	6.25182	2.45158	1.29371	2.45158
Mcm8	NM_025676	0.004181	5.87967	6.58236	5.28939	2.45032	1.29297	2.45032
Rab27b	NM_001082553	0.001685	7.50676	6.25397	4.96166	2.4492	1.29231	2.4492

Fryl	NM_028194	0.000554	10.0393	8.62333	7.3313	2.44871	1.29202	2.44871
Tmem37	NM_019432	0.004244	5.85577	6.34861	5.06199	2.43955	1.28662	2.43955
Fggy	NM_001113412	0.006519	5.19944	6.47516	5.18886	2.43901	1.2863	2.43901
Insl6	NM_013754	0.006423	5.22091	6.09033	4.8053	2.43687	1.28503	2.43687
Tgtp1	NM_011579	0.005201	5.5368	5.39262	4.10761	2.43685	1.28502	2.43685
Tbc1d1	NM_019636	0.002771	6.57407	8.1556	6.87087	2.43636	1.28473	2.43636
Pde7a	NM_001122759	0.004804	5.65977	8.486	7.20403	2.4317	1.28197	2.4317
Gltpd1	NM_024472	0.000185	13.2866	7.54383	6.26269	2.43031	1.28114	2.43031
Gm13051	NM_001037926	0.00148	7.76822	8.90374	7.62354	2.42872	1.28019	2.42872
Cdc25b	NM_023117	0.000992	8.62935	7.31622	6.03656	2.42783	1.27967	2.42783
Capn11	NM_001013767	0.001289	8.0557	5.34794	4.0696	2.4256	1.27834	2.4256
Rbm38	NM_019547	0.000354	11.2643	7.59414	6.31614	2.42502	1.278	2.42502
Rasa3	NM_009025	0.005958	5.3318	9.44138	8.16502	2.42228	1.27637	2.42228
Lipo1	NM_001013770	0.000494	10.3392	7.68673	6.41133	2.42064	1.27539	2.42064
	---	0.001478	7.77179	7.17152	5.8964	2.4202	1.27513	2.4202
Rpp30	NM_019428	0.000368	11.1526	8.65661	7.38165	2.41992	1.27496	2.41992
Dock3	NM_153413	3.94E-05	19.6686	5.30534	4.03068	2.41942	1.27466	2.41942
BC034902	BC034902	0.000921	8.79618	5.55423	4.28049	2.41787	1.27374	2.41787
Gm962	BC147482	0.004048	5.93197	6.60319	5.32982	2.41726	1.27337	2.41726
Flrt3	NM_001172160	0.001918	7.2531	6.2212	4.9479	2.41714	1.2733	2.41714
Tspan14	NM_145928	4.21E-05	19.3451	8.64513	7.37207	2.41674	1.27306	2.41674
Slc12a7	NM_011390	0.000183	13.3243	7.21153	5.9389	2.41601	1.27263	2.41601
Ppp3ca	NM_008913	0.000148	14.0772	10.5869	9.31485	2.41503	1.27204	2.41503
Ormdl2	NM_024180	0.00288	6.506	7.3592	6.09079	2.40895	1.2684	2.40895
Prkar2b	NM_011158	0.005791	5.37424	8.62361	7.3563	2.40713	1.26731	2.40713
Prkaa2	NM_178143	0.003129	6.36216	6.02536	4.75975	2.40429	1.26561	2.40429
Gm2049	ENSMUST0000010897<br>8	0.001533	7.6971	5.19292	3.92893	2.4016	1.264	2.4016
Gm2049	ENSMUST0000010897<br>8	0.001533	7.6971	5.19292	3.92893	2.4016	1.264	2.4016
Ccdc43	NM_025918	0.001198	8.21357	7.306	6.04275	2.40036	1.26325	2.40036
	---	0.001758	7.42363	6.4054	5.14268	2.39947	1.26271	2.39947

Gramd4	NM_172611	0.001092	8.41467	8.84931	7.58767	2.39768	1.26164	2.39768
Mir181b-1	NR_029820	0.001516	7.71903	5.83574	4.5756	2.39518	1.26014	2.39518
Erbb3	NM_010153	0.003104	6.37601	6.15679	4.90042	2.38894	1.25637	2.38894
Mrpl45	NM_025927	0.001208	8.19512	8.67709	7.42109	2.38832	1.256	2.38832
Rnpepl1	NM_181405	0.000528	10.1623	7.32985	6.07478	2.38679	1.25507	2.38679
Spred3	NM_182927	2.66E-05	21.7105	7.23367	5.97891	2.38628	1.25476	2.38628
Ptpn4	NM_019933	0.00282	6.54298	6.68865	5.43394	2.3862	1.25471	2.3862
Pik3ip1	NM_178149	0.002253	6.94871	8.66084	7.40743	2.38404	1.25341	2.38404
Ces2d-ps	NR_033726	0.001039	8.52438	5.966	4.71373	2.38215	1.25227	2.38215
Gm14430	NM_001100415	0.002521	6.74323	9.31752	8.06576	2.38133	1.25176	2.38133
1E+08	NM_001099327	0.002521	6.74323	9.31752	8.06576	2.38133	1.25176	2.38133
0610010B08Rik	ENSMUST00000108991	0.002521	6.74323	9.31752	8.06576	2.38133	1.25176	2.38133
1E+08	NM_001099327	0.002521	6.74323	9.31752	8.06576	2.38133	1.25176	2.38133
Vmn1r43	NM_053220	0.001301	8.03721	4.37405	3.12496	2.37692	1.24909	2.37692
Cdyl2	NM_029441	0.001987	7.18556	6.91667	5.67029	2.37245	1.24638	2.37245
	---	0.002453	6.79253	9.32121	8.07712	2.3687	1.24409	2.3687
Map3k4	NM_011948	0.000591	9.87063	7.86004	6.61604	2.36854	1.244	2.36854
Lpcat4	NM_207206	6.61E-05	17.2601	7.63776	6.39397	2.36819	1.24379	2.36819
Shroom3	NM_015756	0.006496	5.20461	6.05755	4.81388	2.36799	1.24366	2.36799
Chchd3	NM_025336	0.000134	14.425	9.50982	8.26631	2.36774	1.24351	2.36774
	---	0.003364	6.23837	7.79645	6.55368	2.36652	1.24277	2.36652
Syne2	NM_001005510	0.001809	7.36736	6.67353	5.43181	2.36482	1.24173	2.36482
Ddx52	NM_030096	0.003555	6.14555	9.02387	7.7829	2.36358	1.24097	2.36358
Zfp110	NM_022981	0.000148	14.0735	8.77939	7.53925	2.36222	1.24014	2.36222
Grina	NM_023168	0.000615	9.7717	11.2113	9.97213	2.36066	1.23919	2.36066
1700010I14Rik	NM_025851	0.000132	14.4835	5.43487	4.19652	2.35928	1.23835	2.35928
Gjb3	NM_008126	0.003696	6.08098	6.22624	4.98997	2.35589	1.23627	2.35589
Calcoco1	NM_026192	0.001929	7.24243	9.95573	8.71958	2.3557	1.23615	2.3557
	---	0.005955	5.33261	6.00345	4.76786	2.35476	1.23558	2.35476
Vegfc	NM_009506	0.002692	6.62563	8.09335	6.85866	2.35332	1.2347	2.35332

Sik1	NM_010831	0.002596	6.69052	9.11572	7.8828	2.35042	1.23292	2.35042
Cyb5b	NM_025558	8.96E-05	15.9828	9.93692	8.70461	2.34943	1.23231	2.34943
	---	0.001516	7.71955	8.88093	7.64873	2.34925	1.2322	2.34925
Zfp111	NM_019940	0.001371	7.92684	6.77573	5.54429	2.34801	1.23144	2.34801
Fbxl16	NM_001164225	0.000743	9.30185	6.44165	5.21095	2.3468	1.2307	2.3468
Lrp8	NR_033496	0.00686	5.12556	6.80444	5.57441	2.34573	1.23004	2.34573
Suz12	NM_199196	0.002808	6.55034	9.06352	7.83411	2.3447	1.22941	2.3447
Tead2	NM_011565	0.00045	10.5897	8.63629	7.4071	2.34435	1.22919	2.34435
Gm10009	ENSMUST0000007105<br>9	0.000169	13.6014	5.34958	4.12068	2.34388	1.2289	2.34388
Zbtb10	NM_177660	0.002549	6.72313	7.91508	6.68786	2.34116	1.22722	2.34116
Scly	NM_016717	0.003688	6.0846	7.17168	5.9445	2.34108	1.22718	2.34108
	---	0.001374	7.92228	9.59968	8.37357	2.33935	1.22611	2.33935
Gm10752	ENSMUST0000009926<br>8	0.001373	7.92387	4.83651	3.61046	2.33925	1.22605	2.33925
Plekhg5	NM_001004156	0.002211	6.98363	7.38612	6.1616	2.33677	1.22452	2.33677
Tshz2	NM_080455	0.004646	5.71208	5.91967	4.69619	2.33509	1.22348	2.33509
Dcakd	NM_026551	3.57E-06	35.956	8.67118	7.44805	2.33453	1.22313	2.33453
Alcam	NM_009655	0.006279	5.25419	7.76321	6.54022	2.3343	1.22299	2.3343
Rhov	NM_145530	0.00245	6.79458	6.51712	5.29416	2.33425	1.22296	2.33425
Phka2	NM_172783	0.005024	5.59011	7.35728	6.13654	2.33067	1.22075	2.33067
	---	0.001752	7.43008	9.62496	8.40459	2.33006	1.22037	2.33006
Slc37a2	NM_001145960	0.006755	5.14778	6.63656	5.41761	2.32777	1.21895	2.32777
Dlx2	NM_010054	0.000742	9.30577	7.29508	6.07918	2.32287	1.21591	2.32287
	---	0.000208	12.9101	6.49806	5.28281	2.32182	1.21526	2.32182
Abtb2	NM_178890	0.000313	11.6274	7.60681	6.39214	2.32088	1.21467	2.32088
1110002L01Rik	NR_030694	0.002933	6.47446	7.34933	6.13708	2.31698	1.21224	2.31698
Tmem106c	NM_201359	0.002417	6.81955	7.77134	6.55972	2.31597	1.21162	2.31597
C030034I22Rik	NR_026848	0.000199	13.0487	5.79573	4.58412	2.31595	1.2116	2.31595
L3mbtl2	NM_145993	0.00098	8.65483	7.67064	6.45954	2.31515	1.2111	2.31515
Myst2	NM_001195003	9.70E-06	27.9858	9.77149	8.56046	2.31504	1.21104	2.31504
	---	0.002556	6.71834	5.71307	4.50244	2.31439	1.21063	2.31439

2610318N02Rik	BC039993	0.003111	6.37204	5.44383	4.23323	2.31434	1.2106	2.31434
Gpr56	NM_018882	8.67E-05	16.1155	6.51082	5.30088	2.31329	1.20995	2.31329
Erbb2	NM_001003817	2.10E-05	23.0464	7.85235	6.64354	2.31146	1.20881	2.31146
Tmem63b	NM_198167	0.000364	11.1829	8.05435	6.84646	2.30999	1.20788	2.30999
Abr	NM_198895	0.002686	6.62956	9.32049	8.11278	2.30971	1.20771	2.30971
Cpeb4	NM_026252	0.001663	7.53282	9.02988	7.82235	2.30941	1.20752	2.30941
	---	0.000635	9.68856	5.82692	4.61955	2.30918	1.20738	2.30918
Actl6a	NM_019673	0.000245	12.3725	8.5106	7.30353	2.30867	1.20706	2.30867
Pcx	NM_001162946	0.000346	11.3283	7.90305	6.69711	2.30688	1.20594	2.30688
A530032D15Rik	BC094285	0.00183	7.34438	6.30246	5.0969	2.30628	1.20556	2.30628
Lamb3	NM_008484	0.001168	8.26793	6.41213	5.20669	2.30608	1.20544	2.30608
Ermp1	NM_001081213	0.000345	11.3373	9.25042	8.04541	2.3054	1.20501	2.3054
Zhx2	NM_199449	0.000673	9.5461	8.11662	6.91199	2.3048	1.20464	2.3048
Pknox2	NM_001029838	0.000337	11.4077	5.31932	4.11888	2.2981	1.20044	2.2981
Sgca	NM_009161	0.000168	13.6358	6.04142	4.84133	2.29754	1.20009	2.29754
	---	0.001627	7.57634	9.29678	8.09745	2.29634	1.19934	2.29634
Vamp1	NM_001080557	0.000767	9.2262	6.9016	5.70238	2.29615	1.19922	2.29615
Plcd1	NM_019676	0.00013	14.5432	8.66571	7.46661	2.29597	1.1991	2.29597
Slc43a2	NM_173388	0.002821	6.54265	6.87639	5.67914	2.29303	1.19726	2.29303
Slc6a12	NM_133661	0.001294	8.04842	6.4445	5.24753	2.29257	1.19697	2.29257
	---	0.001324	7.99919	5.04074	3.84506	2.29053	1.19568	2.29053
Tmem199	NM_199199	0.000551	10.0509	8.10013	6.9048	2.28998	1.19533	2.28998
Grik5	NM_008168	0.000177	13.4536	7.29866	6.1039	2.28907	1.19476	2.28907
Endod1	NM_028013	3.16E-05	20.7906	7.4063	6.21217	2.28806	1.19413	2.28806
Smc4	NM_133786	0.00698	5.10061	8.74245	7.54905	2.28691	1.1934	2.28691
Tns1	NM_027884	0.002254	6.94843	9.21094	8.01771	2.28664	1.19323	2.28664
Polr2k	NM_001039368	0.000421	10.7744	8.5685	7.37565	2.28604	1.19285	2.28604
Nfkbil2	NM_183091	0.000493	10.3434	5.89056	4.69794	2.28568	1.19262	2.28568
Utrn	NM_011682	0.00259	6.6944	8.93763	7.74517	2.28542	1.19246	2.28542
Pfkm	NM_001163487	3.69E-05	20.0042	10.1691	8.97666	2.28539	1.19244	2.28539
Pigq	NM_011822	8.67E-05	16.1178	8.5895	7.3975	2.28469	1.192	2.28469

Tpd52	NM_001025261	0.001959	7.21296	5.25036	4.05883	2.28395	1.19153	2.28395
2410066E13Rik	BC042507	0.003611	6.11953	7.31626	6.12512	2.28333	1.19114	2.28333
	---	0.003005	6.43222	9.40387	8.21406	2.28123	1.18981	2.28123
2810001A02Rik	AK012625	0.001148	8.30512	8.35041	7.16136	2.28003	1.18905	2.28003
Mif4gd	NR_029442	0.000932	8.77056	7.90173	6.71317	2.27925	1.18856	2.27925
Tmem65	NM_175212	0.002058	7.11887	8.75884	7.57087	2.27832	1.18797	2.27832
Morc3	NM_001045529	0.003794	6.03788	8.9605	7.77278	2.27794	1.18773	2.27794
Prepl	NM_001163622	6.53E-05	17.3119	9.41138	8.22372	2.27783	1.18766	2.27783
	---	0.000137	14.3377	10.3485	9.1619	2.27623	1.18665	2.27623
Dennd1b	NM_001166501	0.001668	7.52663	7.3488	6.16257	2.27558	1.18624	2.27558
Ttll12	NM_183017	0.000236	12.4972	8.90159	7.71595	2.27464	1.18564	2.27464
Acsl3	NM_028817	0.000701	9.44396	8.40877	7.22322	2.27449	1.18554	2.27449
Cxcl10	NM_021274	0.005547	5.43889	6.60037	5.41707	2.27095	1.1833	2.27095
Ccl25	NM_009138	0.000365	11.178	6.79722	5.61713	2.26592	1.1801	2.26592
Orc2	NM_008765	0.001221	8.17217	8.22325	7.0433	2.2657	1.17996	2.2657
Dvl1	NM_010091	0.000103	15.4189	8.59949	7.42046	2.26424	1.17903	2.26424
Nol4	NM_001161483	0.001712	7.47491	4.89444	3.71543	2.2642	1.179	2.2642
9430007A20Rik	ENSMUST00000030328	0.004515	5.75714	5.74358	4.56468	2.26404	1.1789	2.26404
Spns2	NM_153060	0.000857	8.96495	6.66569	5.4873	2.26323	1.17838	2.26323
	---	0.000865	8.9433	3.61039	2.43454	2.25927	1.17586	2.25927
Rnpep	NM_145417	0.000312	11.6387	8.36528	7.18968	2.25886	1.1756	2.25886
Arhgef3	NM_027871	0.005215	5.53269	7.93822	6.76344	2.25758	1.17478	2.25758
Phtf2	NM_172992	0.000496	10.3301	7.37212	6.19793	2.25665	1.17419	2.25665
Ccdc132	NM_001167750	0.000181	13.3683	8.23893	7.0652	2.25595	1.17373	2.25595
Btbd19	NR_024078	0.000102	15.4684	7.09316	5.91952	2.2558	1.17364	2.2558
Efna3	NM_010108	0.003628	6.11198	6.69594	5.5226	2.25533	1.17334	2.25533
A730017L22Rik	AK166222	8.62E-05	16.1393	6.6264	5.45371	2.25432	1.17269	2.25432
4931406P16Rik	BC060233	0.004876	5.63638	8.91836	7.7492	2.2488	1.16916	2.2488
Gm9873	ENSMUST00000063891	0.000421	10.7736	4.81372	3.64526	2.24771	1.16846	2.24771
Ankib1	NM_001003909	3.77E-05	19.8844	9.33708	8.17008	2.24545	1.167	2.24545

	---	0.004085	5.91719	8.69892	7.53486	2.24087	1.16406	2.24087
Cbln3	NM_019820	0.000371	11.1259	5.74295	4.57974	2.23955	1.16321	2.23955
Wwc1	NM_170779	8.91E-05	16.0065	6.47976	5.3186	2.23637	1.16116	2.23637
Hey1	NM_010423	0.001744	7.43876	6.19187	5.03082	2.23621	1.16105	2.23621
Syne2	NM_001005510	0.00152	7.71451	6.42176	5.26211	2.23403	1.15965	2.23403
Ankrd13b	NM_172945	0.000146	14.1105	7.67036	6.51105	2.23351	1.15931	2.23351
Isg20	NM_020583	0.000141	14.2362	8.43051	7.27141	2.23318	1.1591	2.23318
Asap1	NM_010026	0.00015	14.0171	9.95262	8.79394	2.23253	1.15868	2.23253
Itgb5	NM_001145884	0.000301	11.7381	11.3992	10.2423	2.22986	1.15695	2.22986
Trim37	NM_197987	0.000516	10.2213	9.03418	7.87755	2.22937	1.15664	2.22937
Tsta3	NM_031201	0.002528	6.73838	8.78549	7.63022	2.22726	1.15527	2.22726
Abca5	NM_147219	8.66E-06	28.7958	8.62188	7.46684	2.2269	1.15504	2.2269
	---	0.001785	7.39318	4.73849	3.58364	2.22661	1.15485	2.22661
	---	0.003032	6.41666	8.24478	7.09104	2.22489	1.15374	2.22489
Thumpd3	NM_008188	0.005559	5.43576	8.21583	7.0622	2.22473	1.15363	2.22473
	---	0.006003	5.3207	9.15371	8.00048	2.22412	1.15323	2.22412
Sf3a1	NM_026175	9.18E-05	15.8827	8.30392	7.15073	2.22407	1.1532	2.22407
Tspan32	NM_020286	0.000437	10.6709	5.22328	4.0707	2.22312	1.15259	2.22312
Inppl1	NM_010567	0.000237	12.4784	9.6896	8.53723	2.22278	1.15237	2.22278
Coro1c	NM_011779	0.000592	9.86622	10.14	8.98923	2.22039	1.15081	2.22039
Sbf1	NM_001170561	0.000379	11.0665	9.39314	8.24303	2.2193	1.15011	2.2193
Xrcc5	NM_009533	0.00078	9.18651	9.1571	8.00768	2.21825	1.14942	2.21825
Edem2	NM_145537	0.000159	13.8252	7.84201	6.69328	2.21718	1.14873	2.21718
	---	0.000637	9.68306	9.95175	8.80302	2.21718	1.14873	2.21718
Rhbdd1	NM_029777	0.003972	5.96291	9.1329	7.98517	2.21565	1.14773	2.21565
Rsbn1l	NM_001080977	0.000148	14.0617	9.24822	8.10084	2.2151	1.14737	2.2151
Tgfb1	NM_011577	0.000389	10.9908	9.66841	8.52188	2.2138	1.14653	2.2138
Tubg1	NM_134024	0.000524	10.1833	8.59051	7.44675	2.20957	1.14376	2.20957
Jakmip3	NM_028708	0.003317	6.26233	5.66731	4.52498	2.20737	1.14233	2.20737
Chd7	NM_001081417	0.00061	9.78854	6.69278	5.5571	2.19722	1.13568	2.19722
Myo7a	NM_008663	0.000663	9.58154	8.88433	7.74902	2.19667	1.13532	2.19667

Slc26a2	NM_007885	0.000258	12.2157	7.56185	6.42701	2.19595	1.13485	2.19595
Catsper2	NM_153075	0.003096	6.38013	6.07164	4.9375	2.19488	1.13414	2.19488
Tcf7	NM_009331	0.000658	9.60215	6.12708	4.99404	2.1932	1.13304	2.1932
Rnasen	NM_001130149	0.00198	7.19237	8.52232	7.38937	2.19307	1.13295	2.19307
2700078E11Rik	NM_030197	0.00012	14.8478	9.69733	8.5645	2.19288	1.13283	2.19288
4833420G17Rik	NM_001113550	0.000988	8.63854	9.14682	8.01403	2.19282	1.13279	2.19282
Lrrc8d	NM_178701	0.001382	7.90957	7.21836	6.08597	2.1922	1.13238	2.1922
	---	0.003693	6.08256	6.1037	4.9714	2.19208	1.1323	2.19208
Mink1	NM_001045959	0.000543	10.088	8.13913	7.0086	2.18939	1.13053	2.18939
Fgd6	NM_053072	0.000425	10.7495	5.82837	4.69875	2.18801	1.12962	2.18801
Sri	NM_025618	2.65E-05	21.7446	9.97429	8.84679	2.1848	1.1275	2.1848
Haghl	NM_026897	0.000962	8.69803	7.83093	6.7043	2.18348	1.12663	2.18348
Plekhh3	NM_146030	0.005763	5.38138	7.6668	6.54025	2.18337	1.12655	2.18337
Arf3	NM_007478	0.000658	9.60147	9.85631	8.73079	2.1818	1.12552	2.1818
Chn1	NM_001113246	0.003008	6.43003	4.9628	3.83775	2.1811	1.12505	2.1811
Osbpl2	NM_144500	0.002343	6.87636	9.24689	8.12187	2.18106	1.12503	2.18106
	---	0.004484	5.76792	5.06394	3.93952	2.18014	1.12442	2.18014
Mdh1	NM_008618	0.000109	15.2068	10.9745	9.85155	2.17792	1.12295	2.17792
Apol7a	NM_029419	0.002828	6.53786	4.52114	3.39886	2.17691	1.12228	2.17691
Cotl1	NM_028071	0.00102	8.56557	7.22657	6.10681	2.17312	1.11977	2.17312
Ercc2	NM_007949	0.000778	9.19249	7.04057	5.92183	2.17156	1.11873	2.17156
Gpr89	NM_026229	0.000328	11.4815	8.35465	7.23602	2.17141	1.11863	2.17141
Tsc22d3	NM_001077364	0.004368	5.80972	6.7556	5.63904	2.1683	1.11656	2.1683
Dci	NM_010023	9.54E-05	15.7309	9.23343	8.11716	2.16787	1.11628	2.16787
Dgkd	NM_177646	0.000651	9.62602	8.37299	7.25847	2.16523	1.11452	2.16523
Snap47	NM_144521	0.000704	9.43389	9.88383	8.76946	2.165	1.11437	2.165
Spg20	NM_144895	0.000522	10.1925	9.18062	8.06671	2.16432	1.11392	2.16432
Rnf181	NM_025607	0.000202	12.9929	9.77459	8.66087	2.16404	1.11373	2.16404
Dsn1	NM_025853	0.00495	5.61318	7.16444	6.05274	2.16099	1.11169	2.16099
Wdr60	NM_146039	0.002715	6.61029	6.84711	5.73578	2.16046	1.11134	2.16046
Snx15	NM_026912	8.79E-05	16.062	8.69491	7.5838	2.16011	1.11111	2.16011

Tlr3	NM_126166	0.002505	6.75434	6.78022	5.67007	2.15868	1.11015	2.15868
Cerk	NM_145475	0.001996	7.17743	7.44438	6.33457	2.15818	1.10982	2.15818
Traf4	NM_009423	0.001614	7.59278	8.30487	7.19512	2.15808	1.10975	2.15808
Cited2	NM_010828	0.004387	5.80289	10.0612	8.95148	2.15807	1.10974	2.15807
9030418K01Rik	NM_001081289	0.001521	7.71288	6.42586	5.31891	2.1539	1.10695	2.1539
Ppp1r9a	NM_181595	0.000824	9.05722	5.65215	4.54721	2.1509	1.10494	2.1509
Dhcr24	NM_053272	0.006627	5.17545	7.76166	6.65676	2.15084	1.1049	2.15084
Tob1	NM_009427	0.001055	8.49106	9.30287	8.19902	2.14928	1.10385	2.14928
Tgs1	NM_054089	0.005075	5.57459	7.38194	6.27814	2.1492	1.1038	2.1492
Gdpd1	NM_025638	0.00335	6.24556	8.6913	7.58782	2.14872	1.10348	2.14872
6330407J23Rik	NM_026138	0.003195	6.32615	5.21257	4.10983	2.14762	1.10274	2.14762
Pepd	NM_008820	0.000542	10.092	8.29276	7.19017	2.1474	1.10259	2.1474
	---	0.005115	5.56261	4.53733	3.43482	2.14728	1.10251	2.14728
Clcn3	NM_173874	5.09E-05	18.4426	7.09126	5.98886	2.14712	1.1024	2.14712
Smc3	NM_007790	3.39E-05	20.4369	10.1409	9.03898	2.14639	1.10191	2.14639
	---	0.000825	9.05309	10.5673	9.46552	2.14612	1.10173	2.14612
Gm906	BC147515	0.001252	8.11765	4.88223	3.78052	2.14609	1.10171	2.14609
Fubp1	NM_057172	0.00412	5.90331	10.128	9.02722	2.14465	1.10074	2.14465
D5Ertd579e	NM_001081232	0.002055	7.12149	7.44321	6.34337	2.14331	1.09984	2.14331
Tab1	NM_025609	0.001034	8.53474	7.53235	6.43312	2.14241	1.09923	2.14241
Zfp386	NM_001004066	0.002734	6.59755	8.39677	7.29758	2.14234	1.09919	2.14234
Ptprj	NM_008982	0.006447	5.21549	7.1643	6.06588	2.1412	1.09842	2.1412
Tmem8	NM_021793	8.92E-05	16.0026	6.26744	5.16904	2.14118	1.0984	2.14118
Hdac7	NM_019572	0.001834	7.34038	8.78011	7.68214	2.14053	1.09797	2.14053
Rpf1	NM_027371	0.004505	5.76055	9.18984	8.09212	2.14017	1.09773	2.14017
Slc9a3r2	NM_023055	0.000741	9.30974	7.44326	6.34554	2.14016	1.09772	2.14016
Tnr	NM_022312	0.001005	8.59862	5.53495	4.43825	2.13866	1.0967	2.13866
	---	0.006544	5.19388	5.33101	4.2357	2.13659	1.09531	2.13659
Usp6nl	NM_181399	0.004215	5.8669	7.6645	6.56948	2.13616	1.09502	2.13616
Gosr1	NM_016810	0.001476	7.77459	10.0921	8.9977	2.13523	1.09439	2.13523
Serpinb9b	NM_011452	0.000292	11.8345	6.1424	5.04817	2.13498	1.09422	2.13498

Sfi1	NM_030207	0.000537	10.1164	7.63076	6.53697	2.13434	1.09379	2.13434
Slc6a9	NM_008135	0.000734	9.33088	9.53816	8.44459	2.13403	1.09358	2.13403
Chka	NM_013490	0.005258	5.52021	7.30372	6.21059	2.13336	1.09313	2.13336
Unc5b	NM_029770	0.006253	5.26038	8.67498	7.58205	2.13307	1.09293	2.13307
Ppp2r5d	NM_009358	6.74E-05	17.1789	8.79155	7.69868	2.13298	1.09287	2.13298
Fscn1	NM_007984	0.001921	7.25088	10.2132	9.12193	2.13063	1.09128	2.13063
Efhc2	NM_028916	0.000523	10.1858	4.99258	3.9047	2.12561	1.08788	2.12561
	---	0.002993	6.43899	8.46406	7.37723	2.12406	1.08682	2.12406
Ints2	NM_027421	0.000303	11.7201	8.52966	7.4438	2.12264	1.08586	2.12264
Samd9l	NM_010156	0.00237	6.85572	7.49512	6.40936	2.12249	1.08575	2.12249
Tbl3	NM_145396	0.00301	6.42894	8.44376	7.35839	2.12192	1.08537	2.12192
Ppef1	NM_011147	0.002649	6.65423	4.4388	3.35552	2.11885	1.08328	2.11885
Uros	NM_009479	0.000365	11.1725	7.65058	6.56765	2.11833	1.08293	2.11833
Rhobtb2	NM_153514	0.004346	5.8178	6.96879	5.88594	2.11821	1.08285	2.11821
	---	0.002692	6.62526	5.79548	4.71306	2.11758	1.08242	2.11758
Rab28	NM_027295	0.000368	11.1505	9.02215	7.94076	2.11607	1.08139	2.11607
Zfp827	NM_178267	0.006991	5.09831	8.00549	6.92508	2.11464	1.08041	2.11464
	---	0.00012	14.8421	5.65813	4.57789	2.11439	1.08024	2.11439
2610507B11Rik	NM_001002004	6.20E-05	17.5447	10.4345	9.35481	2.11359	1.0797	2.11359
Synrg	NM_001115009	0.000738	9.31774	7.38215	6.30445	2.11067	1.0777	2.11067
Tmem189	NM_145538	0.001532	7.69764	8.69144	7.61381	2.11057	1.07763	2.11057
Chd7	NM_001081417	0.001918	7.25354	5.24336	4.167	2.1087	1.07635	2.1087
Prss23	NM_029614	0.000933	8.76643	6.35732	5.28099	2.10867	1.07633	2.10867
	---	0.000717	9.38895	7.39687	6.32073	2.10839	1.07614	2.10839
2310035C23Rik	NM_173187	4.58E-05	18.9339	8.48258	7.40782	2.10638	1.07476	2.10638
	---	0.001189	8.22901	11.37	10.2954	2.10615	1.07461	2.10615
2010011I20Rik	NM_025912	0.005629	5.41691	7.45535	6.38126	2.1054	1.07409	2.1054
Rnu3b1	NR_004415	0.000941	8.7486	9.50206	8.42805	2.10528	1.07401	2.10528
Rnu3b1	NR_004415	0.000941	8.7486	9.50206	8.42805	2.10528	1.07401	2.10528
Rnu3b1	NR_004415	0.000941	8.7486	9.50206	8.42805	2.10528	1.07401	2.10528
Rnu3b1	NR_004415	0.000941	8.7486	9.50206	8.42805	2.10528	1.07401	2.10528

Thnsl1	NM_177588	0.000585	9.89774	6.59884	5.52601	2.10355	1.07283	2.10355
Pik3c2a	NM_011083	0.001594	7.61746	8.87	7.79894	2.10096	1.07105	2.10096
Chd7	NM_001081417	0.000355	11.2576	7.65359	6.58255	2.10095	1.07104	2.10095
Tm7sf2	NM_028454	9.97E-05	15.5541	7.43523	6.36474	2.10014	1.07049	2.10014
Rnf157	NM_027258	0.002494	6.76273	6.47776	5.40776	2.09943	1.06999	2.09943
Hist1h2bg	NM_178196	0.002094	7.08603	6.18483	5.11517	2.09894	1.06966	2.09894
Nefl	NM_010910	0.000444	10.6253	5.52438	4.45503	2.09849	1.06935	2.09849
Apobec3	NM_001160415	0.006111	5.29413	8.07717	7.008	2.09822	1.06917	2.09822
Nck2	NM_010879	0.000423	10.759	7.78596	6.717	2.09793	1.06896	2.09793
Wnt9a	NM_139298	0.003151	6.35005	7.61238	6.54542	2.09502	1.06696	2.09502
Tead4	NM_011567	0.001213	8.18574	6.52293	5.45632	2.09451	1.06661	2.09451
A430105I19Rik	NM_001001982	0.001434	7.83385	7.84158	6.77536	2.09394	1.06622	2.09394
Vezf1	NM_016686	0.000575	9.93995	9.07274	8.00708	2.09314	1.06567	2.09314
Tbc1d22a	NM_145476	0.002181	7.00963	8.36504	7.30015	2.09202	1.06489	2.09202
Chd7	NM_001081417	3.53E-05	20.218	6.33663	5.27188	2.0918	1.06474	2.0918
Mtmr14	NM_026849	0.004721	5.6868	8.62153	7.55805	2.08997	1.06348	2.08997
Msi2	NM_054043	0.000128	14.604	9.43058	8.36829	2.08824	1.06229	2.08824
Pard6b	NM_021409	6.21E-05	17.5384	6.30057	5.23843	2.08803	1.06214	2.08803
0610010B08Rik	ENSMUST00000108991	0.005169	5.5463	9.95445	8.89269	2.08747	1.06176	2.08747
Gm14430	NM_001100415	0.005169	5.5463	9.95445	8.89269	2.08747	1.06176	2.08747
1E+08	NM_001099327	0.005169	5.5463	9.95445	8.89269	2.08747	1.06176	2.08747
Gm14430	NM_001100415	0.005169	5.5463	9.95445	8.89269	2.08747	1.06176	2.08747
0610010B08Rik	ENSMUST00000108991	0.005169	5.5463	9.95445	8.89269	2.08747	1.06176	2.08747
0610010B08Rik	ENSMUST00000108991	0.005169	5.5463	9.95445	8.89269	2.08747	1.06176	2.08747
0610010B08Rik	ENSMUST00000108991	0.005169	5.5463	9.95445	8.89269	2.08747	1.06176	2.08747
Chac1	NM_026929	0.007056	5.08507	7.89874	6.83942	2.08395	1.05932	2.08395
	---	0.000302	11.7331	4.0158	2.9567	2.08363	1.0591	2.08363
Ciapin1	NM_134141	0.000227	12.617	8.49724	7.43979	2.08125	1.05745	2.08125
Psme2	NM_011190	0.001558	7.66403	7.70162	6.64647	2.07793	1.05515	2.07793

Vps53	NM_026664	0.000544	10.0852	8.48352	7.42877	2.07736	1.05475	2.07736
Pldn	NM_019788	0.002449	6.79532	7.99638	6.94268	2.07584	1.0537	2.07584
Epn2	NM_010148	0.00049	10.3625	8.18589	7.13385	2.07346	1.05204	2.07346
Ttll1	NM_178869	0.000648	9.63855	8.70371	7.6518	2.07328	1.05191	2.07328
	---	0.000601	9.82745	6.60937	5.55789	2.07266	1.05148	2.07266
Zcwpw1	NM_001005426	0.002594	6.69149	6.53074	5.47952	2.07228	1.05122	2.07228
Lrrc16a	NM_026825	0.001764	7.41636	8.17578	7.12488	2.07182	1.0509	2.07182
Efna3	NM_010108	0.004901	5.62846	5.39442	4.3437	2.07156	1.05072	2.07156
	---	0.00124	8.13879	10.9281	9.87768	2.07118	1.05045	2.07118
Zdhhc13	NM_028031	7.60E-05	16.6655	8.78254	7.73277	2.07019	1.04977	2.07019
Pak1	NM_011035	0.001795	7.38238	7.95723	6.90783	2.06966	1.0494	2.06966
Abat	NM_172961	0.005952	5.33339	8.02283	6.97355	2.06949	1.04927	2.06949
Zfp238	NM_001012330	0.001057	8.48751	7.23671	6.18765	2.06919	1.04906	2.06919
C330019L16Rik	NM_001034857	0.000761	9.24353	5.46035	4.4115	2.06888	1.04885	2.06888
Got1	NM_010324	0.004689	5.69768	10.2946	9.24605	2.06848	1.04857	2.06848
Sgms1	NM_001168525	0.000287	11.8876	8.93402	7.88598	2.06772	1.04804	2.06772
	---	0.001224	8.1672	9.09426	8.04661	2.06716	1.04765	2.06716
	---	0.001725	7.46093	9.72593	8.67874	2.0665	1.04719	2.0665
Ptprj	NM_008982	0.006456	5.21346	7.44069	6.39465	2.06485	1.04604	2.06485
Ppp6r3	NM_028999	2.85E-05	21.3491	10.0963	9.05039	2.06462	1.04588	2.06462
Fam49b	NM_144846	0.001767	7.41277	8.13037	7.08495	2.06396	1.04542	2.06396
Tbrg4	NM_134011	0.001015	8.57689	8.09641	7.05107	2.06385	1.04534	2.06385
Rb1	NM_009029	0.006049	5.30927	7.37418	6.32899	2.06365	1.0452	2.06365
5031425E22Rik	BC050254	0.001914	7.25731	6.92861	5.8848	2.06168	1.04382	2.06168
Eif2b2	NM_145445	0.000196	13.103	8.42025	7.3768	2.06114	1.04344	2.06114
Ormdl1	NM_145517	0.000731	9.34133	6.61943	5.57623	2.0608	1.0432	2.0608
Pstpip2	NM_013831	0.001137	8.32681	4.92796	3.88506	2.06036	1.0429	2.06036
Ptrf	NM_008986	4.77E-05	18.7445	9.90544	8.86379	2.05859	1.04165	2.05859
Dennd2a	NM_172477	0.000158	13.8373	8.35407	7.31266	2.05823	1.04141	2.05823
Fign	NM_021716	0.003866	6.0071	4.84187	3.80046	2.05823	1.0414	2.05823
Prr5l	NM_001083810	0.003468	6.18747	4.63249	3.59111	2.0582	1.04139	2.0582

Ces4a	NM_146213	0.001182	8.24105	5.67266	4.63153	2.05784	1.04113	2.05784
Patz1	NM_019574	0.000663	9.58005	7.64666	6.60641	2.05658	1.04025	2.05658
	---	0.003876	6.00271	7.40343	6.3636	2.05599	1.03983	2.05599
Abca2	NM_007379	0.001129	8.34092	7.6526	6.61342	2.05506	1.03918	2.05506
Casp3	NM_009810	0.000257	12.2238	8.75419	7.71559	2.05423	1.03859	2.05423
Cenpt	NM_177150	0.000692	9.47592	7.54686	6.50973	2.05214	1.03713	2.05214
Rhebl1	NM_026967	0.002422	6.81585	6.01662	4.98114	2.04979	1.03548	2.04979
Trit1	NM_025873	0.003122	6.36617	7.287	6.25167	2.04958	1.03533	2.04958
Eed	NM_021876	0.001986	7.18654	8.47843	7.44561	2.04602	1.03282	2.04602
2310001A20Rik	NM_027977	3.31E-06	36.6575	8.747	7.71426	2.04591	1.03274	2.04591
Ddx23	NM_001080981	3.92E-05	19.7001	9.17071	8.14092	2.04173	1.02979	2.04173
	---	0.006986	5.09931	8.84004	7.81096	2.04072	1.02908	2.04072
AI606181	BC116839	4.39E-05	19.1362	6.03527	5.0063	2.04056	1.02897	2.04056
Schip1	NM_001113421	2.42E-05	22.2293	8.28968	7.26073	2.04055	1.02896	2.04055
Mtap	NM_024433	0.004576	5.73586	8.54612	7.51823	2.03904	1.02789	2.03904
Nol11	NM_133702	0.00258	6.70173	8.79518	7.76802	2.038	1.02716	2.038
Kcnh2	NM_013569	0.002899	6.49436	6.1003	5.07471	2.03578	1.02558	2.03578
	---	0.001966	7.20571	8.86202	7.83695	2.03506	1.02507	2.03506
Med1	NM_013634	0.000515	10.2278	8.83026	7.80572	2.03431	1.02454	2.03431
	---	0.001517	7.71778	9.98126	8.95761	2.03306	1.02365	2.03306
9-Mar	NM_001033262	0.004535	5.75006	6.42201	5.39968	2.03119	1.02233	2.03119
Nudcd1	NM_026149	0.000544	10.0822	9.13545	8.11445	2.02932	1.021	2.02932
	---	0.002764	6.57881	7.27063	6.25029	2.02839	1.02034	2.02839
Frk	NM_001159544	0.002536	6.7322	7.77865	6.75873	2.02781	1.01992	2.02781
Sart3	NM_016926	0.000185	13.2947	8.01916	6.99955	2.02736	1.0196	2.02736
Znrf3	NM_001080924	0.003989	5.95583	7.1559	6.13678	2.02668	1.01912	2.02668
S100a10	NM_009112	0.001291	8.05232	12.2044	11.1866	2.02475	1.01775	2.02475
Arhgef10	NM_172751	0.00103	8.54326	7.51194	6.49699	2.02084	1.01496	2.02084
Hpcal1	NM_016677	0.006702	5.15913	8.70101	7.6861	2.02078	1.01491	2.02078
Smap2	NM_133716	0.000835	9.02487	7.89771	6.88296	2.02055	1.01475	2.02055
Elp4	NM_023876	0.002549	6.72297	8.04672	7.03256	2.01973	1.01416	2.01973

Pdzd8	NM_001033222	0.002612	6.6792	9.12087	8.10685	2.01953	1.01402	2.01953
Strn	NM_011500	0.001856	7.31736	8.276	7.26214	2.01932	1.01387	2.01932
Slc48a1	NM_026353	0.004564	5.74001	10.3985	9.38475	2.01921	1.01379	2.01921
	---	0.000755	9.26553	4.98287	3.96909	2.0192	1.01378	2.0192
Med24	NM_011869	0.001695	7.49533	9.04701	8.03329	2.0191	1.01371	2.0191
	---	0.002197	6.99573	8.53078	7.51822	2.01748	1.01255	2.01748
Lmbrd2	NM_177178	0.00032	11.5564	8.9109	7.89863	2.01708	1.01227	2.01708
Sec14l2	NM_144520	0.00152	7.71437	6.26951	5.25753	2.01667	1.01198	2.01667
	---	8.85E-05	16.031	10.122	9.1107	2.01572	1.01129	2.01572
S100a3	NM_011310	0.000191	13.193	5.74131	4.73012	2.01558	1.01119	2.01558
Atp6v0b	NM_033617	0.001028	8.5491	10.8454	9.83448	2.01522	1.01094	2.01522
Zfp53	NM_013843	0.006096	5.29786	7.28102	6.27092	2.01406	1.01011	2.01406
Ipp	NM_008389	0.000216	12.7764	6.30758	5.29845	2.01269	1.00912	2.01269
Il2rb	NM_008368	0.005381	5.48491	5.10308	4.09417	2.01239	1.00891	2.01239
Atxn10	NM_016843	0.000218	12.7513	11.0149	10.006	2.01236	1.00889	2.01236
Crlf1	NM_018827	0.005178	5.54371	9.6537	8.64487	2.01228	1.00883	2.01228
Sf3b2	NM_030109	0.00028	11.9638	9.34525	8.33668	2.0119	1.00856	2.0119
Xpo4	NM_020506	0.001185	8.23645	8.32093	7.3126	2.01159	1.00834	2.01159
Tcfcp2	NM_033476	0.001432	7.83582	8.28185	7.27358	2.01149	1.00827	2.01149
Ccdc28a	NM_144820	0.00656	5.19034	6.54124	5.53362	2.0106	1.00762	2.0106
Ccdc136	NM_145574	0.000351	11.2891	7.62463	6.61736	2.0101	1.00727	2.0101
Yaf2	NR_028315	0.001912	7.2593	8.36473	7.3575	2.01006	1.00724	2.01006
Cd151	NM_009842	6.91E-05	17.0675	9.5792	8.57309	2.00849	1.00611	2.00849
Zfp788	NM_023363	0.000743	9.30321	7.37901	6.37383	2.0072	1.00518	2.0072
Zfp788	NM_023363	0.000743	9.30321	7.37901	6.37383	2.0072	1.00518	2.0072
Ppfibp1	NM_001170433	0.000932	8.77108	11.003	9.99867	2.00597	1.0043	2.00597
Usp20	NM_028846	0.004454	5.77858	7.06912	6.06485	2.00593	1.00427	2.00593
Polr3c	NM_028925	0.001195	8.21733	8.95455	7.95136	2.00442	1.00319	2.00442
Sgms2	NM_028943	0.003219	6.3135	6.90453	5.9018	2.00378	1.00273	2.00378
Mrpl47	NM_029017	0.007028	5.09072	6.43818	5.43571	2.00343	1.00247	2.00343
Lgals9	NM_010708	0.004155	5.88985	9.50825	8.50703	2.00169	1.00122	2.00169

Mlst8	NM_019988	5.06E-05	18.4683	8.19636	7.19536	2.00139	1.001	2.00139
Phf10	NM_024250	7.78E-05	16.5639	8.62532	7.62461	2.00098	1.00071	2.00098
Ndufs5	NM_001030274	0.000584	9.90059	7.49777	6.4972	2.00078	1.00056	2.00078
Ada	NM_007398	0.001149	8.30375	6.7311	5.73054	2.00077	1.00056	2.00077
Slc25a39	NM_026542	0.001132	8.33643	10.4512	9.4516	1.99945	0.999601	1.99945
Rfwd3	NM_146218	0.004999	5.59792	8.64312	7.64368	1.99922	0.999439	1.99922
Abca7	NM_013850	0.000265	12.1361	7.30161	6.30226	1.99909	0.999344	1.99909
Cdh2	NM_007664	0.006867	5.12393	8.61873	7.62141	1.9963	0.997327	1.9963
Plekho1	NM_023320	0.00158	7.63563	8.31792	7.3213	1.99533	0.996624	1.99533
Acadm	NM_007382	0.000877	8.90941	10.3059	9.31126	1.99256	0.994625	1.99256
Dhx32	NM_133941	0.000726	9.35845	7.88799	6.89343	1.99249	0.994569	1.99249
Rarg	NM_011244	0.001353	7.95488	8.29457	7.30078	1.99141	0.993789	1.99141
	---	0.006031	5.31378	4.80693	3.81462	1.98936	0.992307	1.98936
Ikbkg	NM_001136067	0.001482	7.7661	8.18803	7.19581	1.98924	0.992215	1.98924
Hif1an	NM_176958	0.003518	6.16341	7.69638	6.70463	1.98859	0.991747	1.98859
Lpar6	NM_175116	0.005083	5.57231	8.30674	7.31538	1.98806	0.991362	1.98806
Chd7	NM_001081417	0.000803	9.1159	5.87479	4.88387	1.98745	0.990919	1.98745
Smg5	NM_178246	0.001028	8.54767	7.91375	6.92366	1.98631	0.990089	1.98631
Zcchc4	NM_030185	0.003794	6.03773	7.51845	6.52922	1.98512	0.989223	1.98512
Tmem184a	NM_001161548	0.000544	10.0832	5.44389	4.45472	1.98505	0.989179	1.98505
Alg6	NM_001081264	0.003621	6.11518	6.72526	5.73621	1.98488	0.98905	1.98488
Crlf3	NM_018776	1.73E-06	43.1191	8.74471	7.75602	1.98438	0.988687	1.98438
Zfp706	NM_026521	0.000838	9.01712	10.2457	9.2579	1.9832	0.987832	1.9832
Slc3a1	NM_009205	0.005026	5.58966	5.57684	4.59023	1.98153	0.986614	1.98153
Copg2	NM_017478	0.005589	5.42761	9.78899	8.80351	1.97998	0.985485	1.97998
Tubgcp6	NM_001163319	5.91E-05	17.757	6.95912	5.97386	1.97967	0.985259	1.97967
Alox5ap	NM_009663	0.002628	6.66866	5.896	4.91107	1.97922	0.984934	1.97922
Atp6v1c1	NM_025494	0.001478	7.77139	10.3074	9.32296	1.97849	0.984399	1.97849
BC023105	BC023105	0.003841	6.01766	3.67179	2.68797	1.9777	0.983825	1.9777
Pigf	NM_008838	0.004802	5.66019	8.19899	7.2155	1.97723	0.983484	1.97723
Lrrc14	NM_145471	2.65E-05	21.735	8.2016	7.21816	1.97716	0.983431	1.97716

Cpd	NM_007754	0.000236	12.5001	9.18875	8.20535	1.97712	0.983402	1.97712
Cpox	NM_007757	0.00158	7.63627	7.88519	6.90188	1.977	0.983313	1.977
Tcf7l2	NM_001142918	0.000615	9.7697	8.19296	7.21123	1.97484	0.981738	1.97484
Ap1g1	NM_009677	0.000128	14.5936	9.62983	8.64876	1.97393	0.981069	1.97393
	---	0.00411	5.90746	8.79647	7.81543	1.97388	0.981038	1.97388
Apbb1	NM_009685	0.000409	10.8529	7.40845	6.42753	1.97373	0.980924	1.97373
Lss	NM_146006	0.00347	6.18643	7.34691	6.36632	1.97326	0.980584	1.97326
Psme1	NM_011189	0.000319	11.5658	10.2895	9.30928	1.97271	0.980181	1.97271
Irs2	NM_001081212	0.003566	6.14042	7.1492	6.17008	1.97126	0.979121	1.97126
Slc25a10	NM_013770	0.000347	11.3235	7.91845	6.93945	1.9711	0.978998	1.9711
Cdh22	NM_174988	0.000834	9.02655	6.14037	5.1622	1.96995	0.978162	1.96995
Lancl2	NM_133737	0.00606	5.30657	8.53343	7.55538	1.9698	0.978047	1.9698
Pmepa1	NM_022995	0.003814	6.0293	10.2712	9.29341	1.96948	0.977812	1.96948
Tusc5	NM_177709	0.004411	5.79411	5.99601	5.02033	1.96657	0.975684	1.96657
Rfwd3	NM_146218	0.005136	5.55612	8.61755	7.64287	1.96521	0.974682	1.96521
Bcas3	NM_138681	0.000219	12.7387	7.55571	6.58115	1.96504	0.97456	1.96504
Asxl1	NM_001039939	0.00508	5.57299	8.78485	7.81075	1.96441	0.974096	1.96441
Coq5	NM_026504	0.003233	6.30594	7.67382	6.70052	1.96332	0.973298	1.96332
Fam82a1	NM_201361	0.002036	7.13897	7.25459	6.2814	1.96318	0.97319	1.96318
Osgepl1	NM_028091	0.000408	10.8627	6.69105	5.71912	1.96146	0.971929	1.96146
Wbp2	NM_016852	0.000675	9.53563	8.82293	7.85203	1.96006	0.970901	1.96006
Hist1h4j	NM_178210	0.001851	7.32219	5.791	4.82026	1.95985	0.970743	1.95985
Zmym6	NM_177462	0.001154	8.29414	8.55804	7.58783	1.95913	0.970212	1.95913
Phlpp1	NM_133821	0.006583	5.18516	6.74772	5.77868	1.95754	0.969044	1.95754
Git1	NM_001004144	6.87E-05	17.0926	8.51857	7.54957	1.95749	0.969004	1.95749
Phf14	NM_029404	0.006049	5.30944	7.995	7.02601	1.95747	0.968992	1.95747
Terf1	NM_009352	0.000621	9.74632	7.81256	6.84367	1.95732	0.968883	1.95732
Ptar1	NM_028208	0.003192	6.32812	8.59575	7.62689	1.95729	0.968861	1.95729
Mars2	NM_175439	0.00098	8.65671	6.98923	6.02054	1.95707	0.968693	1.95707
Morn4	NM_198108	0.00146	7.79716	8.08179	7.11321	1.95692	0.968581	1.95692
Dcun1d4	NM_001190734	0.003884	5.99948	8.21764	7.25037	1.95514	0.967269	1.95514

Pcyt1b	NM_211138	0.001015	8.57726	6.5121	5.54529	1.95452	0.966812	1.95452
Krt14	NM_016958	0.002542	6.72832	5.33644	4.37077	1.95296	0.965663	1.95296
	---	0.003694	6.08186	9.68493	8.71934	1.95286	0.96559	1.95286
Spata6	NM_026470	0.002422	6.81565	7.04791	6.0827	1.95234	0.965205	1.95234
Hrc	NM_010473	0.00151	7.72754	5.89264	4.92821	1.95129	0.964427	1.95129
Trip12	NM_133975	0.000151	14.0047	11.1011	10.1376	1.95004	0.963502	1.95004
Pot1a	NM_133931	0.004719	5.68758	8.42524	7.46251	1.949	0.962735	1.949
Rpusd1	NM_028009	0.000957	8.71075	7.56867	6.60645	1.9483	0.962218	1.9483
Trpm6	NM_153417	0.00027	12.0752	5.36832	4.40639	1.94793	0.961938	1.94793
Nppb	NM_008726	0.006889	5.11949	5.67778	4.71592	1.94782	0.961859	1.94782
Prss42	NM_153099	0.002937	6.47167	5.01033	4.0485	1.94779	0.961837	1.94779
Cpsf1	NM_001164173	0.000556	10.0264	8.13827	7.17725	1.94668	0.961019	1.94668
Tubb2b	NM_023716	0.001157	8.28881	7.58656	6.62596	1.94612	0.960598	1.94612
2700046G09Rik	NR_033198	0.000105	15.3498	6.33048	5.371	1.94461	0.959482	1.94461
Aco2	NM_080633	0.000115	15.0096	11.2261	10.268	1.94275	0.9581	1.94275
Zc3hc1	NM_172735	0.002579	6.70182	8.13728	7.18309	1.93749	0.954188	1.93749
Smyd4	NM_001102611	0.000393	10.9652	6.92692	5.97276	1.93745	0.95416	1.93745
Oip5	NM_001042653	0.001599	7.61217	4.95537	4.0013	1.93733	0.954067	1.93733
Krit1	NR_033173	0.000311	11.6441	8.92669	7.97324	1.9365	0.953449	1.9365
BC052040	NM_001145898	0.000422	10.7641	7.18063	6.22752	1.93603	0.953105	1.93603
Zfp661	NM_028141	0.000174	13.497	7.04661	6.09352	1.93601	0.953089	1.93601
Ssfa2	NM_080558	0.00483	5.65133	7.50222	6.54954	1.93546	0.952679	1.93546
	---	0.003306	6.26784	6.23247	5.28	1.93518	0.952468	1.93518
Hrsp12	NM_008287	0.000386	11.0128	9.40337	8.45123	1.93475	0.952146	1.93475
	---	0.001371	7.92686	8.30395	7.35226	1.93414	0.951693	1.93414
Inpp5j	NM_172439	0.002296	6.91431	5.92867	4.97854	1.93205	0.950134	1.93205
	---	0.000468	10.484	11.302	10.3523	1.93148	0.949708	1.93148
Scd4	NM_183216	0.006686	5.16252	5.02946	4.07976	1.93147	0.9497	1.93147
6-Mar	NM_172606	0.000665	9.57355	10.3415	9.39182	1.93145	0.949687	1.93145
Arhgef10l	NM_172415	0.006542	5.19427	7.42267	6.47353	1.93072	0.949137	1.93072
Foxo1	NM_019739	0.002131	7.05285	7.73882	6.78992	1.9304	0.948899	1.9304

Rqcd1	NM_021383	0.003921	5.984	8.54903	7.60018	1.93034	0.948853	1.93034
Mcrs1	NM_016766	3.04E-05	20.9899	8.33585	7.38828	1.92862	0.947567	1.92862
Agfg1	NM_010472	2.53E-05	21.9823	10.9671	10.0208	1.92702	0.946369	1.92702
Cnnm2	NM_033569	0.006941	5.10851	8.08083	7.13489	1.92645	0.945947	1.92645
Cd2ap	NM_009847	0.005999	5.3216	9.32632	8.38464	1.92077	0.941682	1.92077
Adam8	NM_007403	0.000561	10.0042	6.48785	5.54644	1.9204	0.941407	1.9204
Anubl1	NM_001081317	0.001063	8.47357	5.59635	4.65507	1.92022	0.941273	1.92022
Smc5	NM_153808	0.000435	10.6848	9.98299	9.04296	1.91857	0.940031	1.91857
Tapbpl	NM_145391	0.001714	7.47283	7.53775	6.59887	1.91704	0.938883	1.91704
Pigy	NM_025574	0.000395	10.9494	7.17215	6.23457	1.91531	0.937578	1.91531
Zfp235	NM_019941	0.005827	5.36482	6.62375	5.68632	1.91512	0.937434	1.91512
Tns3	NM_001083587	0.001364	7.938	8.9794	8.04213	1.91491	0.937275	1.91491
	---	0.004024	5.94168	5.28196	4.3451	1.91435	0.936857	1.91435
Nck2	NM_010879	0.000786	9.16756	8.41079	7.4754	1.91241	0.935393	1.91241
Pon2	NM_183308	0.005645	5.41249	8.63152	7.69718	1.91102	0.934344	1.91102
D330041H03Rik	NR_033554	0.00335	6.24568	5.88509	4.95152	1.91	0.933576	1.91
Hn1l	NM_198937	0.006032	5.31344	8.86733	7.93503	1.90832	0.932305	1.90832
Mapk8ip3	NM_013931	0.000226	12.6363	8.6633	7.73133	1.90787	0.931966	1.90787
Extl1	NM_019578	0.002862	6.51691	5.98887	5.05806	1.90634	0.930806	1.90634
Slurp1	NM_020519	0.004427	5.78827	6.0627	5.13279	1.90515	0.929908	1.90515
Ift140	NM_134126	0.00121	8.19096	7.572	6.6437	1.90303	0.928296	1.90303
Gm3258	NM_011509	0.005826	5.36511	9.1971	8.26928	1.9024	0.927817	1.9024
Wbp1	NM_016757	0.001831	7.3438	9.57942	8.65179	1.90215	0.927634	1.90215
Immt	NM_029673	0.00029	11.8575	9.89333	8.96612	1.9016	0.927213	1.9016
	---	0.003454	6.19421	9.92042	8.99354	1.90117	0.926886	1.90117
Brp16	NM_021555	0.002232	6.96644	7.62639	6.70142	1.89865	0.924974	1.89865
Zc3h3	NM_172121	0.000159	13.8287	7.22746	6.30326	1.89763	0.924201	1.89763
Prg2	NM_008920	0.000456	10.5552	5.05797	4.13389	1.89748	0.924082	1.89748
Brms1	NM_134155	5.37E-05	18.1935	7.65634	6.73404	1.89513	0.922298	1.89513
Kirrel	NM_001170985	0.004059	5.92743	9.25476	8.33277	1.89473	0.92199	1.89473
Mcts2	NM_025543	0.004695	5.69558	8.3299	7.4085	1.89395	0.921397	1.89395

Dusp3	NM_028207	0.001744	7.43935	7.79846	6.87736	1.89356	0.9211	1.89356
Lzts2	NM_145503	0.001427	7.84355	8.52193	7.60127	1.89298	0.920663	1.89298
2410042D21Rik	BC080661	0.003151	6.34987	8.58062	7.6603	1.89253	0.920314	1.89253
Zcchc10	NM_026479	0.001547	7.67839	5.28485	4.36482	1.89215	0.92003	1.89215
	---	0.004413	5.79338	6.95498	6.03522	1.8918	0.919761	1.8918
	---	0.000836	9.02276	11.5853	10.6657	1.89164	0.919639	1.89164
	---	0.006892	5.11884	6.68237	5.76319	1.89105	0.919188	1.89105
1110012J17Rik	NM_001114098	0.004548	5.74575	6.42602	5.50683	1.89105	0.919186	1.89105
Pkd1	NM_013630	1.28E-05	26.097	8.38814	7.46969	1.89008	0.918445	1.89008
Ampd3	NM_009667	0.000789	9.15853	6.76157	5.8434	1.88971	0.918167	1.88971
Vps25	NM_026776	0.001619	7.58632	9.74397	8.82631	1.88906	0.917667	1.88906
Gm14391	NM_001099308	0.001375	7.92082	11.1748	10.2573	1.88877	0.91745	1.88877
Gm14391	NM_001099308	0.001375	7.92082	11.1748	10.2573	1.88877	0.91745	1.88877
Gas2l1	NM_144560	0.000425	10.7483	7.39596	6.47924	1.88781	0.916716	1.88781
Tbp	NM_013684	0.000786	9.16774	8.28531	7.3688	1.88755	0.916512	1.88755
Cnot1	NM_153164	0.001231	8.15358	9.3583	8.44208	1.88717	0.916222	1.88717
Tubgcp6	NM_001163319	0.00027	12.0715	7.43845	6.52369	1.88525	0.914758	1.88525
	---	0.006485	5.20695	9.52216	8.60755	1.88506	0.914608	1.88506
	---	0.000323	11.5349	10.2808	9.36656	1.88455	0.914217	1.88455
Dnmt3a	NM_007872	0.000747	9.28815	7.08286	6.16891	1.88419	0.913943	1.88419
Ppp1r16a	NM_033371	0.000452	10.5786	7.6459	6.73219	1.88388	0.913706	1.88388
Ankrd28	NM_001024604	0.004404	5.79645	9.83813	8.92479	1.88341	0.913348	1.88341
Mfsd7b	NM_001081259	0.003285	6.27884	7.78271	6.86973	1.88293	0.912977	1.88293
Plxnb2	NM_138749	5.82E-05	17.8244	10.5853	9.6724	1.88277	0.912855	1.88277
Gm10209	ENSMUST00000087544	4.27E-05	19.2744	5.88221	4.96983	1.88215	0.912381	1.88215
Gm10825	NR_028580	0.004831	5.65085	6.61781	5.70574	1.88175	0.912072	1.88175
Hdgf	NM_008231	0.001958	7.21359	9.77111	8.85974	1.88082	0.911362	1.88082
Pitpna	NM_008850	0.000455	10.559	10.8076	9.89751	1.87921	0.910124	1.87921
Chuk	NM_007700	0.000881	8.90056	8.90099	7.99139	1.87853	0.909603	1.87853
Tbl1xr1	NM_030732	0.000367	11.158	9.05589	8.14641	1.87836	0.909475	1.87836

Mrpl18	NM_026310	0.001329	7.99154	9.76428	8.85528	1.87774	0.908994	1.87774
Arf2	NM_007477	0.000234	12.5175	9.13218	8.2234	1.87745	0.908775	1.87745
Zmiz2	NM_028601	0.000221	12.7063	7.5371	6.62844	1.87731	0.908666	1.87731
Chd7	NM_001081417	0.001684	7.50756	7.27923	6.37136	1.87627	0.907871	1.87627
Immp1l	NM_028260	0.001645	7.55434	8.44736	7.53968	1.87603	0.907683	1.87603
Slc9a5	NM_001081332	0.001158	8.28686	6.31609	5.40863	1.87574	0.907458	1.87574
Pola2	NM_008893	0.003169	6.34042	7.65584	6.74865	1.87539	0.907188	1.87539
Tapbp	NM_001025313	0.000103	15.432	10.8392	9.93209	1.87533	0.907145	1.87533
Esf1	NM_001081090	0.002506	6.75408	8.68497	7.77794	1.87518	0.907032	1.87518
Hdac5	NM_001077696	0.000267	12.1058	9.01618	8.10948	1.87475	0.906697	1.87475
	---	0.004163	5.8867	9.86044	8.95401	1.87441	0.906438	1.87441
Gtf2i	NM_001080746	0.003516	6.16411	10.166	9.26106	1.87241	0.904898	1.87241
Kcnn1	NM_032397	0.000753	9.27176	6.61753	5.71281	1.87219	0.904728	1.87219
Zfp955a	NM_029952	0.004857	5.64258	8.43608	7.53179	1.87163	0.904296	1.87163
Gm6924	NM_001177567	0.000731	9.34207	4.96303	4.05887	1.87146	0.904166	1.87146
Rfc4	NM_145480	0.005966	5.32993	7.27134	6.36719	1.87144	0.904152	1.87144
Cgn	NM_001037711	0.004919	5.62279	6.19989	5.29706	1.86973	0.902833	1.86973
Chd7	NM_001081417	0.000991	8.63081	5.67269	4.77021	1.86928	0.902479	1.86928
Gm4302	NM_001166634	0.003836	6.01979	5.61427	4.71287	1.86788	0.901403	1.86788
Cyp2u1	NM_027816	0.001769	7.41127	6.09717	5.19662	1.86679	0.900556	1.86679
Ppp1r14b	NM_008889	5.96E-05	17.7195	10.0048	9.10486	1.86599	0.899942	1.86599
Xpo1	NM_134014	0.001309	8.02344	9.87075	8.972	1.86445	0.898752	1.86445
Fam111a	BC038020	0.005924	5.34043	8.07642	7.179	1.86273	0.897422	1.86273
Rnf114	NM_030743	0.000602	9.82217	9.50887	8.613	1.86074	0.895877	1.86074
Syne2	NM_001005510	0.001273	8.08247	5.90373	5.00881	1.8595	0.894916	1.8595
Gm12185	NM_001045540	0.001485	7.76112	4.11303	3.21913	1.8582	0.893903	1.8582
Klc4	NM_029091	0.001184	8.23746	7.0834	6.19053	1.85688	0.892877	1.85688
Vps37b	NM_177876	0.002453	6.79255	6.82641	5.93367	1.8567	0.892737	1.8567
Nup88	NM_172394	0.000567	9.97845	9.0112	8.11878	1.85628	0.892415	1.85628
Hps3	NM_080634	0.001176	8.2536	7.7607	6.8686	1.85587	0.892099	1.85587
Nr1h4	NM_001163700	0.002967	6.45382	5.02915	4.13734	1.8555	0.891809	1.8555

Mreg	NM_001005423	0.002219	6.9773	6.35132	5.45992	1.85497	0.8914	1.85497
Mrpl1	NM_053158	0.005801	5.37151	7.8861	6.99496	1.85464	0.891141	1.85464
8-Sep	NM_033144	0.005554	5.43699	8.41128	7.52015	1.85463	0.89113	1.85463
H1f0	NM_008197	0.002163	7.0253	10.6524	9.76166	1.85406	0.890689	1.85406
Men1	NM_008583	0.000814	9.08442	8.91954	8.02938	1.85339	0.890164	1.85339
Gfm1	NM_138591	0.001166	8.27034	8.87836	7.98825	1.85332	0.890111	1.85332
Grwd1	NM_153419	0.002648	6.65451	7.78224	6.89236	1.85302	0.889881	1.85302
Kcnab2	NM_010598	0.004337	5.82111	6.31504	5.42534	1.85279	0.889702	1.85279
Rpap1	NM_177294	2.43E-06	39.5947	6.94757	6.0581	1.85249	0.889467	1.85249
Vac14	NM_146216	0.000825	9.05321	8.46246	7.57352	1.85182	0.88894	1.85182
	---	0.005558	5.4359	8.16713	7.27826	1.85172	0.888863	1.85172
	---	0.000409	10.8538	9.16334	8.27491	1.85116	0.888432	1.85116
Fhod1	NM_177699	2.69E-05	21.6621	7.33791	6.44995	1.85056	0.887961	1.85056
Vsig10	NM_001033311	0.005573	5.43188	6.30006	5.41222	1.85041	0.887845	1.85041
Tlk2	NM_001112705	0.006988	5.09896	7.87698	6.98917	1.85037	0.887813	1.85037
Dclre1a	NM_018831	0.001719	7.46784	8.01054	7.12307	1.84993	0.887469	1.84993
Mettl2	NM_172567	0.00204	7.13554	8.82795	7.94071	1.84964	0.887244	1.84964
Nbeal2	NM_183276	0.001675	7.51898	6.32429	5.43714	1.84952	0.88715	1.84952
Itgb3	NM_016780	0.003918	5.98529	8.24455	7.3578	1.84901	0.886756	1.84901
Cyth2	NM_011181	0.00028	11.9618	8.65953	7.77285	1.84892	0.886685	1.84892
Rest	NM_011263	0.001494	7.74906	8.62308	7.73662	1.84864	0.886463	1.84864
	---	0.000551	10.0528	9.30995	8.42369	1.84837	0.886257	1.84837
Znhit3	NM_001005223	1.30E-05	26.0019	7.24174	6.35628	1.84736	0.885462	1.84736
	---	0.006997	5.09711	10.4908	9.60558	1.84701	0.88519	1.84701
Scrib	NM_134089	0.000102	15.4489	7.96616	7.08099	1.84698	0.88517	1.84698
Cecr5	NM_144815	0.000402	10.9005	7.72781	6.84414	1.84506	0.883668	1.84506
Ssx2ip	NM_138744	0.000284	11.917	7.39817	6.51554	1.84373	0.882628	1.84373
A130049A11Rik	NR_030676	0.002531	6.73622	3.77103	2.8885	1.8436	0.882525	1.8436
Epb4.1l1	NM_013510	0.000975	8.66775	8.55778	7.67588	1.8428	0.881901	1.8428
Kctd10	NM_001159941	0.000366	11.1698	9.7427	8.86158	1.84181	0.881122	1.84181
Tnrc6b	NM_144812	8.72E-05	16.0933	8.64349	7.76337	1.84054	0.880126	1.84054

Spg11	NM_145531	0.002866	6.51481	7.34522	6.46524	1.84035	0.879981	1.84035
Hsf1	NM_008296	0.000241	12.4257	8.99115	8.11124	1.84027	0.879915	1.84027
Ppp1r14b	NM_008889	0.000135	14.3991	10.021	9.14119	1.84013	0.879809	1.84013
	---	8.02E-05	16.4369	11.8934	11.0144	1.83913	0.879021	1.83913
Apbb2	NM_009686	0.001529	7.70151	9.10344	8.22549	1.83777	0.877955	1.83777
Zfp608	NM_175751	0.003353	6.24409	7.65092	6.77332	1.83731	0.877592	1.83731
	---	0.000217	12.7636	10.8294	9.95199	1.83709	0.877421	1.83709
Tcf20	NM_001114140	0.001213	8.18562	9.16451	8.28709	1.83709	0.87742	1.83709
BC049349	NM_001164581	0.002213	6.98251	5.52309	4.64654	1.83598	0.876551	1.83598
Naa40	NM_027643	0.004605	5.72582	7.42967	6.55315	1.83595	0.876527	1.83595
Tspo	NM_009775	0.001818	7.35717	11.1722	10.2961	1.83547	0.876148	1.83547
Bcl2l13	NM_153516	0.003271	6.28592	8.2696	7.39383	1.83498	0.875768	1.83498
Nf2	NM_010898	0.000482	10.4047	8.33808	7.4624	1.83488	0.875686	1.83488
Ccdc91	NM_025911	0.001921	7.25074	8.57213	7.69688	1.83433	0.875256	1.83433
Tsc2	NM_011647	0.002115	7.06722	8.59517	7.72071	1.83333	0.874467	1.83333
Nfya	NM_001110832	9.35E-05	15.809	8.72194	7.84778	1.83294	0.874158	1.83294
Prpf8	NM_138659	0.000173	13.5302	10.5684	9.69455	1.83259	0.873884	1.83259
Myg1	NM_021713	5.74E-05	17.8917	8.16958	7.2962	1.83195	0.873377	1.83195
Senp1	NM_144851	0.000397	10.9336	7.99304	7.11967	1.83194	0.873371	1.83194
Tmbim1	NM_027154	9.67E-05	15.6771	10.5717	9.69848	1.83173	0.873207	1.83173
Slc9a2	NM_001033289	0.002062	7.11542	5.10676	4.23423	1.83087	0.87253	1.83087
	---	0.001467	7.78736	10.0055	9.13341	1.83026	0.872048	1.83026
5930434B04Rik	BC027257	0.005206	5.53542	5.89047	5.01884	1.82973	0.871628	1.82973
	---	0.003417	6.21206	9.29365	8.423	1.82849	0.87065	1.82849
Wbscr17	NM_145218	0.002503	6.75603	7.04942	6.17907	1.82811	0.87035	1.82811
Dennd5b	NM_177192	0.004494	5.76435	6.6566	5.78682	1.82738	0.869777	1.82738
Eif3m	NM_145380	0.005042	5.58454	8.74899	7.87927	1.82731	0.869721	1.82731
Eepd1	NM_026189	0.002622	6.67241	6.1762	5.30767	1.8258	0.868529	1.8258
	---	0.005326	5.5005	4.40053	3.53203	1.82577	0.868504	1.82577
Ece1	NM_199307	0.000593	9.86021	8.32718	7.45921	1.82508	0.867963	1.82508
	---	0.006621	5.17684	7.53024	6.66317	1.82395	0.867064	1.82395

Wdr47	NM_181400	0.00191	7.26125	7.44858	6.58234	1.82291	0.86624	1.82291
	---	0.000495	10.3316	10.7882	9.92205	1.82286	0.866203	1.82286
Mfge8	NM_008594	0.000269	12.0793	10.8609	9.99525	1.82211	0.865607	1.82211
Ppp2r1a	NM_016891	0.000134	14.4228	10.7685	9.90367	1.82111	0.864819	1.82111
Nub1	NM_016736	0.001075	8.45009	9.29304	8.42832	1.82099	0.86472	1.82099
	---	0.000652	9.62187	10.8565	9.9922	1.82052	0.864352	1.82052
Fam173b	BC030179	0.000935	8.76378	8.01222	7.14794	1.82043	0.86428	1.82043
Cdc27	NM_145436	0.000184	13.3128	9.64737	8.78312	1.82039	0.864245	1.82039
Abca3	NM_013855	0.001434	7.83336	7.75287	6.88897	1.81995	0.863901	1.81995
Slc25a30	NM_026232	0.001725	7.46079	8.6836	7.81992	1.81967	0.863681	1.81967
Cox15	NM_144874	0.005211	5.53409	7.93134	7.06797	1.81928	0.863366	1.81928
Vps13b	NM_177151	0.004864	5.64027	7.9075	7.04422	1.81918	0.863287	1.81918
Mus81	NM_027877	0.001148	8.30542	7.85638	6.99325	1.81897	0.863125	1.81897
Irak2	NM_172161	0.004425	5.78909	6.48586	5.62414	1.8172	0.86172	1.8172
Thoc5	NM_172438	0.00108	8.43921	8.89482	8.03316	1.81712	0.861656	1.81712
Grn	NM_008175	0.00014	14.268	11.5585	10.6973	1.81659	0.861229	1.81659
2310003L22Rik	NM_027093	0.002414	6.82211	5.56887	4.70764	1.81658	0.861228	1.81658
Prpf31	NM_027328	0.002966	6.45472	8.74631	7.88558	1.81597	0.860738	1.81597
Nr2f2	NM_009697	0.003048	6.40758	9.37533	8.51552	1.8148	0.859811	1.8148
	---	0.006717	5.15597	8.65332	7.79351	1.8148	0.859808	1.8148
Pafah1b1	NM_013625	2.87E-05	21.3043	9.59856	8.73884	1.81468	0.859715	1.81468
Laptm4b	NM_033521	0.000143	14.1916	10.7565	9.89717	1.81418	0.859317	1.81418
Fez2	NM_199448	0.000909	8.82819	8.29783	7.4401	1.81219	0.857735	1.81219
Ikzf2	NM_011770	0.003773	6.04716	6.5384	5.68116	1.81157	0.857238	1.81157
Ube3b	NM_054093	0.004028	5.94015	8.02868	7.17207	1.81078	0.856611	1.81078
Rfc1	NM_011258	0.001721	7.46554	8.595	7.7385	1.81065	0.856504	1.81065
Prdm16	NM_027504	0.006234	5.26474	6.1206	5.26418	1.81053	0.856412	1.81053
Gm4371	NR_028311	0.004631	5.71712	4.44324	3.58714	1.81013	0.856096	1.81013
	---	0.00197	7.2021	8.04276	7.18693	1.8098	0.855833	1.8098
	---	0.002966	6.45471	7.1324	6.2768	1.80951	0.855598	1.80951
Eftud2	NM_011431	0.000133	14.4718	9.25599	8.40058	1.80926	0.855402	1.80926

Pign	NM_013784	0.000652	9.62499	9.55295	8.69768	1.80909	0.855262	1.80909
Pigt	NM_133779	6.66E-05	17.2293	9.69534	8.84049	1.80857	0.854853	1.80857
Ttc1	NM_133795	0.000115	14.9897	8.12833	7.27426	1.80759	0.85407	1.80759
Aup1	NM_007517	8.25E-05	16.3227	10.4876	9.63366	1.80747	0.853969	1.80747
Man2b2	NM_008550	0.001344	7.96863	8.29645	7.444	1.80556	0.85245	1.80556
Kpna4	NM_008467	0.004491	5.76547	8.06196	7.21003	1.80492	0.851932	1.80492
Supt4h1	NM_009296	0.000379	11.0656	10.3407	9.48878	1.80485	0.851883	1.80485
Cyb5r3	NM_029787	0.000376	11.093	11.7975	10.9459	1.80451	0.851606	1.80451
Pex13	NM_023651	0.000105	15.3379	8.60902	7.75771	1.80414	0.851307	1.80414
Dnmbp	NM_028029	0.006712	5.15705	7.88563	7.03459	1.80379	0.851034	1.80379
Ndufs1	NM_001160038	0.000117	14.9353	10.0522	9.2012	1.80371	0.85097	1.80371
Naa15	NM_053089	0.005025	5.58978	9.18356	8.3329	1.80333	0.85066	1.80333
Cdk5rap1	NM_025876	0.001185	8.23602	6.61253	5.76307	1.80182	0.849457	1.80182
Pak4	NM_027470	0.002531	6.73622	7.24061	6.39194	1.80084	0.848674	1.80084
Kalrn	NM_177357	0.003716	6.07212	6.99908	6.15122	1.79983	0.847861	1.79983
Erlin1	NM_145502	0.000392	10.971	8.24428	7.39655	1.79966	0.847727	1.79966
Nbr1	NM_008676	0.003168	6.34061	10.5003	9.65275	1.79941	0.847527	1.79941
Snx27	NM_029721	0.005746	5.38582	8.2734	7.42604	1.7992	0.847354	1.7992
Wfs1	NM_011716	0.004774	5.66952	7.50974	6.66325	1.79813	0.846497	1.79813
Chchd7	NM_181391	0.000225	12.6465	8.44802	7.60191	1.79765	0.846111	1.79765
Nol4	NM_199024	0.001641	7.5599	5.68669	4.84202	1.79586	0.844671	1.79586
Mphosph9	NM_001081323	0.000895	8.86202	6.20667	5.36203	1.79582	0.844646	1.79582
U05342	NR_024093	0.000641	9.66491	7.69215	6.84786	1.79539	0.844294	1.79539
Tnfaip1	NM_009395	0.001543	7.68345	8.59245	7.74838	1.7951	0.844063	1.7951
Ap4b1	NM_026193	5.32E-06	32.5394	8.56487	7.72088	1.79501	0.843988	1.79501
Cstf2t	NM_031249	0.006492	5.20549	8.24152	7.39777	1.79471	0.843749	1.79471
Plrg1	NM_016784	3.19E-05	20.7413	10.6087	9.76534	1.79426	0.843386	1.79426
9130023H24Rik	NM_177001	0.000476	10.439	7.89196	7.04876	1.79402	0.8432	1.79402
Zfat	NM_001145888	0.002989	6.44119	5.92906	5.08698	1.79263	0.842081	1.79263
Nsf	NM_008740	0.000255	12.2533	9.09063	8.24899	1.79209	0.841641	1.79209
	---	0.000298	11.7727	11.1651	10.3236	1.79193	0.841513	1.79193

	---	0.006162	5.28207	11.1654	10.3244	1.79134	0.841041	1.79134
E430025E21Rik	NM_153548	0.000145	14.1382	10.4164	9.57544	1.7912	0.840924	1.7912
Nf1	NM_010897	0.005395	5.48091	8.76262	7.92254	1.79016	0.840087	1.79016
Efcab2	NM_026626	0.000977	8.66387	4.66088	3.82148	1.78931	0.839403	1.78931
Katnb1	NM_028805	0.000665	9.57561	7.93611	7.09704	1.7889	0.839076	1.7889
	---	0.00614	5.28716	6.98416	6.1459	1.78789	0.838256	1.78789
Tagap1	NM_147155	0.003244	6.3004	7.98839	7.15014	1.78787	0.838245	1.78787
Gm9938	ENSMUST00000067077	0.000514	10.2329	8.06874	7.23061	1.78773	0.838125	1.78773
Mapk11	NM_011161	0.004172	5.88321	6.67534	5.83723	1.78771	0.838109	1.78771
Pef1	NM_026441	0.000522	10.1922	8.94328	8.10589	1.78683	0.837399	1.78683
Chd7	NM_001081417	0.000439	10.6572	5.29903	4.4618	1.78662	0.837232	1.78662
Nup205	NM_027513	0.002371	6.85463	9.005	8.16991	1.78397	0.835089	1.78397
Ogfr	NM_031373	0.000272	12.0515	9.24436	8.4093	1.78394	0.835066	1.78394
Tcp1	NM_013686	0.001539	7.68896	11.148	10.3144	1.78212	0.833594	1.78212
Magi1	NM_001029850	0.000666	9.57062	7.35871	6.52516	1.78206	0.833546	1.78206
Pacsin2	NM_011862	0.001883	7.28949	9.56684	8.73331	1.78203	0.833522	1.78203
Usp32	NM_001029934	0.002964	6.45613	9.44991	8.61747	1.78069	0.83244	1.78069
Fxr1	NM_001113188	0.003478	6.18248	10.2617	9.42938	1.7805	0.832284	1.7805
Trim8	NM_053100	0.000106	15.3248	7.99599	7.16418	1.77992	0.831816	1.77992
Tbcd	NM_029878	0.000539	10.1073	9.17136	8.34068	1.77852	0.83068	1.77852
Acot8	NM_133240	0.003921	5.98404	6.78243	5.95187	1.77838	0.830562	1.77838
Vezt	NM_172538	0.001829	7.34549	7.53566	6.70576	1.77757	0.829903	1.77757
	---	3.39E-05	20.4256	8.93399	8.10444	1.77713	0.829547	1.77713
Rhbdl3	NM_139228	0.003304	6.26932	6.76365	5.93413	1.7771	0.829522	1.7771
Prkaa1	NM_001013367	0.000283	11.9249	10.462	9.63303	1.77646	0.829003	1.77646
Cog4	NM_133973	0.003096	6.38016	9.10219	8.27319	1.77645	0.829	1.77645
Stard3	NM_021547	0.003035	6.41486	8.71061	7.88192	1.77607	0.828687	1.77607
Rsf1	NM_001081267	0.000285	11.9082	8.6498	7.82122	1.77593	0.828577	1.77593
Eif2s3x	NM_012010	0.002059	7.11796	10.8574	10.0289	1.77586	0.828522	1.77586
Dusp27	NM_001033344	0.001443	7.82048	4.66372	3.83576	1.77517	0.827959	1.77517

Ap4e1	NM_175550	0.005592	5.42678	7.17514	6.3473	1.77504	0.827848	1.77504
Rfx7	NM_001033536	0.005377	5.48622	6.63149	5.80367	1.77501	0.82783	1.77501
	---	0.003928	5.98105	7.64626	6.81847	1.77497	0.827795	1.77497
Traf7	NM_001172113	0.00011	15.165	9.37258	8.54526	1.77438	0.827317	1.77438
Gm14403	NR_036450	0.002389	6.84127	9.48108	8.65409	1.77398	0.82699	1.77398
Mprip	NM_012027	0.001559	7.66321	9.62665	8.80018	1.77334	0.826468	1.77334
Myl6b	NM_172259	0.002545	6.72586	5.50951	4.68345	1.77284	0.826063	1.77284
Gins3	NM_030198	0.000682	9.51343	6.79228	5.96683	1.77209	0.82545	1.77209
	---	0.001279	8.07256	7.58228	6.75691	1.772	0.825377	1.772
Slc45a3	NM_145977	0.000373	11.1104	6.36531	5.54014	1.77174	0.825163	1.77174
Mrpl18	NM_026310	0.001043	8.51598	7.87153	7.04646	1.77162	0.825066	1.77162
Rptor	NM_028898	0.000274	12.0268	7.88917	7.06423	1.77147	0.824944	1.77147
Krt80	NM_028770	0.001246	8.12907	6.18546	5.36089	1.77101	0.82457	1.77101
	---	0.001048	8.50578	10.3023	9.47833	1.77033	0.824016	1.77033
Card10	NM_130859	0.006674	5.16512	6.6876	5.86372	1.77017	0.823888	1.77017
Pik3r2	NM_008841	0.006179	5.2779	8.55585	7.73217	1.76991	0.823677	1.76991
Arhgef17	NM_001081116	0.000777	9.19654	8.39903	7.57601	1.76911	0.823021	1.76911
Dhx38	NM_178380	1.77E-05	24.0507	7.77787	6.95505	1.76886	0.822823	1.76886
Dnajb4	NM_025926	0.001225	8.16547	10.0092	9.18645	1.76872	0.822704	1.76872
	---	0.002976	6.449	8.54646	7.72416	1.76822	0.822299	1.76822
Dgke	NM_019505	0.003886	5.99873	6.14808	5.32601	1.76794	0.82207	1.76794
Foxf1a	NM_010426	0.001752	7.43021	8.44804	7.62626	1.76758	0.821774	1.76758
Myo1c	NM_008659	0.000615	9.76989	9.65808	8.83652	1.76732	0.821567	1.76732
Dhx8	NM_144831	0.000749	9.2834	8.34238	7.52201	1.76586	0.820369	1.76586
Gpn1	NM_133756	0.00456	5.74145	6.77107	5.95131	1.76511	0.819759	1.76511
Bcl2l1	NM_009743	0.001301	8.03678	7.99759	7.1779	1.76502	0.819687	1.76502
Ccdc134	NM_172428	0.00407	5.9233	7.52553	6.70595	1.76489	0.819575	1.76489
Tinf2	NM_145705	0.003166	6.34195	7.14612	6.32659	1.76483	0.819528	1.76483
Zbtb46	NM_028125	0.003817	6.02806	6.87581	6.05643	1.76466	0.819386	1.76466
Eif4b	NM_145625	0.002962	6.45709	10.7878	9.96964	1.76318	0.818179	1.76318
Helq	NM_001081107	0.000439	10.6598	6.8854	6.0674	1.76296	0.817997	1.76296

	---	0.000312	11.6296	10.2268	9.40882	1.76293	0.817972	1.76293
Kat2a	NM_020004	0.001832	7.34185	7.96713	7.14935	1.76269	0.817778	1.76269
Efr3a	NM_133766	0.000921	8.79642	10.4383	9.6209	1.76218	0.81736	1.76218
Atpaf1	NM_181040	0.006836	5.13058	6.82473	6.00779	1.76166	0.816937	1.76166
	---	0.00244	6.8026	10.1808	9.36401	1.7615	0.816806	1.7615
Zxdc	NM_173002	0.006358	5.23593	7.86198	7.04575	1.76079	0.816224	1.76079
Lyz1	NM_013590	0.003	6.43462	4.83802	4.02198	1.76057	0.816047	1.76057
Accn2	NM_009597	0.001975	7.19719	5.83815	5.02277	1.75976	0.815377	1.75976
Capza2	NM_007604	1.74E-05	24.1503	11.0831	10.2688	1.7584	0.814265	1.7584
8430419L09Rik	NM_028982	0.002903	6.49201	7.11967	6.30665	1.75688	0.813017	1.75688
Hexdc	NM_001146073	0.005998	5.32188	6.46478	5.6518	1.75684	0.812979	1.75684
	---	0.005412	5.4763	6.79304	5.98009	1.7568	0.812951	1.7568
Ano8	NM_001164679	0.00302	6.42313	7.96755	7.15539	1.75584	0.812164	1.75584
1810037I17Rik	NM_024461	0.000534	10.1308	10.7548	9.94272	1.7558	0.812128	1.7558
Lman2l	NM_001013374	0.001325	7.99781	8.85472	8.04318	1.75508	0.811533	1.75508
Pex1	NM_027777	0.001212	8.18696	7.25446	6.44328	1.75465	0.811185	1.75465
Etv3	NM_001083318	0.000226	12.6344	8.14841	7.33736	1.75449	0.81105	1.75449
Ahcyl2	NM_021414	0.000998	8.61421	9.09902	8.28871	1.75359	0.810309	1.75359
1110014N23Rik	NM_001081041	0.000476	10.4357	8.20746	7.39723	1.75348	0.810225	1.75348
Arrb1	NM_177231	0.00098	8.65477	6.85124	6.04141	1.753	0.809822	1.753
Gm14403	NR_036450	0.003856	6.01135	6.82991	6.02067	1.7523	0.809246	1.7523
Gm14288	NM_001033123	0.003856	6.01135	6.82991	6.02067	1.7523	0.809246	1.7523
Lpin2	NM_001164885	0.001147	8.30642	8.01734	7.20871	1.75154	0.808626	1.75154
Inpp5k	NM_008916	0.001742	7.44087	9.23007	8.42193	1.75096	0.808143	1.75096
Mrps26	NM_207207	0.00208	7.09897	8.45181	7.64381	1.75077	0.807992	1.75077
Fam190a	NM_183310	0.001852	7.32096	5.43687	4.62891	1.75073	0.807955	1.75073
Snord104	NR_030703	0.004877	5.63633	9.11389	8.30601	1.75065	0.807887	1.75065
Fam178a	BC060679	0.006444	5.21636	8.67102	7.86361	1.75007	0.807414	1.75007
2210009G21Rik	NM_001038641	0.003276	6.28341	6.35095	5.54356	1.75004	0.80739	1.75004
Vsig10	NM_001033311	3.56E-05	20.1786	6.35364	5.54649	1.74975	0.80715	1.74975
Zfyve27	NM_177319	0.000198	13.0642	8.23724	7.43106	1.74857	0.806178	1.74857

Ddx56	NM_026538	0.001062	8.4768	8.86081	8.05463	1.74857	0.806175	1.74857
Sorbs3	NM_011366	1.87E-05	23.7442	7.44888	6.64422	1.74673	0.80466	1.74673
	---	0.000609	9.79591	5.38618	4.58206	1.74608	0.80412	1.74608
Polr2b	NM_153798	0.000207	12.9179	10.3616	9.55756	1.74602	0.804072	1.74602
Slc25a35	NM_028048	0.00205	7.12634	5.40034	4.59639	1.74588	0.803952	1.74588
	---	0.002757	6.58299	9.29402	8.49012	1.74581	0.8039	1.74581
Atg2a	NM_194348	0.000235	12.502	7.79693	6.99324	1.74556	0.803686	1.74556
Mynn	NM_030557	0.006169	5.28035	7.96459	7.16119	1.74521	0.803399	1.74521
Ubxn8	NM_178648	0.004166	5.88559	7.4006	6.59731	1.74508	0.803289	1.74508
Pold2	NM_008894	0.004913	5.62466	8.09179	7.28864	1.7449	0.803146	1.7449
Btrc	NM_001037758	1.69E-05	24.3565	8.54643	7.74374	1.74434	0.802682	1.74434
Mms19	NM_028152	0.000293	11.8243	8.597	7.79458	1.74402	0.802419	1.74402
Tpi1	NM_009415	0.000523	10.1889	12.0689	11.2669	1.74352	0.802004	1.74352
Nfat5	NM_133957	0.003454	6.19398	9.04683	8.24511	1.74318	0.801722	1.74318
Nav2	NM_175272	0.000139	14.287	6.0419	5.24041	1.7429	0.801489	1.7429
Gm1943	NR_002928	0.002839	6.53103	9.53242	8.73122	1.74255	0.801197	1.74255
Fam160a2	NM_199009	0.000308	11.6678	7.73338	6.93226	1.74245	0.801121	1.74245
Rab3gap1	NM_178690	0.000184	13.3044	9.10387	8.30328	1.74181	0.800587	1.74181
Pdik1l	NM_146156	0.000174	13.5129	6.79518	5.99477	1.7416	0.800412	1.7416
2310016M24Rik	NM_183256	0.000728	9.35187	8.64418	7.84507	1.74002	0.799104	1.74002
Dhh	NM_007857	0.000271	12.0608	5.63497	4.83592	1.73996	0.79905	1.73996
Comt1	NM_001111062	0.000757	9.257	9.04869	8.2498	1.73976	0.798887	1.73976
Snrnp200	NM_177214	0.000195	13.1126	9.75365	8.95492	1.73957	0.798728	1.73957
Tmx2	NM_025868	0.002326	6.89015	9.91502	9.11672	1.73905	0.798302	1.73905
Lin7c	NM_011699	0.001398	7.88676	9.62258	8.82449	1.7388	0.798091	1.7388
Alg10b	NM_001033441	0.000428	10.7261	9.83836	9.04041	1.73862	0.797944	1.73862
	---	0.0034	6.22043	7.75628	6.95928	1.73748	0.796995	1.73748
Dnajc10	NM_024181	0.003217	6.31436	10.4035	9.6066	1.73733	0.796872	1.73733
Clcn6	NM_011929	0.000488	10.3733	7.56889	6.77211	1.73722	0.796783	1.73722
Ndrg3	NM_180956	0.001435	7.83211	9.29241	8.4958	1.73701	0.796602	1.73701
Prkcdbp	NM_028444	0.000184	13.3059	10.3677	9.5712	1.73686	0.796486	1.73686

Brf2	NM_025686	0.006754	5.1479	6.56509	5.76948	1.7358	0.795604	1.7358
Slc25a33	NM_027460	0.000848	8.98727	5.53307	4.73752	1.73574	0.795551	1.73574
Lrch4	NM_146164	0.006072	5.30362	6.92061	6.12567	1.73501	0.794942	1.73501
Trem2	NM_031254	0.000642	9.66113	6.73366	5.93878	1.73493	0.794878	1.73493
Tmem231	NM_001033321	0.001346	7.96507	8.02477	7.22995	1.73485	0.794815	1.73485
Crnkl1	NM_025820	6.21E-05	17.537	8.42207	7.62827	1.73364	0.793805	1.73364
Ccdc102a	NM_001033533	0.001627	7.57663	7.68101	6.88725	1.73358	0.79375	1.73358
Speg	NM_007463	0.003195	6.32622	6.64253	5.84914	1.73315	0.793393	1.73315
Cep78	NM_198019	0.004619	5.72116	6.67296	5.8798	1.73287	0.79316	1.73287
2310008H09Rik	NM_023197	7.29E-05	16.8397	7.28089	6.48786	1.73271	0.793032	1.73271
Blvra	NM_026678	6.44E-05	17.3736	8.74448	7.95147	1.73268	0.793006	1.73268
Dcaf15	NM_172502	0.000658	9.60147	6.80246	6.01001	1.73201	0.792451	1.73201
Psme4	NM_134013	5.20E-05	18.3376	9.38636	8.59432	1.73152	0.792042	1.73152
Azin1	NM_001102458	0.006561	5.19004	10.2748	9.48327	1.73086	0.79149	1.73086
Srp68	NM_146032	0.000874	8.91721	10.309	9.51803	1.7302	0.790935	1.7302
Poldip3	NM_178627	0.000703	9.43609	10.539	9.7485	1.72968	0.790504	1.72968
Uba3	NM_011666	0.005561	5.43499	10.3545	9.56414	1.72953	0.790377	1.72953
Cyp39a1	NM_018887	0.0003	11.7466	6.77909	5.9888	1.72943	0.790297	1.72943
Zfml	NM_008717	0.005684	5.40205	9.04032	8.25043	1.72895	0.789895	1.72895
Stip1	NM_016737	0.001011	8.586	9.95018	9.16062	1.72855	0.789561	1.72855
Gcdh	NM_008097	0.001337	7.97886	8.15865	7.36921	1.7284	0.789438	1.7284
2310043J07Rik	NM_027158	0.005984	5.32532	5.66321	4.87383	1.72833	0.789381	1.72833
Zc3h6	NM_178404	0.004902	5.62829	6.67482	5.88548	1.72829	0.789343	1.72829
Tmx2	NM_025868	0.001797	7.37988	9.93069	9.14145	1.72816	0.789236	1.72816
Pgam1	NM_023418	0.004852	5.64414	10.6388	9.84966	1.728	0.789107	1.728
Pgam1	NM_023418	0.004852	5.64414	10.6388	9.84966	1.728	0.789107	1.728
Hs3st3a1	NM_178870	0.001895	7.27646	5.74223	4.95346	1.7276	0.788768	1.7276
Immp2l	NM_053122	0.006138	5.28766	5.51916	4.73076	1.72716	0.788398	1.72716
Zfp710	NM_175433	0.002049	7.12732	6.2026	5.41444	1.72688	0.788165	1.72688
Peo1	NM_153796	0.001077	8.44513	8.01346	7.22552	1.72661	0.787939	1.72661
Prpf38b	NM_025845	4.94E-05	18.578	9.48226	8.69527	1.72546	0.786982	1.72546

Cnst	NM_146105	0.000491	10.3541	6.8622	6.07545	1.72518	0.786745	1.72518
Far1	NM_026143	8.64E-05	16.1307	9.44169	8.65509	1.725	0.7866	1.725
Imp4	NM_178601	0.000261	12.1751	8.55741	7.7715	1.72419	0.785915	1.72419
Chd7	NM_001081417	0.001259	8.10585	5.94477	5.15895	1.72407	0.785818	1.72407
	---	0.001926	7.24527	8.82831	8.04258	1.72396	0.785724	1.72396
Fbf1	NM_172571	0.005279	5.51428	7.3249	6.53929	1.72383	0.785617	1.72383
Tmod3	NM_016963	0.002049	7.12732	9.98013	9.19482	1.72346	0.785308	1.72346
Rhot2	NM_145999	0.001251	8.12056	6.97376	6.18994	1.72168	0.783818	1.72168
Fam69a	NM_026062	0.003337	6.25216	9.25368	8.46996	1.72156	0.783718	1.72156
0610007P22Rik	NM_026676	0.001017	8.57312	9.15707	8.37394	1.72086	0.783126	1.72086
Stxbp3a	NM_011504	0.003314	6.2637	9.1129	8.32998	1.72061	0.782916	1.72061
Hs6st1	NM_015818	0.007064	5.08341	8.14101	7.35922	1.71926	0.781789	1.71926
Med13	NM_001080931	0.001397	7.88792	9.55229	8.77092	1.71877	0.781375	1.71877
Tra2a	ENSMUST0000010141<br>7	0.002637	6.66241	8.5504	7.76914	1.71863	0.781262	1.71863
Cog1	NM_013581	0.001704	7.48481	7.88973	7.10865	1.71842	0.78108	1.71842
Dip2b	NM_001159361	0.004881	5.63506	8.2642	7.48354	1.71792	0.780666	1.71792
Zfp207	NM_001130169	0.000546	10.074	9.42922	8.64918	1.71719	0.780046	1.71719
Hus1	NM_008316	0.006149	5.28517	7.00684	6.22742	1.71645	0.779424	1.71645
Gm6194	NR_033512	0.004643	5.71306	6.58364	5.8043	1.71635	0.779342	1.71635
2410089E03Rik	NM_001162906	0.00315	6.3508	7.65749	6.87846	1.71598	0.779031	1.71598
Srsf2ip	NM_028148	0.000543	10.0893	9.54876	8.76978	1.71591	0.778976	1.71591
1810013D10Rik	NM_001145433	0.001315	8.01378	7.07455	6.29581	1.71563	0.778739	1.71563
Otud7b	NM_001025613	0.000251	12.2965	8.07145	7.29281	1.71551	0.77864	1.71551
	---	0.00395	5.97179	9.12879	8.35034	1.71529	0.778454	1.71529
	---	0.003095	6.38063	9.61054	8.83232	1.71502	0.778228	1.71502
	---	0.001574	7.64381	9.04464	8.26688	1.71448	0.777768	1.71448
Ltv1	NM_181470	0.005816	5.36774	7.94617	7.16866	1.71416	0.777501	1.71416
Decr2	NM_011933	0.000548	10.0634	9.51361	8.73687	1.71325	0.776739	1.71325
Puf60	NM_028364	0.000182	13.3442	10.6622	9.8855	1.71322	0.776709	1.71322
Kptn	NM_133727	0.002459	6.78847	6.92125	6.14454	1.71321	0.776705	1.71321

Tspan17	NM_028841	0.000989	8.63428	7.40572	6.62915	1.71305	0.776567	1.71305
Retsat	NM_026159	0.000441	10.6432	7.96577	7.18958	1.71261	0.776197	1.71261
Arhgap23	NM_021493	0.001643	7.55706	8.11408	7.33813	1.71232	0.775953	1.71232
Rhot1	NM_001163354	0.000665	9.57367	8.65754	7.88197	1.71186	0.775564	1.71186
Nucks1	NM_175294	0.003012	6.4279	9.73166	8.95676	1.71107	0.774899	1.71107
Cdipt	NM_026638	0.000603	9.82056	9.60953	8.83475	1.71092	0.774776	1.71092
Atl2	NM_019717	0.000427	10.7366	9.26663	8.49208	1.71066	0.774553	1.71066
Tmem183a	NM_020588	0.000925	8.78809	8.31643	7.54196	1.71057	0.774477	1.71057
Slc25a32	NM_172402	0.00025	12.3174	8.93733	8.16359	1.7097	0.773743	1.7097
Ccdc61	NM_001033314	2.18E-06	40.6698	6.70147	5.92778	1.70964	0.773695	1.70964
Taok1	NM_144825	0.000347	11.3235	9.20717	8.43375	1.70932	0.773419	1.70932
Gm14326	NM_001190302	0.000913	8.81604	9.72564	8.9532	1.70816	0.772439	1.70816
Ywhae	NM_009536	8.02E-05	16.438	10.7908	10.0185	1.70789	0.772213	1.70789
Mrpl9	NM_030116	0.005066	5.57737	8.12807	7.35588	1.70786	0.772188	1.70786
Smarcal1	NM_018817	0.006082	5.30137	8.18712	7.41541	1.7073	0.771718	1.7073
Smyd5	NM_144918	0.005393	5.48156	8.09009	7.31868	1.70693	0.771404	1.70693
Jmjd4	NM_178659	0.000996	8.61828	6.9348	6.16365	1.70662	0.771142	1.70662
	---	0.006773	5.14394	8.04079	7.27094	1.70509	0.76985	1.70509
Wbscr27	NM_024479	0.006151	5.28467	7.1516	6.38275	1.70391	0.768847	1.70391
	---	0.005712	5.39482	10.0611	9.29267	1.7034	0.768413	1.7034
Ktn1	NM_008477	0.000557	10.0236	8.46897	7.70063	1.7033	0.768336	1.7033
Ube2z	NM_172300	6.31E-05	17.4633	9.23053	8.46239	1.70307	0.768139	1.70307
Zbtb7c	NM_145356	0.003702	6.07835	6.72786	5.96012	1.70259	0.767734	1.70259
Zc3h3	NM_172121	2.34E-05	22.4197	7.10811	6.34044	1.70253	0.767677	1.70253
	---	0.003488	6.17763	10.5479	9.78032	1.70244	0.767603	1.70244
1200014J11Rik	NM_025818	0.001078	8.44266	8.75552	7.98796	1.7024	0.767568	1.7024
Rhbdf1	NM_010117	0.001393	7.89338	8.61962	7.85242	1.70196	0.767197	1.70196
Lactb	NM_030717	0.005974	5.32773	6.56581	5.79903	1.70148	0.766789	1.70148
Suclg1	NM_019879	0.003437	6.20236	8.97167	8.20504	1.70129	0.76663	1.70129
Skil	NM_011386	0.003735	6.06387	9.67864	8.91317	1.69992	0.76547	1.69992
1110002N22Rik	NM_183275	0.001565	7.65445	6.20469	5.43926	1.69987	0.765426	1.69987

Tmem116	NM_001161627	0.000487	10.3779	5.28741	4.52204	1.69981	0.765371	1.69981
Camta1	NM_001081557	0.000541	10.0989	4.93717	4.17239	1.69911	0.764782	1.69911
Arhgef19	NM_172520	0.00217	7.019	6.45272	5.68832	1.69866	0.764396	1.69866
Ppp1ca	NM_031868	0.000204	12.9753	10.8599	10.0967	1.69725	0.763201	1.69725
C1rl	NM_181344	0.003128	6.36286	7.50487	6.74182	1.69708	0.763051	1.69708
Ap2a1	NM_007458	0.000455	10.5592	8.85331	8.09045	1.69685	0.762861	1.69685
Rfc3	NM_027009	0.003516	6.16395	7.07809	6.31528	1.69679	0.762809	1.69679
Traf3ip1	NM_028718	0.002785	6.56496	6.92709	6.16444	1.69661	0.762656	1.69661
Arhgef18	NM_133962	0.000714	9.39897	7.71018	6.94772	1.69638	0.762461	1.69638
Ncaph2	NM_001115132	4.38E-05	19.1512	9.0739	8.3116	1.6962	0.762302	1.6962
2310044H10Rik	NM_197991	0.002589	6.69505	9.08735	8.32511	1.69612	0.762237	1.69612
Sars2	NM_023637	0.001062	8.47531	6.545	5.78295	1.6959	0.76205	1.6959
Dgat1	NM_010046	0.005641	5.41364	8.4704	7.70896	1.69519	0.761448	1.69519
Agpat3	NM_053014	0.003133	6.35991	8.18048	7.4191	1.69512	0.761388	1.69512
Smg6	NM_001002764	0.005217	5.53224	8.83533	8.07405	1.69499	0.76128	1.69499
Smarcb1	NM_011418	3.02E-05	21.0411	9.79743	9.03688	1.69414	0.760549	1.69414
	---	0.000219	12.7408	11.15	10.3895	1.69409	0.760512	1.69409
Ahsa2	NM_172391	0.001025	8.55582	9.19597	8.43677	1.69255	0.759196	1.69255
Gatsl3	NM_028022	0.003453	6.19449	7.62404	6.86537	1.69193	0.758666	1.69193
Rock2	NM_009072	0.001464	7.7903	9.12793	8.36945	1.69171	0.758486	1.69171
Arhgef2	NM_008487	0.002225	6.97207	9.32022	8.56221	1.69117	0.758019	1.69117
Ddx27	NM_153065	0.001192	8.22355	8.59318	7.83519	1.69113	0.757989	1.69113
Ppp6r1	NM_172894	0.001449	7.81191	8.72893	7.97115	1.69089	0.757783	1.69089
Trmt6	NM_175113	0.002796	6.55845	6.98312	6.2257	1.69046	0.75742	1.69046
Rbm16	NM_134123	0.000679	9.52398	8.74144	7.98424	1.69021	0.757201	1.69021
	---	0.002014	7.1601	10.4459	9.68905	1.68981	0.756859	1.68981
Ing3	NM_023626	0.005482	5.45688	7.63256	6.87583	1.68965	0.756723	1.68965
Camkk1	NM_018883	0.00356	6.14358	6.55449	5.79922	1.68795	0.755274	1.68795
Exosc1	NM_025644	0.000158	13.8386	9.53242	8.77762	1.68739	0.754797	1.68739
	---	1.35E-05	25.7375	10.8188	10.064	1.68734	0.754749	1.68734
Pop4	NM_025390	0.000204	12.9668	7.35845	6.6038	1.68723	0.754653	1.68723

Ufsp1	NM_027356	0.002761	6.58042	6.11084	5.35623	1.68717	0.754607	1.68717
Mknk2	NM_021462	0.002157	7.03053	8.49638	7.74194	1.68698	0.75444	1.68698
Fam38b	NM_001039485	0.000699	9.4511	6.4491	5.69494	1.68665	0.75416	1.68665
5-Mar	NM_027314	0.001325	7.99876	9.69309	8.93902	1.68654	0.754068	1.68654
Gnao1	NM_010308	0.004983	5.6029	7.24666	6.49331	1.68571	0.753355	1.68571
Eif3c	NM_146200	0.000197	13.0857	10.728	9.97483	1.68554	0.75321	1.68554
Ap3s2	NM_009682	0.001963	7.20846	6.85334	6.10215	1.68317	0.751184	1.68317
Plekhb2	NM_145516	0.002956	6.4608	9.16246	8.41132	1.68313	0.751147	1.68313
Tmem161b	NM_175187	0.001803	7.3735	7.59431	6.8433	1.68297	0.75101	1.68297
Atp13a3	NM_001128096	0.00017	13.5942	9.5975	8.84688	1.68252	0.750621	1.68252
Rabggta	NM_019519	0.000902	8.84465	7.46497	6.71552	1.68115	0.749453	1.68115
Mtmr3	NM_028860	0.00048	10.416	9.21437	8.46517	1.68087	0.749204	1.68087
Narfl	NM_026238	0.001126	8.34731	7.19153	6.44245	1.68072	0.749081	1.68072
	---	0.006363	5.23475	7.3938	6.64478	1.68065	0.749019	1.68065
Ccndbp1	NM_010761	0.001243	8.13418	7.99758	7.24888	1.68028	0.748698	1.68028
Zc3h7b	NM_001081016	0.001478	7.7709	8.20057	7.45189	1.68026	0.748683	1.68026
Rrm2b	NM_199476	0.005509	5.44929	7.79656	7.04818	1.67991	0.748382	1.67991
Zfp329	NM_026046	0.00348	6.18152	7.12353	6.3754	1.67962	0.748135	1.67962
Ddx18	NM_025860	0.000331	11.4599	10.0755	9.32783	1.6791	0.747688	1.6791
Rhd	NM_011270	0.006346	5.2387	4.97822	4.2319	1.6775	0.746314	1.6775
Psmf1	NM_212446	0.000654	9.61481	8.24308	7.49728	1.67691	0.745806	1.67691
Map3k11	NM_022012	0.000337	11.4039	7.47031	6.72509	1.67623	0.745223	1.67623
Ppil4	NM_026141	0.000239	12.4514	8.7367	7.99154	1.67616	0.745156	1.67616
Chd7	NM_001081417	0.004374	5.80751	5.05444	4.30953	1.67587	0.744912	1.67587
Stxbp2	NM_011503	0.000599	9.83871	7.83108	7.08628	1.67574	0.7448	1.67574
Mov10	NM_001163440	0.002692	6.62535	8.06138	7.31729	1.67491	0.744087	1.67491
Mark2	NM_007928	0.000517	10.2162	7.91516	7.17118	1.67479	0.743976	1.67479
Dok1	NM_010070	9.82E-05	15.617	8.90765	8.16387	1.67456	0.743783	1.67456
Nop10	NM_025403	0.001647	7.55292	9.44733	8.70376	1.67432	0.743576	1.67432
Lmf1	NM_029624	0.005685	5.40192	8.76449	8.02098	1.67425	0.743514	1.67425
	---	0.001153	8.2954	8.76534	8.02215	1.67387	0.743188	1.67387

4930528A17Rik	NR_028384	0.001242	8.135	5.03876	4.29607	1.67329	0.742685	1.67329
Il17rc	NM_134159	0.00306	6.40028	8.65037	7.908	1.67292	0.742365	1.67292
E330009J07Rik	NM_175528	0.000169	13.6069	6.47978	5.7375	1.67282	0.742285	1.67282
Btaf1	NM_001080706	0.00149	7.75536	9.10226	8.3601	1.67269	0.742166	1.67269
Axin1	NM_009733	0.000659	9.59799	7.4141	6.67209	1.6725	0.742004	1.6725
Ctnna1	NM_009818	0.000121	14.8124	10.4559	9.7142	1.67212	0.741677	1.67212
Rtn1	NM_153457	0.003406	6.21762	5.80944	5.06786	1.672	0.741575	1.672
Bop1	NM_013481	0.000333	11.4432	8.92441	8.18314	1.67165	0.741277	1.67165
4922501C03Rik	NM_199316	0.000509	10.2595	6.11171	5.37053	1.67155	0.741187	1.67155
Rtcd1	NM_025517	0.003083	6.38741	8.84354	8.10249	1.67139	0.74105	1.67139
Snord16a	NR_028548	0.000575	9.94013	9.61994	8.87913	1.67112	0.740813	1.67112
Med31	NM_026068	0.00188	7.29207	8.0204	7.27961	1.67109	0.740787	1.67109
Katnal2	NM_027721	0.000108	15.2429	4.94832	4.20773	1.67087	0.740596	1.67087
Agfg2	NM_178162	0.001061	8.47844	7.75019	7.00998	1.67042	0.740213	1.67042
Prkag1	NM_016781	0.00157	7.64856	10.3421	9.602	1.67026	0.740076	1.67026
Luc7l3	NM_026313	0.000567	9.97734	10.1314	9.39175	1.66973	0.739617	1.66973
Zswim1	NM_028028	0.00278	6.56815	6.52588	5.78701	1.66887	0.738869	1.66887
Rictor	NM_030168	0.001533	7.69682	9.13222	8.39346	1.66875	0.738765	1.66875
Zfyve20	NM_030081	0.004445	5.78195	8.27345	7.53488	1.66853	0.738575	1.66853
Sez6l2	NM_144926	0.000555	10.0344	5.34558	4.60709	1.66844	0.738497	1.66844
Prmt10	NM_001081240	0.004212	5.86803	8.01151	7.27392	1.66738	0.737583	1.66738
Exosc2	NM_144886	0.003348	6.24683	8.11549	7.37855	1.66664	0.736939	1.66664
Shprh	NM_172937	0.000838	9.01589	8.17244	7.43614	1.6659	0.736302	1.6659
Lrrc14b	NM_001033042	0.000101	15.5233	6.06418	5.3281	1.66563	0.736071	1.66563
Larp4	NM_001024526	0.001326	7.99589	6.99842	6.26249	1.66548	0.735938	1.66548
Xab2	NM_026156	2.17E-05	22.8511	8.74687	8.01102	1.66538	0.735852	1.66538
Frmd8	NM_026169	0.001298	8.04204	7.82082	7.08521	1.66511	0.735617	1.66511
Adam10	NM_007399	0.000427	10.7352	10.4102	9.67476	1.66496	0.73549	1.66496
Sqrdl	NM_021507	0.004945	5.6147	8.15598	7.42116	1.66419	0.73482	1.66419
Mis12	NM_025993	0.002442	6.80124	8.04176	7.3071	1.66401	0.734661	1.66401
Ranbp6	NM_177721	0.001811	7.36512	8.02529	7.29109	1.66348	0.734202	1.66348

Rg9mtd3	NM_027266	0.001473	7.77897	6.93391	6.20038	1.6627	0.733529	1.6627
Kcmf1	NM_019715	0.001685	7.50734	10.1165	9.3834	1.66225	0.733138	1.66225
Bcar1	NM_009954	0.003794	6.03794	7.58017	6.84743	1.66179	0.732738	1.66179
Dnajc7	NM_019795	0.002178	7.01177	9.77187	9.03915	1.66177	0.732721	1.66177
Cacna1b	NM_001042528	0.003979	5.95998	5.66053	4.92818	1.66134	0.732349	1.66134
Fmnl2	NM_172409	0.000376	11.0891	8.35412	7.62181	1.6613	0.732314	1.6613
Kpnb1	NM_008379	0.000472	10.4612	10.2651	9.53308	1.66101	0.732062	1.66101
	---	0.00681	5.13595	9.78201	9.05021	1.66071	0.731805	1.66071
Sepx1	NM_013759	0.002457	6.78971	8.93859	8.20742	1.65998	0.731162	1.65998
Prpsap1	NM_026364	0.000622	9.74096	9.54569	8.81466	1.65981	0.731022	1.65981
Cyc1	NM_025567	0.003106	6.37491	9.97057	9.23973	1.65961	0.730844	1.65961
Slc12a6	NM_133649	0.002503	6.75621	8.18129	7.45062	1.65941	0.730671	1.65941
Srpk2	NM_009274	0.006299	5.24953	9.31769	8.58736	1.65901	0.730323	1.65901
Nfatc2ip	NM_010900	0.005821	5.36648	6.56822	5.83821	1.65865	0.730007	1.65865
Rnf135	NM_028019	0.002759	6.5817	6.75289	6.02296	1.65856	0.729929	1.65856
Usp37	NM_176972	0.000226	12.6294	6.93729	6.20834	1.65744	0.728956	1.65744
Asb8	NM_001170711	0.001796	7.38108	8.83323	8.10434	1.65736	0.728888	1.65736
Psmd11	NM_178616	1.06E-06	48.7977	10.0744	9.346	1.65675	0.728353	1.65675
Mrps7	NM_025305	0.000531	10.1469	8.75718	8.02912	1.65642	0.728065	1.65642
Anapc1	NM_008569	0.001289	8.05669	10.0058	9.27772	1.65639	0.72804	1.65639
Tmem93	NM_025318	0.001025	8.55518	8.05299	7.32527	1.65601	0.727715	1.65601
Eif3m	NM_145380	0.000203	12.9843	9.8676	9.13995	1.65594	0.727647	1.65594
Slc35b4	NM_021435	0.001631	7.57146	9.32493	8.59754	1.65564	0.727386	1.65564
Cds2	NM_138651	0.001281	8.06925	8.75596	8.02908	1.65505	0.726876	1.65505
Larp7	NM_138593	0.005406	5.47799	7.53704	6.81037	1.65482	0.726673	1.65482
Zfp568	NM_001167872	0.002988	6.44169	6.711	5.98454	1.65457	0.726458	1.65457
Ptk2	NM_007982	0.000665	9.57577	8.84456	8.11909	1.65344	0.725472	1.65344
Afmid	NM_027827	0.006012	5.31847	5.80251	5.07763	1.65276	0.724876	1.65276
Akap9	NM_194462	0.001267	8.09361	7.94351	7.2198	1.65143	0.723712	1.65143
Nras	NM_010937	0.000407	10.8672	8.2855	7.5625	1.65061	0.722995	1.65061
Lpcat3	NM_145130	0.000835	9.02561	8.79736	8.07446	1.6505	0.722902	1.6505

9130404D08Rik	NM_001167939	0.001621	7.58408	9.21146	8.48878	1.65025	0.722687	1.65025
Phka1	NM_008832	0.004404	5.79651	6.99069	6.26833	1.64988	0.722363	1.64988
Anxa1	NM_010730	0.001729	7.45634	12.2923	11.5703	1.64954	0.722064	1.64954
Ints1	NM_026748	0.000359	11.2213	7.21364	6.49165	1.64946	0.721992	1.64946
Fuk	NM_172283	0.003618	6.11642	7.40494	6.68394	1.64833	0.721001	1.64833
Rbm14	NM_019869	0.007078	5.08048	7.70598	6.98515	1.64813	0.720829	1.64813
Ipo8	NM_001081113	0.002827	6.5386	9.13083	8.41012	1.648	0.720712	1.648
Als2	NM_028717	0.003973	5.96241	7.13509	6.41444	1.64792	0.720645	1.64792
Rorb	NM_001043354	0.005122	5.56051	5.4266	4.70641	1.6474	0.720188	1.6474
Mphosph6	NM_026758	0.007066	5.08301	7.89673	7.17691	1.64697	0.719813	1.64697
Eif1ad	NM_027236	0.002352	6.86932	8.42444	7.70468	1.64691	0.71976	1.64691
Zbtb7b	NM_009565	0.000131	14.5112	7.78457	7.06581	1.64577	0.71876	1.64577
Mkrn2	NM_023290	0.004477	5.77052	8.16581	7.44733	1.64544	0.718473	1.64544
	---	0.001146	8.30819	10.2367	9.51847	1.64511	0.718187	1.64511
Ndufb8	NM_026061	0.004648	5.71128	7.97828	7.26072	1.64439	0.717555	1.64439
Thoc6	NM_001008425	0.001296	8.04553	7.94225	7.22506	1.64398	0.717192	1.64398
Gltp	NM_019821	0.002218	6.97819	7.97633	7.26061	1.64229	0.715711	1.64229
Pkn2	NM_178654	0.000957	8.70905	9.50457	8.78927	1.64182	0.715297	1.64182
Wbscr16	NM_033572	0.003047	6.40795	4.92849	4.21342	1.64156	0.715067	1.64156
Atg9a	NM_001003917	0.000138	14.3234	7.80003	7.08562	1.64081	0.714409	1.64081
	---	0.003055	6.40353	9.83367	9.11957	1.64046	0.714101	1.64046
Abhd8	NM_022419	0.00523	5.52834	7.87275	7.15869	1.64042	0.714063	1.64042
Wfikkn1	NM_001100454	0.002002	7.17133	6.8654	6.15147	1.64026	0.713926	1.64026
0610007P22Rik	NM_026676	6.12E-05	17.6002	7.68539	6.97146	1.64026	0.713923	1.64026
	---	0.001661	7.53499	7.82268	7.10893	1.64006	0.713749	1.64006
Drg1	NM_007879	0.004615	5.72262	9.31307	8.59951	1.63985	0.713563	1.63985
Actn4	NM_021895	0.001566	7.65368	11.2937	10.5803	1.63966	0.713398	1.63966
Ehd2	NM_153068	0.001562	7.65938	10.5582	9.84548	1.63886	0.712696	1.63886
Apbb1ip	NM_019456	0.002223	6.97401	6.34091	5.62824	1.63884	0.712677	1.63884
Unc13b	NM_001081413	7.09E-05	16.9584	5.69303	4.98073	1.63842	0.712304	1.63842
Wdr19	NM_153391	0.005805	5.37067	8.05017	7.33827	1.63795	0.711892	1.63795

Bend4	NM_001164806	0.001201	8.20704	6.10511	5.39341	1.63773	0.7117	1.63773
Tnrc6a	NM_144925	0.002004	7.16964	8.70172	7.99121	1.63638	0.710509	1.63638
	---	0.002715	6.61025	11.2325	10.5222	1.63609	0.710249	1.63609
Commd5	NM_025536	0.000477	10.4321	7.91022	7.20021	1.63582	0.710015	1.63582
Mrpl35	NM_025430	0.002359	6.86422	8.70764	7.99779	1.63563	0.709847	1.63563
4930519F16Rik	BC107248	0.001362	7.93985	3.71759	3.0079	1.63545	0.709689	1.63545
Pcbp2	NM_001103165	0.000553	10.0422	12.202	11.4925	1.63527	0.709526	1.63527
Tprn	NM_175286	0.000172	13.5523	5.97239	5.26288	1.63525	0.709507	1.63525
Cnot4	NM_016877	0.000381	11.0498	7.97428	7.26496	1.63503	0.709316	1.63503
Pus3	NM_023292	0.001074	8.45112	7.36737	6.65836	1.63468	0.709007	1.63468
Zfp120	NM_181266	0.003594	6.12733	5.33857	4.62964	1.6346	0.708936	1.6346
Ide	NM_031156	0.000504	10.2875	9.69403	8.98545	1.63419	0.708578	1.63419
Pdzk1ip1	NM_001164557	0.00599	5.32383	5.38156	4.6732	1.63395	0.708362	1.63395
Gm527	BC099503	0.004767	5.67167	6.96226	6.25434	1.63345	0.707921	1.63345
2310061I04Rik	NM_001033630	4.11E-06	34.7016	7.60986	6.90291	1.63234	0.706943	1.63234
Ap2b1	NM_001035854	4.17E-05	19.3897	9.83803	9.1311	1.63233	0.706932	1.63233
Tnpo3	NM_177296	0.000251	12.2982	10.2195	9.51321	1.63156	0.706256	1.63156
Dyrk1b	NM_001037957	0.00528	5.51379	7.35598	6.6498	1.63148	0.70618	1.63148
Mrpl28	NM_024227	0.000689	9.48531	7.81675	7.11138	1.63057	0.705374	1.63057
Pms1	NM_153556	0.005324	5.50106	6.30466	5.59981	1.62998	0.704858	1.62998
Stxbp3a	NM_011504	0.002687	6.62885	9.2948	8.59019	1.6297	0.704607	1.6297
Trappc10	NM_001081055	0.000216	12.7832	8.47237	7.76781	1.62966	0.704568	1.62966
Erc2	NM_177814	0.00331	6.26619	5.49074	4.78619	1.62963	0.704547	1.62963
Wdr1	NM_011715	0.000616	9.7664	11.0452	10.3408	1.62943	0.704367	1.62943
Sun2	NM_194342	0.006188	5.27584	8.7401	8.03672	1.62832	0.703384	1.62832
	---	0.002885	6.50325	7.55071	6.84777	1.62782	0.702939	1.62782
Dlgap1	NM_177639	0.000915	8.8127	4.93816	4.23556	1.62744	0.702602	1.62744
Nup62	NM_053074	0.002071	7.1071	8.84512	8.14267	1.62727	0.70245	1.62727
Ywhaz	NM_011740	0.001109	8.38144	10.5631	9.86071	1.62722	0.702414	1.62722
Lass2	NM_029789	0.000341	11.3693	10.435	9.73303	1.62674	0.701987	1.62674
Rnu1b1	NR_004412	0.002725	6.60382	7.17456	6.47295	1.62632	0.701612	1.62632

Rnu1b1	NR_004412	0.002725	6.60382	7.17456	6.47295	1.62632	0.701612	1.62632
Rnu1b1	NR_004412	0.002725	6.60382	7.17456	6.47295	1.62632	0.701612	1.62632
Rnu1b1	NR_004412	0.002725	6.60382	7.17456	6.47295	1.62632	0.701612	1.62632
Rnu1b1	NR_004412	0.002725	6.60382	7.17456	6.47295	1.62632	0.701612	1.62632
Fiz1	NM_011813	0.000248	12.3353	7.61664	6.91512	1.62623	0.701528	1.62623
Qk	NM_021881	0.000402	10.904	9.0921	8.39141	1.62529	0.700699	1.62529
Cops7b	NM_172974	0.000184	13.3212	7.46368	6.76308	1.62518	0.700602	1.62518
Hnrnph1	NM_021510	0.000958	8.70678	11.4434	10.7428	1.62516	0.700583	1.62516
Inpp5b	NM_008385	0.000641	9.66518	7.79886	7.09833	1.62511	0.700536	1.62511
Capza1	NM_009797	0.000294	11.8173	10.8326	10.1331	1.62386	0.699429	1.62386
Chd7	NM_001081417	0.005624	5.41817	6.64274	5.94354	1.62361	0.699202	1.62361
Mtif2	NM_133767	0.006797	5.13875	8.29327	7.59408	1.6236	0.699198	1.6236
Rint1	NM_177323	0.000903	8.84269	7.82719	7.12802	1.62358	0.699176	1.62358
Tuba1b	NM_011654	0.003604	6.12271	12.2628	11.5639	1.62325	0.698882	1.62325
Zfp740	NM_153194	0.001536	7.69288	8.54164	7.84373	1.62215	0.697907	1.62215
Hcn1	NM_010408	0.005735	5.38879	4.88701	4.18939	1.62183	0.69762	1.62183
Ubash3b	NM_176860	0.00443	5.78737	4.90956	4.21229	1.62143	0.697267	1.62143
Phf5a	NM_026737	0.001344	7.96842	9.82985	9.13265	1.62135	0.697195	1.62135
Tm6sf1	NM_145375	0.000277	11.9967	5.36698	4.66981	1.62132	0.697172	1.62132
Scamp5	NM_020270	0.006061	5.30641	7.08229	6.38513	1.62131	0.697156	1.62131
	---	0.00068	9.51745	10.5799	9.88309	1.62089	0.696787	1.62089
Mpv17l2	NM_183170	0.003139	6.35644	8.82143	8.12501	1.62047	0.696413	1.62047
Trpc1	NM_011643	0.003709	6.07528	6.99956	6.30325	1.62036	0.696315	1.62036
Trp53bp1	NM_013735	0.005495	5.45313	8.2002	7.50394	1.6203	0.696257	1.6203
Paip2b	NM_146169	0.003307	6.26759	8.56535	7.86925	1.62012	0.696103	1.62012
Pex3	NM_019961	0.001893	7.27839	9.01417	8.31837	1.61978	0.695797	1.61978
Ubxn4	NM_026390	0.003045	6.40917	10.0151	9.31935	1.61975	0.695775	1.61975
Usp40	NM_001198573	0.001702	7.48659	8.43207	7.73632	1.61973	0.695754	1.61973
Klhl11	NM_172565	0.000645	9.65178	8.79483	8.09928	1.6195	0.695549	1.6195
Micall1	NM_177461	0.006967	5.1033	7.24137	6.5461	1.61919	0.695269	1.61919
Mkln1	NM_013791	0.000124	14.7048	9.31235	8.6174	1.61883	0.694954	1.61883

1300001I01Rik	BC072573	0.004812	5.65715	8.82144	8.12654	1.61878	0.694905	1.61878
	---	0.005234	5.52723	9.79671	9.10186	1.61872	0.694855	1.61872
Bckdk	NM_009739	0.000286	11.8953	9.58301	8.88828	1.61858	0.694731	1.61858
Vps33a	NM_029929	0.000716	9.39201	8.80102	8.10669	1.61813	0.694327	1.61813
Bard1	NM_007525	0.006387	5.22933	4.8675	4.17331	1.61798	0.694193	1.61798
2810422O20Rik	BC050143	0.003989	5.95566	5.87747	5.18394	1.61723	0.693528	1.61723
Pdxp	NM_020271	0.003189	6.32937	5.95027	5.25726	1.61665	0.693005	1.61665
	---	0.006874	5.12257	10.6696	9.97674	1.61643	0.692815	1.61643
Phf21b	NR_030731	8.64E-05	16.1285	5.67194	4.97914	1.61642	0.692799	1.61642
Gtf3c3	NM_001033194	0.001298	8.04121	8.44694	7.75427	1.61627	0.692671	1.61627
Sart1	AF129931	0.002194	6.99878	6.14182	5.45044	1.61483	0.691379	1.61483
Cse1l	NM_023565	0.002778	6.56949	10.298	9.60664	1.61481	0.691368	1.61481
Mtmr4	NM_133215	0.004838	5.64862	8.58081	7.89067	1.61344	0.690138	1.61344
	---	0.00086	8.95577	9.03281	8.34289	1.61319	0.689913	1.61319
Sh3bp4	NM_133816	0.000992	8.62918	7.61898	6.9291	1.61315	0.689884	1.61315
Maf1	NM_001164607	0.004721	5.68707	9.74405	9.05453	1.61275	0.689522	1.61275
Cnot3	NM_146176	0.003405	6.21829	8.09468	7.406	1.61181	0.688679	1.61181
	---	0.000366	11.1639	11.0271	10.3387	1.61148	0.688383	1.61148
2310044G17Rik	NM_173735	0.000428	10.7281	7.34042	6.65204	1.61147	0.688378	1.61147
Ppp1r7	NM_023200	0.004467	5.77397	9.06465	8.3763	1.61144	0.688353	1.61144
Nsmce2	NM_026746	0.006259	5.25889	8.93764	8.24974	1.61094	0.687901	1.61094
2400003C14Rik	NM_028018	5.22E-05	18.3241	10.0219	9.33404	1.6109	0.687868	1.6109
Kazald1	NM_178929	0.000191	13.1837	6.83813	6.15035	1.61081	0.687786	1.61081
Wdr54	NM_023790	0.005425	5.47256	6.66538	5.97793	1.61044	0.687451	1.61044
Gatad2b	NM_139304	0.000344	11.3505	9.0134	8.32607	1.6103	0.687331	1.6103
Fbxo25	NM_025785	0.000242	12.4128	8.75173	8.06459	1.61009	0.687144	1.61009
Slc6a13	NM_144512	0.004898	5.62955	5.4546	4.76748	1.61007	0.687128	1.61007
	---	0.000287	11.8887	10.097	9.41009	1.60981	0.686893	1.60981
Otub1	NM_134150	0.003695	6.08133	9.67821	8.9914	1.60973	0.686817	1.60973
Rnu12	NR_004432	0.002016	7.15769	7.02918	6.34299	1.60903	0.686193	1.60903
Jazf1	NM_001168277	0.004699	5.69431	6.62429	5.93831	1.6088	0.685982	1.6088

Pcbp3	NM_021568	0.002875	6.50904	7.34224	6.65652	1.60851	0.685721	1.60851
Gm10589	ENSMUST00000098096	0.003875	6.00329	6.37091	5.68649	1.60705	0.684414	1.60705
Apol10b	NM_177820	0.000273	12.0336	5.37572	4.69137	1.60697	0.684342	1.60697
Exoc7	NM_016857	0.000957	8.71001	8.20066	7.51668	1.60656	0.683979	1.60656
Trmt112	NM_001166370	0.006141	5.28708	7.67295	6.98941	1.60608	0.683544	1.60608
Maea	NM_021500	0.000242	12.4085	10.3982	9.71477	1.60594	0.683416	1.60594
Noc4l	NM_153570	0.005166	5.54713	8.04234	7.35903	1.60582	0.68331	1.60582
Cnot1	NM_153164	0.002194	6.99862	10.2088	9.5263	1.60494	0.68252	1.60494
Stk16	NM_011494	0.000209	12.8885	8.63661	7.95413	1.6049	0.682487	1.6049
Caskin2	NM_080643	0.001064	8.47125	6.74078	6.05881	1.60433	0.681975	1.60433
Elk3	NM_013508	0.000588	9.8855	9.18528	8.50332	1.60432	0.681962	1.60432
C2cd3	NM_001017985	0.001548	7.67724	7.86116	7.17923	1.60429	0.681935	1.60429
Hr	NM_021877	0.002143	7.04249	5.66454	4.98282	1.60405	0.681719	1.60405
Pwwp2b	NM_001098636	0.006329	5.24271	7.03866	6.35698	1.60401	0.681687	1.60401
Stard10	NM_019990	0.004791	5.66382	7.41307	6.7315	1.60389	0.681571	1.60389
Rad51l3	NM_011235	0.000435	10.6835	7.704	7.02272	1.60357	0.681287	1.60357
Rdm1	NM_025654	0.000815	9.08125	6.81194	6.13071	1.60351	0.68123	1.60351
Ywhaz	NM_011740	5.64E-05	17.9677	12.237	11.5563	1.6029	0.680682	1.6029
Mad2l2	NM_027985	0.005207	5.53509	6.57249	5.89197	1.60271	0.680513	1.60271
Nup85	NM_001002929	0.002527	6.73883	7.91606	7.23561	1.60264	0.680447	1.60264
Tubgcp3	NM_198031	0.000352	11.282	8.30953	7.6292	1.6025	0.680327	1.6025
Ralgps1	NM_175211	0.000804	9.11281	6.31217	5.63192	1.60241	0.680244	1.60241
	---	0.000767	9.22624	4.35271	3.67346	1.6013	0.679246	1.6013
1700013F07Rik	BC116223	0.001255	8.1127	5.16817	4.48898	1.60124	0.679193	1.60124
Dhrs13	NM_183286	0.002197	6.99571	6.02523	5.34627	1.60098	0.678958	1.60098
Ankfy1	NM_009671	0.00309	6.38393	9.00961	8.33161	1.59992	0.678	1.59992
1500032L24Rik	BC092006	0.002709	6.61447	10.8463	10.1686	1.59966	0.677764	1.59966
Smcr8	NM_001085440	0.003251	6.29658	7.79821	7.12048	1.59963	0.677734	1.59963
Rnf213	ENSMUST00000131035	0.004634	5.71601	6.30969	5.63202	1.59956	0.677674	1.59956
	---	0.001621	7.58398	10.0594	9.38181	1.59943	0.677556	1.59943

Ndufa4	NM_010886	0.00237	6.85559	10.9859	10.3084	1.59929	0.677434	1.59929
Brd7	NM_012047	0.004433	5.78629	8.31089	7.63468	1.59793	0.676205	1.59793
Pphln1	NM_146062	0.000884	8.89134	9.44625	8.77005	1.59793	0.676204	1.59793
Psip1	NM_133948	0.0029	6.49379	7.22124	6.54503	1.59793	0.676204	1.59793
Ralgapb	NM_177658	0.002725	6.60365	8.93118	8.2552	1.59768	0.675976	1.59768
Psmd12	NM_025894	0.000259	12.2036	9.57054	8.89554	1.59659	0.674997	1.59659
Dcaf7	NM_027946	0.003332	6.25454	9.67259	8.99775	1.59642	0.674842	1.59642
	---	7.07E-05	16.9714	7.28311	6.60834	1.59635	0.674777	1.59635
6720401G13Rik	NR_015505	0.001835	7.3393	7.79551	7.12084	1.59623	0.67467	1.59623
Rps6kb2	NM_021485	0.000244	12.3907	7.63054	6.95627	1.59579	0.67427	1.59579
Smpd3	NM_021491	0.000317	11.5881	5.41029	4.7361	1.5957	0.67419	1.5957
Pafah2	NM_133880	0.002494	6.76259	7.55332	6.87925	1.59556	0.674064	1.59556
BC017647	NM_145430	0.001906	7.26571	7.47326	6.79954	1.59518	0.673724	1.59518
Th1l	NM_020580	0.000158	13.8307	7.26944	6.59591	1.59498	0.673534	1.59498
Zfp1	NM_001037665	0.003361	6.24033	7.37388	6.70096	1.5943	0.672923	1.5943
Ppp1r11	NM_029632	0.001306	8.02814	7.88765	7.21485	1.59416	0.6728	1.59416
Pcm1	NM_023662	0.002354	6.86819	9.29552	8.6231	1.59374	0.672417	1.59374
Mavs	NM_144888	0.002416	6.82068	8.66547	7.99356	1.59318	0.67191	1.59318
1500011H22Rik	BC019498	0.000529	10.1569	8.56517	7.89344	1.59298	0.671729	1.59298
5430437P03Rik	NM_026636	0.000405	10.8841	9.93171	9.26001	1.59295	0.671698	1.59295
Kdm5a	NM_145997	0.002032	7.14272	9.2715	8.60007	1.59266	0.671436	1.59266
Tcp11l1	NM_177190	0.001391	7.89654	6.9292	6.25794	1.59246	0.671257	1.59246
Alpk1	NM_027808	0.005531	5.4434	7.54879	6.87768	1.5923	0.671112	1.5923
Pppde2	NM_134095	0.003871	6.0049	8.34013	7.66967	1.59158	0.670462	1.59158
Osbp2	NM_152818	0.002126	7.05732	6.44529	5.77547	1.59088	0.669822	1.59088
Ubxn1	NM_146093	0.002163	7.02469	9.18373	8.51398	1.5908	0.669753	1.5908
Mrps5	NM_029963	0.006349	5.23788	7.23252	6.56381	1.58965	0.668711	1.58965
Pcyox1l	NM_172832	0.000744	9.30006	7.16699	6.49838	1.58954	0.668606	1.58954
Ikbkb	NM_010546	0.000921	8.79678	8.23455	7.56663	1.58878	0.667921	1.58878
Phf12	NM_174852	0.00076	9.2469	8.6401	7.97268	1.58823	0.667418	1.58823
D330012F22Rik	NM_178752	0.005308	5.50569	5.30589	4.6385	1.5882	0.667388	1.5882

Tada3	NM_133932	0.004003	5.95034	7.70276	7.03538	1.58819	0.667388	1.58819
Rnf40	NM_172281	0.000282	11.9419	8.25451	7.58716	1.58815	0.667343	1.58815
	---	0.007056	5.08511	10.6599	9.99303	1.58762	0.666868	1.58762
Ghdc	NM_031871	0.003842	6.01702	6.58521	5.91838	1.58758	0.666828	1.58758
Iqsec1	NM_001134384	0.000806	9.10845	7.91638	7.24998	1.58711	0.666402	1.58711
Xpo5	NM_028198	0.001034	8.53605	8.52838	7.86205	1.58703	0.666328	1.58703
Gm10615	ENSMUST0000009835 5	0.000335	11.422	4.60118	3.93488	1.58699	0.666297	1.58699
Ppp1r12b	NM_001081307	0.00249	6.76588	6.68522	6.01903	1.58687	0.666186	1.58687
D330041H03Rik	NR_033554	0.004247	5.85472	6.46994	5.80378	1.58685	0.666162	1.58685
	---	0.004788	5.66484	11.5708	10.9047	1.58677	0.666094	1.58677
Tesk2	NM_146151	0.0024	6.83266	6.76051	6.09517	1.58594	0.665341	1.58594
Iqgap1	NM_016721	0.001075	8.45008	9.75647	9.09128	1.58578	0.665194	1.58578
Tyk2	NM_018793	0.005132	5.55737	6.78276	6.11774	1.58559	0.665019	1.58559
Ddx20	NM_017397	0.004292	5.83788	7.78435	7.1199	1.58497	0.664453	1.58497
Strbp	NM_009261	0.000438	10.663	5.43535	4.77142	1.58439	0.66393	1.58439
Tsen54	NM_029557	0.000132	14.4737	7.80549	7.14215	1.58374	0.663339	1.58374
	---	0.001964	7.20755	8.40538	7.74208	1.5837	0.663301	1.5837
	---	0.000143	14.1973	5.24635	4.58329	1.58344	0.66306	1.58344
Med19	NM_025885	0.002191	7.0008	7.84615	7.18314	1.58339	0.663016	1.58339
Nr2c2ap	NM_001025586	0.006129	5.28996	7.74432	7.08134	1.58336	0.662986	1.58336
Disp2	NM_170593	0.003397	6.22207	5.13945	4.47679	1.583	0.662662	1.583
Ino80d	NM_001114609	0.001554	7.66929	7.70052	7.03856	1.58223	0.66196	1.58223
Letm1	NM_019694	0.0036	6.12457	7.45055	6.78869	1.58211	0.661851	1.58211
Cyhr1	NM_019396	0.000287	11.8869	8.64238	7.98149	1.58106	0.660888	1.58106
Tmem150a	NM_144916	2.39E-05	22.3002	8.73435	8.07382	1.58067	0.660533	1.58067
	---	0.002574	6.70546	4.38347	3.72293	1.58066	0.660531	1.58066
Vps52	NM_172620	0.000175	13.4905	7.711	7.0507	1.58041	0.660299	1.58041
Mrps18a	NM_026768	0.004808	5.6583	8.05026	7.39044	1.57988	0.659817	1.57988
Pds5b	NM_175310	0.003076	6.39166	8.4291	7.77046	1.57859	0.658634	1.57859
Cd3eap	NM_145822	0.000492	10.3495	6.9358	6.27744	1.57829	0.65836	1.57829

Sall1	NM_021390	0.003198	6.32488	5.73982	5.08146	1.57829	0.658358	1.57829
Srrm2	NM_175229	0.001232	8.15242	10.4003	9.74195	1.57827	0.658343	1.57827
Arid2	NM_175251	0.003008	6.42999	8.7297	8.0718	1.57779	0.657907	1.57779
Triobp	NM_001039156	0.002293	6.91651	7.32883	6.67112	1.57758	0.657709	1.57758
Vps35	NM_022997	0.002586	6.69749	10.3656	9.7086	1.57684	0.657039	1.57684
Ttc12	NM_172770	0.004857	5.64253	6.9628	6.30595	1.57664	0.656852	1.57664
Plekha5	NM_144920	0.000719	9.38059	7.65115	6.99447	1.57645	0.65668	1.57645
Arrb2	NM_145429	0.001509	7.72831	6.43403	5.77873	1.57495	0.655302	1.57495
Dhx36	NM_028136	0.000588	9.88539	9.23304	8.57805	1.5746	0.654989	1.5746
Pno1	NM_025443	0.001525	7.70727	8.20897	7.55402	1.57457	0.654955	1.57457
Obfc1	NM_175360	0.002838	6.53187	8.27087	7.61595	1.57453	0.654923	1.57453
Dppa2	NM_028615	0.000509	10.2576	4.91519	4.26052	1.57426	0.654669	1.57426
Tox4	NM_023434	0.000726	9.36023	8.27462	7.62007	1.57412	0.654549	1.57412
4632419I22Rik	BC067002	0.000486	10.3836	7.95498	7.30083	1.57369	0.654151	1.57369
Dbndd2	NM_001048227	0.00401	5.94748	5.37489	4.72133	1.57305	0.653565	1.57305
Kdm4b	NM_172132	0.002419	6.81847	8.19884	7.54587	1.5724	0.652966	1.5724
Nek8	NM_080849	0.000764	9.23583	6.62603	5.97317	1.57229	0.652869	1.57229
Exosc5	NM_138586	4.05E-05	19.5301	7.96961	7.31685	1.57217	0.652759	1.57217
Ncoa2	NM_008678	0.004803	5.66009	7.63807	6.98546	1.57201	0.652608	1.57201
Eri2	NM_027698	0.004578	5.7353	6.99945	6.34815	1.57058	0.651301	1.57058
Kctd9	NM_001111028	0.006461	5.2123	7.98247	7.3315	1.57021	0.650961	1.57021
Pdhx	NM_175094	0.002539	6.73026	7.37882	6.72829	1.56974	0.650528	1.56974
Gtf3c1	NM_207239	0.000508	10.2645	8.3463	7.69585	1.56966	0.65045	1.56966
Med23	NM_001166416	0.001218	8.17732	8.48496	7.8355	1.56858	0.649457	1.56858
Atp5g2	NM_026468	0.001173	8.25871	11.1402	10.4924	1.5668	0.647825	1.5668
	---	0.000707	9.42356	6.29033	5.64294	1.56633	0.647389	1.56633
Ehd1	NM_010119	0.004802	5.66016	8.83676	8.18983	1.56583	0.646926	1.56583
	---	0.001329	7.9912	11.7347	11.088	1.56556	0.646678	1.56556
Pmpca	NM_173180	0.001003	8.60356	9.2139	8.56729	1.56548	0.646608	1.56548
4931408A02Rik	AF358257	0.003676	6.09023	5.87352	5.22727	1.56509	0.64625	1.56509
Nop56	NM_024193	0.00591	5.34387	10.4049	9.75872	1.56504	0.646199	1.56504

	---	0.00704	5.08828	8.77686	8.13078	1.56492	0.646089	1.56492
Rbm27	NM_172626	0.006189	5.27541	8.22598	7.58052	1.56424	0.645465	1.56424
Asna1	NM_019652	0.000683	9.50648	9.66988	9.02455	1.56409	0.645327	1.56409
Eps15	NM_007943	4.74E-05	18.7746	9.1736	8.52847	1.56388	0.64513	1.56388
Atp5b	NM_016774	0.000652	9.62235	12.1286	11.4836	1.56379	0.64505	1.56379
Pigw	NM_027388	0.001486	7.76	6.69604	6.05114	1.56364	0.644905	1.56364
Nkiras2	NM_028024	0.006565	5.18915	6.86675	6.22236	1.56309	0.644396	1.56309
Lnpep	NM_172827	0.001016	8.57498	10.3647	9.72186	1.56135	0.642797	1.56135
Rtn3	NM_001003934	0.005418	5.47458	11.2686	10.6261	1.56104	0.642503	1.56104
Phb	NM_008831	0.000155	13.8982	10.0514	9.4091	1.56078	0.642263	1.56078
Kdm1a	NM_133872	0.000155	13.9107	9.29912	8.65705	1.56056	0.642067	1.56056
Mapk3	NM_011952	0.007069	5.08246	9.61694	8.97488	1.56056	0.642063	1.56056
Acer3	NM_025408	0.000279	11.9692	6.65053	6.00892	1.56007	0.64161	1.56007
Lmnb2	NM_010722	0.000728	9.35343	6.61895	5.97742	1.55998	0.641531	1.55998
Ulk3	NM_027895	0.000245	12.3823	6.40543	5.76413	1.55973	0.641297	1.55973
Gak	NM_153569	0.001569	7.65029	8.92423	8.28294	1.55972	0.641288	1.55972
Zfp687	NM_030074	0.004542	5.74772	7.37717	6.73596	1.55964	0.641217	1.55964
St3gal3	NM_009176	0.004109	5.90765	7.8578	7.21676	1.55945	0.641038	1.55945
Capza1	NM_009797	0.000453	10.5705	10.6516	10.0107	1.5593	0.640896	1.5593
Cdk12	NM_001109626	0.005025	5.58992	7.81503	7.17418	1.55925	0.640852	1.55925
Sfxn3	NM_053197	0.001512	7.72528	10.0375	9.39734	1.55853	0.640184	1.55853
Anxa4	NM_013471	0.002337	6.88169	10.307	9.66696	1.55834	0.640014	1.55834
Rell1	NM_145923	0.002395	6.83645	8.85596	8.21667	1.55756	0.639288	1.55756
Rnf185	NM_145355	0.003554	6.14631	8.23782	7.5988	1.55727	0.639022	1.55727
Eif2ak2	NM_011163	0.006054	5.30798	7.73121	7.0923	1.55715	0.638904	1.55715
2810407C02Rik	NM_001040396	0.000641	9.66742	11.0014	10.3626	1.55709	0.63885	1.55709
Tnk2	NM_016788	0.001928	7.2436	7.27919	6.64045	1.55697	0.638739	1.55697
Gli3	NM_008130	0.001468	7.78529	6.84412	6.20574	1.55659	0.638387	1.55659
1110006O24Rik	NR_027810	0.000478	10.4281	6.33325	5.69499	1.55645	0.638258	1.55645
Psme3	NM_011192	0.00065	9.6311	9.26617	8.62801	1.55635	0.638162	1.55635
Atf6b	NM_017406	0.000994	8.62339	8.23139	7.59338	1.55618	0.638011	1.55618

Tom1l2	NM_153080	0.000707	9.422	8.16727	7.5307	1.55462	0.636563	1.55462
Ftsj3	NM_025310	0.004649	5.71096	9.87356	9.23707	1.55455	0.636496	1.55455
Timm17a	NM_011590	0.000372	11.1201	10.1876	9.55126	1.55442	0.636372	1.55442
Idh3b	NM_130884	0.002011	7.16236	10.8516	10.2153	1.55434	0.636298	1.55434
	---	0.001787	7.39083	9.66715	9.03126	1.5539	0.635893	1.5539
1700081L11Rik	BC054752	2.03E-05	23.2464	8.69385	8.05834	1.55349	0.635515	1.55349
Dhx35	NM_145742	0.002343	6.8766	7.86592	7.23048	1.55341	0.63544	1.55341
Shoc2	NM_019658	0.001354	7.95329	9.58072	8.94533	1.55335	0.635383	1.55335
Zfp707	NM_001081065	0.003268	6.28759	6.91182	6.27689	1.55287	0.634933	1.55287
5830418K08Rik	NM_176976	0.006294	5.25076	6.20342	5.56851	1.55283	0.634902	1.55283
Nup160	NM_021512	0.00432	5.82745	8.7761	8.14142	1.5526	0.634683	1.5526
Larp4	NM_001024526	0.00101	8.58703	9.96743	9.3333	1.55201	0.634133	1.55201
Slc4a3	NM_009208	0.006023	5.31561	7.27409	6.64002	1.55194	0.634076	1.55194
	---	0.002112	7.06968	9.72886	9.09534	1.55134	0.633516	1.55134
H2-M3	NM_013819	0.002629	6.66761	6.602	5.96849	1.55134	0.633512	1.55134
Mff	NM_029409	0.001479	7.76975	9.43233	8.79891	1.55123	0.633415	1.55123
Syngr2	NM_009304	0.000763	9.23824	9.8419	9.20936	1.5503	0.632544	1.5503
Limk2	NM_010718	0.001457	7.80143	7.83084	7.19884	1.5497	0.631993	1.5497
Derl2	NM_033562	0.005682	5.40281	9.07778	8.44601	1.54946	0.631764	1.54946
Zmynd8	NM_027230	0.003685	6.08596	9.26077	8.6296	1.54882	0.631173	1.54882
Por	NM_008898	0.002762	6.57999	9.31371	8.68266	1.54869	0.631048	1.54869
Trabd	NM_026485	0.006981	5.10035	8.10643	7.47572	1.54833	0.630714	1.54833
Gmeb2	NM_198169	0.000968	8.68398	6.9342	6.30385	1.54794	0.630346	1.54794
B3gntl1	NM_178664	0.003759	6.0531	5.91449	5.28438	1.54768	0.630107	1.54768
Prmt2	NM_133182	0.003116	6.36947	8.83788	8.20779	1.54766	0.630092	1.54766
Ubl3	NM_011908	0.00655	5.19241	9.2587	8.62939	1.54682	0.629309	1.54682
Strn4	NM_133789	0.000202	13.0074	7.71849	7.08929	1.54671	0.629202	1.54671
Ssrp1	NM_182990	0.000994	8.62359	9.70808	9.07897	1.54661	0.629106	1.54661
Kctd15	NM_146188	0.00069	9.48316	7.56836	6.93954	1.5463	0.628825	1.5463
Hoxa7	NM_010455	0.001517	7.71781	7.1678	6.53935	1.5459	0.628448	1.5459
Rabl5	NM_026073	0.002726	6.603	7.94114	7.31372	1.5448	0.627419	1.5448

Zfp689	NM_175163	0.003278	6.28268	7.37691	6.74993	1.54433	0.626978	1.54433
Cnnm4	NM_033570	0.001225	8.16482	6.55839	5.9315	1.54423	0.626884	1.54423
Ubr1	NM_009461	0.005033	5.58748	8.52292	7.89618	1.54407	0.626742	1.54407
Camta1	NM_001081557	0.001319	8.00838	8.36525	7.7386	1.54397	0.626648	1.54397
Hip1r	NM_145070	0.00457	5.73801	7.05705	6.43068	1.54367	0.626365	1.54367
Wtap	NM_001113533	0.000463	10.511	10.2655	9.63916	1.54367	0.626362	1.54367
Gpi1	NM_008155	0.00421	5.86879	11.4661	10.8405	1.54284	0.625587	1.54284
	---	0.001178	8.24912	12.2398	11.6147	1.54237	0.625152	1.54237
Dph1	NM_144491	0.006589	5.18378	7.99544	7.37066	1.54198	0.624781	1.54198
Psmc3ip	NM_008949	0.003579	6.13431	5.64029	5.01554	1.54194	0.624747	1.54194
Dcun1d2	NM_001024504	0.006632	5.17429	7.87463	7.2501	1.54172	0.624537	1.54172
Ctdspl2	NM_212450	0.005725	5.39128	7.09895	6.47461	1.5415	0.624335	1.5415
Grlf1	NM_172739	0.001734	7.44977	7.94536	7.32132	1.54118	0.624037	1.54118
Ccdc86	NM_023731	8.86E-05	16.0281	7.6854	7.06142	1.54111	0.623972	1.54111
Myef2	NM_001162417	0.005787	5.3752	8.03606	7.41236	1.54082	0.623703	1.54082
Tubgcp2	NM_133755	0.005182	5.5426	7.87067	7.24709	1.5407	0.623581	1.5407
Recql5	NM_130454	6.51E-05	17.3288	6.91971	6.2964	1.5404	0.623309	1.5404
	---	0.00227	6.93489	11.1295	10.5063	1.54018	0.623102	1.54018
Trpt1	NM_153597	0.000387	11.0054	6.55029	5.92836	1.53894	0.621937	1.53894
	---	0.003397	6.22198	3.88968	3.26885	1.53776	0.620834	1.53776
Srsf2	NM_011358	0.006931	5.11059	10.5929	9.97203	1.53775	0.620825	1.53775
Atp6v0c	NM_009729	0.004804	5.65964	11.2996	10.6789	1.53755	0.620631	1.53755
Tuba1b	NM_011654	0.006437	5.21776	12.536	11.9154	1.5375	0.620588	1.5375
Btbd11	NM_028709	0.001461	7.79577	6.30967	5.68938	1.53718	0.620289	1.53718
2310067B10Rik	NM_028014	0.004948	5.61364	7.99522	7.37548	1.5366	0.619738	1.5366
Baiap2	NM_130862	0.000726	9.35819	8.83909	8.21973	1.53619	0.619359	1.53619
1600027N09Rik	NM_028479	0.001719	7.46787	6.20564	5.58642	1.53605	0.619223	1.53605
Dido1	NM_175551	0.000636	9.68431	7.85903	7.24015	1.53568	0.618876	1.53568
Ndufs8	NM_144870	0.000666	9.57126	8.3894	7.77087	1.53532	0.618535	1.53532
Ccng2	NM_007635	0.002865	6.51501	8.9466	8.3283	1.53506	0.618297	1.53506
Becn1	NM_019584	0.00265	6.65364	9.06604	8.4479	1.5349	0.618145	1.5349

Med25	NM_029365	0.002922	6.48047	8.1053	7.48817	1.53382	0.61713	1.53382
Tnks1bp1	NM_001081260	0.000125	14.6759	9.34595	8.72955	1.53305	0.616405	1.53305
Atrn	NM_009730	0.000991	8.63122	8.61486	7.99903	1.53244	0.615833	1.53244
Srgap2	NM_001081011	0.002413	6.82263	7.80301	7.18735	1.53225	0.615652	1.53225
Mogat2	NM_177448	0.001361	7.94188	5.30803	4.69273	1.53187	0.615292	1.53187
Appbp2	NM_025825	0.004893	5.63123	10.5137	9.89924	1.53096	0.614437	1.53096
Crtc1	NM_001004062	0.001434	7.83335	7.09343	6.4791	1.53084	0.614328	1.53084
Rab11fip5	NM_001003955	0.006872	5.12303	8.01338	7.39932	1.53056	0.614061	1.53056
Fam134c	BC016089	0.000946	8.73579	9.20584	8.59218	1.53014	0.613662	1.53014
Kdm5c	NM_013668	0.001493	7.75041	8.58142	7.96776	1.53013	0.613656	1.53013
	---	0.000335	11.4264	9.15881	8.54534	1.52993	0.613465	1.52993
	---	4.26E-05	19.2901	11.9604	11.3471	1.52968	0.613226	1.52968
Atp5g2	NM_026468	0.001623	7.5813	10.9729	10.3601	1.5292	0.612779	1.5292
Smurf1	NM_001038627	0.005143	5.5541	8.12929	7.51666	1.52905	0.612635	1.52905
	---	0.001572	7.64561	9.87877	9.26644	1.52873	0.612332	1.52873
Wdr59	NM_001170743	0.000754	9.26796	7.96137	7.34954	1.52819	0.611828	1.52819
Vrk2	NM_027260	0.005691	5.40023	7.32309	6.7114	1.52805	0.61169	1.52805
Wdfy1	NM_001111279	0.002061	7.116	8.05277	7.44189	1.52719	0.610881	1.52719
Ngrn	NR_028053	0.000192	13.165	7.60165	6.99099	1.52696	0.610661	1.52696
Fgd2	NM_001159538	0.00597	5.3287	6.38351	5.77336	1.52642	0.61015	1.52642
Rap2b	NM_028712	0.005823	5.36609	8.43326	7.82328	1.52623	0.609977	1.52623
Cant1	NM_029502	0.002937	6.4719	7.49402	6.88411	1.52616	0.609905	1.52616
Git2	NM_019834	0.002648	6.65467	7.98419	7.37476	1.52565	0.609426	1.52565
Mlkl	NM_029005	0.001185	8.23709	8.45303	7.84362	1.52563	0.609403	1.52563
Cd9	NM_007657	0.002722	6.60541	11.7469	11.1375	1.52563	0.609402	1.52563
Gpi1	NM_008155	0.001773	7.40677	11.111	10.5019	1.52536	0.609153	1.52536
Shroom4	NM_001040459	0.004804	5.65958	5.51228	4.90317	1.52532	0.609107	1.52532
1700021K19Rik	NM_172615	0.004094	5.91374	7.6092	7.00014	1.52527	0.609065	1.52527
	---	0.000565	9.98486	9.42171	8.81277	1.52514	0.608942	1.52514
Myo1d	NM_177390	0.006767	5.14513	8.05322	7.44458	1.52482	0.608635	1.52482
Dnajb1	NM_018808	0.004767	5.6717	8.38172	7.77339	1.5245	0.608333	1.5245

	---	0.000174	13.51	11.5715	10.9636	1.52402	0.607885	1.52402
Tomm22	NM_172609	0.000522	10.1908	8.24323	7.6361	1.52322	0.607124	1.52322
Mgea5	NM_023799	0.000881	8.89878	8.85401	8.24691	1.5232	0.607102	1.5232
D430042O09Rik	BC158019	0.00036	11.2139	7.05362	6.4467	1.523	0.606916	1.523
Taok3	NM_001081308	0.000207	12.9267	7.72287	7.11599	1.52297	0.606884	1.52297
Heatr6	NM_145432	5.99E-05	17.6993	8.16326	7.55673	1.52259	0.606525	1.52259
Acp6	NM_019800	0.000813	9.08766	8.13198	7.52705	1.5209	0.604927	1.5209
Necap1	NM_026267	0.002651	6.65271	8.984	8.37957	1.52038	0.604431	1.52038
Nup50	NM_016714	0.001257	8.10901	9.19285	8.58863	1.52015	0.60421	1.52015
Gls2	NM_001033264	0.005963	5.33061	5.45163	4.84745	1.52011	0.604179	1.52011
Gtf3c2	NM_027901	0.001618	7.58767	8.97421	8.37011	1.52004	0.604109	1.52004
Csnk2a1	NM_007788	7.26E-06	30.1006	10.2667	9.66274	1.51991	0.603988	1.51991
Sipa1l3	NM_001081028	5.05E-05	18.4778	7.36277	6.75921	1.51947	0.603566	1.51947
Chfr	NM_172717	0.005642	5.41325	8.03343	7.42988	1.51945	0.603548	1.51945
	---	0.001193	8.22147	7.107	6.50358	1.51932	0.603422	1.51932
Stub1	NM_019719	0.001171	8.26176	9.67338	9.07001	1.51926	0.603373	1.51926
Camk2b	NM_007595	0.003387	6.22685	5.92917	5.32581	1.51925	0.603358	1.51925
Suv420h2	NM_146177	0.002633	6.66473	7.65126	7.04792	1.51923	0.603337	1.51923
Csnk2a1	NM_007788	1.62E-05	24.6136	10.506	9.90308	1.51874	0.602879	1.51874
Srsf1	NM_173374	0.001247	8.1275	10.5441	9.94185	1.51811	0.602281	1.51811
Coq9	NM_026452	0.000694	9.47018	8.94349	8.34167	1.51764	0.601826	1.51764
Pmvk	NM_026784	0.004227	5.86204	6.29366	5.69256	1.51687	0.601094	1.51687
Caprin1	NM_016739	0.00023	12.582	10.8581	10.2571	1.51676	0.600996	1.51676
Gbgt1	NM_139197	0.00513	5.55809	4.73149	4.13067	1.51658	0.60082	1.51658
Zfp335	NM_199027	0.000445	10.6198	6.99532	6.39453	1.51655	0.60079	1.51655
Cmas	NM_009908	0.001276	8.07852	8.92624	8.3257	1.51628	0.600541	1.51628
Cyfip1	NM_001164661	0.000955	8.71485	9.22248	8.62206	1.51616	0.600421	1.51616
Abcf1	NM_013854	0.003905	5.99073	8.99615	8.39583	1.51606	0.600325	1.51606
Psmc5	NM_008950	0.004089	5.91554	9.85821	9.25843	1.51548	0.599779	1.51548
Cox8a	NM_007750	0.0011	8.39831	10.0698	9.47009	1.51538	0.599676	1.51538
	---	0.0009	8.84907	5.28151	4.68191	1.5153	0.5996	1.5153

Cideb	NM_009894	0.002801	6.5552	5.43207	4.83267	1.51509	0.599403	1.51509
Palm	NM_023128	0.006777	5.14306	7.59382	6.99449	1.51501	0.59933	1.51501
4632404H12Rik	ENSMUST00000038450	0.001515	7.72112	5.40195	4.80263	1.515	0.599318	1.515
	---	0.000361	11.208	9.44349	8.84554	1.51356	0.597943	1.51356
Kdm2a	NM_001001984	0.000169	13.5981	8.95946	8.36153	1.51354	0.597923	1.51354
Lmln	NM_172823	6.27E-05	17.4904	5.62355	5.02593	1.51322	0.597619	1.51322
	---	0.000687	9.49262	11.8381	11.2406	1.5131	0.597506	1.5131
Dstyk	NM_172516	0.001371	7.9271	8.62193	8.02508	1.51241	0.59685	1.51241
Anln	NM_028390	0.000499	10.312	5.1433	4.54656	1.5123	0.596741	1.5123
Nudt13	NM_026341	0.000491	10.3536	7.63165	7.03493	1.51227	0.596715	1.51227
Eif2c2	NM_153178	0.000545	10.0815	8.37805	7.78186	1.51172	0.596193	1.51172
Tuba1b	NM_011654	0.006583	5.18512	12.237	11.6409	1.51162	0.596098	1.51162
Ttc27	NM_152817	0.001857	7.31573	8.12861	7.53319	1.51091	0.595415	1.51091
Ccl17	NM_011332	0.000172	13.5389	5.19051	4.59569	1.51028	0.59482	1.51028
Atp11a	NM_015804	0.001308	8.02604	8.73489	8.14034	1.51	0.59455	1.51
Osbpl9	NM_133885	0.001032	8.53912	9.72445	9.13001	1.50989	0.594443	1.50989
	---	0.000259	12.1955	8.23311	7.63868	1.50988	0.59443	1.50988
Otud4	NM_001081164	0.005088	5.57073	9.22752	8.63326	1.5097	0.594267	1.5097
5730455P16Rik	NM_027472	0.00263	6.66686	8.37592	7.78242	1.50891	0.593503	1.50891
Atrn	NM_009730	0.001084	8.43028	8.29751	7.70449	1.5084	0.593017	1.5084
Prcc	NM_033573	0.001525	7.70724	8.61778	8.02483	1.50833	0.592955	1.50833
	---	0.000672	9.54683	10.6578	10.0651	1.50803	0.592663	1.50803
Phkb	NM_199446	0.002095	7.08491	9.0497	8.45726	1.5078	0.592441	1.5078
Bat1a	NM_019693	0.005555	5.43681	10.0437	9.45142	1.50766	0.592313	1.50766
Lars2	NM_153168	0.002245	6.95596	7.29929	6.70705	1.50758	0.592239	1.50758
Mrpl51	NM_025595	0.002665	6.64307	9.37049	8.7783	1.50753	0.592186	1.50753
Upf1	NM_001122829	0.000756	9.26152	8.36412	7.77236	1.50709	0.591762	1.50709
Sfmbt2	NM_177386	0.007005	5.09535	5.06876	4.4776	1.50646	0.591163	1.50646
Bbx	NM_027444	0.006326	5.24333	7.96178	7.37106	1.506	0.590719	1.506
Ppip5k2	NM_173760	0.005049	5.58239	8.41561	7.82502	1.50586	0.590592	1.50586

Mat2a	NM_145569	0.00021	12.8775	11.3805	10.7901	1.50571	0.590441	1.50571
	---	0.001134	8.33111	7.4808	6.89094	1.50511	0.589869	1.50511
Eml6	NM_146016	4.18E-05	19.3744	6.24501	5.65546	1.50478	0.58955	1.50478
Dot1l	NM_199322	0.003472	6.18511	6.96773	6.37823	1.50472	0.589499	1.50472
Calcoco2	NM_029755	0.003318	6.2618	4.74658	4.15722	1.50457	0.589354	1.50457
Tubb2b	NM_023716	0.003548	6.14923	6.9356	6.34648	1.50432	0.589113	1.50432
	---	0.002491	6.76513	8.4092	7.82025	1.50415	0.58895	1.50415
	---	0.004153	5.89052	7.76295	7.17402	1.50414	0.588935	1.50414
Leng8	NM_172736	0.000355	11.2524	8.08733	7.49862	1.5039	0.588712	1.5039
Pck2	NM_028994	0.00655	5.19252	8.63178	8.04334	1.50362	0.588441	1.50362
	---	0.002653	6.65134	11.5159	10.9275	1.50355	0.588374	1.50355
C030016D13Rik	NR_027987	0.004093	5.91422	4.41424	3.82718	1.50218	0.587058	1.50218
Gnaq	NM_008139	6.20E-05	17.5409	8.82298	8.23618	1.50192	0.586804	1.50192
Taco1	NM_027346	0.001249	8.12332	7.44594	6.85929	1.50176	0.58665	1.50176
	---	0.00642	5.2216	10.1395	9.55365	1.50095	0.585878	1.50095
Mtfr1	NM_026182	0.003162	6.34394	8.32474	7.73891	1.5009	0.585828	1.5009
Zbed4	NM_181412	0.001483	7.76514	6.58934	6.00356	1.50085	0.585784	1.50085
Hltf	NM_009210	0.002122	7.06105	7.92995	7.34477	1.50023	0.585181	1.50023
2310039H08Rik	NM_025966	0.002177	7.01323	7.95169	7.36694	1.49977	0.584744	1.49977
Spna2	NM_001076554	0.000777	9.19626	10.2202	9.63579	1.49941	0.584399	1.49941
Btbd10	NM_133700	0.006968	5.10306	7.37373	6.7903	1.49841	0.583434	1.49841
Cenpj	NM_001014996	0.003001	6.43403	6.22711	5.64423	1.49783	0.582872	1.49783
Fblim1	NM_133754	0.004966	5.60824	6.78647	6.2043	1.49709	0.58216	1.49709
Wwp2	NM_025830	0.00279	6.56203	7.71464	7.13316	1.49638	0.581477	1.49638
Shkbp1	NM_138676	0.001669	7.52631	7.04613	6.46489	1.49614	0.581243	1.49614
Glrp1	NM_008132	0.000998	8.61495	4.71913	4.13807	1.49595	0.581059	1.49595
Tmem188	NM_029074	0.000264	12.1369	9.28382	8.70365	1.49503	0.580173	1.49503
Atp6v0c	NM_009729	0.002043	7.13274	11.1684	10.5885	1.49477	0.579928	1.49477
Psmd3	NM_009439	0.000352	11.2784	8.48951	7.90962	1.49474	0.579895	1.49474
Phrf1	NM_001081118	0.001531	7.69987	8.26048	7.6807	1.49462	0.579775	1.49462
	---	0.001804	7.37267	4.66384	4.08414	1.49454	0.579698	1.49454

Ccdc47	NM_026009	9.30E-05	15.8339	10.028	9.44858	1.49423	0.579403	1.49423
Riok2	NM_025934	0.003378	6.23155	9.09922	8.52003	1.49401	0.579193	1.49401
N4bp2l2	NM_201369	0.001656	7.54123	8.27275	7.69397	1.49359	0.578782	1.49359
Fer1l6	ENSMUST0000003708 6	0.003282	6.2802	4.54273	3.96446	1.49305	0.578265	1.49305
Brsk1	NM_001003920	0.002169	7.01998	7.33751	6.75961	1.49267	0.577899	1.49267
Trim24	NM_145076	0.001053	8.49407	7.88253	7.30479	1.4925	0.577734	1.4925
Ube3a	NM_011668	0.00564	5.41383	8.9763	8.39917	1.49188	0.577128	1.49188
Arl2	NM_019722	0.00558	5.4299	9.35721	8.78017	1.49178	0.577037	1.49178
Mars	NM_001171582	0.003225	6.31052	9.54184	8.96567	1.49089	0.576178	1.49089
Cpsf7	NM_172302	0.000412	10.8301	7.75646	7.18065	1.49051	0.575803	1.49051
	---	0.000359	11.2228	12.7235	12.1483	1.48988	0.575192	1.48988
D030016E14Rik	NM_177240	0.004249	5.85386	8.20078	7.62567	1.48979	0.57511	1.48979
Med30	NM_027212	0.001051	8.50013	6.59895	6.02401	1.48961	0.574937	1.48961
Phb	NM_008831	9.83E-05	15.6122	9.96679	9.39233	1.48912	0.574459	1.48912
Pnpt1	NM_027869	0.003202	6.32239	8.85049	8.27608	1.48908	0.574417	1.48908
Hdgfrp2	NM_008233	0.002999	6.43524	7.89153	7.31745	1.48873	0.57408	1.48873
Tns4	NM_172564	0.00211	7.07128	5.50875	4.93469	1.48871	0.57406	1.48871
Cpsf6	NM_001013391	0.002979	6.44693	8.54123	7.9675	1.48836	0.573724	1.48836
Nipsnap1	NM_008698	0.000902	8.84446	7.2837	6.71017	1.48816	0.573525	1.48816
Ccdc66	NM_177111	0.006645	5.17145	7.04588	6.47325	1.48724	0.572633	1.48724
Slc35f3	NM_175434	1.84E-05	23.815	5.58099	5.00883	1.48675	0.572162	1.48675
Npepps	NM_008942	0.00018	13.379	9.68561	9.11471	1.48545	0.570899	1.48545
AI846148	NM_001033139	0.006826	5.13271	6.6943	6.12365	1.4852	0.570655	1.4852
Tpst2	NM_009419	0.002713	6.61148	8.18776	7.61795	1.48433	0.569812	1.48433
Ccdc123	NM_028120	0.003291	6.27571	6.4246	5.85486	1.48425	0.569735	1.48425
Zfp84	NM_023750	0.001427	7.84361	7.55746	6.98787	1.48411	0.569598	1.48411
	---	0.002041	7.13484	9.50385	8.93439	1.48397	0.569462	1.48397
Fam199x	NM_146261	0.005167	5.54685	6.70045	6.13172	1.48322	0.568729	1.48322
Gemin6	NM_026053	0.002553	6.72011	7.06514	6.49653	1.4831	0.568612	1.4831
Gin1	NM_026250	0.006645	5.1715	6.28762	5.71922	1.48288	0.568399	1.48288

Cltc	NM_001003908	0.001878	7.29381	11.572	11.0045	1.48198	0.567523	1.48198
Zfp68	NM_013844	0.005685	5.40199	8.809	8.24149	1.48195	0.567501	1.48195
	---	0.000991	8.63051	10.3873	9.81979	1.48192	0.567466	1.48192
Htt	NM_010414	0.001109	8.37979	7.3195	6.75207	1.48188	0.567429	1.48188
Ints3	NM_145540	0.000459	10.5349	8.47822	7.91131	1.48134	0.566907	1.48134
	---	0.002365	6.85978	7.15262	6.58572	1.48134	0.566901	1.48134
Ergic1	NM_026170	0.003899	5.99322	10.2993	9.73247	1.48122	0.566789	1.48122
Nudcd3	NM_173748	0.004156	5.88925	8.69171	8.12501	1.48114	0.566704	1.48114
	---	0.001437	7.82952	9.59834	9.0321	1.48065	0.566231	1.48065
	---	0.004965	5.60857	10.1003	9.53418	1.48056	0.56614	1.48056
Cwc25	NM_026186	0.002724	6.60454	6.87198	6.30592	1.48047	0.566058	1.48047
Pdcd11	NM_011053	0.001353	7.95506	8.43815	7.87233	1.48023	0.565818	1.48023
Pam	NM_013626	0.003117	6.36868	10.307	9.74136	1.48006	0.565659	1.48006
Gm10572	ENSMUST00000097894	0.000267	12.1046	5.50645	4.94174	1.47909	0.564709	1.47909
Otud1	NM_027715	0.000529	10.1568	5.98667	5.4227	1.47834	0.563975	1.47834
Plxna1	NM_008881	0.002234	6.96492	8.99776	8.43411	1.47801	0.563652	1.47801
Rbm15	NM_001045807	0.005414	5.47558	7.67351	7.10999	1.47788	0.563525	1.47788
Bptf	NM_176850	0.004221	5.86458	7.91362	7.35099	1.47695	0.562623	1.47695
Ube2k	NM_016786	2.40E-05	22.2811	9.7739	9.21206	1.47614	0.561833	1.47614
Dpp9	NM_172624	0.002141	7.0439	7.93545	7.37378	1.47599	0.561678	1.47599
Trp53inp2	NM_178111	0.000745	9.29625	7.57149	7.01004	1.47575	0.561446	1.47575
Ap1b1	NM_007454	0.000459	10.5361	9.26563	8.70459	1.47533	0.561039	1.47533
4632411B12Rik	BC132293	0.000423	10.7584	8.53029	7.96953	1.47505	0.56076	1.47505
	---	0.004076	5.92088	8.80994	8.25001	1.47421	0.559937	1.47421
Rprd1b	NM_027434	6.07E-05	17.6379	7.85121	7.29154	1.47394	0.559673	1.47394
Trmt1	NM_198020	0.000883	8.89516	9.0466	8.48711	1.47375	0.559487	1.47375
	---	0.005087	5.57083	10.5871	10.0281	1.47324	0.55899	1.47324
Eif1	NM_011508	0.0039	5.99249	11.6535	11.0947	1.47305	0.558803	1.47305
Srpk1	NM_016795	0.002267	6.93739	9.33679	8.77812	1.47292	0.558676	1.47292
Ddb1	NM_015735	0.001904	7.26809	11.6391	11.0808	1.47253	0.558295	1.47253

AA474408	ENSMUST00000098110	0.004066	5.92466	6.20237	5.64426	1.47233	0.5581	1.47233
Raf1	NM_029780	0.000905	8.83656	10.1478	9.58967	1.47232	0.55809	1.47232
Hps1	NM_019424	0.00017	13.5801	7.37249	6.81459	1.47212	0.557898	1.47212
Nsmce4a	NM_001162855	0.003303	6.26942	8.33811	7.78025	1.47208	0.557858	1.47208
Ube3c	NM_133907	0.002614	6.67777	9.4869	8.92929	1.47184	0.557617	1.47184
Scn3a	NM_018732	0.003078	6.39056	4.98765	4.43046	1.4714	0.557194	1.4714
	---	0.002585	6.69791	9.87906	9.3233	1.46995	0.555766	1.46995
Noc3l	NM_021315	0.00134	7.97395	8.57924	8.02358	1.46984	0.55566	1.46984
Gga3	NM_173048	0.001526	7.70612	7.14987	6.59454	1.46951	0.555331	1.46951
Ppia	NM_008907	0.001133	8.33313	12.4942	11.9392	1.46923	0.555062	1.46923
Klf8	NM_173780	0.002604	6.68461	4.54618	3.99114	1.4692	0.555036	1.4692
Sympk	NM_026605	0.000603	9.82016	9.02848	8.47363	1.46902	0.55485	1.46902
1300010F03Rik	NM_027906	0.005898	5.34695	7.8055	7.2511	1.46856	0.554402	1.46856
Atp6v0c	NM_009729	0.003794	6.03797	11.3327	10.7783	1.46852	0.55436	1.46852
Atf7	NM_146065	0.00095	8.72542	9.53204	8.97784	1.46836	0.554207	1.46836
Rab11fip3	NM_001162869	0.002271	6.93455	7.21387	6.66024	1.46778	0.553634	1.46778
Cstad	NM_030137	0.004562	5.74084	5.04773	4.49507	1.46679	0.552666	1.46679
Ccdc101	NM_029339	0.00509	5.57018	7.16685	6.61504	1.46592	0.551809	1.46592
0610038B21Rik	NR_028125	0.001277	8.07656	5.75995	5.20821	1.46585	0.551741	1.46585
Apoa1bp	NM_144897	0.005399	5.47994	8.99761	8.44616	1.46556	0.55145	1.46556
Usp39	NM_138592	0.004843	5.64697	9.22989	8.67868	1.46532	0.551213	1.46532
H3f3b	NM_008211	3.74E-05	19.9312	11.4787	10.9277	1.46513	0.551026	1.46513
Cstf3	NM_145529	0.004055	5.92936	7.15029	6.59936	1.46503	0.55093	1.46503
Pigo	NM_020035	8.28E-05	16.3041	6.762	6.212	1.46409	0.55	1.46409
Hist1h2be	NM_001177653	0.00143	7.83932	6.51067	5.96071	1.46404	0.549953	1.46404
Top3a	NM_009410	0.003625	6.11326	7.15317	6.60337	1.46388	0.549802	1.46388
Eif4ebp1	NM_007918	0.00196	7.21211	9.12664	8.57686	1.46386	0.549779	1.46386
Bub3	NM_009774	0.001457	7.80027	10.0608	9.5112	1.46367	0.54959	1.46367
Jmjd6	NM_033398	0.0035	6.17177	7.98027	7.43095	1.4634	0.549326	1.4634
	---	0.002089	7.09023	5.43484	4.88552	1.4634	0.54932	1.4634

Tmem184b	NM_172608	0.004814	5.65633	9.18822	8.6391	1.46319	0.549121	1.46319
Fam173a	BC096050	0.0062	5.27282	8.55292	8.00421	1.46278	0.548715	1.46278
Gm8995	AK172683	0.004051	5.93065	4.34063	3.79219	1.4625	0.548439	1.4625
Nudt22	NM_026675	0.002907	6.48981	7.81272	7.26453	1.46225	0.548191	1.46225
2810046L04Rik	NM_173382	0.000494	10.3361	7.283	6.73507	1.46199	0.547933	1.46199
Cstf1	NM_024199	0.000398	10.9315	8.06925	7.52188	1.46142	0.547367	1.46142
	---	0.000263	12.1584	12.0462	11.4989	1.46134	0.547292	1.46134
Plekhm2	NM_001033150	0.001596	7.61485	7.09466	6.54737	1.46133	0.547284	1.46133
Nudt4	NM_027722	5.52E-06	32.2348	10.969	10.4219	1.46115	0.547104	1.46115
Gnaz	NM_010311	0.004241	5.85691	5.27965	4.73282	1.46087	0.546828	1.46087
Ikzf5	NM_175115	0.005915	5.34265	8.74796	8.20127	1.46073	0.546692	1.46073
Nsfl1c	NM_198326	0.002466	6.78336	9.27844	8.73268	1.45979	0.545759	1.45979
Vps18	NM_172269	0.001873	7.29974	7.69847	7.15361	1.45888	0.544863	1.45888
Mll2	NM_001033276	0.001576	7.64105	6.71334	6.16877	1.45858	0.544568	1.45858
Hps4	NM_138646	0.00311	6.37282	7.56427	7.02003	1.45826	0.544248	1.45826
Aplf	NM_001170489	0.000196	13.1061	6.48998	5.94577	1.45822	0.544206	1.45822
Etfdh	NM_025794	0.004349	5.81669	9.65482	9.11102	1.45781	0.543801	1.45781
Rbck1	NM_019705	1.01E-05	27.7043	8.18851	7.64516	1.45735	0.543348	1.45735
Cnnm3	NM_001039551	0.000388	10.9976	6.75493	6.2116	1.45734	0.543335	1.45734
Lrrfip1	NM_008515	0.002884	6.50338	7.20348	6.6608	1.45668	0.542683	1.45668
Elk4	NM_007923	0.002141	7.04439	7.5623	7.01967	1.45662	0.542628	1.45662
Atp6v0a1	NM_016920	0.000596	9.84836	8.45403	7.91148	1.45654	0.542546	1.45654
Sart1	NM_016882	0.004361	5.8123	7.76674	7.22428	1.45646	0.542467	1.45646
Gpr172b	NM_029643	0.001566	7.65384	7.09097	6.54872	1.45625	0.542255	1.45625
Stk35	NM_183262	0.001272	8.08525	8.02663	7.4846	1.45602	0.542033	1.45602
Ndufa10	NM_024197	0.000926	8.78443	9.85551	9.31388	1.45562	0.54163	1.45562
Pcyt2	NM_024229	0.006164	5.28159	7.39977	6.85833	1.45542	0.541434	1.45542
	---	0.001871	7.30151	8.89896	8.35779	1.45514	0.541163	1.45514
Pvr	NM_027514	0.005676	5.40426	9.60174	9.06069	1.45503	0.541052	1.45503
Chac2	NM_026527	0.002248	6.95328	5.97668	5.43585	1.45482	0.540836	1.45482
Pes1	NM_022889	0.000546	10.0761	9.34038	8.7996	1.45476	0.540786	1.45476

Atp5g2	NM_026468	0.001033	8.53671	10.6118	10.0713	1.45447	0.540495	1.45447
	---	0.001664	7.53214	5.47147	4.93115	1.45429	0.540314	1.45429
	---	0.005658	5.40914	6.53878	5.99889	1.45386	0.539884	1.45386
Sap130	NM_172965	0.006107	5.2952	7.98063	7.44088	1.45372	0.539752	1.45372
Plac1	NM_019538	0.004122	5.90281	4.36393	3.82477	1.45312	0.539158	1.45312
Uqcrc2	NM_025899	2.34E-05	22.4274	10.6184	10.0793	1.45307	0.539102	1.45307
Eif5a2	NM_177586	0.003692	6.08277	7.29805	6.75944	1.45257	0.538603	1.45257
Fam120b	NR_033586	0.006721	5.15496	7.55257	7.01424	1.45229	0.538326	1.45229
Pld4	NM_178911	0.005908	5.34423	4.51682	3.97977	1.451	0.53705	1.451
Dmwd	NM_010058	0.000692	9.47427	7.27504	6.73859	1.45041	0.536459	1.45041
Itprip	NM_001001738	0.006543	5.19398	6.2126	5.67618	1.45036	0.536413	1.45036
Flii	NM_022009	0.000718	9.38719	9.39349	8.85735	1.45008	0.536136	1.45008
Casc3	NM_138660	0.000347	11.3219	7.35133	6.81572	1.44955	0.535607	1.44955
F630110N24Rik	BC119790	0.003288	6.27707	7.10006	6.56462	1.44939	0.535444	1.44939
Tars2	NM_027931	0.002606	6.6831	8.01157	7.47711	1.4484	0.534459	1.4484
Timm22	NM_019818	0.005297	5.50882	7.55194	7.01794	1.44794	0.534002	1.44794
Aldoa	NM_001177307	0.003208	6.31907	12.1066	11.5726	1.44792	0.533982	1.44792
Psmc4	NM_011874	0.002684	6.63065	9.08221	8.549	1.44715	0.533212	1.44715
Dcaf13	NM_198606	0.00053	10.1512	9.27558	8.74241	1.44711	0.533177	1.44711
Timm50	NM_025616	0.002104	7.07693	7.67742	7.14465	1.44671	0.532775	1.44671
Acox1	NM_015729	0.003053	6.40438	9.49988	8.96726	1.44655	0.532614	1.44655
	---	0.000245	12.3788	9.65279	9.1203	1.44643	0.532496	1.44643
Mvk	NM_023556	0.000291	11.8473	7.02837	6.49616	1.44614	0.532206	1.44614
	---	0.001832	7.34286	9.29976	8.76763	1.44606	0.53213	1.44606
Ddx54	NM_028041	0.001144	8.31176	8.36377	7.83193	1.44577	0.531842	1.44577
Adar	NM_001038587	0.000993	8.62509	7.71017	7.1786	1.4455	0.531569	1.4455
Slc25a26	NM_026255	0.005459	5.46299	7.26956	6.73807	1.44542	0.531489	1.44542
Smpd1	NM_011421	0.002124	7.05953	9.48297	8.95154	1.44536	0.531432	1.44536
Cep152	NM_001081091	0.004459	5.77703	5.63585	5.10445	1.44533	0.531401	1.44533
Rnf139	NM_175226	0.000691	9.47981	8.74354	8.21251	1.44496	0.53103	1.44496
Mast3	NM_199308	0.000733	9.33539	6.76927	6.23839	1.44481	0.53088	1.44481

9130401M01Rik	BC050895	0.003766	6.04991	8.12003	7.58918	1.44478	0.530854	1.44478
Fam40a	NM_153563	0.002702	6.6189	8.30223	7.77176	1.4444	0.530467	1.4444
Calcb	NM_054084	0.001281	8.07018	5.0202	4.48993	1.4442	0.530271	1.4442
Trove2	NM_013835	0.004531	5.75153	8.76887	8.23869	1.44411	0.530178	1.44411
Gzf1	NM_028986	0.003265	6.28941	8.11241	7.58225	1.44409	0.530164	1.44409
Arfgef1	NM_001102430	0.001568	7.65076	8.95967	8.42957	1.44403	0.530097	1.44403
Ciao1	NM_025296	0.006537	5.19529	8.72713	8.19735	1.44371	0.529782	1.44371
Pigg	NM_001081234	0.000317	11.5877	8.02531	7.49556	1.44368	0.529754	1.44368
Ogfod1	NM_177767	0.000715	9.39675	8.00063	7.47094	1.44362	0.52969	1.44362
Usp15	NM_027604	0.0022	6.99332	8.88132	8.35175	1.4435	0.529571	1.4435
Poll	NM_020032	0.003242	6.30107	5.99589	5.46634	1.44348	0.529554	1.44348
Farsa	NM_025648	0.00021	12.8743	8.01498	7.48554	1.44337	0.529442	1.44337
Dlg4	NM_007864	0.001412	7.8661	8.31732	7.78838	1.44286	0.528936	1.44286
Kirrel3	NM_001190911	0.000126	14.649	5.41291	4.88411	1.44274	0.528808	1.44274
Mrps23	NM_024174	0.001126	8.3471	8.80211	8.27357	1.44247	0.528541	1.44247
Tbc1d22b	NM_198647	0.002751	6.58698	6.69444	6.16607	1.44229	0.528363	1.44229
Mll2	NM_001033276	0.006145	5.28591	7.23433	6.70637	1.44189	0.52796	1.44189
Galk1	NM_016905	0.000257	12.2252	7.8488	7.32146	1.44127	0.527336	1.44127
Depdc5	NM_001025426	0.000295	11.8066	6.91468	6.38803	1.44058	0.526648	1.44058
Gipc1	NM_018771	0.000818	9.07388	8.42789	7.90125	1.44058	0.526645	1.44058
Fbxo7	NM_153195	0.003451	6.19537	7.83633	7.31015	1.44011	0.526182	1.44011
Ubr5	NM_001081359	0.003464	6.18916	9.92085	9.39476	1.44002	0.526093	1.44002
Eny2	NM_175009	0.004467	5.7741	9.51403	8.98839	1.43957	0.52564	1.43957
Csrp2bp	NM_181417	0.001639	7.5626	8.0421	7.51659	1.43943	0.525502	1.43943
Wdr43	NM_175639	0.006404	5.22528	9.74221	9.21694	1.4392	0.52527	1.4392
Ppargc1a	NR_027710	0.003238	6.30319	4.79276	4.26755	1.43915	0.525219	1.43915
Bicd2	NM_001039179	0.001603	7.60665	7.64139	7.1164	1.43892	0.52499	1.43892
Mrpl44	NM_001081210	0.006134	5.28864	7.03688	6.5123	1.43851	0.52458	1.43851
Tmem57	NM_025382	0.0007	9.44664	8.38205	7.85752	1.43846	0.524525	1.43846
Cct6a	NM_009838	0.006255	5.25993	10.1196	9.5952	1.43834	0.524406	1.43834
Gclm	NM_008129	0.004468	5.77354	8.94054	8.41636	1.43812	0.524186	1.43812

Luc7l	NM_028190	0.004743	5.67979	8.62132	8.09768	1.43758	0.523641	1.43758
Rnps1	NM_009070	0.003328	6.25686	9.36032	8.83682	1.43745	0.523507	1.43745
Atf7ip	NM_019426	0.000638	9.6761	9.64686	9.12367	1.43712	0.523183	1.43712
Ndufv1	NM_133666	0.000866	8.93844	9.57453	9.05156	1.43692	0.522976	1.43692
	---	0.000548	10.0651	12.3648	11.8418	1.43689	0.522951	1.43689
Atl3	NM_146091	0.000258	12.2155	10.5439	10.0216	1.43629	0.522349	1.43629
	---	0.004547	5.74583	10.1798	9.65752	1.43619	0.522243	1.43619
	---	0.004899	5.62922	9.81908	9.2969	1.43613	0.522182	1.43613
Foxp4	NM_001110824	0.001466	7.78816	6.88167	6.36013	1.43548	0.521538	1.43548
	---	0.000163	13.7295	12.1332	11.6118	1.43534	0.521388	1.43534
Mrps5	NM_029963	0.002103	7.07836	7.46072	6.9402	1.43447	0.520522	1.43447
Wdr81	NM_138950	0.003292	6.27516	7.4899	6.97097	1.43289	0.518933	1.43289
Wdtc1	NM_199306	0.002983	6.44462	7.16432	6.64568	1.43261	0.518646	1.43261
	---	0.003052	6.40495	11.6101	11.0916	1.43252	0.518551	1.43252
Ppp1r13l	NM_001010836	0.004883	5.63443	6.26233	5.7441	1.4322	0.518231	1.4322
Unk	NM_172569	0.000607	9.80308	7.31978	6.80158	1.43216	0.518195	1.43216
Casp8	NM_009812	0.002264	6.93979	8.76309	8.24515	1.43191	0.517942	1.43191
Tmem85	NM_026519	0.003887	5.99799	9.8616	9.34377	1.43181	0.517837	1.43181
	---	0.000672	9.54788	6.18045	5.66275	1.43167	0.517695	1.43167
Ccdc13	NM_028384	0.00547	5.45994	5.27895	4.76205	1.43088	0.516902	1.43088
Tsku	NM_001168541	0.000732	9.33802	7.36309	6.84651	1.43057	0.516586	1.43057
H3f3b	NM_008211	0.002447	6.79749	11.8308	11.3147	1.43008	0.516091	1.43008
Xrcc1	NM_009532	0.005396	5.4806	7.20207	6.68605	1.43	0.516013	1.43
	---	0.007039	5.08854	3.33535	2.81943	1.42991	0.51592	1.42991
Cisd1	BC013522	0.002743	6.59183	5.98598	5.47137	1.42861	0.514611	1.42861
Mafg	NM_010756	0.002509	6.75146	8.42094	7.90646	1.42848	0.514481	1.42848
Clptm1	NM_019649	0.000525	10.1765	10.1902	9.6758	1.42838	0.514379	1.42838
Klhl7	NM_026448	0.005924	5.3402	8.75204	8.23829	1.42776	0.513751	1.42776
Vmn2r43	NM_198961	0.001003	8.60455	3.84029	3.32687	1.42743	0.513421	1.42743
Smarcad1	NM_007958	0.004771	5.67037	9.1253	8.61196	1.42735	0.51334	1.42735
	---	0.00219	7.00214	9.49065	8.9778	1.42686	0.512848	1.42686

Pcdh10	NM_001098171	0.001651	7.54753	4.67638	4.16357	1.42682	0.512806	1.42682
Samm50	NM_178614	0.001262	8.101	8.66375	8.15095	1.42682	0.512803	1.42682
Tug1	NR_002321	0.005058	5.57989	10.1268	9.61514	1.42572	0.511695	1.42572
Ccdc97	NM_028771	0.000335	11.4196	7.42374	6.9121	1.42567	0.51164	1.42567
Tubgcp4	NM_153387	0.003458	6.1922	7.99414	7.48256	1.42561	0.511576	1.42561
Prmt6	NM_178891	0.001714	7.47265	6.70654	6.19515	1.42542	0.511389	1.42542
Mrgpre	NM_175534	0.001442	7.82171	5.69266	5.1813	1.4254	0.511368	1.4254
	---	0.002439	6.8033	9.73222	9.22086	1.42539	0.511354	1.42539
Mrpl16	NM_025606	0.004487	5.76695	7.70253	7.19168	1.42489	0.510852	1.42489
Ipo11	NM_029665	0.000812	9.0917	8.63049	8.11977	1.42476	0.510722	1.42476
Msh6	NM_010830	0.006347	5.23846	7.46296	6.95356	1.42347	0.509408	1.42347
	---	0.003065	6.39797	9.16964	8.66025	1.42345	0.509392	1.42345
Trappc9	NM_180662	0.002155	7.03206	7.67991	7.17074	1.42322	0.509162	1.42322
Sbno1	NM_001081203	0.003147	6.35213	9.20276	8.69372	1.42311	0.509043	1.42311
Eri1	NM_026067	0.000514	10.2325	8.30344	7.79485	1.42266	0.508591	1.42266
Pnpla6	NM_001122818	0.002204	6.99011	7.8522	7.34394	1.42234	0.508264	1.42234
Uba2	NM_016682	0.002172	7.0169	9.50313	8.99546	1.42175	0.507667	1.42175
4933407H18Rik	NM_001081101	0.000107	15.2929	6.9268	6.41949	1.4214	0.507315	1.4214
Eif3d	NM_018749	0.005227	5.52919	9.95156	9.44432	1.42132	0.507234	1.42132
Mlx	NM_011550	0.004429	5.78776	8.42681	7.92005	1.42085	0.506752	1.42085
Dnajc5	NM_016775	0.00355	6.14825	8.8182	8.31151	1.42078	0.506687	1.42078
Spats2	NM_139140	0.000961	8.69901	9.10133	8.59471	1.42072	0.506618	1.42072
Ankrd40	NM_027799	0.002374	6.85218	8.61667	8.11009	1.42068	0.506585	1.42068
Btbd2	NM_145361	0.00052	10.201	8.03788	7.53178	1.4202	0.506095	1.4202
Fastkd5	NM_198176	0.002447	6.7972	6.96759	6.46167	1.42003	0.50592	1.42003
Timm17a	NM_011590	0.000978	8.66019	9.13579	8.62991	1.41998	0.505872	1.41998
Snd1	NM_019776	0.000692	9.47644	10.0898	9.58444	1.4195	0.50538	1.4195
Spred1	NM_033524	0.005232	5.52769	8.8059	8.30083	1.41919	0.505068	1.41919
Ncbp1	NM_001033201	0.003246	6.29901	8.76974	8.26477	1.41909	0.504967	1.41909
Tmed4	NM_134020	0.000923	8.79309	10.137	9.63214	1.41902	0.504896	1.41902
Mrpl46	NM_023331	0.005898	5.34687	6.7915	6.2868	1.41883	0.504706	1.41883

Wdr70	NM_001081402	0.000196	13.1	8.90549	8.40102	1.4186	0.504469	1.4186
Mrpl49	NM_026246	0.002587	6.69652	8.52968	8.02535	1.41846	0.504329	1.41846
Crtc2	NM_028881	0.000465	10.4984	7.80001	7.29591	1.41824	0.504105	1.41824
Gpatch8	NM_001159492	0.003631	6.11044	7.44835	6.94427	1.41822	0.50408	1.41822
Olfr1392	NM_146470	0.004263	5.84844	4.35552	3.85147	1.41819	0.504055	1.41819
Akap8l	NM_017476	0.003723	6.06887	8.14544	7.64203	1.41757	0.503418	1.41757
Rnps1	NM_009070	0.005729	5.39037	9.34856	8.84532	1.41739	0.503239	1.41739
Polr1c	NM_009085	0.00137	7.92831	9.0338	8.53064	1.41732	0.50316	1.41732
	---	0.000555	10.0313	6.39095	5.88811	1.417	0.502836	1.417
Urgcp	NM_178623	0.003582	6.133	7.67185	7.16915	1.41686	0.502698	1.41686
Fam193a	BC099925	0.005624	5.4181	6.89404	6.39141	1.4168	0.502632	1.4168
Rgl2	NM_009059	0.000877	8.91093	7.69096	7.18864	1.41649	0.50232	1.41649
Dpf2	NM_011262	0.002447	6.79751	9.30188	8.79979	1.41626	0.502087	1.41626
Ddx18	NM_025860	0.004305	5.83279	9.63703	9.13516	1.41605	0.501869	1.41605
Sumf1	NM_145937	0.002575	6.70495	9.66604	9.16418	1.41603	0.501855	1.41603
Ube2q1	NM_027315	0.00072	9.37736	8.61579	8.11424	1.41573	0.501547	1.41573
Ptpn12	NM_011203	0.000969	8.68133	9.28845	8.78729	1.41535	0.501159	1.41535
Vps39	NM_147153	0.000707	9.42338	8.65572	8.15492	1.415	0.500801	1.415
Mark3	NM_021516	0.005258	5.52031	8.37811	7.87811	1.41422	0.500007	1.41422
Ggnbp2	NM_153144	0.000622	9.7405	9.66015	9.16026	1.41411	0.499892	1.41411
	---	0.004056	5.92879	10.5446	10.045	1.41381	0.499589	1.41381
Ganab	NM_008060	0.004302	5.83388	10.9642	10.4647	1.41367	0.499447	1.41367
	---	0.006813	5.1353	9.92119	9.42244	1.41299	0.498754	1.41299
Nr3c1	NM_008173	0.001565	7.65485	9.45186	8.95311	1.41298	0.498746	1.41298
Itfg3	NM_207217	0.000143	14.1982	9.41352	8.91489	1.41287	0.498625	1.41287
Rad23b	NM_009011	0.000716	9.39319	9.20388	8.7057	1.41243	0.498181	1.41243
Gde1	NM_019580	0.000579	9.92228	9.05853	8.56105	1.41175	0.497481	1.41175
Pptc7	NM_177242	0.000578	9.92926	8.09793	7.60081	1.4114	0.497123	1.4114
Sin3b	NM_009188	0.004522	5.75473	8.73078	8.23373	1.41133	0.497052	1.41133
Fam116b	NM_027081	0.000337	11.4097	6.03749	5.54129	1.4105	0.496204	1.4105
Tbc1d25	NM_172478	4.04E-05	19.5479	6.85888	6.36273	1.41045	0.496156	1.41045

Nhp2l1	NM_011482	0.002299	6.91195	11.3732	10.8771	1.41038	0.496084	1.41038
	---	0.003031	6.41684	10.9549	10.4592	1.41	0.495696	1.41
Smek2	NM_134034	0.004918	5.62328	9.28796	8.79249	1.40978	0.495468	1.40978
Ptpn3	NM_011207	0.002975	6.44934	6.21355	5.71845	1.40942	0.495105	1.40942
Cox4nb	NM_010926	0.006499	5.20378	7.79663	7.3023	1.40866	0.494324	1.40866
Ptrh1	NM_178595	0.001036	8.53013	7.89144	7.3972	1.40857	0.494236	1.40857
Nfe2l1	NM_008686	0.006656	5.16912	10.3661	9.87202	1.4084	0.494053	1.4084
Cnot7	NM_011135	0.000381	11.054	9.15517	8.66159	1.40794	0.493583	1.40794
Ube2v1	NM_023230	0.000904	8.83958	9.08271	8.5893	1.40776	0.493402	1.40776
Lass5	NM_028015	0.001006	8.59684	10.1517	9.65844	1.40764	0.493274	1.40764
Gatad1	NM_026033	0.001863	7.30974	9.51028	9.01802	1.40665	0.492259	1.40665
Mrpl10	NM_026154	0.002043	7.13244	8.75527	8.26309	1.40657	0.492181	1.40657
Kif13a	NM_010617	0.006335	5.24123	7.08033	6.5884	1.40633	0.491932	1.40633
Ppp3r1	NM_024459	0.003341	6.25037	9.2614	8.76949	1.40631	0.491912	1.40631
Prpf3	NM_027541	0.001567	7.65239	7.96606	7.47524	1.40524	0.49082	1.40524
Arl2bp	NM_024191	0.003689	6.08408	10.187	9.69645	1.40493	0.490503	1.40493
	---	0.001912	7.25942	8.78294	8.29315	1.40424	0.489792	1.40424
Ppia	NM_008907	0.000516	10.2221	12.4706	11.9827	1.40236	0.487856	1.40236
Emp3	NM_010129	0.005108	5.56467	11.4358	10.9482	1.40208	0.487569	1.40208
Paf1	NM_019458	7.69E-05	16.6128	9.39656	8.9091	1.40197	0.487456	1.40197
Stat6	NM_009284	0.002889	6.5007	9.29444	8.807	1.40196	0.487443	1.40196
Helz	NM_198298	0.004719	5.68773	8.06703	7.57993	1.40163	0.487102	1.40163
Mut	NM_008650	0.000425	10.747	9.0398	8.55282	1.40151	0.486981	1.40151
Dot1l	ENSMUST0000010533 6	0.002844	6.52836	6.88032	6.39407	1.40079	0.486243	1.40079
Strada	NM_028126	0.00152	7.7146	7.7539	7.2685	1.39997	0.485398	1.39997
Nhp2l1	NM_011482	0.002813	6.5476	11.0823	10.5969	1.39996	0.485388	1.39996
Paics	NM_025939	0.005798	5.37232	11.8101	11.3247	1.39995	0.485379	1.39995
Rbm28	NM_133925	0.000837	9.01847	7.46789	6.98367	1.39884	0.484227	1.39884
Csnk1e	NM_013767	0.00232	6.89495	9.13701	8.6528	1.39882	0.48421	1.39882
Sec24b	NM_207209	0.004136	5.89715	8.76239	8.27861	1.39841	0.483786	1.39841

Ewsr1	NM_007968	0.003805	6.03297	6.81941	6.33608	1.39796	0.483328	1.39796
Cct2	NM_007636	0.003902	5.99172	11.571	11.0877	1.39791	0.483269	1.39791
Gcn1l1	NM_172719	0.000566	9.98076	8.37501	7.89179	1.39786	0.483221	1.39786
Dsc1	ENSMUST00000025187	0.002573	6.70662	6.8465	6.3633	1.39784	0.4832	1.39784
Casq2	NM_009814	0.001382	7.91043	4.85138	4.36837	1.39766	0.483017	1.39766
Smarce1	NM_020618	0.001148	8.30494	10.0583	9.57543	1.39756	0.482909	1.39756
	---	0.004632	5.71674	9.0993	8.61716	1.39682	0.482141	1.39682
Snrnp48	NM_026382	0.002533	6.73441	6.60589	6.12384	1.39672	0.48204	1.39672
Rnf115	NM_026406	0.004779	5.66784	8.68645	8.20453	1.3966	0.481922	1.3966
St13	NM_133726	0.003632	6.11014	10.0903	9.60884	1.39616	0.481465	1.39616
Iffo1	NM_178787	0.003815	6.02872	7.11501	6.63407	1.39566	0.480948	1.39566
Snx17	NM_153680	0.005301	5.5079	9.69137	9.21101	1.3951	0.480364	1.3951
Oscp1	NM_172701	0.000404	10.8853	6.13768	5.65739	1.39502	0.480291	1.39502
Flywch1	NM_153791	0.006953	5.10602	7.38096	6.90089	1.39481	0.480064	1.39481
4931428F04Rik	NM_028888	0.004999	5.59786	5.64794	5.16876	1.39395	0.47918	1.39395
Haus5	NM_027999	0.004986	5.60183	6.23808	5.75903	1.39382	0.479046	1.39382
Lyrm7	NM_029327	0.001478	7.77152	4.38829	3.90941	1.39366	0.478879	1.39366
Eif4enif1	NM_023743	0.000682	9.51305	8.0339	7.55508	1.3936	0.478818	1.3936
	---	0.000105	15.347	6.67894	6.20142	1.39235	0.477522	1.39235
Ube2v1	NM_023230	0.001203	8.20293	9.34742	8.87003	1.39222	0.477386	1.39222
Xpnpep1	NM_133216	0.002535	6.73334	9.56006	9.08278	1.39211	0.477274	1.39211
	---	0.003314	6.26376	5.15139	4.67423	1.39201	0.477168	1.39201
Dthd1	NM_001170705	0.004714	5.68919	3.52717	3.05018	1.39183	0.476985	1.39183
Tasp1	NM_175225	0.000319	11.5645	7.00403	6.52752	1.39138	0.476517	1.39138
Smarce1	NM_020618	0.00146	7.79651	10.053	9.57659	1.39127	0.476399	1.39127
Pabpn1	NM_019402	0.000813	9.08719	9.23258	8.75621	1.39124	0.476368	1.39124
Tph1	NM_009414	0.003053	6.40443	4.4529	3.97662	1.39115	0.476278	1.39115
Tapt1	NM_173764	0.000676	9.53204	9.53929	9.06332	1.39085	0.475971	1.39085
Ppia	NM_008907	0.000515	10.2302	12.5868	12.1108	1.39082	0.475937	1.39082
Rabif	NM_145510	0.002332	6.88497	8.05526	7.57939	1.39076	0.475871	1.39076

Rev1	NM_019570	0.002184	7.00729	8.30369	7.82807	1.39051	0.475618	1.39051
Mxra8	NM_024263	0.006007	5.31971	11.2056	10.7302	1.39029	0.475383	1.39029
Parp4	NM_001145978	0.001222	8.17028	7.43385	6.95863	1.39013	0.475222	1.39013
Pdxp	NM_020271	0.001191	8.22622	6.02229	5.54712	1.39008	0.475172	1.39008
Secisbp2l	NM_177608	0.002157	7.02983	8.18215	7.70861	1.38852	0.473544	1.38852
Ppp4r1	NM_146081	0.000143	14.1971	9.31413	8.84076	1.38835	0.473374	1.38835
Gabbr1	NM_019439	0.002275	6.93061	7.18045	6.70744	1.38801	0.473015	1.38801
Gnb1	NM_008142	0.005992	5.32338	11.654	11.1812	1.38781	0.472805	1.38781
Efna5	NM_207654	0.001471	7.78085	6.75804	6.28574	1.38732	0.472297	1.38732
Eps15l1	NM_007944	0.000704	9.4347	8.01023	7.538	1.38725	0.472231	1.38725
3000002C10Rik	NR_033215	0.006387	5.22922	5.62547	5.15368	1.38683	0.471786	1.38683
	---	0.00034	11.384	12.207	11.7352	1.38677	0.471731	1.38677
B230315N10Rik	NM_001105557	0.004834	5.64997	8.67178	8.2004	1.38644	0.471382	1.38644
Smarce1	NM_020618	0.001502	7.73839	10.1731	9.70174	1.38642	0.471365	1.38642
Ptdss2	NM_013782	0.000458	10.539	7.8344	7.36325	1.38622	0.471152	1.38622
Snrpb2	NM_021335	0.004034	5.93758	9.17606	8.70672	1.38448	0.469348	1.38448
Zkscan17	NM_172941	0.000819	9.06959	7.52047	7.05117	1.38444	0.469305	1.38444
Tomm40	NM_001109748	0.001452	7.80741	8.49058	8.02168	1.38405	0.468899	1.38405
Fbxo11	NM_001081034	0.004588	5.7318	9.38961	8.92089	1.38388	0.46872	1.38388
Stac2	NM_146028	0.005385	5.48374	5.686	5.21752	1.38365	0.468482	1.38365
BC053749	BC053749	0.002064	7.11351	5.70527	5.23679	1.38365	0.468482	1.38365
Eif1	NM_011508	0.003714	6.07311	10.6212	10.1528	1.38365	0.468481	1.38365
Micall2	NM_174850	0.004232	5.86027	6.37794	5.91011	1.38302	0.467823	1.38302
Brd1	NM_001033274	0.003684	6.08665	8.73945	8.27215	1.38252	0.4673	1.38252
Ppp1r12b	NM_001081307	0.001265	8.0956	5.04159	4.57553	1.38133	0.466056	1.38133
Ube2n	NM_080560	0.004946	5.61441	8.98163	8.51565	1.38126	0.465982	1.38126
Mpv17l	NM_033564	0.007062	5.08372	5.99487	5.52907	1.38108	0.465797	1.38108
Dhx57	NM_001163759	0.005509	5.44929	8.29157	7.82584	1.38101	0.465724	1.38101
Diablo	NM_023232	0.005983	5.32568	6.77615	6.31137	1.38011	0.46478	1.38011
Prpf6	NM_133701	0.002714	6.61061	9.47406	9.00958	1.37983	0.464487	1.37983
Zfp384	NM_175557	0.001959	7.21244	8.8639	8.39942	1.37982	0.464477	1.37982

	---	0.004768	5.6714	9.41046	8.94614	1.37967	0.464322	1.37967
	---	0.001646	7.55307	9.90512	9.44113	1.37935	0.463988	1.37935
Stx11	NM_029075	0.005675	5.40468	7.54806	7.08412	1.37931	0.463944	1.37931
Csn3	NM_007786	0.001715	7.47172	3.60442	3.14083	1.37897	0.463586	1.37897
Tcf3	NM_001164147	0.005666	5.40689	8.49109	8.02815	1.37835	0.462943	1.37835
Gm5113	NM_001033540	0.001671	7.52331	5.83681	5.37568	1.37662	0.46113	1.37662
Ctbs	NM_028836	0.001375	7.92064	7.19604	6.73518	1.37636	0.460859	1.37636
Scyl2	NM_198021	0.003467	6.18772	9.00352	8.54352	1.37554	0.46	1.37554
Gcap14	NM_027045	0.000928	8.78062	9.24221	8.78227	1.37549	0.459942	1.37549
AY358078	AY358078	0.004596	5.72909	3.18368	2.72418	1.37506	0.459495	1.37506
Ispd	NM_178629	0.004873	5.63753	4.82567	4.36641	1.37483	0.459257	1.37483
Tom1	NM_011622	0.004407	5.7957	8.06496	7.60611	1.37444	0.458848	1.37444
Rela	NM_009045	0.00107	8.46042	8.39139	7.93275	1.37424	0.458632	1.37424
Ipo9	NM_153774	0.005099	5.56722	9.65688	9.19846	1.37403	0.458415	1.37403
Supt5h	NM_013676	0.001456	7.80279	9.61244	9.15409	1.37397	0.458349	1.37397
Atad1	NM_026487	0.002698	6.6211	9.9092	9.45144	1.3734	0.457754	1.3734
Cd200	NM_010818	0.001858	7.31517	4.11555	3.65799	1.37323	0.457569	1.37323
D830014E11Rik	ENSMUST0000005599<br>4	0.005706	5.39646	4.56104	4.10359	1.37312	0.457457	1.37312
Vti1a	NM_016862	0.004493	5.76477	7.30557	6.84858	1.37268	0.456991	1.37268
Nedd8	NM_008683	0.000524	10.1825	10.3637	9.9068	1.37258	0.456895	1.37258
Zfp511	NM_027201	0.001647	7.55195	8.12975	7.67309	1.37236	0.456659	1.37236
Fam100b	NM_176902	8.46E-05	16.2159	7.59915	7.14253	1.37232	0.456622	1.37232
Hsp90ab1	NM_008302	0.001541	7.68568	12.2834	11.827	1.3721	0.456388	1.3721
Ngdn	NM_026890	0.005088	5.57077	7.40303	6.94666	1.37208	0.456368	1.37208
Lsg1	NM_178069	0.00535	5.49388	8.56411	8.10812	1.37173	0.455992	1.37173
Abcf2	NM_013853	0.000814	9.08399	9.55832	9.10239	1.37166	0.455928	1.37166
Ikzf5	NM_175115	0.001703	7.48557	9.39248	8.93662	1.3716	0.455857	1.3716
Stk11	NM_011492	0.00525	5.52259	9.47773	9.02187	1.37159	0.455854	1.37159
Cul4a	NM_146207	0.002991	6.44022	8.6326	8.17688	1.37146	0.455712	1.37146
Cabin1	NM_172549	0.006104	5.29601	7.66016	7.20464	1.37128	0.455522	1.37128

1700001G17Rik	NR_033199	0.00657	5.18798	5.9971	5.54217	1.37072	0.454929	1.37072
Mks1	NM_001039684	0.00519	5.54019	6.57498	6.12046	1.37033	0.454521	1.37033
Cpne1	NM_170588	0.002329	6.88789	9.18706	8.73259	1.37028	0.454469	1.37028
Vta1	NM_025418	0.000569	9.96668	9.10092	8.64669	1.37006	0.454236	1.37006
Atp13a1	NM_133224	0.0051	5.567	8.07611	7.62203	1.36991	0.454082	1.36991
Wbp11	NM_021714	0.001238	8.14282	8.328	7.87478	1.3691	0.453223	1.3691
Zfp668	NM_146259	0.001999	7.17455	5.71224	5.25972	1.36843	0.452525	1.36843
Lrpprc	NM_028233	0.004856	5.64293	9.13513	8.68475	1.3664	0.45038	1.3664
Rnf6	NM_028774	0.002631	6.66607	9.01827	8.56933	1.36504	0.448945	1.36504
Wdr92	NM_178909	0.001309	8.02423	7.01658	6.5679	1.36478	0.448671	1.36478
Doc2g	NM_021791	0.000686	9.49607	6.42844	5.98003	1.36453	0.448404	1.36453
Ppia	NM_008907	0.002188	7.00334	12.7013	12.253	1.36444	0.448305	1.36444
Rfk	NM_019437	0.002841	6.53021	9.07292	8.62528	1.36381	0.44764	1.36381
Dhrs7b	NM_001172112	0.000667	9.56735	7.99766	7.55023	1.3636	0.447425	1.3636
Dak	NM_145496	0.004678	5.70133	7.73833	7.29107	1.36345	0.447263	1.36345
	---	0.0031	6.37802	5.86793	5.42074	1.36338	0.447193	1.36338
Dhx34	NM_027883	0.003764	6.05095	6.84031	6.39318	1.36333	0.447132	1.36333
B4galt2	NM_017377	0.00311	6.37242	7.20448	6.75745	1.36323	0.447029	1.36323
Rpl30	NM_009083	0.000836	9.02102	9.013	8.56638	1.36285	0.446625	1.36285
Xrcc2	NM_020570	0.001846	7.32769	5.97054	5.52421	1.36257	0.446327	1.36257
1810009A15Rik	BC047099	0.001255	8.1135	6.37809	5.93266	1.36172	0.44543	1.36172
	---	0.003742	6.0608	9.13988	8.69462	1.36155	0.445254	1.36155
	---	0.005214	5.53303	9.59686	9.15217	1.36102	0.444692	1.36102
Acyp1	NM_025421	0.004716	5.68861	6.34363	5.89913	1.36084	0.444499	1.36084
Ankrd50	NM_001167883	0.003901	5.99216	7.92148	7.47701	1.36081	0.444465	1.36081
Ube2n	NM_080560	0.00415	5.89185	8.93447	8.4901	1.36071	0.444365	1.36071
Mkks	NM_021527	0.00237	6.8554	7.6616	7.21728	1.36067	0.444319	1.36067
Grsf1	NM_178700	0.003627	6.11246	8.79569	8.35182	1.36024	0.443864	1.36024
Sugp1	NM_027481	0.005538	5.4415	7.36565	6.92229	1.35976	0.443354	1.35976
Pfn1	NM_011072	0.00685	5.12751	10.3755	9.9322	1.3597	0.443289	1.3597
BC030336	NM_001164580	0.004144	5.89408	8.59415	8.15105	1.35952	0.443097	1.35952

Tom1	NM_011622	0.004753	5.67648	8.10915	7.66643	1.35916	0.442714	1.35916
Akap8	NM_019774	0.000858	8.96216	9.25632	8.81363	1.35914	0.442691	1.35914
	---	0.001879	7.29265	7.24387	6.80184	1.35852	0.442031	1.35852
Zfp64	NM_009564	0.003079	6.39003	6.93075	6.48898	1.35827	0.441774	1.35827
Cfl1	NM_007687	0.00052	10.2044	11.5485	11.1071	1.35792	0.441402	1.35792
Ewsr1	NM_007968	0.006499	5.20379	10.558	10.1171	1.35747	0.44092	1.35747
Kif3b	NM_008444	0.002846	6.52707	8.29512	7.85466	1.35704	0.440466	1.35704
Asb7	NR_003961	0.006271	5.2562	5.90169	5.46143	1.35685	0.440264	1.35685
Glt25d1	NM_146211	0.005325	5.5009	9.84253	9.40262	1.35652	0.439914	1.35652
Klhdc3	NM_027910	0.000194	13.143	8.73126	8.29141	1.35646	0.439847	1.35646
Rgs19	NM_026446	0.005811	5.36894	8.0015	7.56216	1.35599	0.439344	1.35599
Sharpin	NM_025340	0.00303	6.4174	9.23566	8.79639	1.35592	0.43927	1.35592
2310057M21Rik	BC044749	0.00134	7.97482	7.71308	7.2739	1.35584	0.439182	1.35584
Cdk17	NM_146239	0.0053	5.50805	8.29952	7.8607	1.3555	0.438823	1.3555
Nap1l2	NM_008671	0.003113	6.37109	3.32226	2.88362	1.35533	0.43864	1.35533
Pi4k2a	NM_145501	0.003257	6.29367	8.6903	8.25249	1.35455	0.437816	1.35455
Ndufa3	NM_025348	7.28E-06	30.0709	8.81037	8.37266	1.35445	0.437711	1.35445
Gatsl2	NM_030719	0.002443	6.80012	6.93196	6.49446	1.35426	0.4375	1.35426
Fxr2	NM_011814	0.004111	5.90708	8.66918	8.23203	1.35392	0.437148	1.35392
Commd9	NM_029635	0.003039	6.41236	7.24792	6.81113	1.35359	0.436789	1.35359
G3bp2	NM_011816	0.003199	6.32406	9.79979	9.36386	1.35279	0.435937	1.35279
Zbtb49	NM_029162	0.000555	10.0319	6.43215	5.9976	1.35149	0.434549	1.35149
	---	0.004713	5.68967	10.4328	9.99856	1.35115	0.43419	1.35115
Ces2f	BC117742	4.58E-05	18.9358	3.77467	3.34073	1.35092	0.43394	1.35092
Tpp2	NM_009418	0.00043	10.7149	8.89865	8.46488	1.35075	0.433764	1.35075
4933434E20Rik	NM_025762	0.000745	9.29478	9.69649	9.26308	1.35042	0.433412	1.35042
	---	0.001575	7.64211	4.37349	3.94091	1.34964	0.432578	1.34964
Mmab	NM_029956	0.001358	7.94646	7.14994	6.71743	1.34958	0.432511	1.34958
Ppia	NM_008907	0.00565	5.4113	12.3399	11.9075	1.34947	0.432388	1.34947
Terf2	NM_009353	0.0003	11.756	7.67265	7.2407	1.34906	0.431958	1.34906
Evi5	NM_007964	0.004811	5.6575	8.30081	7.86903	1.34889	0.431773	1.34889

	---	0.004259	5.85006	10.8395	10.4084	1.34823	0.431066	1.34823
Slc45a4	NM_001033219	0.001091	8.41617	7.807	7.37606	1.34811	0.430941	1.34811
Ppm1h	NM_001110218	0.000623	9.73829	5.76801	5.33725	1.34795	0.430762	1.34795
Mrpl21	NM_172252	0.003906	5.99006	7.46954	7.03897	1.34776	0.430563	1.34776
Atp1a1	NM_144900	0.007019	5.09264	9.95705	9.52657	1.34768	0.430479	1.34768
Lcmt1	NM_025304	0.000156	13.8901	7.25517	6.82566	1.34677	0.429507	1.34677
Usp47	NM_133758	0.00015	14.0237	10.0301	9.60107	1.34634	0.429044	1.34634
Rras	NM_009101	0.00037	11.1393	9.48749	9.05845	1.34634	0.429042	1.34634
Usp30	NM_001033202	0.0003	11.7533	7.53709	7.10848	1.34594	0.42861	1.34594
	---	0.00512	5.56097	4.16801	3.73944	1.3459	0.428575	1.3459
	---	0.000682	9.5103	9.1225	8.69488	1.34501	0.427615	1.34501
E2f3	NM_010093	0.005948	5.33439	7.10491	6.67738	1.34493	0.427526	1.34493
Adipor1	NM_028320	0.000618	9.75646	9.24931	8.82214	1.34459	0.42717	1.34459
Rnf20	NM_001163263	0.004843	5.64709	8.56001	8.13345	1.34402	0.426556	1.34402
Rai12	NM_018740	0.003365	6.23801	9.03535	8.60935	1.34351	0.426006	1.34351
Acd	NM_001012638	0.005146	5.55336	6.82473	6.39934	1.34293	0.425381	1.34293
Vps11	NM_027889	0.00423	5.86119	9.06924	8.64448	1.34235	0.42476	1.34235
Utp6	NM_144826	0.000852	8.97751	8.84631	8.42383	1.34023	0.422479	1.34023
	---	0.001927	7.24425	9.24664	8.82419	1.34021	0.422456	1.34021
1500002O20Rik	NM_028047	0.000315	11.6037	6.76438	6.34233	1.33982	0.422044	1.33982
Inppl1	NM_010567	8.18E-05	16.3542	5.24052	4.81853	1.33977	0.421985	1.33977
Lins	NM_152815	0.005325	5.50085	6.99221	6.57112	1.33893	0.421084	1.33893
Calm1	NM_009790	0.001447	7.81516	10.6882	10.2679	1.33818	0.420274	1.33818
Ppp1r12b	NM_001081307	0.000746	9.29137	6.84792	6.4277	1.33813	0.420218	1.33813
Notch2	NM_010928	0.000134	14.4332	9.19304	8.77308	1.33789	0.419957	1.33789
Flot2	NM_008028	0.001416	7.85919	7.62245	7.20268	1.33771	0.419771	1.33771
Tti1	NM_029282	0.005454	5.46464	7.66489	7.24537	1.33748	0.41952	1.33748
Plp2	NM_019755	0.006293	5.25097	10.8731	10.4538	1.33734	0.419368	1.33734
Vps4b	NM_009190	0.002151	7.03529	10.1005	9.68231	1.33627	0.418206	1.33627
Suv420h1	NM_001167885	0.001254	8.11488	8.45282	8.03543	1.3355	0.417381	1.3355
Dnttip2	NM_153806	0.005658	5.40898	7.52182	7.105	1.33499	0.416824	1.33499

Prdx3	NM_007452	0.000754	9.26647	10.2169	9.8005	1.33455	0.416359	1.33455
Ctbp2	NM_009980	0.002722	6.60586	7.98643	7.57092	1.33378	0.415517	1.33378
Insc	NM_173767	0.001457	7.80138	5.46415	5.04892	1.33351	0.415227	1.33351
Hdac10	NM_199198	0.003793	6.0382	7.22221	6.80789	1.33267	0.414316	1.33267
Zfand3	NM_148926	0.006183	5.27697	8.12174	7.70752	1.33258	0.414222	1.33258
Itch	NM_008395	0.005835	5.36302	9.4138	9.00168	1.33063	0.412113	1.33063
Glg1	NM_009149	0.000108	15.2492	10.4445	10.0324	1.33062	0.412102	1.33062
	---	0.00161	7.59796	2.99573	2.58399	1.33028	0.411734	1.33028
Mthfsd	NM_172761	0.005736	5.38843	7.35461	6.94312	1.33006	0.41149	1.33006
Xpnpep3	NM_177310	0.002979	6.44681	7.47776	7.06644	1.3299	0.411319	1.3299
Psma5	NM_011967	0.00422	5.86503	8.94953	8.53878	1.32938	0.410752	1.32938
Sgcb	NM_011890	0.005042	5.58466	9.29093	8.88126	1.32839	0.409674	1.32839
Dpp3	NM_133803	6.84E-05	17.1126	8.50822	8.09865	1.32829	0.409575	1.32829
Naf1	NM_001163564	0.003033	6.4159	6.95454	6.54585	1.32748	0.408693	1.32748
Pvrl3	NM_021496	0.004172	5.88336	7.48997	7.08176	1.32704	0.408213	1.32704
Mett11d1	NM_001029990	0.006909	5.11527	7.05149	6.64362	1.32673	0.407874	1.32673
Trpc4ap	NM_001163452	0.002773	6.57292	10.2664	9.85947	1.32583	0.406898	1.32583
	---	0.000408	10.8593	10.1994	9.79267	1.32568	0.406738	1.32568
	---	0.001787	7.39127	10.5257	10.1192	1.32545	0.406482	1.32545
Gm13034	NR_030771	0.003378	6.23167	7.92736	7.52093	1.3254	0.40643	1.3254
Slmo2	NM_025531	0.005472	5.45953	8.38104	7.9747	1.32532	0.406343	1.32532
	---	0.006019	5.31659	10.8524	10.4468	1.3247	0.405665	1.3247
Trmt112	NM_001166370	0.000985	8.64468	10.4977	10.0921	1.32464	0.405602	1.32464
Pycrl	NM_025412	0.00421	5.8688	8.28822	7.88272	1.32455	0.405504	1.32455
Ing5	NM_025454	0.004893	5.63097	7.46499	7.05958	1.32447	0.405412	1.32447
Trmt112	NM_001166370	0.001417	7.85825	10.4936	10.0889	1.32377	0.404647	1.32377
Alg14	NM_024178	0.004911	5.62547	7.89774	7.49354	1.32336	0.404208	1.32336
Nutf2	NM_026532	0.002894	6.49743	10.049	9.64487	1.32325	0.404086	1.32325
Nprl3	NM_181569	0.003735	6.06365	8.18025	7.77671	1.32275	0.403541	1.32275
Fbxo3	NM_212433	0.005173	5.54516	9.23835	8.83516	1.32243	0.403189	1.32243
Bzw1	NM_025824	0.004994	5.59943	11.3141	10.9111	1.32228	0.403025	1.32228

Cops3	NM_011991	0.003286	6.27814	9.02562	8.62355	1.3214	0.402071	1.3214
Agpat4	NM_026644	0.004222	5.86402	7.90696	7.5049	1.32139	0.40206	1.32139
Brpf3	NM_001081315	5.82E-05	17.8242	6.36679	5.96478	1.32135	0.40201	1.32135
1110018G07Rik	NM_178065	0.00296	6.45813	8.22759	7.82647	1.32053	0.401119	1.32053
Ifne	NM_177348	0.004443	5.78252	5.15175	4.75088	1.3203	0.40087	1.3203
Pacrgl	NM_025755	0.003148	6.3519	6.81354	6.41309	1.31992	0.400448	1.31992
Chd1	NM_007690	0.004705	5.69218	8.66455	8.26512	1.31899	0.399435	1.31899
Med9	NM_138675	0.002938	6.47149	6.98304	6.58395	1.31867	0.399087	1.31867
Poldip2	NM_026389	0.000818	9.07195	9.08278	8.68392	1.31847	0.398861	1.31847
Rag1ap1	NM_009057	0.00372	6.07034	9.97394	9.57539	1.31818	0.398544	1.31818
Ceacam2	NM_001113368	0.003725	6.06835	4.83964	4.44244	1.31695	0.397205	1.31695
Ccdc129	NM_001081665	0.000147	14.1083	3.95432	3.55739	1.3167	0.396928	1.3167
Tecpr1	NM_027410	0.003965	5.96569	7.54516	7.14877	1.3162	0.396382	1.3162
2210404J11Rik	NM_001039552	0.005511	5.44863	7.91551	7.5192	1.31614	0.396312	1.31614
Usp36	NM_001033528	0.000427	10.7315	7.47554	7.07934	1.31604	0.396208	1.31604
Ewsr1	NM_007968	0.002046	7.12999	6.65109	6.25539	1.31558	0.395702	1.31558
Lpxn	NM_134152	0.001332	7.98722	4.53736	4.14166	1.31558	0.395698	1.31558
Nt5c3l	NM_026561	0.001554	7.66952	8.13857	7.7429	1.31555	0.395668	1.31555
Acot10	NM_022816	0.00456	5.74136	5.66921	5.27396	1.31518	0.395255	1.31518
Alkbh6	NM_198027	0.003571	6.13803	6.75855	6.36334	1.31513	0.395207	1.31513
Lgtn	NM_001136070	0.000273	12.0371	6.89073	6.49554	1.31511	0.395187	1.31511
Cyp4b1	NM_007823	0.002298	6.912	4.98427	4.58919	1.31501	0.395077	1.31501
Ogg1	NM_010957	0.002421	6.81644	6.81523	6.4202	1.31497	0.395029	1.31497
Lias	NM_024471	0.002488	6.76733	8.18517	7.79057	1.31458	0.394605	1.31458
Tdg	NM_011561	0.004041	5.93495	7.92839	7.53383	1.31454	0.394561	1.31454
Pax2	NM_011037	0.002298	6.9124	5.65494	5.26108	1.3139	0.393855	1.3139
Usp5	NM_013700	0.002958	6.45924	9.24689	8.85309	1.31385	0.393802	1.31385
Scarna17	NR_028560	0.001192	8.22291	11.0699	10.6763	1.31366	0.393597	1.31366
Scarna17	NR_028560	0.001192	8.22291	11.0699	10.6763	1.31366	0.393597	1.31366
Tdg	NM_011561	0.002679	6.63415	8.00879	7.61523	1.31363	0.393558	1.31363
Tceb2	NM_026305	0.001651	7.54806	10.1407	9.74747	1.31337	0.393271	1.31337

Slc28a2	NM_172980	0.004297	5.83573	4.87162	4.47881	1.31295	0.392817	1.31295
Dvl2	NM_007888	0.003629	6.11136	7.89794	7.5052	1.31289	0.392742	1.31289
Klhl32	NM_001163020	0.003933	5.9789	4.36054	3.9699	1.31097	0.39064	1.31097
Helb	NM_080446	0.004638	5.71479	7.93987	7.54964	1.3106	0.390226	1.3106
Psmd8	NM_026545	0.004339	5.82045	9.31976	8.92962	1.31052	0.390137	1.31052
Stradb	NM_172656	0.001651	7.54791	7.07253	6.68278	1.31017	0.38975	1.31017
Nlrp4e	NM_001004194	0.002568	6.70951	3.66046	3.27072	1.31016	0.38974	1.31016
Snf8	NM_033568	0.004774	5.66944	7.92705	7.53734	1.31013	0.389715	1.31013
Nsfl1c	NM_198326	0.006265	5.2576	9.35445	8.9653	1.30963	0.389158	1.30963
	---	0.00062	9.75109	5.08675	4.69773	1.30951	0.389024	1.30951
	---	0.00062	9.75109	5.08675	4.69773	1.30951	0.389024	1.30951
Wdr45	NM_172372	0.004116	5.90517	8.94643	8.55775	1.3092	0.388686	1.3092
Sf3b3	NM_133953	0.007035	5.0893	10.49	10.1014	1.30914	0.388621	1.30914
Bat3	NM_057171	0.00048	10.4148	9.49049	9.10248	1.30859	0.388013	1.30859
Aktip	NM_010241	0.006067	5.30496	8.47859	8.09075	1.30843	0.38784	1.30843
Glyr1	NM_001079814	0.000181	13.3783	9.2831	8.89528	1.30841	0.387818	1.30841
	---	0.001078	8.44226	5.57919	5.19178	1.30804	0.387408	1.30804
Hnrnpul1	NM_144922	0.002253	6.94926	9.25138	8.86562	1.30655	0.385767	1.30655
Fbxw11	NM_134015	0.002702	6.61897	8.75956	8.37382	1.30653	0.385744	1.30653
Eif4h	NM_033561	0.005071	5.57569	10.7591	10.3746	1.30536	0.384443	1.30536
Nhp2l1	NM_011482	0.005745	5.38625	10.8817	10.498	1.30469	0.383707	1.30469
C330006K01Rik	NM_172725	0.002384	6.84488	6.88125	6.49815	1.30414	0.383096	1.30414
	---	0.007012	5.0939	10.3766	9.99377	1.30387	0.382799	1.30387
1700037H04Rik	BC071212	0.002693	6.62496	7.03789	6.65553	1.30347	0.382353	1.30347
4732418C07Rik	BC059213	0.000818	9.0736	7.56152	7.18003	1.30268	0.381488	1.30268
Stim1	NM_009287	0.005287	5.51168	7.58644	7.20618	1.30158	0.380262	1.30158
Olfr368	NM_146374	0.000904	8.84118	4.86517	4.48514	1.30137	0.380035	1.30137
Ppapdc2	NM_028922	0.000182	13.3418	6.81201	6.43198	1.30137	0.380032	1.30137
Pofut1	NM_080463	0.005353	5.49299	7.13961	6.75981	1.30116	0.379798	1.30116
Brf1	NM_028193	0.002301	6.90981	8.07867	7.70041	1.29977	0.378261	1.29977
Znrf2	NM_199143	0.004276	5.84389	8.12868	7.75073	1.2995	0.377958	1.2995

	---	0.005713	5.39453	2.72348	2.3457	1.29934	0.377775	1.29934
Psma1	NM_011965	0.000115	14.9897	9.68115	9.30346	1.29926	0.377694	1.29926
Ube2i	NM_011665	0.001414	7.86204	10.5488	10.1719	1.2986	0.376954	1.2986
Trim41	NM_145377	0.001048	8.50594	8.39756	8.02123	1.29803	0.376326	1.29803
Ube2m	NM_145578	0.003539	6.15325	8.97129	8.59563	1.29743	0.375662	1.29743
Kif3c	NM_008445	0.007011	5.09418	6.71014	6.33484	1.29711	0.375299	1.29711
6330577E15Rik	NM_026377	0.003821	6.02639	9.5082	9.13332	1.29673	0.374883	1.29673
	---	0.004386	5.80306	9.97933	9.60474	1.29647	0.374587	1.29647
Gm6682	NR_033599	0.004256	5.8512	9.32129	8.94679	1.29639	0.374498	1.29639
D11Wsu99e	NM_138598	0.004795	5.6625	7.76628	7.3919	1.29628	0.374378	1.29628
Eif4a3	NM_138669	0.000307	11.6823	9.13765	8.76353	1.29605	0.374123	1.29605
Psme2	NM_011190	0.006377	5.23159	8.93858	8.56522	1.29536	0.373358	1.29536
Nutf2	NM_026532	0.004441	5.78323	10.0298	9.65711	1.29475	0.372677	1.29475
Nutf2	NM_026532	0.004441	5.78323	10.0298	9.65711	1.29475	0.372677	1.29475
	---	0.003149	6.35106	11.0293	10.6574	1.29411	0.37196	1.29411
Ccdc109b	NM_025779	0.0009	8.8497	6.35749	5.98558	1.29407	0.371912	1.29407
Plagl2	NM_018807	0.001754	7.42765	6.55183	6.18042	1.29362	0.371416	1.29362
Abi2	NM_001198571	0.00162	7.58604	7.55206	7.18177	1.29261	0.370288	1.29261
	---	0.001007	8.59422	11.4534	11.0832	1.29252	0.370185	1.29252
Tm9sf3	NM_133352	0.000641	9.66597	10.9614	10.5919	1.29193	0.36953	1.29193
Whamm	NM_001004185	0.006111	5.29423	6.83165	6.46245	1.29163	0.369198	1.29163
Creb1	NM_133828	0.005872	5.3535	8.60323	8.23441	1.2913	0.368826	1.2913
Eif3a	NM_010123	0.001047	8.50767	11.1161	10.7492	1.28954	0.366858	1.28954
Lsm12	NM_172947	0.004004	5.94962	10.3255	9.95923	1.28899	0.366244	1.28899
	---	0.005021	5.59108	9.83942	9.47525	1.28715	0.364175	1.28715
Fkbp1a	NM_008019	0.002357	6.86593	8.96963	8.60565	1.28697	0.363975	1.28697
Tmem120b	NM_001039723	0.001646	7.55343	6.9067	6.54305	1.28668	0.363653	1.28668
Erich1	NM_001034862	0.005381	5.48505	6.8562	6.4932	1.2861	0.363005	1.2861
Mapk1	NM_011949	0.001402	7.88038	10.1386	9.77612	1.28563	0.362472	1.28563
Hist1h2ba	NM_175663	0.000898	8.85481	4.72151	4.3596	1.28512	0.361909	1.28512
	---	0.00064	9.67027	10.1014	9.74072	1.28405	0.360703	1.28405

Mrpl2	NM_025302	0.001903	7.26834	8.57199	8.21188	1.28353	0.360117	1.28353
Nhp2l1	NM_011482	0.006298	5.24976	10.8801	10.52	1.28347	0.360045	1.28347
Rfng	NM_009053	0.000593	9.86122	7.07235	6.71336	1.28252	0.358986	1.28252
Ncoa5	NM_144892	0.000191	13.1829	8.11193	7.75347	1.28205	0.358455	1.28205
Gm12070	NR_002890	0.000782	9.17955	12.2033	11.8451	1.2818	0.35817	1.2818
	---	0.001796	7.38078	3.49105	3.13293	1.28175	0.358119	1.28175
Mtap1s	NM_173013	1.97E-06	41.7264	7.4054	7.04761	1.28146	0.357793	1.28146
	---	0.001082	8.43499	10.1887	9.83099	1.28135	0.35766	1.28135
Pias4	NM_021501	0.005109	5.56437	7.95339	7.59575	1.28132	0.357636	1.28132
Zfr	NM_011767	0.002662	6.64505	9.64052	9.28405	1.28029	0.35647	1.28029
Tgfbrap1	NM_001013025	0.000935	8.7618	6.86727	6.51098	1.28013	0.356289	1.28013
Dnajc14	NM_028873	9.78E-05	15.6302	9.19575	8.83974	1.27989	0.356015	1.27989
C030006K11Rik	NM_145472	0.000465	10.499	7.2763	6.92117	1.2791	0.355134	1.2791
Ftsjd2	NM_028791	0.004651	5.71023	9.11833	8.76552	1.27704	0.352808	1.27704
Nhlrc2	NM_025811	0.004222	5.86422	8.28695	7.93424	1.27696	0.352711	1.27696
	---	0.003698	6.08022	3.02395	2.67127	1.27693	0.352685	1.27693
1810030O07Rik	BC137660	0.001137	8.32615	8.80991	8.45734	1.27684	0.352575	1.27684
	---	0.00442	5.7908	11.4714	11.1189	1.27679	0.352516	1.27679
Oaz1	NM_008753	1.75E-06	42.9979	11.4789	11.1276	1.27571	0.351304	1.27571
Smarca5	NM_053124	0.003432	6.20483	9.48609	9.13577	1.27484	0.350319	1.27484
Rrp7a	NM_029101	0.002148	7.03845	8.40466	8.0549	1.27435	0.349757	1.27435
Eftud1	NM_175317	0.00023	12.5833	8.06673	7.71776	1.27365	0.348967	1.27365
Eif4a3	NM_138669	3.68E-05	20.0135	9.28233	8.9364	1.27097	0.345929	1.27097
Nupl1	NM_170591	0.003378	6.23146	8.43468	8.08929	1.27049	0.345382	1.27049
Smg7	NM_001160257	0.004026	5.94083	7.90907	7.56375	1.27043	0.345314	1.27043
Sdcbp2	NM_145535	3.79E-05	19.8599	5.88069	5.5355	1.27032	0.345189	1.27032
Chchd2	NM_024166	0.004494	5.76461	10.7837	10.4402	1.2689	0.343578	1.2689
Hnf1a	ENSMUST0000003153 5	0.001119	8.36003	8.11406	7.77056	1.26883	0.343501	1.26883
	---	0.000971	8.67627	4.01239	3.66918	1.26857	0.343204	1.26857
2310003H01Rik	NM_027980	0.000425	10.7448	6.41726	6.07423	1.26842	0.343032	1.26842

Gm5965	ENSMUST00000009191	0.005031	5.58794	4.12288	3.78021	1.26811	0.342677	1.26811
Taf10	NM_020024	0.001893	7.27883	6.23153	5.88952	1.26752	0.34201	1.26752
Agap3	NM_139153	0.00192	7.25166	7.15404	6.81286	1.2668	0.341186	1.2668
	---	0.004173	5.88286	5.09223	4.75149	1.26641	0.340741	1.26641
Mett10d	NM_026197	0.004175	5.8822	7.96561	7.6252	1.26612	0.340408	1.26612
Map3k10	NM_001081292	0.002141	7.04381	6.45483	6.11499	1.26561	0.339837	1.26561
Mad1l1	NM_010752	0.001502	7.73818	6.93482	6.59545	1.2652	0.339364	1.2652
Nol9	NM_028727	0.003975	5.96146	8.42889	8.08967	1.26507	0.339221	1.26507
Hadha	NM_178878	0.00708	5.08006	9.55085	9.21248	1.26433	0.33837	1.26433
Sf3b1	NM_031179	0.003653	6.10025	10.9683	10.6311	1.26324	0.337126	1.26324
Rrp1	NM_010925	0.002144	7.042	9.94504	9.60898	1.2623	0.336053	1.2623
Trim26	NM_001025599	6.29E-05	17.4787	6.60331	6.26751	1.26208	0.335805	1.26208
Gm5072	NM_001114678	0.00394	5.9758	3.65244	3.31823	1.26069	0.33421	1.26069
Zdhhc6	NM_001033573	9.41E-05	15.7841	8.60614	8.27246	1.26023	0.333688	1.26023
Ctnnbl1	NM_025680	0.001942	7.22976	8.15111	7.81754	1.26013	0.333571	1.26013
Anapc4	NM_024213	0.002332	6.88544	9.55722	9.22367	1.26011	0.33355	1.26011
Hspa4	NM_008300	0.004325	5.82541	9.49686	9.1634	1.26003	0.333457	1.26003
Ccl25	NR_033527	0.006017	5.31728	6.24753	5.91421	1.25991	0.333323	1.25991
Lce1h	NM_026335	0.00355	6.14819	5.82335	5.49004	1.2599	0.333307	1.2599
Agl	NM_001081326	0.005928	5.33929	7.82182	7.48997	1.25863	0.331851	1.25863
Mrps18b	NM_025878	0.003017	6.42496	9.15748	8.82585	1.25844	0.331634	1.25844
Oaz1	NM_008753	0.000343	11.357	11.1831	10.8521	1.25785	0.330959	1.25785
Chchd2	NM_024166	0.003021	6.42274	11.2865	10.957	1.25659	0.329519	1.25659
Atg9b	NM_001002897	7.33E-05	16.8181	5.94634	5.61684	1.25658	0.329499	1.25658
Slc4a1ap	NM_009206	0.001407	7.87341	7.4111	7.08173	1.25646	0.329366	1.25646
Polr2c	NM_009090	0.000774	9.20538	8.66063	8.33224	1.25561	0.328384	1.25561
Gapdh	GU214026	0.000941	8.74833	12.0959	11.7687	1.25458	0.32721	1.25458
Gm5072	NM_001114678	0.001454	7.80437	4.03068	3.70424	1.25392	0.326446	1.25392
Rbm42	NM_133693	0.003552	6.14709	7.92135	7.59597	1.25299	0.325379	1.25299
Ppia	NM_008907	0.003823	6.02542	12.393	12.0679	1.25276	0.32511	1.25276

Atpaf2	NM_145427	0.000735	9.32767	7.63804	7.31335	1.25239	0.324687	1.25239
Mki67ip	NM_026472	0.000361	11.2093	8.74782	8.42377	1.25184	0.324049	1.25184
Dhx15	NM_007839	0.006343	5.23941	10.6987	10.3764	1.25034	0.322315	1.25034
Prkcsh	NM_008925	0.00269	6.62639	9.8035	9.48119	1.25033	0.32231	1.25033
Chchd2	NM_024166	0.004102	5.91061	11.0829	10.762	1.24912	0.320917	1.24912
Rnh1	NM_001172101	0.001071	8.45733	9.53836	9.21851	1.2482	0.319852	1.2482
	---	0.002431	6.8093	8.06028	7.74217	1.2467	0.318109	1.2467
Tomm34	NM_025996	0.004171	5.88362	8.75829	8.44047	1.24645	0.317821	1.24645
Ankrd46	NM_175134	0.006374	5.23225	8.99774	8.68185	1.24478	0.315887	1.24478
Casp6	NM_009811	0.003949	5.97224	7.50474	7.18916	1.24451	0.315576	1.24451
Megf8	NM_001160400	0.00402	5.94335	7.70621	7.39106	1.24415	0.315156	1.24415
Cops6	NM_012002	0.006259	5.25894	9.64487	9.3302	1.24372	0.314664	1.24372
Pomp	NM_025624	0.006995	5.0975	10.4036	10.0896	1.24313	0.313975	1.24313
	---	0.005417	5.47484	4.36769	4.05416	1.24274	0.31353	1.24274
Atxn2	NM_009125	0.001634	7.56787	8.54231	8.22918	1.2424	0.313135	1.2424
	---	0.0014	7.88261	3.24416	2.93121	1.24225	0.312951	1.24225
Rnf213	ENSMUST00000131035	0.004836	5.64945	7.28798	6.97605	1.24137	0.311928	1.24137
Mfsd1	NM_025813	0.001438	7.82789	10.6825	10.3731	1.23915	0.309351	1.23915
Chd8	NM_201637	0.001828	7.34632	8.26606	7.95774	1.23826	0.308317	1.23826
Cspp1	NM_026493	0.006423	5.22099	7.27843	6.97086	1.23762	0.307564	1.23762
Scaf1	NM_001008422	0.005807	5.37004	7.37697	7.07	1.2371	0.306965	1.2371
Cpt1b	NM_009948	0.004397	5.79914	5.68374	5.37724	1.2367	0.306498	1.2367
	---	0.00073	9.34436	3.24545	2.93908	1.2366	0.306377	1.2366
Coro1b	NM_011778	0.004087	5.9166	10.4326	10.1276	1.23546	0.305047	1.23546
	---	0.004829	5.6517	10.2606	9.95632	1.23484	0.30432	1.23484
Rpl13a	NM_009438	0.001519	7.71611	12.5718	12.2685	1.23395	0.303288	1.23395
Edc4	NM_181594	0.00391	5.9886	7.99511	7.69227	1.23357	0.302845	1.23357
Nit1	NR_033728	0.003258	6.29276	7.84955	7.54877	1.23181	0.300783	1.23181
Zfp106	NM_011743	0.002459	6.78856	8.29844	7.99821	1.23133	0.300221	1.23133
Rnf168	NM_027355	0.000863	8.94863	7.74195	7.44288	1.23035	0.299074	1.23035

Zbtb2	NM_001033466	0.001007	8.59493	6.7117	6.41271	1.23028	0.298983	1.23028
Mrps15	NM_025544	0.007005	5.09543	9.15115	8.85248	1.23001	0.298671	1.23001
Tdg	NM_172552	0.000869	8.93107	7.89041	7.59272	1.22918	0.297692	1.22918
Vps37a	NM_033560	0.000335	11.4259	7.53485	7.23885	1.22773	0.295999	1.22773
Rbm33	NM_028234	9.78E-05	15.6325	7.61128	7.3171	1.22618	0.294172	1.22618
Ctu1	NM_145582	0.006066	5.30519	6.22464	5.93054	1.22612	0.2941	1.22612
Ppp5c	NM_011155	0.002472	6.77867	9.62743	9.33359	1.2259	0.293838	1.2259
Vmn2r-ps14	NR_002888	0.002426	6.81276	9.6729	9.38031	1.22485	0.2926	1.22485
	---	0.004895	5.63044	5.17686	4.88481	1.22438	0.292054	1.22438
Dctn3	NM_016890	0.002648	6.65494	8.02984	7.73988	1.2226	0.289957	1.2226
Gapdh	NM_008084	0.002912	6.4866	12.9568	12.668	1.22161	0.288789	1.22161
Aspscr1	NM_026877	0.005122	5.56028	8.22163	7.93317	1.22134	0.288464	1.22134
Tyw1	NM_001015876	0.006642	5.17227	7.52045	7.23233	1.22104	0.288116	1.22104
Sumo2	NM_133354	0.005027	5.58924	10.7293	10.4457	1.21724	0.283615	1.21724
	---	0.000746	9.2941	9.98884	9.70569	1.21685	0.283153	1.21685
R3hdm1	NM_181750	0.003384	6.22874	7.55425	7.27166	1.21637	0.282584	1.21637
Sh2b1	NM_011363	0.002302	6.90899	7.47434	7.19278	1.21551	0.281561	1.21551
Gm12070	NR_002890	0.006098	5.29726	12.863	12.582	1.21507	0.281043	1.21507
Spats1	NM_027649	0.001265	8.09594	4.19433	3.91338	1.215	0.280951	1.215
Mlf2	NM_145385	0.000213	12.8205	10.0719	9.79237	1.21383	0.279566	1.21383
	---	0.004923	5.62159	2.90862	2.62953	1.21343	0.279091	1.21343
Fau	NM_007990	0.006302	5.2489	10.878	10.6012	1.21147	0.276758	1.21147
BC067068	NM_207522	0.002899	6.49463	5.87714	5.60099	1.21096	0.276154	1.21096
Eif4g2	NM_013507	0.004357	5.81378	11.4551	11.18	1.21007	0.275089	1.21007
AW209491	NM_134067	0.005719	5.39294	6.52754	6.25264	1.20991	0.274899	1.20991
Abce1	NM_015751	0.001328	7.99356	10.413	10.1388	1.20933	0.274213	1.20933
Dvl3	NM_007889	0.006873	5.12282	7.22317	6.94954	1.20884	0.273629	1.20884
Cdc73	NM_145991	0.003508	6.16809	8.77441	8.50204	1.20779	0.272369	1.20779
D19Wsu162e	NM_001177812	0.005085	5.57149	9.30656	9.03461	1.20745	0.271958	1.20745
Mast4	NM_175171	0.00391	5.98858	7.1621	6.89047	1.20717	0.271631	1.20717
Qars	NM_133794	0.005824	5.36565	9.88122	9.60964	1.20713	0.271576	1.20713

Ctbp1	NM_013502	0.000684	9.50336	9.51591	9.24807	1.20401	0.267844	1.20401
Eif4e	NM_007917	0.002884	6.50345	10.2285	9.96267	1.20229	0.265786	1.20229
Zfp507	NM_177739	0.001437	7.82988	8.1631	7.90276	1.19776	0.260335	1.19776
	---	0.006174	5.27896	3.72651	3.46694	1.19712	0.259573	1.19712
	---	0.004116	5.90504	6.84686	6.58801	1.19652	0.258848	1.19652
Serpinb7	NM_027548	0.00057	9.96473	2.76385	2.50618	1.19555	0.257679	1.19555
AI894139	NM_178898	0.000411	10.8405	5.52249	5.26685	1.19386	0.255638	1.19386
Brd8	NM_030147	0.004651	5.71031	7.79185	7.53626	1.19383	0.255593	1.19383
Fam168a	BC079886	0.000565	9.98737	7.449	7.19484	1.19264	0.254159	1.19264
Tmco7	NM_173037	0.001563	7.65811	6.53026	6.27625	1.19252	0.254015	1.19252
Gm13637	XM_911155	6.87E-05	17.093	4.81622	4.56381	1.1912	0.25241	1.1912
Nudt9	NM_028794	0.004604	5.72642	8.77828	8.52631	1.19084	0.251976	1.19084
Rpl41	NM_018860	0.005714	5.39415	11.42	11.1681	1.19079	0.251919	1.19079
Rpl41	NM_018860	0.005714	5.39415	11.42	11.1681	1.19079	0.251919	1.19079
Rpl41	NM_018860	0.005714	5.39415	11.42	11.1681	1.19079	0.251919	1.19079
Sufu	NM_015752	0.000665	9.57407	7.07786	6.82678	1.1901	0.251081	1.1901
Srsf10	NM_001080387	0.004131	5.89905	9.96163	9.71091	1.1898	0.250724	1.1898
	---	0.000412	10.8297	3.47758	3.22746	1.18931	0.25012	1.18931
Rnf41	NM_001164237	0.004658	5.70789	7.90005	7.65073	1.18864	0.249313	1.18864
Sepn1	NM_029100	0.000531	10.1457	7.22454	6.97555	1.18837	0.24899	1.18837
Mfap3	NM_145426	0.005419	5.47415	8.26271	8.01488	1.18742	0.247825	1.18742
Srsf11	NM_001093752	0.000455	10.5589	8.90873	8.66216	1.18638	0.246563	1.18638
BC002230	NM_183155	0.005253	5.52175	7.06031	6.81551	1.18494	0.24481	1.18494
Gm10046	NR_033484	0.006919	5.11306	5.85614	5.6156	1.18143	0.240538	1.18143
Ube2d3	NM_025356	0.000999	8.61312	11.6271	11.3872	1.18086	0.239832	1.18086
Olfr1034	NM_001011872	0.001887	7.28498	3.15799	2.91916	1.18003	0.238829	1.18003
Glra1	NM_020492	0.003948	5.97268	3.78758	3.5493	1.17958	0.238273	1.17958
Dld	NM_007861	0.005647	5.41211	9.45519	9.21711	1.17942	0.238078	1.17942
	---	0.005495	5.45321	2.57443	2.33637	1.1794	0.238054	1.1794
Rnf4	NM_011278	0.003257	6.29338	10.5499	10.3156	1.17633	0.234299	1.17633
Ces2b	NM_198171	0.005502	5.45134	4.07172	3.83756	1.17622	0.234159	1.17622

	---	0.003551	6.14741	3.35279	3.1214	1.17396	0.231384	1.17396
	---	0.005821	5.36642	5.82903	5.59931	1.1726	0.229714	1.1726
	---	0.002294	6.91566	5.85052	5.62208	1.17157	0.228439	1.17157
	---	0.006852	5.12717	2.94339	2.71544	1.17117	0.227954	1.17117
Clns1a	NM_023671	0.006735	5.15213	8.31579	8.09367	1.16645	0.222123	1.16645
	---	0.004206	5.87028	5.84773	5.62577	1.16632	0.221963	1.16632
	---	0.002482	6.77128	5.82591	5.60418	1.16614	0.221735	1.16614
	---	0.002482	6.77128	5.82591	5.60418	1.16614	0.221735	1.16614
	---	0.002482	6.77128	5.82591	5.60418	1.16614	0.221735	1.16614
	---	0.002482	6.77128	5.82591	5.60418	1.16614	0.221735	1.16614
Mapk1ip1	NM_001045483	0.007001	5.09615	6.61269	6.39514	1.16276	0.217551	1.16276
Raly	NM_001139513	0.002897	6.49551	9.46514	9.24811	1.16234	0.217037	1.16234
E4f1	NM_007893	0.004858	5.64241	7.8338	7.61694	1.1622	0.216861	1.1622
Slc15a5	NM_177787	0.003967	5.96493	4.59709	4.38268	1.16023	0.214411	1.16023
Nop14	NM_029278	0.000478	10.4251	9.0134	8.80423	1.15602	0.209171	1.15602
Slc10a7	NM_029736	0.006053	5.30843	7.42683	7.21779	1.15592	0.209038	1.15592
Tmem49	NM_029478	0.002166	7.02243	10.2515	10.0464	1.15276	0.205088	1.15276
Gm5177	NR_033630	0.000591	9.87039	11.8775	11.6753	1.15044	0.202189	1.15044
Gm8994	NM_001142734	0.004334	5.82225	5.50843	5.30913	1.14814	0.199298	1.14814
	---	0.001782	7.39655	2.81325	2.62092	1.1426	0.192325	1.1426
Flad1	NM_177041	0.005698	5.39856	6.24518	6.05447	1.14133	0.190711	1.14133
Cttnbp2nl	NM_030249	0.006833	5.13122	7.29713	7.11024	1.13831	0.18689	1.13831
1700024P16Rik	NM_001162980	0.00062	9.75004	3.54951	3.36512	1.13634	0.184395	1.13634
Rnf103	NM_009543	0.003861	6.00895	8.07305	7.88895	1.13611	0.184103	1.13611
Wdr18	NM_175450	0.006606	5.18002	8.05832	7.87861	1.13265	0.179708	1.13265
Ube2d3	NM_025356	0.004364	5.81111	11.4565	11.2778	1.13181	0.178632	1.13181
Amfr	NM_011787	0.004289	5.83882	4.86822	4.69709	1.12594	0.171131	1.12594
Phtf1	NM_013629	0.005011	5.59407	7.192	7.02564	1.12222	0.166357	1.12222
	---	0.002793	6.56	5.4237	5.26298	1.11785	0.160723	1.11785
Rnf187	NM_022423	0.004449	5.78051	8.8041	8.64468	1.11684	0.159422	1.11684
	---	0.000748	9.28668	4.4568	4.29838	1.11607	0.158426	1.11607

Olfr723	NM_001011530	0.005359	5.49123	3.58017	3.42488	1.11365	0.15529	1.11365
Mageb18	NM_173783	0.003796	6.03714	3.09643	2.9438	1.11159	0.152624	1.11159
	---	0.002963	6.45628	2.7949	2.6436	1.11057	0.151299	1.11057
Zfp397	NM_027007	0.006467	5.21097	7.743	7.59502	1.10802	0.147981	1.10802
Bloc1s3	NM_177692	0.002381	6.84745	7.90172	7.75639	1.10599	0.145332	1.10599
Slc25a34	NM_001013780	0.002215	6.98103	5.11254	4.97889	1.09706	0.133643	1.09706
4921521F21Rik	BC051128	0.000737	9.32099	3.53419	3.40514	1.09357	0.129043	1.09357
Llgl1	NM_008502	0.005481	5.45708	7.38207	7.25949	1.08869	0.122589	1.08869
Immp1l	NM_028260	0.004161	5.88756	4.1676	4.04503	1.08868	0.122576	1.08868
Hephl1	NM_001164797	0.003144	6.35409	3.38444	3.2688	1.08345	0.115638	1.08345
Adam3	NM_009619	0.002053	7.12374	3.41739	3.30736	1.07925	0.110031	1.07925
Gypa	NM_010369	0.005716	5.3938	2.84613	2.73945	1.07675	0.106687	1.07675
	---	0.004695	5.69571	4.51181	4.41516	1.06929	0.09665	1.06929
Olfr1257	NM_146982	0.005505	5.4503	2.92327	2.82831	1.06804	0.09496	1.06804
Mir106a	NR_029657	0.004678	-5.70142	2.65739	2.74192	0.943089	-0.08453	-1.06035
Sdccag3	NM_026563	0.004927	-5.62031	7.65128	7.73847	0.941357	-0.08719	-1.0623
Spam1	NM_001079875	0.003636	-6.10804	2.65983	2.74903	0.940043	-0.0892	-1.06378
Olfr994	NM_146433	0.006914	-5.11427	2.6584	2.74832	0.939571	-0.08993	-1.06432
	---	0.002157	-7.02981	2.8058	2.90289	0.934914	-0.0971	-1.06962
	---	0.005722	-5.39216	2.95877	3.05897	0.932907	-0.1002	-1.07192
8030411F24Rik	NM_030135	0.001362	-7.94123	3.26259	3.36738	0.929941	-0.10479	-1.07534
Fam32a	NM_026455	0.00505	-5.58232	8.12597	8.2533	0.915526	-0.12733	-1.09227
Galntl6	NM_175032	0.005876	-5.35257	3.90037	4.03175	0.912958	-0.13138	-1.09534
Ak4	NM_001177602	0.00019	-13.1999	6.03268	6.16619	0.911609	-0.13351	-1.09696
Wfikkn2	NM_181819	0.004844	-5.64662	4.02248	4.16576	0.905454	-0.14329	-1.10442
Olfr376	NM_001172686	0.000791	-9.15134	3.15902	3.30414	0.904301	-0.14513	-1.10583
Ube2d2	NM_019912	0.001833	-7.34118	9.19007	9.33638	0.903556	-0.14631	-1.10674
Tbc1d9b	NM_029745	0.003081	-6.38846	7.94346	8.09341	0.901281	-0.14995	-1.10953
Isy1	NM_133934	0.005285	-5.51241	7.83386	7.98529	0.900355	-0.15144	-1.11067
	---	0.002278	-6.92826	3.76713	3.92322	0.897453	-0.15609	-1.11426
Cyp4f39	NM_177307	0.002142	-7.04291	3.7743	3.93112	0.897	-0.15682	-1.11483

Zfp408	NM_001033451	0.003618	-6.11668	6.15439	6.31234	0.896297	-0.15795	-1.1157
Gm459	ENSMUST00000103337	0.003203	-6.32186	4.45223	4.61093	0.895835	-0.1587	-1.11628
Olfr1219	NM_146899	0.004079	-5.91972	3.01043	3.16948	0.895616	-0.15905	-1.11655
Gm5820	NM_001033789	0.000179	-13.4059	3.92534	4.08545	0.894956	-0.16011	-1.11737
Olfr1509	NM_020514	0.005122	-5.56029	2.77962	2.94253	0.893222	-0.16291	-1.11954
	---	0.005075	-5.57455	2.49032	2.67187	0.881757	-0.18155	-1.1341
Olfr1066	NM_001011735	0.002953	-6.46251	2.50045	2.68553	0.879598	-0.18508	-1.13688
Tmem18	NM_172049	0.00077	-9.21728	8.14395	8.33045	0.878738	-0.1865	-1.138
	---	0.001461	-7.79454	6.62123	6.80962	0.87758	-0.1884	-1.1395
Atp9b	NM_015805	0.000158	-13.8499	7.89204	8.08121	0.87711	-0.18917	-1.14011
	---	0.001629	-7.57403	3.46656	3.65607	0.876905	-0.18951	-1.14038
	---	0.004191	-5.87583	3.33575	3.52532	0.876868	-0.18957	-1.14042
Mir379	NR_029880	0.001109	-8.3809	2.62965	2.82432	0.873772	-0.19467	-1.14446
	---	0.001914	-7.25714	2.56822	2.76305	0.873674	-0.19483	-1.14459
Naa35	NM_030153	0.004183	-5.87908	8.74231	8.94168	0.87093	-0.19937	-1.1482
	---	0.003111	-6.37184	4.94273	5.14287	0.870466	-0.20014	-1.14881
Cox16	NM_025461	0.003429	-6.20648	6.13772	6.33845	0.87011	-0.20073	-1.14928
	---	0.002521	-6.74285	3.29222	3.49634	0.868072	-0.20411	-1.15198
Tal1	NM_011527	0.004487	-5.76688	5.4998	5.70393	0.868059	-0.20414	-1.152
Ndfip2	NM_029561	0.003594	-6.12767	8.00882	8.21344	0.867766	-0.20462	-1.15238
	---	0.002487	-6.76802	7.54181	7.74645	0.867753	-0.20464	-1.1524
Nek9	NM_145138	0.007094	-5.0774	8.80371	9.00953	0.867048	-0.20582	-1.15334
Tnfsf4	NM_009452	0.000797	-9.13557	3.43005	3.63754	0.866041	-0.20749	-1.15468
Ppm1a	NM_008910	0.00502	-5.59129	8.61893	8.82711	0.865627	-0.20818	-1.15523
Hbxip	NM_026774	0.003735	-6.06383	7.73191	7.9435	0.863584	-0.21159	-1.15797
Trappc2	NM_025432	0.006608	-5.17959	7.95413	8.16615	0.863329	-0.21202	-1.15831
Atg4a	NM_174875	0.006447	-5.21563	7.5565	7.76974	0.862597	-0.21324	-1.15929
Tmem156	ENSMUST00000043352	0.000462	-10.5168	2.94589	3.16231	0.860698	-0.21642	-1.16185
	---	0.005656	-5.40958	5.36884	5.58801	0.85906	-0.21917	-1.16406
9430053O09Rik	AK140789	0.006114	-5.29358	3.16526	3.38617	0.85802	-0.22092	-1.16547

Ttc17	NM_183106	0.000799	-9.12963	7.74866	7.96965	0.857979	-0.22099	-1.16553
	---	0.00621	-5.2704	2.55869	2.7799	0.857846	-0.22121	-1.16571
Rps2	NM_008503	0.006978	-5.10086	12.5853	12.807	0.857534	-0.22174	-1.16614
	---	0.000431	-10.7112	5.08821	5.31003	0.857488	-0.22181	-1.1662
Morf4l1	NM_001039147	0.005906	-5.34474	11.0763	11.2987	0.857146	-0.22239	-1.16666
Tmem29	NM_001164684	0.000585	-9.89538	4.15064	4.37432	0.856382	-0.22367	-1.1677
Gm10445	ENSMUST00000101229	0.005279	-5.51405	4.18342	4.41328	0.852718	-0.22986	-1.17272
Slc17a4	NM_177016	0.001251	-8.11959	3.58005	3.8131	0.850833	-0.23305	-1.17532
	---	0.002203	-6.99042	12.8278	13.0619	0.850205	-0.23412	-1.17619
Tysnd1	NM_027912	0.001924	-7.24761	7.49788	7.73229	0.85003	-0.23441	-1.17643
10-Mar	NM_172568	0.002826	-6.53931	3.74095	3.97695	0.849096	-0.236	-1.17772
Olfr472	NM_146774	0.001088	-8.42276	2.28264	2.51964	0.848508	-0.237	-1.17854
P2rx5	NM_033321	0.002002	-7.17084	4.99044	5.22766	0.848378	-0.23722	-1.17872
Supv3l1	NM_181423	0.00037	-11.1351	6.769	7.00991	0.846209	-0.24091	-1.18174
Fbxl3	NM_015822	0.006197	-5.27356	8.3301	8.57338	0.844825	-0.24328	-1.18368
Gnpda1	NM_011937	0.000583	-9.90476	7.2499	7.49388	0.844414	-0.24398	-1.18425
Wdr34	NM_001008498	0.004419	-5.79121	7.45822	7.70245	0.844266	-0.24423	-1.18446
Klhl20	NM_001039482	0.003397	-6.22227	7.35477	7.59932	0.844081	-0.24455	-1.18472
Stac	NM_016853	0.006722	-5.15491	4.00346	4.24816	0.843991	-0.2447	-1.18485
	---	0.003964	-5.96594	3.71677	3.9619	0.843741	-0.24513	-1.1852
4930408F14Rik	BC117727	0.002884	-6.50352	5.92565	6.1709	0.843668	-0.24525	-1.1853
Mcm3ap	NM_019434	0.005967	-5.32953	6.79789	7.04331	0.843566	-0.24543	-1.18544
Timm13	NM_013895	8.69E-05	-16.1092	7.86914	8.11662	0.842365	-0.24748	-1.18713
Pdcd6	NM_011051	8.03E-05	-16.4309	8.0319	8.28384	0.839768	-0.25194	-1.19081
	---	0.002334	-6.88334	5.42828	5.68111	0.839247	-0.25283	-1.19154
Csnk1a1	NM_146087	0.004634	-5.71625	10.2966	10.5515	0.838044	-0.2549	-1.19326
Nckap1	NM_016965	0.005061	-5.57892	10.0416	10.2982	0.837053	-0.25661	-1.19467
4930403L05Rik	NR_033171	0.002153	-7.03386	4.86537	5.1226	0.83669	-0.25723	-1.19519
Gm626	XM_985917	0.001269	-8.09019	3.58112	3.83956	0.835989	-0.25844	-1.19619
Atp5o	NM_138597	0.000993	-8.6268	8.49484	8.75585	0.834503	-0.26101	-1.19832

Ucn	NM_021290	0.002561	-6.71467	5.39945	5.66055	0.834447	-0.26111	-1.1984
Zfp592	NM_178707	0.002212	-6.98287	7.19355	7.45489	0.834316	-0.26133	-1.19859
Cacnb4	NM_001037099	0.004217	-5.86604	3.93011	4.19249	0.833717	-0.26237	-1.19945
Gm10880	ENSMUST0000010334 4	0.001966	-7.20581	3.65211	3.91631	0.832658	-0.2642	-1.20097
Mir98	NR_029753	0.003725	-6.068	3.59906	3.86492	0.831697	-0.26587	-1.20236
Cdk16	NM_011049	0.003751	-6.05675	7.96607	8.23365	0.830711	-0.26758	-1.20379
Ysk4	NM_011737	5.38E-05	-18.1828	3.46388	3.73221	0.830281	-0.26833	-1.20441
Psmd9	NM_026000	0.005174	-5.5449	7.73981	8.00837	0.830148	-0.26856	-1.2046
	---	0.005735	-5.38883	2.29338	2.5624	0.829884	-0.26902	-1.20499
Stx4a	NM_009294	0.004898	-5.62964	8.38167	8.6507	0.829876	-0.26903	-1.205
Hmcn1	NM_001024720	0.005197	-5.53823	2.82991	3.10019	0.829162	-0.27028	-1.20604
Cdc23	NM_178347	0.00079	-9.15701	8.50266	8.77675	0.826971	-0.27409	-1.20923
Kdelr1	NM_133950	0.003658	-6.0984	8.94737	9.22192	0.826703	-0.27456	-1.20962
Elovl1	NM_019422	0.000764	-9.23482	8.58929	8.8645	0.826328	-0.27521	-1.21017
Med4	NM_026119	0.002982	-6.44507	7.06483	7.34189	0.825269	-0.27706	-1.21173
Speer4d	NM_025759	0.002226	-6.97114	3.14649	3.42629	0.823707	-0.2798	-1.21402
Mir708	NR_030489	0.000745	-9.29467	3.53017	3.81077	0.823247	-0.2806	-1.2147
Ttc39c	NM_028341	0.00094	-8.7493	6.64333	6.92695	0.821529	-0.28362	-1.21724
Sec61a2	NM_021305	0.004473	-5.77192	8.64427	8.93192	0.819231	-0.28766	-1.22066
	---	0.003775	-6.04594	6.84299	7.13167	0.818651	-0.28868	-1.22152
Vmn2r43	NM_198961	0.001238	-8.14285	4.02455	4.3134	0.81855	-0.28886	-1.22167
Pafah1b2	NM_008775	0.006876	-5.12216	9.28382	9.5754	0.817005	-0.29158	-1.22398
Vps24	NM_025783	0.005978	-5.32692	8.84067	9.13512	0.815379	-0.29446	-1.22642
Prm1	NM_013637	0.001454	-7.80549	4.55637	4.8511	0.81523	-0.29472	-1.22665
Mir376c	NR_030270	0.004274	-5.8443	2.31557	2.61099	0.814836	-0.29542	-1.22724
Galnt11	NM_144908	0.004985	-5.60217	7.53463	7.83219	0.813623	-0.29757	-1.22907
Fbxl17	NM_015794	0.004454	-5.77858	8.46213	8.76061	0.813108	-0.29848	-1.22985
2700062C07Rik	BC084681	0.002281	-6.92652	4.6813	4.98212	0.811793	-0.30082	-1.23184
Lemd2	NM_146075	0.002041	-7.13456	7.55321	7.8546	0.81147	-0.30139	-1.23233
Snap23	NM_001177792	0.00343	-6.20575	8.71625	9.01774	0.811415	-0.30149	-1.23242

Ptges2	NM_133783	0.006328	-5.24279	6.51989	6.82192	0.811109	-0.30203	-1.23288
Dpp8	NM_028906	0.005983	-5.32551	8.55403	8.8565	0.810862	-0.30247	-1.23326
Nek4	NM_011849	0.00681	-5.13595	6.17493	6.47889	0.810023	-0.30397	-1.23453
Morf4l1	NM_001039147	0.00125	-8.12244	7.52746	7.83202	0.809691	-0.30456	-1.23504
Cox7c	NM_007749	0.006759	-5.14684	9.48362	9.79061	0.808327	-0.30699	-1.23712
Med10	NM_138596	0.002234	-6.96477	8.75426	9.06367	0.806974	-0.30941	-1.2392
BC089597	NM_145424	0.0009	-8.84926	4.06328	4.37333	0.806615	-0.31005	-1.23975
Ttc37	NM_001081352	0.004019	-5.94379	8.11823	8.42887	0.806286	-0.31064	-1.24025
	---	0.000478	-10.4263	2.95148	3.26235	0.806156	-0.31087	-1.24045
A230051N06Rik	ENSMUST0000007022 5	0.00308	-6.38937	4.30771	4.6189	0.805978	-0.31119	-1.24073
Speer4e	NM_001122661	0.005525	-5.44496	3.01749	3.32946	0.805544	-0.31197	-1.2414
Bcl6b	NM_007528	0.003044	-6.40974	4.60599	4.9189	0.805019	-0.31291	-1.24221
	---	0.005201	-5.53697	2.33941	2.65338	0.804423	-0.31397	-1.24313
Rgs22	NM_001195748	0.00024	-12.4359	3.61358	3.9281	0.804123	-0.31451	-1.24359
Slc25a29	NM_181328	0.006293	-5.25091	5.88607	6.20114	0.803817	-0.31506	-1.24406
Nap1l4	NM_008672	0.000336	-11.4169	9.26947	9.58699	0.802444	-0.31753	-1.24619
Rab8b	NM_173413	0.001148	-8.30592	8.74791	9.06577	0.802261	-0.31786	-1.24648
Gcg	NM_008100	0.000504	-10.2871	3.41747	3.73618	0.801787	-0.31871	-1.24721
Wwp1	NM_177327	0.002214	-6.98167	7.52401	7.84296	0.801653	-0.31895	-1.24742
Ica1l	NM_027407	0.001157	-8.28728	3.61336	3.93442	0.800483	-0.32106	-1.24925
Smad4	NM_008540	0.000228	-12.6044	9.1324	9.45457	0.799862	-0.32218	-1.25022
	---	0.000494	-10.337	4.89642	5.22008	0.79904	-0.32366	-1.2515
Psmd14	NM_021526	0.003622	-6.11452	8.58837	8.91299	0.798511	-0.32462	-1.25233
Mbtps1	NM_019709	0.003179	-6.33484	9.99212	10.3168	0.798494	-0.32465	-1.25236
Ulk4	NM_177589	0.002329	-6.88761	4.89298	5.21775	0.798424	-0.32477	-1.25247
Agbl2	NM_178755	0.00413	-5.89954	3.41946	3.74506	0.797965	-0.3256	-1.25319
Tmod2	NM_016711	0.003416	-6.21266	4.35183	4.67964	0.796742	-0.32782	-1.25511
Tusc1	NM_026954	0.002955	-6.46101	6.77077	7.09912	0.796447	-0.32835	-1.25558
H3f3a	NM_008210	0.002373	-6.85296	9.87916	10.2089	0.795697	-0.32971	-1.25676
Psmc1	NM_008947	0.005938	-5.33671	8.65771	8.989	0.794827	-0.33129	-1.25814

	---	0.001959	-7.21263	12.2996	12.6335	0.793382	-0.33391	-1.26043
H3f3a	NM_008210	0.005269	-5.51703	9.53024	9.86562	0.792579	-0.33537	-1.2617
Osbpl5	NM_024289	0.005718	-5.39322	7.29916	7.63527	0.792172	-0.33612	-1.26235
Plcb3	NM_008874	0.005709	-5.39564	7.83102	8.16814	0.791624	-0.33711	-1.26323
Foxs1	NM_010226	0.002744	-6.59153	4.99453	5.33209	0.791376	-0.33756	-1.26362
Gm10400	NR_033555	0.001826	-7.34869	3.49524	3.83315	0.791186	-0.33791	-1.26393
Ankrd16	NM_177268	0.001953	-7.21854	6.66461	7.00345	0.79068	-0.33884	-1.26473
Fsd1	NM_183178	0.000768	-9.22246	5.33545	5.67497	0.790308	-0.33951	-1.26533
Fut4	NM_010242	0.005533	-5.44278	4.61458	4.9551	0.789755	-0.34052	-1.26622
Shox2	NM_013665	0.006341	-5.23987	6.3889	6.72982	0.789535	-0.34093	-1.26657
Bend3	NM_199028	0.002524	-6.74117	5.79021	6.13184	0.789147	-0.34164	-1.26719
Setd3	NM_028262	0.000167	-13.6478	7.76485	8.10723	0.78874	-0.34238	-1.26785
	---	0.003967	-5.96468	2.36251	2.70564	0.78833	-0.34313	-1.2685
Srp9	NM_012058	0.002125	-7.05798	8.57643	8.91998	0.788099	-0.34355	-1.26888
	---	0.004388	-5.80225	5.3012	5.64602	0.787409	-0.34481	-1.26999
Gas2	NM_008087	0.002008	-7.16514	5.2795	5.62511	0.786972	-0.34562	-1.27069
	---	0.00456	-5.74157	6.81705	7.16297	0.786809	-0.34591	-1.27096
Vkorc1l1	NM_027121	0.005346	-5.49479	8.20399	8.55239	0.785453	-0.3484	-1.27315
	---	0.006775	-5.14353	10.7051	11.0542	0.785067	-0.34911	-1.27378
Cnpy2	NM_019953	0.004176	-5.8816	8.54424	8.89443	0.784476	-0.3502	-1.27474
Fsip1	NM_027759	0.000187	-13.2654	3.80822	4.15859	0.784386	-0.35037	-1.27488
Gm10012	NR_028042	0.005216	-5.5325	9.43126	9.78743	0.78124	-0.35616	-1.28002
Rft1	NM_177815	0.002744	-6.59133	7.90525	8.26282	0.780482	-0.35756	-1.28126
Tbrg1	NM_025289	0.005935	-5.33748	9.56965	9.92751	0.780319	-0.35786	-1.28153
	---	0.00693	-5.11091	4.20612	4.56469	0.779939	-0.35857	-1.28215
	---	0.003845	-6.0161	11.5256	11.885	0.779479	-0.35942	-1.28291
	---	0.000745	-9.29674	5.51105	5.87057	0.779423	-0.35952	-1.283
Rcor1	NM_198023	0.00034	-11.3787	7.70474	8.0647	0.779185	-0.35996	-1.28339
Ndufs6	NM_010888	0.001943	-7.22826	7.87008	8.23035	0.779021	-0.36027	-1.28366
Ifi27l2b	NM_145449	0.004299	-5.83517	5.36767	5.72846	0.778742	-0.36078	-1.28412
Cdca4	NM_028023	0.00232	-6.89455	8.24278	8.6057	0.777588	-0.36292	-1.28603

	---	0.003836	-6.01996	2.94608	3.30921	0.777473	-0.36314	-1.28622
	---	0.00016	-13.8022	7.04048	7.40389	0.777323	-0.36342	-1.28647
Smok2a	NM_013741	0.003758	-6.05358	3.31797	3.68339	0.776242	-0.36542	-1.28826
Reep3	NM_178606	0.005958	-5.33176	8.15434	8.52051	0.775838	-0.36617	-1.28893
Fam195b	NM_001033231	0.000994	-8.6232	8.71986	9.08833	0.774603	-0.36847	-1.29098
Gm1673	BC147444	0.00393	-5.98	5.84824	6.21727	0.774307	-0.36902	-1.29148
Rplp1	NM_018853	0.001396	-7.88965	12.5204	12.8934	0.77216	-0.37303	-1.29507
Hiatl1	NM_133680	0.001127	-8.3462	8.26657	8.6403	0.771784	-0.37373	-1.2957
Ghrl	NM_021488	0.003119	-6.36786	5.22271	5.59763	0.771146	-0.37493	-1.29677
Pcdhb13	NM_053138	4.32E-05	-19.2165	4.37558	4.75051	0.771143	-0.37493	-1.29678
6430598A04Rik	NM_175521	0.002789	-6.56252	4.62836	5.00645	0.769457	-0.37809	-1.29962
Afg3l2	NM_027130	0.000212	-12.8458	8.1074	8.48593	0.769224	-0.37852	-1.30001
Tmem70	NM_027415	0.001745	-7.43822	7.49337	7.87277	0.768757	-0.3794	-1.3008
Taf9	NM_027592	0.006327	-5.24303	6.89389	7.27617	0.76722	-0.38229	-1.30341
Dpm3	NM_026767	0.00407	-5.92318	6.75686	7.1395	0.767031	-0.38264	-1.30373
Bicc1	NM_031397	0.002007	-7.1667	4.01094	4.39468	0.766446	-0.38374	-1.30472
Olfr1006	NM_146570	0.006555	-5.19126	3.72759	4.11139	0.766416	-0.3838	-1.30477
	---	0.002902	-6.49264	10.4396	10.8236	0.76627	-0.38408	-1.30502
Comtd1	NM_026965	0.006319	-5.24497	6.00089	6.38535	0.766065	-0.38446	-1.30537
	---	0.006337	-5.24067	11.5698	11.955	0.765695	-0.38516	-1.306
Rxra	NM_011305	0.005695	-5.39919	6.66774	7.05377	0.765232	-0.38603	-1.30679
	---	0.002702	-6.61868	3.17149	3.55795	0.765005	-0.38646	-1.30718
Elmo1	NM_080288	0.005229	-5.52867	3.62503	4.01174	0.764871	-0.38671	-1.30741
Ppan	NM_145610	0.002032	-7.14315	7.37055	7.7573	0.764848	-0.38676	-1.30745
Itsn1	NM_010587	0.0011	-8.39756	6.87023	7.2572	0.764731	-0.38698	-1.30765
	---	0.00355	-6.14807	8.79658	9.18474	0.764103	-0.38816	-1.30872
Ppp6c	NM_024209	0.000129	-14.5853	7.95713	8.34537	0.76406	-0.38824	-1.3088
Sec11a	NM_019951	0.000937	-8.75689	10.0053	10.3945	0.763591	-0.38913	-1.3096
	---	0.004919	-5.6229	7.76693	8.15642	0.7634	-0.38949	-1.30993
Manea	NM_172865	0.003884	-5.99925	7.80973	8.19946	0.763274	-0.38973	-1.31015
	---	0.007044	-5.08738	8.3195	8.70953	0.763114	-0.39003	-1.31042

Hgsnat	NM_029884	0.000342	-11.3644	9.39888	9.78892	0.76311	-0.39004	-1.31043
Gca	NM_145523	0.001779	-7.39937	3.1413	3.53149	0.763031	-0.39019	-1.31056
Ahsa1	NM_146036	0.005996	-5.32242	8.80868	9.20087	0.761971	-0.39219	-1.31239
D330045A20Rik	NM_175326	0.003435	-6.2035	3.36026	3.75374	0.761293	-0.39348	-1.31355
Oplah	NM_153122	0.005878	-5.35193	5.7606	6.15433	0.761157	-0.39373	-1.31379
Arhgef6	NM_152801	0.002764	-6.57825	3.31159	3.70567	0.760977	-0.39408	-1.3141
Cmpk2	NM_020557	0.000308	-11.674	3.26698	3.66191	0.760522	-0.39494	-1.31489
	---	0.002543	-6.72722	2.43811	2.8337	0.760181	-0.39559	-1.31548
Pin4	NM_027181	0.005368	-5.48878	7.77095	8.16796	0.759431	-0.39701	-1.31677
	---	0.005113	-5.56311	10.0377	10.4358	0.758869	-0.39808	-1.31775
Dock1	NM_001033420	0.000407	-10.8669	8.49967	8.89822	0.758623	-0.39855	-1.31818
Ss18	NM_009280	0.005151	-5.55183	7.05169	7.45024	0.758622	-0.39855	-1.31818
Ascc3	NM_001146089	0.003383	-6.22928	7.70402	8.10283	0.758481	-0.39882	-1.31842
B230354O11Rik	AK046228	0.003612	-6.11911	4.18625	4.58566	0.758171	-0.3994	-1.31896
Mrrf	NM_026422	0.001699	-7.49093	8.15299	8.5532	0.75775	-0.40021	-1.3197
Scarna13	NR_028576	0.003923	-5.98318	6.05721	6.46038	0.7562	-0.40316	-1.3224
Prss12	NM_008939	0.004872	-5.63772	4.98632	5.39029	0.755777	-0.40397	-1.32314
Hnrnpa0	NM_029872	0.000972	-8.67472	8.06571	8.47002	0.755598	-0.40431	-1.32346
Rbms2	NM_001039080	0.001419	-7.85527	8.96664	9.3714	0.755364	-0.40476	-1.32386
Amy2a5	NM_001042711	0.003512	-6.16626	3.05097	3.45618	0.755126	-0.40521	-1.32428
Amy2a5	NM_001042711	0.003512	-6.16626	3.05097	3.45618	0.755126	-0.40521	-1.32428
Amy2a5	NM_001042711	0.003512	-6.16626	3.05097	3.45618	0.755126	-0.40521	-1.32428
Amy2a5	NM_001042711	0.003512	-6.16626	3.05097	3.45618	0.755126	-0.40521	-1.32428
Hoxb3	NM_001079869	0.003632	-6.10982	5.81212	6.21777	0.754895	-0.40565	-1.32469
Pdcd6ip	NM_001164677	0.004113	-5.90609	8.82604	9.23346	0.75397	-0.40742	-1.32631
Mpzl1	NM_001083897	0.001444	-7.8192	8.1831	8.59151	0.753454	-0.40841	-1.32722
Pin4	NM_027181	0.00574	-5.38738	7.90132	8.31082	0.752883	-0.4095	-1.32823
	---	0.0048	-5.66096	7.79517	8.20995	0.750135	-0.41478	-1.33309
Dync2h1	NM_029851	0.007098	-5.07645	5.45384	5.86959	0.74963	-0.41575	-1.33399
Ccdc114	NM_001033243	0.003364	-6.23841	4.13006	4.54733	0.748842	-0.41727	-1.3354
	---	0.001685	-7.50692	11.0534	11.4722	0.748058	-0.41878	-1.3368

Ttpa	NM_015767	0.003733	-6.06477	3.46446	3.88364	0.747849	-0.41918	-1.33717
Fem1b	NM_010193	0.007021	-5.0921	8.14849	8.56823	0.747555	-0.41975	-1.33769
Glrx2	NM_001038592	0.003786	-6.04132	9.52658	9.94636	0.747539	-0.41978	-1.33772
Prss53	NM_001081268	0.003092	-6.38234	5.27944	5.69957	0.747357	-0.42013	-1.33805
Slc4a7	NM_001033270	0.006879	-5.12142	7.06075	7.48116	0.747213	-0.42041	-1.33831
Abcb10	NM_019552	0.006043	-5.31082	6.21557	6.63653	0.746929	-0.42096	-1.33882
Tep1	NM_009351	0.000804	-9.11379	6.51365	6.93565	0.746389	-0.422	-1.33978
Izumo4	NM_027829	0.001088	-8.42287	5.98913	6.41309	0.745374	-0.42396	-1.34161
E130012A19Rik	BC055770	0.005522	-5.44567	6.01246	6.43729	0.744923	-0.42484	-1.34242
Dym	NM_027727	0.004234	-5.85935	7.54309	7.96804	0.744865	-0.42495	-1.34252
Ttll9	NM_001083618	0.003517	-6.16383	4.47369	4.899	0.744678	-0.42531	-1.34286
Daf2	NM_007827	0.002404	-6.82928	2.80976	3.23558	0.744418	-0.42582	-1.34333
Hdac2	NM_008229	0.005113	-5.56311	9.24268	9.66864	0.744343	-0.42596	-1.34347
Srsy	EU052291	0.000881	-8.89843	2.86459	3.2907	0.744265	-0.42611	-1.34361
Mir434	NR_029953	0.001112	-8.37473	3.09202	3.5182	0.744227	-0.42619	-1.34368
Mfsd7c	NM_145447	0.002045	-7.13103	3.83019	4.25671	0.744056	-0.42652	-1.34398
Bdh1	NM_175177	0.001745	-7.43815	4.52588	4.95446	0.74299	-0.42858	-1.34591
Smad1	NM_008539	0.001076	-8.447	7.08812	7.51739	0.742638	-0.42927	-1.34655
Fbxw2	NM_013890	0.001245	-8.13061	8.01987	8.44925	0.742581	-0.42938	-1.34665
Zfp395	NM_199029	0.005753	-5.38417	6.88019	7.31084	0.741928	-0.43065	-1.34784
Tor1b	NM_133673	0.000647	-9.64353	8.61426	9.048	0.74034	-0.43374	-1.35073
AI317395	NM_144821	0.003405	-6.21792	4.88683	5.32142	0.739907	-0.43459	-1.35152
Npr2	NM_173788	0.005741	-5.3873	7.60461	8.0406	0.739185	-0.43599	-1.35284
Srp54c	NM_001100110	0.006652	-5.16993	9.34431	9.78148	0.73858	-0.43717	-1.35395
5133401N09Rik	NM_198004	0.004186	-5.87794	7.92619	8.36341	0.73856	-0.43721	-1.35399
Rundc2a	NM_001163498	0.002911	-6.48749	5.22264	5.66098	0.737985	-0.43834	-1.35504
Ier5l	NM_030244	0.001508	-7.73067	6.74648	7.18721	0.736758	-0.44074	-1.3573
Daglb	NM_144915	0.000787	-9.16469	6.12621	6.56711	0.736671	-0.44091	-1.35746
Arhgef9	NM_001033329	0.003554	-6.14639	6.51052	6.95222	0.736267	-0.4417	-1.3582
Rfwd2	NM_011931	0.002993	-6.43918	8.06429	8.50671	0.7359	-0.44242	-1.35888
Creb3l1	NM_011957	0.00214	-7.04495	7.2335	7.67636	0.735674	-0.44286	-1.3593

1700036A12Rik	ENSMUST0000006435 1	0.003988	-5.95624	5.09359	5.53697	0.735411	-0.44338	-1.35978
Mtx2	NM_016804	0.001418	-7.85671	7.29841	7.7432	0.734692	-0.44479	-1.36112
Cybasc3	NM_201351	0.005727	-5.39089	8.16768	8.61254	0.734657	-0.44486	-1.36118
Cpsf3	NM_018813	0.001671	-7.52353	8.02719	8.47271	0.734319	-0.44552	-1.36181
Fundc1	NM_028058	0.003172	-6.33864	8.17117	8.61708	0.73412	-0.44591	-1.36218
Pcca	NM_144844	0.00428	-5.84205	7.24952	7.69746	0.733088	-0.44794	-1.36409
Sypl	NM_013635	0.001895	-7.27693	8.41979	8.86871	0.732593	-0.44892	-1.36501
Trpv2	NM_011706	0.005174	-5.54475	4.66612	5.11576	0.732225	-0.44964	-1.3657
Atp1a2	NM_178405	0.004005	-5.94948	5.26525	5.71521	0.73206	-0.44997	-1.36601
Lmbr1	NM_020295	0.005	-5.59744	7.58327	8.03372	0.731815	-0.45045	-1.36647
Speer4d	NM_025759	0.003762	-6.05161	2.9831	3.43363	0.731775	-0.45053	-1.36654
Arcn1	NM_145985	0.00084	-9.00952	8.60077	9.05139	0.731729	-0.45062	-1.36663
Timm10	NM_013899	0.000248	-12.3418	6.45447	6.90564	0.731454	-0.45116	-1.36714
Snora26	NR_031758	0.006523	-5.19837	6.6794	7.13078	0.731339	-0.45139	-1.36736
Ccdc85a	NM_181577	0.006109	-5.29482	4.29901	4.75099	0.731038	-0.45198	-1.36792
	---	0.001425	-7.8466	5.06571	5.51843	0.730666	-0.45272	-1.36861
Aldh5a1	NM_172532	0.005034	-5.58696	4.24695	4.70004	0.730474	-0.4531	-1.36897
Sall2	NM_015772	8.65E-05	-16.124	5.42558	5.87909	0.730262	-0.45351	-1.36937
Gne	NM_015828	0.00064	-9.67082	6.98547	7.44196	0.728756	-0.45649	-1.3722
Pogk	NM_175170	0.006842	-5.12928	5.99919	6.45719	0.727998	-0.45799	-1.37363
Syngr1	NM_207708	0.0026	-6.68758	6.41276	6.87113	0.727805	-0.45838	-1.37399
Zc3h15	NM_026934	0.00198	-7.19192	8.19777	8.65687	0.72744	-0.4591	-1.37468
Ufc1	NM_025388	0.000761	-9.24491	8.08323	8.54234	0.727435	-0.45911	-1.37469
Ei24	NM_007915	0.002608	-6.68201	7.90807	8.36794	0.727054	-0.45987	-1.37541
Cdnf	NM_177647	0.000455	-10.5581	4.50236	4.96291	0.726706	-0.46056	-1.37607
Evc2	NM_145920	0.002542	-6.72786	6.74787	7.20851	0.726664	-0.46064	-1.37615
Lrrc8a	NM_177725	0.002082	-7.09723	7.56252	8.02353	0.726479	-0.46101	-1.3765
Ap4s1	NM_021710	0.001187	-8.2322	5.26063	5.72359	0.725495	-0.46296	-1.37837
Adam5	NM_007401	0.002763	-6.57887	3.08751	3.55114	0.725158	-0.46363	-1.37901
Ssbp2	NM_024272	0.00416	-5.88793	9.73266	10.1969	0.724874	-0.4642	-1.37955

Zfp295	NM_175428	0.005073	-5.57525	6.8419	7.30641	0.724719	-0.46451	-1.37984
Wtip	NM_207212	0.003609	-6.12041	6.91149	7.37607	0.724684	-0.46458	-1.37991
Srp19	NM_025527	0.001783	-7.3949	6.66869	7.13355	0.724539	-0.46486	-1.38019
Nme2	NM_008705	0.005298	-5.50864	9.86994	10.3349	0.724481	-0.46498	-1.3803
Tmed10	NM_026775	0.00298	-6.44645	7.9667	8.43187	0.724386	-0.46517	-1.38048
Rfwd2	NM_011931	0.004099	-5.91176	8.02804	8.49321	0.724386	-0.46517	-1.38048
	---	0.004645	-5.71244	10.3354	10.8017	0.723821	-0.4663	-1.38156
Thbs1	NM_011580	0.001665	-7.53055	11.7496	12.2167	0.723408	-0.46712	-1.38234
Osbpl7	NM_001081434	0.001799	-7.37735	5.59049	6.05833	0.723049	-0.46784	-1.38303
Rnf207	NM_001033489	0.004725	-5.68566	5.40435	5.87243	0.722927	-0.46808	-1.38326
	---	0.003125	-6.36418	5.00906	5.47739	0.722799	-0.46833	-1.38351
Dpagt1	NM_007875	0.002151	-7.03569	7.48266	7.951	0.722793	-0.46835	-1.38352
Impact	NM_008378	0.003707	-6.07601	10.7305	11.1993	0.722553	-0.46883	-1.38398
Srp54b	NM_001100109	0.005621	-5.41895	9.34742	9.81749	0.721931	-0.47007	-1.38517
Tmem14c	NM_025387	0.001513	-7.7234	8.02957	8.49979	0.721854	-0.47022	-1.38532
Col16a1	NM_028266	0.006588	-5.18393	7.38718	7.85911	0.721	-0.47193	-1.38696
Fam165b	NM_138743	0.001274	-8.08052	7.59345	8.06582	0.720775	-0.47238	-1.3874
Zfp217	NM_001033299	0.004749	-5.67774	6.4636	6.93605	0.720739	-0.47245	-1.38746
	---	0.003423	-6.20907	5.53836	6.01137	0.720464	-0.473	-1.38799
Ppp1cc	NM_013636	0.000267	-12.1067	10.4661	10.9414	0.71931	-0.47532	-1.39022
Cep350	NM_001039184	0.003194	-6.32657	6.84349	7.31918	0.719123	-0.47569	-1.39058
Polr3d	NM_025945	0.006185	-5.27655	6.79228	7.26968	0.71827	-0.4774	-1.39223
Wrnip1	NM_030215	0.000347	-11.3175	7.27344	7.75153	0.717928	-0.47809	-1.3929
Psma3	NM_011184	0.002047	-7.12922	8.89357	9.37176	0.717876	-0.47819	-1.393
Cxx1a	NM_024170	0.00097	-8.67792	7.80687	8.28586	0.717478	-0.47899	-1.39377
Srsy	EU052291	0.00045	-10.587	2.63278	3.11183	0.717453	-0.47904	-1.39382
Efhd2	NM_025994	0.002329	-6.88747	7.4681	7.94751	0.717271	-0.47941	-1.39417
Atpif1	NM_007512	0.000135	-14.4059	9.61409	10.0942	0.716905	-0.48015	-1.39488
Slc25a22	NM_026646	0.000161	-13.7796	6.46829	6.94862	0.716817	-0.48032	-1.39506
Herpud2	NM_020586	0.005026	-5.58961	8.77777	9.25856	0.716583	-0.48079	-1.39551
Ndufa11	NM_027244	0.002599	-6.68829	7.88957	8.37044	0.716546	-0.48087	-1.39558

Rps23	NM_024175	0.00347	-6.18609	10.2679	10.7488	0.716518	-0.48093	-1.39564
Cdk7	NM_009874	0.006188	-5.27571	8.05259	8.53537	0.715596	-0.48278	-1.39744
Max	NM_008558	4.40E-05	-19.1263	8.09371	8.57661	0.715534	-0.48291	-1.39756
Ppp1cc	NM_013636	0.001258	-8.10752	10.5081	10.9912	0.715435	-0.48311	-1.39775
	---	0.002538	-6.73062	10.6036	11.0878	0.714904	-0.48418	-1.39879
	---	0.001752	-7.42926	2.08815	2.57504	0.713561	-0.48689	-1.40142
Alg5	NM_025442	0.001856	-7.31744	7.77466	8.26286	0.712916	-0.4882	-1.40269
	---	2.06E-05	-23.1625	8.01568	8.50413	0.712795	-0.48844	-1.40293
Tirap	NM_001177845	0.003865	-6.00757	6.44012	6.92864	0.712757	-0.48852	-1.403
	---	0.001648	-7.55079	10.8376	11.3266	0.712507	-0.48902	-1.40349
Caml	NM_007596	0.001874	-7.29863	6.86452	7.35361	0.712472	-0.4891	-1.40356
Ilvbl	NM_173751	0.000701	-9.44443	7.32897	7.8182	0.712401	-0.48924	-1.4037
	---	0.006949	-5.10688	3.46492	3.95567	0.711656	-0.49075	-1.40517
Paqr8	NM_028829	0.005386	-5.48358	5.21825	5.70906	0.711629	-0.4908	-1.40523
Bend6	NM_177235	0.000799	-9.12915	3.82866	4.31966	0.71153	-0.491	-1.40542
Syap1	NM_025932	0.003644	-6.10475	7.35423	7.8456	0.71135	-0.49137	-1.40578
Col5a2	NM_007737	0.001146	-8.30905	10.3629	10.8571	0.709959	-0.49419	-1.40853
Srsy	EU052291	0.000537	-10.1159	2.8517	3.34654	0.709638	-0.49484	-1.40917
Anapc16	NM_025514	0.000896	-8.86127	8.68579	9.18263	0.708658	-0.49684	-1.41112
Itm2b	NM_008410	0.002226	-6.97138	11.3329	11.8304	0.708321	-0.49752	-1.41179
Mras	NM_008624	0.005109	-5.56423	5.85581	6.35448	0.707758	-0.49867	-1.41291
Srsy	EU052291	0.001584	-7.6309	2.85446	3.35332	0.707668	-0.49886	-1.41309
Bbs7	NM_027810	0.001267	-8.09319	5.86737	6.36762	0.706987	-0.50025	-1.41445
	---	0.000971	-8.67665	3.7782	4.27867	0.706877	-0.50047	-1.41467
Khk	NM_008439	0.005491	-5.45431	6.15442	6.6553	0.706674	-0.50088	-1.41508
Tdrd3	NM_172605	0.00175	-7.43197	7.54051	8.04186	0.706447	-0.50135	-1.41553
Rnase10	NM_029145	0.001775	-7.40451	3.26578	3.76779	0.706121	-0.50201	-1.41619
	---	0.00169	-7.50067	6.68544	7.18775	0.705975	-0.50231	-1.41648
	---	0.005413	-5.47605	3.0822	3.58468	0.705895	-0.50247	-1.41664
Mrps16	NM_025440	0.003858	-6.01021	7.64613	8.14909	0.705657	-0.50296	-1.41712
Leo1	NM_001039522	0.001155	-8.29262	6.2529	6.75651	0.705341	-0.50361	-1.41775

Hprt	NM_013556	0.000844	-8.99816	8.5725	9.07796	0.704434	-0.50547	-1.41958
Dock6	NM_177030	0.0031	-6.37802	6.14354	6.65072	0.703601	-0.50717	-1.42126
Erp44	NM_029572	0.006086	-5.30019	8.61657	9.12379	0.703576	-0.50722	-1.42131
Slc10a3	NM_145406	0.005677	-5.40395	6.80077	7.308	0.703575	-0.50722	-1.42131
Refbp2	NM_019484	0.006898	-5.11757	5.30098	5.80838	0.70349	-0.5074	-1.42148
Psen1	NM_008943	0.002078	-7.10018	8.37204	8.88006	0.703188	-0.50802	-1.4221
Mir411	NR_029916	0.000594	-9.85879	2.61924	3.13062	0.701548	-0.51139	-1.42542
Il17d	NM_145837	0.005752	-5.38441	4.74371	5.25655	0.700841	-0.51284	-1.42686
Anapc13	NM_181394	0.002649	-6.6543	8.43751	8.95272	0.699691	-0.51521	-1.4292
Eno2	NM_013509	0.002438	-6.80369	8.63634	9.15187	0.699536	-0.51553	-1.42952
	---	0.006478	-5.20849	2.80047	3.3164	0.699342	-0.51593	-1.42992
Vps26b	NM_178027	0.002034	-7.14086	8.06835	8.58572	0.698644	-0.51737	-1.43134
Gm505	BC158088	0.001508	-7.72995	6.40974	6.9278	0.698308	-0.51806	-1.43203
Dexi	NM_021428	0.002244	-6.9563	6.97452	7.49307	0.698074	-0.51855	-1.43251
Btg1	NM_007569	0.005069	-5.57654	10.9964	11.5152	0.697975	-0.51875	-1.43272
Pdxdc1	NM_053181	0.001582	-7.63291	8.46884	8.98973	0.696945	-0.52088	-1.43483
Iqsec2	NM_001005475	0.004377	-5.80658	5.44776	5.96878	0.696876	-0.52103	-1.43498
Tmem185b	NM_146103	0.000382	-11.0479	6.38469	6.906	0.696735	-0.52132	-1.43527
	---	0.002299	-6.91143	5.10412	5.62555	0.696683	-0.52143	-1.43537
Gan	NM_001081151	0.000452	-10.5789	6.06346	6.58504	0.696608	-0.52158	-1.43553
Clip3	NM_001081114	0.000719	-9.3815	7.94472	8.46665	0.696443	-0.52192	-1.43587
Pnpla7	NM_146251	0.001801	-7.3753	6.49777	7.02306	0.69482	-0.52529	-1.43922
AI597468	NM_001013028	0.004353	-5.81503	7.70041	8.22601	0.69467	-0.5256	-1.43953
Mtap4	NM_008633	0.001461	-7.79472	7.90794	8.43381	0.694537	-0.52588	-1.43981
	---	0.002233	-6.96538	6.48313	7.00943	0.694334	-0.5263	-1.44023
Cxx1a	NM_024170	0.00107	-8.46016	7.80467	8.33138	0.694134	-0.52671	-1.44064
Fam110b	BC055683	0.002889	-6.50089	5.86093	6.38823	0.693851	-0.5273	-1.44123
	---	0.003137	-6.35758	9.61278	10.1401	0.693839	-0.52733	-1.44126
Spin1	NM_146043	0.000421	-10.776	8.62168	9.14929	0.693704	-0.52761	-1.44154
Kctd5	NM_027008	0.005661	-5.40817	8.31306	8.84285	0.692655	-0.52979	-1.44372
Casd1	NM_145398	0.003362	-6.23935	8.09227	8.62219	0.692593	-0.52992	-1.44385

	---	0.002122	-7.06059	4.39064	4.92069	0.692531	-0.53005	-1.44398
Gm4983	XR_106691	0.001861	-7.31212	5.94668	6.47831	0.691775	-0.53163	-1.44556
Gm4983	XR_106691	0.001861	-7.31212	5.94668	6.47831	0.691775	-0.53163	-1.44556
Gopc	NM_053187	0.000124	-14.7241	7.62722	8.15897	0.691715	-0.53175	-1.44568
	---	0.000107	-15.2699	9.913	10.4466	0.69082	-0.53362	-1.44756
Twist2	NM_007855	0.000684	-9.50601	8.4669	9.00088	0.690648	-0.53398	-1.44792
	---	0.004137	-5.89677	6.80496	7.3393	0.690475	-0.53434	-1.44828
	---	0.001887	-7.28505	8.8533	9.3884	0.690115	-0.53509	-1.44903
Tnnt3	NM_001163664	0.004606	-5.72547	6.05324	6.58922	0.68969	-0.53598	-1.44993
Chst2	NM_018763	0.004367	-5.80996	7.19732	7.73383	0.689437	-0.53651	-1.45046
Adamtsl1	NM_029967	0.003339	-6.25101	4.36894	4.90556	0.689384	-0.53662	-1.45057
Shroom1	NM_027917	0.001373	-7.92409	4.88692	5.42362	0.689344	-0.5367	-1.45065
Zcchc18	NM_001035510	0.000313	-11.6245	4.32982	4.86736	0.688946	-0.53754	-1.45149
Rplp1	NM_018853	0.001407	-7.87217	11.3865	11.9242	0.688912	-0.53761	-1.45156
Jak1	NM_146145	0.001916	-7.25511	9.79044	10.3281	0.688891	-0.53765	-1.45161
Ppif	NM_134084	0.002756	-6.58338	6.19025	6.72853	0.688588	-0.53829	-1.45225
Trmt61b	NR_015549	0.001243	-8.13403	3.81565	4.35557	0.687807	-0.53993	-1.4539
Olfr1314	NM_146450	0.001023	-8.55886	3.00806	3.54889	0.687377	-0.54083	-1.45481
Smyd2	NM_026796	0.005142	-5.55449	7.34974	7.89128	0.687033	-0.54155	-1.45553
Tmem35	NM_026239	0.003255	-6.29429	4.10807	4.65121	0.686272	-0.54315	-1.45715
Gm5533	ENSMUST00000070987	0.003909	-5.98874	3.2208	3.76446	0.686029	-0.54366	-1.45766
Cpped1	NM_146067	0.00069	-9.48224	6.94409	7.48779	0.68601	-0.5437	-1.4577
Dlg2	NM_011807	0.0006	-9.83174	4.16799	4.71364	0.685086	-0.54564	-1.45967
Mettl11a	NM_170592	0.004385	-5.80352	7.97779	8.52424	0.684702	-0.54645	-1.46049
Ipo4	NM_024267	0.002816	-6.54574	8.91738	9.46446	0.684404	-0.54708	-1.46112
	---	0.001302	-8.03446	3.95548	4.50258	0.684394	-0.5471	-1.46115
Lamp2	NM_001017959	0.004844	-5.64684	10.8235	11.3717	0.683858	-0.54823	-1.46229
Chpt1	NM_144807	0.005283	-5.51306	7.71337	8.26218	0.683585	-0.54881	-1.46288
	---	0.006338	-5.24048	3.84653	4.39569	0.683418	-0.54916	-1.46323
Mgat2	NM_146035	0.002773	-6.57271	7.886	8.43518	0.683411	-0.54918	-1.46325

Tusc3	BC069898	0.001943	-7.22898	4.56849	5.1177	0.683392	-0.54921	-1.46329
Selk	NM_019979	0.003599	-6.12542	2.82507	3.37507	0.683021	-0.55	-1.46408
Gm6484	NM_001080940	0.000419	-10.7869	5.44689	5.99777	0.682601	-0.55089	-1.46498
Sytl5	NM_177704	0.004508	-5.75951	3.45212	4.00363	0.682305	-0.55151	-1.46562
Asph	NM_023066	0.004297	-5.83588	7.35489	7.90653	0.682244	-0.55164	-1.46575
Ddi2	NM_001017966	0.001604	-7.60504	7.64092	8.19264	0.682205	-0.55172	-1.46583
Tfdp1	NM_009361	0.004671	-5.7036	8.40291	8.95574	0.681681	-0.55283	-1.46696
	---	0.004025	-5.94117	8.93968	9.4928	0.681542	-0.55313	-1.46726
Ern1	NM_023913	0.006303	-5.24861	6.95886	7.51429	0.680452	-0.55544	-1.46961
Depdc6	NM_001037937	0.000894	-8.86556	7.76474	8.32035	0.68037	-0.55561	-1.46979
	---	0.00389	-5.9968	7.35047	7.90621	0.680307	-0.55574	-1.46993
2010107E04Rik	NM_027360	0.005992	-5.32325	9.47732	10.0331	0.680297	-0.55576	-1.46995
Mapk6	NM_015806	0.001606	-7.60276	8.32019	8.87623	0.680167	-0.55604	-1.47023
Manf	NM_029103	0.004021	-5.94281	8.27863	8.8347	0.680148	-0.55608	-1.47027
Npl	NM_028749	0.001652	-7.54584	3.92794	4.48404	0.68014	-0.5561	-1.47029
Sidt2	NM_172257	0.002069	-7.10895	8.55412	9.11102	0.67976	-0.5569	-1.47111
F830001A07Rik	AK089550	0.00422	-5.86478	4.62193	5.17887	0.67974	-0.55695	-1.47115
Apol6	NM_028010	0.004312	-5.83044	4.54911	5.10779	0.678921	-0.55869	-1.47293
Pigh	NM_029988	0.000255	-12.251	5.33764	5.89633	0.678916	-0.5587	-1.47294
Morf4l2	NM_019768	0.006256	-5.25976	9.80676	10.3667	0.678322	-0.55996	-1.47423
H2-K1	NM_001001892	0.006439	-5.21739	8.32977	8.89089	0.677775	-0.56112	-1.47542
Cebpb	NM_009883	0.001976	-7.1963	8.19123	8.75287	0.677531	-0.56164	-1.47595
Cep120	NM_178686	0.000888	-8.88215	7.15015	7.71204	0.677415	-0.56189	-1.4762
	---	0.000344	-11.3444	3.18156	3.74408	0.677121	-0.56252	-1.47684
Atp5sl	NM_025504	0.000961	-8.69929	6.15496	6.71796	0.676892	-0.563	-1.47734
Speer8-ps1	NR_001584	0.006081	-5.30146	4.623	5.18788	0.676009	-0.56489	-1.47927
Srsy	EU052291	0.003504	-6.17008	2.81014	3.37504	0.676002	-0.5649	-1.47929
Uimc1	NM_011307	8.82E-05	-16.0463	6.7131	7.27854	0.675751	-0.56544	-1.47984
Cltb	NM_028870	0.000434	-10.6921	7.74067	8.30622	0.6757	-0.56555	-1.47995
Akap13	NM_029332	0.002145	-7.04037	7.93311	8.49943	0.675337	-0.56632	-1.48074
Sh3pxd2b	NM_177364	0.001397	-7.88841	8.36282	8.92958	0.675131	-0.56676	-1.4812

Slc9a6	NM_172780	0.000203	-12.9788	8.06721	8.63404	0.675097	-0.56683	-1.48127
Hoxb5	NM_008268	0.001762	-7.41861	5.21261	5.77986	0.674901	-0.56725	-1.4817
Arhgap1	NR_027373	0.001619	-7.58647	7.65768	8.22566	0.674558	-0.56799	-1.48245
Clptm1l	NM_146047	0.000922	-8.79417	9.4355	10.0035	0.674536	-0.56803	-1.4825
Appl1	NM_145221	0.002805	-6.55269	7.652	8.22004	0.674534	-0.56804	-1.48251
Sh3bgrl	NM_019989	0.001245	-8.12965	10.1464	10.7147	0.674426	-0.56827	-1.48274
Vps8	NM_001081366	0.000611	-9.78471	7.40116	7.97143	0.673493	-0.57027	-1.4848
Ier3ip1	NM_025409	1.49E-05	-25.1158	7.82647	8.39733	0.673213	-0.57087	-1.48541
	---	0.001405	-7.87581	4.55185	5.12422	0.672513	-0.57237	-1.48696
Zdhhc1	NM_175160	0.002172	-7.01702	6.96562	7.53815	0.67244	-0.57252	-1.48712
Bach1	NM_007520	0.002956	-6.46041	7.96825	8.54233	0.671713	-0.57408	-1.48873
Fermt2	NM_146054	0.006463	-5.212	8.76951	9.34571	0.67073	-0.5762	-1.49091
Trappc4	NM_021789	0.001749	-7.43292	7.8364	8.41275	0.670661	-0.57634	-1.49107
Fam115a	BC031718	0.004228	-5.86177	8.51282	9.09206	0.669315	-0.57924	-1.49406
Laptm4a	NM_008640	0.001227	-8.16192	11.3445	11.924	0.669175	-0.57955	-1.49438
Cul7	NM_025611	0.000172	-13.5366	7.3356	7.91515	0.669173	-0.57955	-1.49438
Hlf	NM_172563	0.003572	-6.13787	4.64076	5.22191	0.668435	-0.58114	-1.49603
Ninl	NM_207204	0.002896	-6.49662	6.11325	6.69488	0.668208	-0.58163	-1.49654
Il1r2	NM_010555	0.005234	-5.52726	3.88938	4.47212	0.667692	-0.58274	-1.4977
Mmgt1	NM_146234	0.002864	-6.51614	7.47043	8.05479	0.666945	-0.58436	-1.49937
Cnih	NM_009919	0.003276	-6.28375	9.102	9.68717	0.666572	-0.58517	-1.50021
LOC100041704	ENSMUST0000010601<br>0	0.003028	-6.41894	2.81812	3.40338	0.666529	-0.58526	-1.50031
5430411K18Rik	NM_001195633	0.00181	-7.36571	6.60504	7.1909	0.666252	-0.58586	-1.50093
St8sia2	NM_009181	0.001381	-7.9123	4.46971	5.05592	0.666091	-0.58621	-1.5013
Mical2	NM_001193305	0.001014	-8.57861	7.6569	8.24406	0.665649	-0.58717	-1.50229
	---	0.004552	-5.74441	3.99989	4.58766	0.665372	-0.58777	-1.50292
Slc31a2	NM_025286	0.004768	-5.67132	7.51704	8.1071	0.664319	-0.59005	-1.5053
	---	0.000546	-10.0737	9.00953	9.60043	0.66393	-0.5909	-1.50618
Rnd3	NM_028810	0.000178	-13.4264	9.36758	9.95857	0.663888	-0.59099	-1.50628
Txndc11	NM_029582	0.001557	-7.66584	7.26833	7.86023	0.663467	-0.5919	-1.50723

Gm98	NM_001033481	0.000516	-10.2224	4.9982	5.59111	0.663003	-0.59291	-1.50829
Sgce	NM_001130190	0.000813	-9.08809	8.79221	9.38556	0.662804	-0.59335	-1.50874
Wdr82	NM_029896	0.000208	-12.8987	9.19992	9.79496	0.662028	-0.59504	-1.51051
Grin2d	NM_008172	0.000529	-10.1598	4.97279	5.56932	0.661345	-0.59653	-1.51207
Atad2b	NM_001099628	4.77E-05	-18.7487	6.41481	7.01255	0.660789	-0.59774	-1.51334
Znf512b	NM_001164597	0.002438	-6.80376	5.88423	6.48502	0.659396	-0.60078	-1.51654
Pdzrn3	NM_018884	0.00389	-5.99701	8.80398	9.40492	0.659324	-0.60094	-1.5167
	---	5.92E-05	-17.7522	9.0743	9.67528	0.65931	-0.60097	-1.51674
Xpr1	NM_011273	0.001962	-7.21001	7.50541	8.10676	0.659134	-0.60136	-1.51714
Mcart6	ENSMUST00000113070	0.001217	-8.17932	5.40677	6.00844	0.658994	-0.60166	-1.51746
Phf2	NM_011078	1.08E-05	-27.2624	6.56646	7.16814	0.658983	-0.60169	-1.51749
Zfp820	NM_029281	0.00681	-5.13612	4.14134	4.74313	0.658935	-0.60179	-1.5176
Nrbf2	NM_001036293	0.005476	-5.45851	6.90123	7.50303	0.658931	-0.6018	-1.51761
	---	0.001733	-7.45095	9.4631	10.0652	0.658786	-0.60212	-1.51794
Tm2d1	NM_053157	0.003287	-6.27795	7.84262	8.44563	0.658382	-0.603	-1.51888
Psmg1	NM_019537	0.001828	-7.34655	7.74247	8.34761	0.657407	-0.60514	-1.52113
A030009H04Rik	NR_027827	0.000163	-13.7256	4.91892	5.52496	0.656999	-0.60604	-1.52207
Mobkl2a	NM_172457	0.001085	-8.42961	7.45432	8.06137	0.656538	-0.60705	-1.52314
0610007P08Rik	NM_001013608	0.005369	-5.48827	6.363	6.97124	0.655995	-0.60824	-1.5244
LOC100039552	ENSMUST00000111407	0.004918	-5.62315	2.94709	3.55656	0.655437	-0.60947	-1.5257
Atf1	NM_007497	0.003528	-6.15835	7.54292	8.1524	0.655432	-0.60948	-1.52571
Srsy	EU052291	0.001017	-8.57246	2.84539	3.45497	0.655388	-0.60958	-1.52581
Armcx4	ENSMUST00000055842	0.001768	-7.41225	5.23926	5.84934	0.655157	-0.61009	-1.52635
Eif4ebp2	NM_010124	0.001819	-7.3567	6.47403	7.08446	0.655	-0.61043	-1.52672
Mir412	NR_029917	0.005149	-5.55242	4.0293	4.6404	0.654697	-0.6111	-1.52742
Pex11b	NM_011069	0.004214	-5.86726	6.8445	7.45611	0.654463	-0.61162	-1.52797
Srsy	XR_106493	0.000197	-13.0909	2.82929	3.44265	0.653673	-0.61336	-1.52982
Col11a1	NM_007729	0.007088	-5.07849	5.49492	6.10896	0.653364	-0.61404	-1.53054
Sh3bp5	NM_011894	0.003644	-6.10449	6.99156	7.60582	0.653267	-0.61426	-1.53077
LOC100043371	ENSMUST0000010156	0.000187	-13.2588	6.03669	6.65159	0.652973	-0.61491	-1.53146

	8							
E2f6	NM_033270	0.001543	-7.68394	6.49147	7.10646	0.652935	-0.61499	-1.53155
Sytl5	NM_177704	0.004568	-5.73878	3.95745	4.57293	0.652711	-0.61548	-1.53207
Tbce	NM_178337	0.000655	-9.61327	7.28921	7.90531	0.652432	-0.6161	-1.53273
	---	0.001313	-8.01817	3.76833	4.38695	0.651296	-0.61862	-1.5354
LOC100041704	ENSMUST00000106010	0.006377	-5.23159	3.228	3.84663	0.65129	-0.61863	-1.53541
Zfp14	NM_011748	0.006805	-5.13718	5.91023	6.53067	0.650472	-0.62044	-1.53735
Cmtm3	NM_024217	0.000321	-11.5506	9.71378	10.3355	0.64989	-0.62173	-1.53872
G6pc3	NM_175935	0.002045	-7.1309	7.92672	8.54866	0.649796	-0.62194	-1.53894
Tead1	NM_001166584	0.003941	-5.97577	8.83709	9.45988	0.649411	-0.6228	-1.53986
Mir668	NR_030424	0.00137	-7.92877	5.18099	5.80383	0.649394	-0.62283	-1.5399
Mmadhc	NM_133839	0.000918	-8.80434	7.90713	8.53064	0.649091	-0.62351	-1.54062
Slc17a5	NM_172773	0.000627	-9.71972	8.16638	8.78996	0.649057	-0.62358	-1.5407
Tmed10	NM_026775	1.67E-05	-24.4282	9.5449	10.1702	0.648283	-0.6253	-1.54254
Gmds	NM_146041	0.003828	-6.02306	7.08499	7.71101	0.647958	-0.62603	-1.54331
	---	0.003928	-5.98089	10.2234	10.8494	0.647955	-0.62603	-1.54332
Mir494	NR_030269	0.003529	-6.15815	4.21812	4.84421	0.647928	-0.6261	-1.54338
Agtpbp1	NM_023328	0.001047	-8.50737	6.66075	7.28717	0.647783	-0.62642	-1.54373
Mir99a	NR_029535	0.005466	-5.46109	4.24424	4.87094	0.647659	-0.62669	-1.54402
Ddit4	NM_029083	0.00078	-9.1845	8.18266	8.8096	0.647547	-0.62694	-1.54429
Acat1	NM_144784	0.000412	-10.8314	8.35623	8.98355	0.647376	-0.62733	-1.5447
Irak1	NM_001177973	0.000142	-14.2233	7.09427	7.72248	0.646977	-0.62821	-1.54565
Rassf1	NM_019713	0.002407	-6.82687	7.06489	7.69391	0.646617	-0.62902	-1.54651
Evi2a	NM_001033711	0.005103	-5.56618	4.46122	5.09109	0.646232	-0.62988	-1.54743
Gm13777	XM_915186	0.000152	-13.9686	6.19705	6.82975	0.64497	-0.6327	-1.55046
Mir539	NR_030262	0.001457	-7.80062	2.62922	3.26237	0.644766	-0.63315	-1.55095
	---	0.003848	-6.01467	5.65728	6.29118	0.644435	-0.63389	-1.55175
Aldh3a2	NM_007437	0.004997	-5.59847	9.0916	9.72716	0.643688	-0.63557	-1.55355
Gm1973	NM_029288	0.002189	-7.00287	2.68356	3.31947	0.643534	-0.63591	-1.55392
Eif1a	NM_010120	0.001022	-8.56122	7.87946	8.51645	0.643054	-0.63699	-1.55508

Trp53bp2	NM_173378	0.00018	-13.396	7.11091	7.7496	0.642298	-0.63869	-1.55691
	---	0.001221	-8.171	6.60564	7.24634	0.641403	-0.6407	-1.55908
Cr1l	NM_013499	0.002322	-6.89306	6.75915	7.40031	0.641195	-0.64117	-1.55959
Arf4	NM_007479	0.003108	-6.37372	10.6059	11.2472	0.641134	-0.6413	-1.55974
Tmed9	NM_026211	0.006454	-5.21396	7.83156	8.47577	0.63984	-0.64422	-1.56289
Cpxm2	NM_018867	0.0067	-5.15959	4.3157	4.96043	0.639608	-0.64474	-1.56346
Atg2b	NM_029654	0.004243	-5.85619	6.9343	7.57935	0.639475	-0.64504	-1.56378
Twf2	NM_011876	0.006494	-5.20499	6.31385	6.95962	0.639152	-0.64577	-1.56457
9430020K01Rik	NM_001081963	0.002387	-6.84226	7.43188	8.07879	0.638647	-0.64691	-1.56581
Hivep1	NM_007772	0.001543	-7.68367	7.13452	7.783	0.637954	-0.64848	-1.56751
Siva1	NM_013929	0.006963	-5.10398	6.77895	7.42889	0.637309	-0.64994	-1.5691
Zbtb34	NM_001085507	0.005878	-5.35206	5.85951	6.50985	0.63713	-0.65034	-1.56954
	---	0.002451	-6.7944	7.38792	8.03904	0.636782	-0.65113	-1.5704
Sec61b	NM_024171	0.001151	-8.30023	7.38481	8.03613	0.636695	-0.65133	-1.57061
Zc3h13	NM_026083	0.000174	-13.5031	7.34632	7.99795	0.636558	-0.65164	-1.57095
	---	0.001833	-7.34106	3.77751	4.42957	0.636371	-0.65206	-1.57141
Ust	BC138155	0.000784	-9.175	3.36498	4.01862	0.635675	-0.65364	-1.57313
LOC665406	ENSMUST00000115943	0.001546	-7.68023	2.84028	3.49405	0.635617	-0.65377	-1.57327
H13	NM_001159551	0.003042	-6.41084	8.86476	9.52004	0.634954	-0.65528	-1.57492
Nrbf2	NM_001036293	0.004578	-5.73534	6.84759	7.50353	0.63466	-0.65594	-1.57565
Nrbf2	NM_001036293	0.004578	-5.73534	6.84759	7.50353	0.63466	-0.65594	-1.57565
Nvl	NM_026171	0.000522	-10.1915	7.64353	8.30032	0.634285	-0.6568	-1.57658
Brp44	NM_027430	2.10E-05	-23.0346	6.88706	7.54392	0.634259	-0.65686	-1.57664
Abl1	NM_001112703	0.001099	-8.40038	7.72107	8.37907	0.633756	-0.658	-1.57789
Thnsl2	NM_178413	0.002107	-7.07415	4.67638	5.33455	0.633683	-0.65817	-1.57808
Elac1	NM_053255	0.005356	-5.49212	6.28242	6.9412	0.633411	-0.65879	-1.57875
Kiss1r	NM_053244	0.00419	-5.87633	5.17558	5.83682	0.632333	-0.66125	-1.58145
Epm2a	NM_010146	0.002553	-6.72013	4.34451	5.0067	0.631921	-0.66219	-1.58248
Mia3	NM_177389	0.003387	-6.22691	7.50773	8.17008	0.631847	-0.66235	-1.58266
Fastkd2	NM_172422	0.005852	-5.35866	6.55429	7.21685	0.631756	-0.66256	-1.58289

Sdcbp	NM_001098227	0.002557	-6.71731	9.94604	10.6112	0.630634	-0.66513	-1.58571
Itpkb	NM_001081175	0.001108	-8.38247	6.65194	7.31753	0.630433	-0.66558	-1.58621
Tsr2	NM_001164578	0.001036	-8.53039	7.30866	7.97446	0.630339	-0.6658	-1.58645
Cep350	NM_001039184	0.003117	-6.36868	6.27108	6.93708	0.630253	-0.666	-1.58666
Ap3b1	NM_009680	0.000257	-12.224	8.48893	9.15496	0.630238	-0.66603	-1.5867
Auh	NM_016709	0.003286	-6.27837	7.75915	8.42589	0.62993	-0.66674	-1.58748
Gng12	NM_001177560	0.005272	-5.51608	9.91958	10.5872	0.62955	-0.66761	-1.58843
Tprkb	NM_001170488	0.006946	-5.10751	7.72867	8.39656	0.629426	-0.66789	-1.58875
2200002K05Rik	BC055786	0.002467	-6.7827	4.02873	4.6972	0.629174	-0.66847	-1.58938
Abhd4	NM_134076	0.006719	-5.15551	8.51631	9.18532	0.628939	-0.66901	-1.58998
	---	0.005078	-5.57358	2.80861	3.47852	0.628547	-0.66991	-1.59097
Mrpl52	NM_026851	9.71E-05	-15.6604	7.67031	8.3409	0.628247	-0.6706	-1.59173
Syngap1	ENSMUST00000081285	0.003332	-6.25479	6.7611	7.43183	0.62819	-0.67073	-1.59188
	---	0.002531	-6.73631	9.16808	9.83909	0.628067	-0.67101	-1.59219
Sox12	NM_011438	0.000565	-9.98548	4.77604	5.44751	0.627868	-0.67147	-1.59269
Aldh2	NM_009656	0.002466	-6.78315	9.19493	9.86693	0.627637	-0.672	-1.59328
Mblac1	NM_177878	0.004245	-5.85529	5.22783	5.90194	0.62672	-0.67411	-1.59561
Dusp12	NM_023173	0.000571	-9.95925	5.86957	6.54602	0.625707	-0.67644	-1.59819
Tsnax	NM_016909	0.004768	-5.67139	7.48039	8.15716	0.625564	-0.67677	-1.59856
C230055K05Rik	NM_001039231	0.00699	-5.0985	4.47327	5.15059	0.625327	-0.67732	-1.59916
Ptp4a1	NM_011200	0.000505	-10.2784	9.47843	10.1565	0.624985	-0.67811	-1.60004
Mfap5	NM_015776	0.005757	-5.38301	8.27839	8.95691	0.624806	-0.67852	-1.6005
B930041F14Rik	NM_178699	0.003524	-6.16026	5.31736	5.99665	0.624471	-0.67929	-1.60136
Elovl5	NM_134255	1.53E-05	-24.9581	9.31685	9.99653	0.624301	-0.67969	-1.60179
Srsy	XR_106493	0.0027	-6.61991	3.1583	3.8397	0.623563	-0.68139	-1.60369
Stt3a	NM_008408	0.001529	-7.70278	10.8212	11.5051	0.622442	-0.68399	-1.60658
9030624G23Rik	ENSMUST00000101538	4.87E-05	-18.6455	5.97019	6.65467	0.622231	-0.68448	-1.60712
Maged1	NM_019791	0.003775	-6.04616	9.34024	10.0254	0.621951	-0.68513	-1.60784
Gm5458	NM_001024706	0.000136	-14.3897	4.11824	4.8038	0.621768	-0.68555	-1.60832
Epdr1	NM_134065	0.001172	-8.26056	8.90172	9.58731	0.62175	-0.68559	-1.60836

Adamts2	NM_175643	0.003875	-6.003	8.22391	8.90981	0.621617	-0.6859	-1.60871
6330403K07Rik	NM_134022	0.004257	-5.85075	4.64173	5.32767	0.621599	-0.68595	-1.60876
Ndufa1	NM_019443	0.003149	-6.35109	7.53883	8.22602	0.621062	-0.68719	-1.61014
Creb3l2	NM_178661	0.001844	-7.32926	7.06669	7.75401	0.621004	-0.68733	-1.61029
Gtf3c6	NM_026113	0.000535	-10.1301	6.32819	7.01697	0.620377	-0.68878	-1.61192
B4galt6	NM_019737	0.001095	-8.40869	6.03802	6.72735	0.620141	-0.68933	-1.61254
Rab3gap2	NM_001163754	0.001223	-8.16817	7.33966	8.02989	0.619753	-0.69024	-1.61355
Mir218-2	NR_029799	0.002954	-6.46148	3.94684	4.6371	0.619739	-0.69027	-1.61358
Ypel4	NM_001005342	0.0032	-6.32339	6.47335	7.16367	0.619716	-0.69032	-1.61364
Wnk3-ps	BC043119	0.00446	-5.77646	3.07202	3.76359	0.619179	-0.69157	-1.61504
Uchl1	NM_011670	0.005128	-5.55869	4.87519	5.56824	0.618545	-0.69305	-1.6167
	---	0.001619	-7.58711	2.92089	3.61542	0.617912	-0.69453	-1.61835
Parp6	NM_029922	0.002916	-6.48404	6.96376	7.65858	0.617785	-0.69482	-1.61869
Ppp4r4	NM_028980	0.001233	-8.15135	2.98264	3.67814	0.617494	-0.6955	-1.61945
Ap3s1	NM_009681	0.000397	-10.9342	7.87124	8.56734	0.617237	-0.6961	-1.62012
Def6	NM_027185	0.005495	-5.45323	5.14385	5.84201	0.616355	-0.69817	-1.62244
Adcy4	NM_080435	0.005856	-5.35749	5.11533	5.81383	0.616212	-0.6985	-1.62282
Ptp4a1	NM_011200	0.000448	-10.6021	9.48196	10.1809	0.61603	-0.69893	-1.6233
	---	0.006988	-5.09899	8.49199	9.19103	0.615982	-0.69904	-1.62342
	---	0.00021	-12.8654	8.8195	9.51984	0.61543	-0.70033	-1.62488
Stag3	NM_016964	0.004895	-5.63052	3.96093	4.6627	0.614819	-0.70177	-1.6265
Nsmaf	NM_010945	0.00552	-5.44621	7.39571	8.09823	0.614499	-0.70252	-1.62734
Ppib	NM_011149	0.000783	-9.17596	10.4618	11.1657	0.613893	-0.70394	-1.62895
Mir143	NR_029601	0.000828	-9.04559	5.2099	5.91497	0.613409	-0.70508	-1.63023
Srm	NM_009272	0.001257	-8.10967	7.82869	8.53432	0.613175	-0.70563	-1.63086
Pou6f1	NM_010127	0.003622	-6.1146	5.57795	6.28439	0.612831	-0.70644	-1.63177
Cln5	NM_001033242	0.002341	-6.878	6.90711	7.61385	0.612702	-0.70674	-1.63212
Pgrmc1	NM_016783	0.005597	-5.42526	8.32517	9.03254	0.612437	-0.70737	-1.63282
LOC100504530	XM_003085484	0.005554	-5.43707	3.07499	3.78244	0.612403	-0.70745	-1.63291
LOC100504530	XM_003085484	0.005554	-5.43707	3.07499	3.78244	0.612403	-0.70745	-1.63291
LOC100504530	XM_003085484	0.005554	-5.43707	3.07499	3.78244	0.612403	-0.70745	-1.63291

Spire1	NM_194355	0.001826	-7.34901	5.8615	6.56904	0.612362	-0.70754	-1.63302
Hccs	NM_008222	0.002913	-6.4864	7.91245	8.62106	0.611909	-0.70861	-1.63423
	---	0.001309	-8.02343	8.3477	9.05719	0.611535	-0.70949	-1.63523
4930583H14Rik	NR_028121	0.004923	-5.62169	3.37635	4.08594	0.611493	-0.70959	-1.63534
Arhgap20	NM_175535	0.000443	-10.6307	3.98062	4.69034	0.61144	-0.70972	-1.63548
	---	0.001404	-7.87675	3.03022	3.73998	0.611425	-0.70975	-1.63552
Zfp286	NM_138949	0.004098	-5.91194	3.86435	4.57532	0.610909	-0.71097	-1.63691
Gm1973	NM_029288	0.002836	-6.53319	3.39965	4.11126	0.610641	-0.7116	-1.63762
Slc35f5	NM_028787	3.93E-05	-19.6881	9.45344	10.1652	0.610579	-0.71175	-1.63779
Evc	NM_021292	8.52E-05	-16.1871	7.02147	7.73444	0.610065	-0.71297	-1.63917
2310079N02Rik	NM_025636	0.000552	-10.0443	5.93071	6.64467	0.609641	-0.71397	-1.64031
Fam84b	NM_001162926	0.001655	-7.54317	4.75539	5.46973	0.609485	-0.71434	-1.64073
Chsy1	NM_001081163	0.005896	-5.34745	8.94482	9.65987	0.609187	-0.71504	-1.64153
Aebp1	NM_009636	0.00104	-8.52281	10.8639	11.5805	0.60851	-0.71665	-1.64336
St5	NM_001001326	0.006597	-5.18215	7.09195	7.80972	0.608039	-0.71777	-1.64463
Pdap1	NM_001033313	0.00075	-9.28119	6.91013	7.62976	0.607253	-0.71963	-1.64676
Rsl1	NM_001013769	0.004866	-5.63961	5.11903	5.83963	0.606843	-0.7206	-1.64787
Siva1	NM_013929	0.000122	-14.7829	6.00874	6.72986	0.606623	-0.72113	-1.64847
Rnaseh1	NM_011275	0.005142	-5.55427	5.95366	6.6751	0.606491	-0.72144	-1.64883
Lima1	NM_001113545	0.00569	-5.4005	9.02768	9.74947	0.606343	-0.72179	-1.64923
Zfyve16	NM_173392	0.005368	-5.48854	6.38003	7.10448	0.605228	-0.72445	-1.65227
Cox5a	NM_007747	0.003208	-6.31925	8.33331	9.05785	0.605191	-0.72454	-1.65237
Serf1	NM_011353	0.00311	-6.37233	5.84675	6.57145	0.605123	-0.7247	-1.65256
Fam151b	NM_001163627	0.000688	-9.49088	4.82106	5.54631	0.604891	-0.72525	-1.65319
Ap3s1	NM_009681	0.001003	-8.60347	8.76141	9.48698	0.604759	-0.72557	-1.65355
Pgf	NM_008827	0.005181	-5.54286	4.35098	5.07692	0.604599	-0.72595	-1.65399
Hspg2	NM_008305	0.000361	-11.2031	8.07062	8.79684	0.604486	-0.72622	-1.6543
Sucla2	NM_011506	0.001938	-7.23348	8.29478	9.02116	0.604417	-0.72638	-1.65449
1810063B07Rik	NM_026209	0.000173	-13.5218	4.44148	5.16815	0.604298	-0.72667	-1.65481
Nkd1	NM_027280	0.000255	-12.2507	5.22588	5.95308	0.604072	-0.72721	-1.65543
Ssty2	NM_023546	0.004002	-5.95068	3.29194	4.02185	0.60294	-0.72991	-1.65854

	---	0.002455	-6.7909	10.1283	10.8585	0.602817	-0.73021	-1.65888
Clasp2	NM_001114347	0.00174	-7.44382	7.00438	7.73523	0.602548	-0.73085	-1.65962
	---	0.003177	-6.33594	3.99407	4.72502	0.602508	-0.73095	-1.65973
C530028O21Rik	NM_175696	0.004903	-5.62784	5.34596	6.07692	0.602506	-0.73095	-1.65973
Rps4y2	NR_003634	8.43E-06	-28.9875	5.14329	5.87441	0.602438	-0.73112	-1.65992
Ap3s1	NM_009681	0.001446	-7.81606	7.7998	8.53154	0.602178	-0.73174	-1.66064
Foxo3	ENSMUST00000105502	0.002601	-6.68655	9.4875	10.2211	0.601416	-0.73357	-1.66274
Capn7	NM_009796	0.002889	-6.50052	7.6681	8.40222	0.601184	-0.73412	-1.66338
	---	0.00075	-9.27933	4.53963	5.27447	0.600884	-0.73484	-1.66421
Lgmn	NM_011175	0.001019	-8.56743	9.97883	10.7138	0.600836	-0.73496	-1.66435
Zmiz1	ENSMUST00000162645	0.001439	-7.82647	9.35754	10.0927	0.600746	-0.73517	-1.6646
Gga2	NM_028758	0.000444	-10.6236	7.07164	7.80761	0.600412	-0.73598	-1.66552
	---	0.003101	-6.37768	8.34387	9.08006	0.600322	-0.73619	-1.66577
Glul	NM_008131	0.005351	-5.49345	8.334	9.07068	0.600119	-0.73668	-1.66633
Prdx1	NM_011034	0.006417	-5.22231	9.42972	10.1669	0.599931	-0.73713	-1.66686
Slc29a3	NM_023596	0.000289	-11.8629	6.79763	7.53497	0.599847	-0.73733	-1.66709
Adk	NM_134079	0.00032	-11.558	8.43894	9.17629	0.599839	-0.73735	-1.66711
Ptp4a1	NM_011200	0.000258	-12.2175	9.29059	10.0293	0.599283	-0.73869	-1.66866
Traf3ip2	NM_134000	0.003882	-6.00022	7.0453	7.78418	0.599203	-0.73888	-1.66888
Sirt1	NM_019812	0.006237	-5.26422	6.30955	7.04943	0.59879	-0.73988	-1.67004
Copz2	NM_019877	0.000288	-11.8769	9.34781	10.0878	0.598746	-0.73999	-1.67016
Rfpl4b	NM_001177783	0.00235	-6.87141	3.10628	3.8471	0.598396	-0.74083	-1.67113
Lix1l	NM_001163170	0.000834	-9.0279	9.10378	9.84576	0.597921	-0.74197	-1.67246
Mpp1	NM_008621	0.000189	-13.2282	8.11099	8.85452	0.597276	-0.74353	-1.67427
Atp6v1h	NM_133826	0.001884	-7.28765	8.22453	8.9689	0.596929	-0.74437	-1.67524
Itga4	NM_010576	0.003228	-6.30871	4.2314	4.97584	0.596899	-0.74444	-1.67532
Ntan1	NM_010946	0.000442	-10.6385	7.01446	7.76095	0.596051	-0.74649	-1.67771
Copb2	NM_015827	0.006818	-5.13427	9.94586	10.6924	0.596045	-0.74651	-1.67772
LOC100041256	BC089466	0.001327	-7.99453	3.42249	4.16946	0.595856	-0.74697	-1.67826
Stc2	NM_011491	0.005169	-5.54626	4.74885	5.50045	0.593947	-0.75159	-1.68365

Nptx1	NM_008730	0.000924	-8.78912	4.90077	5.65254	0.593875	-0.75177	-1.68386
	---	0.003284	-6.27919	4.38861	5.14082	0.593692	-0.75221	-1.68437
Hhat	NM_144881	0.000328	-11.4901	4.92736	5.68094	0.593132	-0.75357	-1.68596
Ptp4a1	NM_011200	0.000214	-12.8037	9.19081	9.94512	0.59283	-0.75431	-1.68682
Lgals8	NM_018886	0.000844	-9.00013	8.80731	9.56232	0.592542	-0.75501	-1.68765
Dusp19	NM_024438	0.001735	-7.44873	6.65051	7.40631	0.592218	-0.7558	-1.68857
	---	0.004567	-5.73894	8.68309	9.43957	0.591942	-0.75647	-1.68936
Gnl3	NM_153547	0.000506	-10.2723	8.41154	9.16868	0.591664	-0.75715	-1.69015
Cacnb2	NM_023116	0.003665	-6.09504	6.33926	7.09779	0.591096	-0.75854	-1.69177
Zfp810	NM_145612	0.002217	-6.97889	4.06153	4.82026	0.591015	-0.75873	-1.692
Zfp618	NM_028326	0.005768	-5.38007	5.01779	5.77662	0.590977	-0.75883	-1.69211
Sec61b	NM_024171	0.002381	-6.84689	5.93515	6.69776	0.58943	-0.76261	-1.69655
Ngly1	NM_021504	0.001532	-7.69762	7.09319	7.85631	0.58922	-0.76312	-1.69716
Ear1	NM_007894	0.000404	-10.8849	3.57925	4.34381	0.588636	-0.76455	-1.69884
	---	0.002604	-6.68491	3.23616	4.00079	0.588601	-0.76464	-1.69894
Bdh2	NM_001172055	0.006095	-5.29812	8.37727	9.14458	0.587509	-0.76732	-1.7021
Ifi27l1	NM_026790	0.002623	-6.67201	8.744	9.51195	0.587251	-0.76795	-1.70285
Hoxd9	NM_013555	0.000284	-11.9182	4.99532	5.7636	0.58712	-0.76827	-1.70323
Gk5	NM_177352	0.005879	-5.35178	5.77139	6.54039	0.586824	-0.769	-1.70409
Camp	NM_009921	0.004404	-5.79672	4.9171	5.68768	0.586184	-0.77057	-1.70595
Nbas	NM_027706	0.001287	-8.05888	6.80747	7.57846	0.586016	-0.77099	-1.70644
Sec61b	NM_024171	0.002632	-6.66585	6.58229	7.35357	0.5859	-0.77128	-1.70678
Hoxb8	NM_010461	0.003055	-6.40343	4.76012	5.53172	0.585767	-0.7716	-1.70716
E130311K13Rik	BC125500	0.000725	-9.36295	6.43032	7.20202	0.585725	-0.77171	-1.70729
Ddx50	NM_053183	1.20E-05	-26.5042	8.50824	9.2802	0.58562	-0.77196	-1.70759
Gm13363	NR_002688	0.000376	-11.092	9.12066	9.8927	0.58559	-0.77204	-1.70768
	---	0.001695	-7.49464	3.22769	4.00027	0.585367	-0.77259	-1.70833
Reep5	NM_007874	0.000341	-11.3707	8.00581	8.77858	0.585292	-0.77277	-1.70855
Vill	NM_001164567	0.005783	-5.37636	5.38597	6.15895	0.585207	-0.77298	-1.7088
	---	0.000757	-9.25753	3.39449	4.16751	0.585191	-0.77302	-1.70884
Rtl1	NM_184109	0.0013	-8.03874	5.05217	5.82882	0.583719	-0.77665	-1.71315

Qsox1	NM_001024945	0.000628	-9.71791	9.38057	10.1578	0.583482	-0.77724	-1.71385
Adamts10	NM_172619	0.001291	-8.05257	6.312	7.08942	0.583412	-0.77741	-1.71405
Ssty2	NM_023546	0.002462	-6.7858	3.35442	4.13215	0.583283	-0.77773	-1.71443
Uggt2	NM_001081252	0.003973	-5.96249	7.80299	8.58101	0.583168	-0.77802	-1.71477
Ssty2	NM_023546	0.005603	-5.42367	3.06537	3.84343	0.58315	-0.77806	-1.71482
	---	3.19E-05	-20.7434	8.88303	9.66134	0.58305	-0.77831	-1.71512
Smpd2	NM_009213	0.002339	-6.88014	7.09906	7.87827	0.582686	-0.77921	-1.71619
Galnt2	NM_139272	0.001998	-7.17464	8.09585	8.87619	0.582231	-0.78034	-1.71753
Tmed8	NM_001033475	0.005858	-5.35715	5.70388	6.48538	0.581759	-0.78151	-1.71892
Tet1	NM_027384	0.0055	-5.45175	4.12637	4.9084	0.581546	-0.78203	-1.71955
Akap11	NM_001164503	0.005248	-5.52299	8.14317	8.92568	0.581357	-0.78251	-1.72011
Ssty2	NM_023546	0.005731	-5.38974	3.54474	4.32846	0.580867	-0.78372	-1.72157
	---	0.004484	-5.76801	5.57244	6.35675	0.580632	-0.7843	-1.72226
Dnaja4	NM_021422	0.000811	-9.09425	6.69647	7.48174	0.580246	-0.78526	-1.72341
Ifnar1	NM_010508	0.000158	-13.8421	7.72	8.50565	0.580093	-0.78565	-1.72386
Pja1	NM_001083110	0.003106	-6.37474	7.37155	8.15968	0.579094	-0.78813	-1.72683
Btbd6	NM_201646	0.000349	-11.3038	5.21733	6.00618	0.578803	-0.78886	-1.7277
Foxo3	NM_019740	0.001282	-8.06822	8.00728	8.79637	0.578709	-0.78909	-1.72799
Ifitm5	NM_053088	0.006916	-5.11378	6.07252	6.86178	0.57864	-0.78926	-1.72819
Prickle3	NM_175097	0.005982	-5.32592	4.81715	5.60659	0.578571	-0.78944	-1.7284
Rhobtb3	NM_028493	0.000458	-10.5419	7.32523	8.11508	0.578407	-0.78984	-1.72889
Pias2	NM_001164168	0.000414	-10.8222	7.70253	8.49374	0.577859	-0.79121	-1.73053
Rpl11	NM_025919	0.004354	-5.81485	9.04033	9.83226	0.577572	-0.79193	-1.73138
Kdr	NM_010612	0.005378	-5.48585	3.92253	4.71491	0.577392	-0.79238	-1.73193
Txndc5	NM_145367	0.000905	-8.83662	9.68875	10.4819	0.577086	-0.79314	-1.73284
Pls3	NM_145629	0.005877	-5.35229	9.84616	10.6405	0.576602	-0.79435	-1.7343
Gm7120	NM_001039244	0.004147	-5.8931	7.29665	8.09165	0.576342	-0.795	-1.73508
Ltbr	NM_010736	0.001672	-7.52218	7.25207	8.04713	0.576321	-0.79506	-1.73515
Mrpl24	NM_026591	0.006524	-5.19813	8.39495	9.19079	0.576008	-0.79584	-1.73609
Dpm2	NM_010073	0.003904	-5.99083	7.97171	8.768	0.575828	-0.79629	-1.73663
Gm1973	NM_029288	0.000131	-14.5176	3.10764	3.90443	0.575626	-0.7968	-1.73724

Gm10524	ENSMUST00000097503	0.003031	-6.41712	5.96461	6.76272	0.575102	-0.79811	-1.73882
Kcnma1	NM_010610	0.005371	-5.48782	4.10733	4.90562	0.57503	-0.79829	-1.73904
Fam36a	NM_025511	0.000244	-12.3834	6.43737	7.23612	0.574847	-0.79875	-1.73959
Tmcc2	NM_178874	0.002035	-7.14006	5.0029	5.80336	0.574169	-0.80045	-1.74165
Anks6	NM_001024136	0.002288	-6.92044	4.48188	5.28288	0.573949	-0.80101	-1.74231
Diap2	NM_172493	0.004852	-5.64429	6.39251	7.19454	0.573541	-0.80203	-1.74355
Fgfr1	NM_010206	0.003391	-6.22497	9.13051	9.93281	0.573435	-0.8023	-1.74388
Nln	NM_029447	0.003222	-6.31208	6.80705	7.61028	0.573064	-0.80323	-1.74501
Fzd7	NM_008057	0.002917	-6.48348	8.31789	9.12135	0.572972	-0.80346	-1.74528
Fut11	NM_028428	0.000187	-13.2585	7.34498	8.14927	0.572642	-0.80429	-1.74629
Stag1	NM_009282	0.000129	-14.5791	8.13958	8.94481	0.572271	-0.80523	-1.74742
Steap3	NM_001085409	0.001174	-8.25574	8.62457	9.43187	0.571448	-0.80731	-1.74994
Tmem167	NM_025335	0.005022	-5.59071	8.27106	9.07932	0.571071	-0.80826	-1.7511
Arl15	NM_172595	0.002164	-7.02374	7.24197	8.05079	0.570852	-0.80881	-1.75177
Ehd3	NM_020578	0.002104	-7.07703	5.54012	6.34946	0.570643	-0.80934	-1.75241
Slc25a1	NM_153150	0.000123	-14.755	7.65764	8.46698	0.570641	-0.80934	-1.75241
Pgm3	NM_028352	0.005026	-5.58943	6.72558	7.53807	0.569399	-0.81249	-1.75624
Npc2	NM_023409	0.001961	-7.21042	9.40582	10.2193	0.569022	-0.81344	-1.7574
Mir496	NR_030437	0.000944	-8.73974	3.02283	3.83661	0.568887	-0.81379	-1.75782
6720456H20Rik	NM_172600	0.000473	-10.4536	6.45283	7.26746	0.568553	-0.81463	-1.75885
Ssr1	NM_025965	0.002061	-7.11572	9.29653	10.1132	0.567769	-0.81662	-1.76128
Fam38a	NM_001037298	0.005104	-5.56589	7.76481	8.58299	0.567158	-0.81818	-1.76318
Tm9sf2	NM_080556	0.001512	-7.72465	9.52955	10.3478	0.567113	-0.81829	-1.76332
	---	0.003619	-6.11583	6.96873	7.7878	0.566808	-0.81907	-1.76427
Igsf8	NM_080419	0.000604	-9.81745	6.40397	7.22443	0.566265	-0.82045	-1.76596
Adamtsl1	NM_029967	0.00267	-6.64018	4.68161	5.50261	0.56605	-0.821	-1.76663
Lrrc49	NM_145616	0.001014	-8.57949	7.52544	8.34669	0.565952	-0.82125	-1.76693
Isca1	NM_026921	0.002777	-6.57035	8.02769	8.84948	0.56574	-0.82179	-1.7676
Cpt1c	NM_153679	0.004836	-5.64931	7.24462	8.06671	0.565621	-0.82209	-1.76797
Gpc6	NM_001079844	0.005257	-5.52044	8.20359	9.02645	0.565318	-0.82286	-1.76891

rp9	NM_018739	0.004651	-5.71027	6.16205	6.98603	0.564882	-0.82398	-1.77028
Lpgat1	NM_001134829	0.003103	-6.37643	7.85844	8.68355	0.564438	-0.82511	-1.77167
Gm3579	AY140896	0.006384	-5.22987	3.7509	4.57718	0.563981	-0.82628	-1.77311
Arsg	NM_028710	0.002579	-6.70216	5.19249	6.01889	0.563932	-0.82641	-1.77326
Zc3h12c	NM_001162921	0.000254	-12.2677	6.96599	7.79252	0.563884	-0.82653	-1.77341
Synpo2	NM_080451	0.001313	-8.01803	4.42007	5.24732	0.563599	-0.82726	-1.77431
C2cd2	NM_174847	0.003892	-5.99589	5.59396	6.42211	0.563252	-0.82815	-1.7754
Adam33	NM_033615	0.000815	-9.08256	5.6442	6.47235	0.563251	-0.82815	-1.77541
Prrc1	NM_028447	0.001071	-8.45666	7.34701	8.17556	0.563096	-0.82855	-1.77589
Serinc5	NM_172588	0.000983	-8.64781	8.04385	8.87433	0.562343	-0.83048	-1.77828
Ptpn9	NM_019651	0.001015	-8.57704	7.53885	8.37079	0.561771	-0.83195	-1.78008
Nrip1	NM_173440	0.000359	-11.2265	6.62155	7.45458	0.56135	-0.83303	-1.78142
Fmn1	NM_010230	0.001671	-7.52389	5.31411	6.14774	0.561117	-0.83363	-1.78216
	---	0.003938	-5.97683	7.04936	7.88367	0.560849	-0.83432	-1.78301
Mir381	NR_029882	0.000858	-8.96105	2.8024	3.63696	0.560753	-0.83456	-1.78332
Rab40b	NM_139147	0.004556	-5.74276	5.00616	5.84129	0.560532	-0.83513	-1.78402
Tmem39a	NM_026407	0.002838	-6.53218	9.37293	10.2105	0.55959	-0.83756	-1.78702
B4galt1	NM_022305	0.00013	-14.535	8.86526	9.7032	0.559441	-0.83794	-1.7875
Plxna2	NM_008882	0.002971	-6.45197	6.28973	7.12795	0.559332	-0.83822	-1.78785
Cmpk1	NM_025647	0.004627	-5.71859	7.86884	8.70819	0.558895	-0.83935	-1.78924
Emilin1	NM_133918	0.004003	-5.95028	8.65823	9.49884	0.558406	-0.84061	-1.79081
Gm8008	XM_001477516	0.004714	-5.68938	3.9543	4.79505	0.558353	-0.84075	-1.79098
Gm13251	NM_001085522	0.000272	-12.0542	8.88102	9.72226	0.558164	-0.84124	-1.79159
LOC100504530	XM_003085484	0.003076	-6.39177	3.25864	4.09991	0.558152	-0.84127	-1.79163
LOC100504530	XM_003085484	0.003076	-6.39177	3.25864	4.09991	0.558152	-0.84127	-1.79163
Rab18	NM_181070	0.003913	-5.98728	8.71148	9.55281	0.55813	-0.84133	-1.7917
2310008H04Rik	BC089026	0.006191	-5.27514	6.6411	7.48261	0.558058	-0.84151	-1.79193
Parp16	NM_177460	0.001852	-7.32143	5.06328	5.90681	0.557277	-0.84353	-1.79444
	---	0.004467	-5.7739	6.62295	7.46748	0.556892	-0.84453	-1.79568
Sntb2	NM_009229	0.003552	-6.14706	7.45142	8.29765	0.556234	-0.84624	-1.7978
Ehhadh	NM_023737	0.003864	-6.00794	4.09717	4.94422	0.555921	-0.84705	-1.79882

Gm2695	XR_106736	0.002305	-6.90664	7.39047	8.23784	0.555799	-0.84737	-1.79921
Tbc1d8b	NM_001081499	0.001738	-7.44521	8.01342	8.86276	0.555035	-0.84935	-1.80169
Atp7a	NM_001109757	0.001256	-8.11185	7.32636	8.17574	0.555024	-0.84938	-1.80173
D830030K20Rik	NM_177135	0.004156	-5.8895	2.51801	3.3675	0.554981	-0.84949	-1.80186
Fam102a	NM_153560	0.005705	-5.39672	6.56595	7.41737	0.554238	-0.85142	-1.80428
Gm1973	NM_029288	1.02E-05	-27.6301	3.72155	4.57402	0.553834	-0.85248	-1.8056
Gm1973	NM_029288	2.81E-05	-21.4191	3.10107	3.95469	0.553397	-0.85361	-1.80702
Gm1973	NM_029288	2.81E-05	-21.4191	3.10107	3.95469	0.553397	-0.85361	-1.80702
Tceal8	NM_001168578	0.000237	-12.4854	8.59779	9.4517	0.55328	-0.85392	-1.8074
Ssty2	NM_023546	0.003665	-6.09484	3.54961	4.40375	0.553195	-0.85414	-1.80768
	---	0.001124	-8.35072	7.32907	8.18354	0.553069	-0.85447	-1.80809
	---	8.04E-05	-16.4249	4.54472	5.39965	0.552894	-0.85492	-1.80866
Akap11	NM_001164503	0.002107	-7.07464	8.63686	9.49234	0.55268	-0.85549	-1.80937
Zmat3	NM_009517	0.001814	-7.36182	5.84923	6.70585	0.552247	-0.85662	-1.81079
Mrvi1	NM_010826	0.00085	-8.98228	4.23114	5.089	0.551769	-0.85786	-1.81235
Stat3	NM_213659	0.0023	-6.91086	9.22176	10.0817	0.550964	-0.85997	-1.815
Tspan6	NM_019656	0.003141	-6.35539	8.23321	9.0934	0.55088	-0.86019	-1.81528
	---	0.00113	-8.34033	9.21728	10.0784	0.550521	-0.86113	-1.81646
Cacnb1	NM_031173	0.001545	-7.68147	4.91656	5.77813	0.550351	-0.86158	-1.81702
Col23a1	NM_153393	0.001715	-7.47207	5.83525	6.69705	0.550266	-0.8618	-1.8173
Pde11a	NM_001081033	0.000148	-14.0729	4.60377	5.46615	0.550047	-0.86237	-1.81803
	---	0.006665	-5.16709	9.51776	10.3806	0.549882	-0.86281	-1.81857
Acaa1a	NM_130864	0.004825	-5.65302	6.84996	7.71319	0.54972	-0.86323	-1.81911
Sik2	NM_178710	2.29E-05	-22.5492	6.87921	7.74309	0.549472	-0.86388	-1.81993
Ifitm2	NM_030694	0.003902	-5.99196	10.0931	10.9573	0.549356	-0.86419	-1.82031
Mir145	NR_029557	0.005395	-5.48112	5.87656	6.74108	0.549228	-0.86452	-1.82074
Gpr180	NM_021434	0.003486	-6.17841	6.56061	7.42523	0.549192	-0.86462	-1.82086
Map4k4	NM_008696	6.34E-05	-17.4418	8.16915	9.03447	0.548924	-0.86532	-1.82175
Ubtd2	NM_173784	0.001619	-7.58636	8.2724	9.13808	0.548787	-0.86568	-1.8222
Npc1	NM_008720	0.00061	-9.79095	7.91234	8.77806	0.54877	-0.86573	-1.82226
Fbxl7	BC050864	0.000354	-11.2666	3.43841	4.30457	0.548607	-0.86616	-1.8228

Slc38a10	NM_024249	0.003856	-6.01112	8.13419	9.00058	0.548516	-0.86639	-1.8231
Nup93	NM_172410	0.002027	-7.14735	8.41614	9.28397	0.547969	-0.86783	-1.82492
Ssty2	NM_023546	0.005712	-5.39489	3.30081	4.1692	0.547755	-0.8684	-1.82563
Gm10035	NM_001081471	0.00079	-9.15693	5.6535	6.52217	0.547653	-0.86867	-1.82597
Ankrd57	NM_172939	0.000396	-10.947	5.67037	6.53956	0.547457	-0.86918	-1.82663
Cln3	NM_001146311	0.005596	-5.42557	5.94843	6.8189	0.54697	-0.87047	-1.82826
Ankrd44	NM_001081433	0.003622	-6.11475	5.75578	6.62639	0.546916	-0.87061	-1.82843
Dio2	NM_010050	0.000706	-9.42669	4.15191	5.02435	0.546224	-0.87244	-1.83075
Cep350	NM_001039184	0.000181	-13.3677	5.0264	5.90057	0.545568	-0.87417	-1.83295
Crem	NM_001110856	0.006286	-5.25266	4.75252	5.62728	0.545345	-0.87476	-1.8337
Fam158a	AB054001	0.001341	-7.97246	5.66261	6.53806	0.545085	-0.87545	-1.83458
Nr4a3	NM_015743	0.004099	-5.91169	4.87806	5.75372	0.545004	-0.87566	-1.83485
Xk	NM_023500	0.003138	-6.35733	4.47382	5.34967	0.54493	-0.87586	-1.8351
	---	0.005847	-5.3598	6.20831	7.08726	0.543762	-0.87895	-1.83904
Zfp395	NM_199029	0.003159	-6.3459	6.32503	7.2041	0.543718	-0.87907	-1.83919
Higd1a	NM_019814	0.002596	-6.69016	5.03199	5.91132	0.543621	-0.87933	-1.83952
	---	0.000662	-9.5849	9.04545	9.92533	0.543413	-0.87988	-1.84022
D14Ertd449e	NM_025311	0.000877	-8.90975	6.93729	7.81987	0.542394	-0.88259	-1.84368
Mbnl2	NM_175341	0.002062	-7.11507	9.71312	10.5963	0.542163	-0.8832	-1.84446
Ankrd6	NM_001012450	0.004646	-5.71217	5.03387	5.91727	0.542088	-0.8834	-1.84472
Rpl11	NM_025919	0.003278	-6.28233	10.2543	11.1377	0.542079	-0.88343	-1.84475
Zcchc3	NM_175126	0.000111	-15.1537	4.65985	5.54345	0.542013	-0.8836	-1.84498
LOC100040031	BC089467	0.002183	-7.00815	3.63625	4.52048	0.541775	-0.88423	-1.84578
Fbln2	NM_007992	0.003311	-6.26539	10.7665	11.6518	0.54138	-0.88529	-1.84713
L3mbtl3	NM_172787	6.97E-05	-17.0331	7.35421	8.2397	0.541302	-0.88549	-1.8474
Isca1	NM_026921	0.002123	-7.06024	8.57942	9.46508	0.541238	-0.88567	-1.84762
D14Ertd449e	NM_025311	0.000826	-9.0504	7.43062	8.31668	0.54109	-0.88606	-1.84812
Rpl11	NM_025919	0.001499	-7.74236	10.6535	11.54	0.540916	-0.88652	-1.84872
	---	0.00662	-5.17707	3.06984	3.95694	0.5407	-0.8871	-1.84946
Igfbp7	NM_001159518	0.001954	-7.21814	10.1533	11.0406	0.54065	-0.88723	-1.84963
Rps12	NM_011295	0.004722	-5.68666	10.3816	11.2692	0.540524	-0.88757	-1.85006

D14Ertd449e	NM_025311	0.00077	-9.21769	7.26162	8.15088	0.539889	-0.88926	-1.85223
Abhd14a	NM_001110271	0.000856	-8.96653	5.96335	6.85398	0.539376	-0.89064	-1.85399
Akap11	NM_001164503	0.001825	-7.34976	7.99491	8.88583	0.539269	-0.89092	-1.85436
Nod1	NM_172729	0.002024	-7.14999	5.80076	6.6926	0.538928	-0.89184	-1.85554
Rps12	NM_011295	0.005446	-5.46671	10.2169	11.1102	0.538411	-0.89322	-1.85732
Carm1	NM_021531	0.000477	-10.4338	7.20834	8.10259	0.538028	-0.89425	-1.85864
Zfp9	NM_011763	4.76E-05	-18.757	6.02037	6.9147	0.537997	-0.89433	-1.85875
Zadh2	NM_146090	0.000855	-8.97015	7.55453	8.44894	0.537968	-0.89441	-1.85885
Yipf5	NM_023311	0.001496	-7.74622	7.87906	8.77394	0.537793	-0.89488	-1.85945
Lmna	NM_001002011	0.00033	-11.471	8.42439	9.31933	0.537771	-0.89494	-1.85953
Rps12	NM_011295	0.005646	-5.41228	10.4501	11.3492	0.536223	-0.8991	-1.8649
LOC100039753	NM_001017394	0.005293	-5.50999	3.47412	4.37324	0.536212	-0.89912	-1.86493
Rps12	NM_011295	0.003581	-6.1334	10.2456	11.1449	0.536126	-0.89936	-1.86523
Prr5	NM_146061	0.000446	-10.6113	5.87343	6.77306	0.536025	-0.89963	-1.86559
Naaladl2	ENSMUST00000099184	0.000105	-15.3512	3.73465	4.63585	0.53544	-0.9012	-1.86762
Rps12	NM_011295	0.005086	-5.57118	10.7455	11.6478	0.535029	-0.90231	-1.86906
Igdcc4	NM_020043	0.006848	-5.1279	4.72359	5.62641	0.534841	-0.90282	-1.86971
Zbtb4	NM_029348	0.003743	-6.06032	5.82986	6.73531	0.533868	-0.90545	-1.87312
Pqlc3	NM_172574	0.006997	-5.09698	7.61887	8.52559	0.533394	-0.90673	-1.87479
Acpl2	NM_153420	0.00301	-6.42932	6.11898	7.0263	0.533177	-0.90731	-1.87555
Acot6	NM_172580	0.001004	-8.60104	3.82643	4.73458	0.532866	-0.90816	-1.87664
Tead3	NM_001098226	0.001769	-7.41095	6.25516	7.16535	0.532117	-0.91019	-1.87929
Ppp1r3b	NM_177741	5.57E-05	-18.0224	4.78259	5.69331	0.531919	-0.91072	-1.87999
Sh3kbp1	NM_021389	0.001642	-7.55789	8.24575	9.15669	0.531838	-0.91094	-1.88027
D830030K20Rik	NM_177135	0.00651	-5.20143	3.20707	4.11892	0.531503	-0.91185	-1.88146
Rpl11	NM_025919	0.002302	-6.90941	10.7628	11.6769	0.530695	-0.91404	-1.88432
Sptlc2	NM_011479	0.000255	-12.249	8.45977	9.37399	0.530629	-0.91422	-1.88456
1700025G04Rik	NM_197990	0.001771	-7.40861	4.90578	5.82061	0.530405	-0.91483	-1.88535
	---	0.0047	-5.6938	9.33252	10.2479	0.530221	-0.91534	-1.88601
Slc37a4	NM_008063	0.002226	-6.97138	5.66949	6.58486	0.530207	-0.91537	-1.88605

Jph1	NM_020604	0.001649	-7.55032	5.22841	6.14449	0.529946	-0.91608	-1.88698
Lypla2	NM_011942	0.006097	-5.29773	6.59624	7.51298	0.529704	-0.91674	-1.88785
	---	0.004159	-5.88837	4.45997	5.37704	0.529583	-0.91707	-1.88828
Scap	NM_001001144	0.00304	-6.41173	7.74019	8.65847	0.52914	-0.91828	-1.88986
	---	0.000336	-11.4176	2.8214	3.73976	0.529111	-0.91836	-1.88996
Adam17	NM_009615	0.00068	-9.5207	8.66201	9.58076	0.528966	-0.91875	-1.89048
Fbxo17	NM_015796	0.002797	-6.55723	4.84246	5.7623	0.528569	-0.91984	-1.8919
Golga7	NM_020585	0.006042	-5.31112	8.96329	9.88434	0.528125	-0.92105	-1.89349
	---	0.006048	-5.30946	4.01094	4.93442	0.527236	-0.92348	-1.89668
Bmyc	NM_023326	8.91E-05	-16.0045	5.63387	6.55797	0.527009	-0.9241	-1.8975
Chmp1a	NM_145606	0.001292	-8.05166	7.54314	8.46748	0.526921	-0.92434	-1.89782
Icosl	NM_015790	0.006331	-5.24207	4.90874	5.8334	0.526804	-0.92466	-1.89824
Tcf12	NM_011544	0.000182	-13.348	8.88722	9.81199	0.526763	-0.92477	-1.89839
Ndufc2	NM_024220	0.000379	-11.0654	8.98035	9.90595	0.526461	-0.9256	-1.89948
Osgep	NM_133676	0.0011	-8.39873	6.17314	7.10138	0.5255	-0.92824	-1.90295
Mir409	NR_029913	0.000951	-8.72382	3.99038	4.91896	0.525374	-0.92858	-1.90341
Cdk7	NM_009874	0.001985	-7.18714	6.57093	7.50219	0.524398	-0.93127	-1.90695
Cd164	NM_016898	0.004132	-5.89885	10.5155	11.4486	0.523716	-0.93314	-1.90943
Soat1	NM_009230	0.003816	-6.0284	8.56053	9.49422	0.52352	-0.93368	-1.91015
Lage3	NM_025410	0.006635	-5.17362	5.66112	6.5956	0.523231	-0.93448	-1.9112
Irak1bp1	NM_022986	0.000223	-12.6839	6.41414	7.34867	0.523211	-0.93454	-1.91128
	---	0.006733	-5.15236	2.45453	3.39089	0.522552	-0.93635	-1.91369
LOC100039753	NM_001017394	0.003039	-6.41267	3.53361	4.47059	0.522325	-0.93698	-1.91452
Galnt13	NM_173030	0.006182	-5.27724	3.14241	4.07967	0.522223	-0.93726	-1.91489
Rps12	NM_011295	0.006181	-5.27752	10.4682	11.4073	0.521561	-0.93909	-1.91732
Chchd10	NM_175329	0.00595	-5.33369	5.32786	6.26764	0.521312	-0.93978	-1.91824
Csrp1	NM_007791	9.65E-06	-28.0189	9.91863	10.8597	0.520863	-0.94102	-1.91989
Ssr4	NM_001166480	0.000738	-9.3197	8.72414	9.66658	0.520352	-0.94244	-1.92178
Fam38a	NM_001037298	0.002685	-6.6298	7.75287	8.69557	0.520259	-0.9427	-1.92212
Pacs1	NM_153129	0.000166	-13.6568	5.48626	6.42953	0.520055	-0.94326	-1.92287
Ank2	NM_178655	0.000561	-10.0051	5.99785	6.94137	0.519961	-0.94352	-1.92322

Rsph9	NM_029338	0.003429	-6.20644	4.65355	5.59798	0.519636	-0.94443	-1.92442
Lancl3	NM_173414	0.002403	-6.83039	4.45503	5.39991	0.519475	-0.94487	-1.92502
Il17ra	NM_008359	0.001531	-7.69897	7.36517	8.31089	0.519173	-0.94571	-1.92614
Fam55d	BC094249	0.000872	-8.92451	5.95595	6.9018	0.519125	-0.94585	-1.92632
Cnrip1	NM_029861	0.001584	-7.63091	5.76574	6.71273	0.518716	-0.94698	-1.92784
Nr1h3	NM_013839	0.006736	-5.15176	4.7875	5.73731	0.517698	-0.94982	-1.93163
Galt	NM_016658	0.002281	-6.92604	5.92514	6.87549	0.517507	-0.95035	-1.93234
Rilpl2	NM_030259	0.004744	-5.67923	5.40227	6.35283	0.51743	-0.95057	-1.93263
S1pr2	NM_010333	0.000517	-10.2182	6.70884	7.66136	0.516731	-0.95252	-1.93524
Gabre	NM_017369	0.003776	-6.04555	3.38973	4.34306	0.516438	-0.95333	-1.93634
Nus1	NM_030250	0.000615	-9.76798	8.84996	9.80345	0.516382	-0.95349	-1.93655
Nagk	NM_019542	0.005806	-5.37042	6.93618	7.89004	0.516252	-0.95385	-1.93704
Eif1b	NM_026892	0.006777	-5.14297	7.09065	8.04504	0.516056	-0.9544	-1.93777
Nlrx1	NM_178420	0.001448	-7.81336	5.47464	6.43494	0.513951	-0.9603	-1.94571
Palld	NM_001081390	0.003132	-6.36055	6.48804	7.44986	0.513408	-0.96182	-1.94777
Igsf3	NM_207205	0.004294	-5.83707	6.56438	7.52717	0.513065	-0.96279	-1.94907
9930014A18Rik	NR_030696	0.002437	-6.80466	5.56588	6.52894	0.512967	-0.96306	-1.94944
D030025P21Rik	NR_028577	0.001642	-7.55893	4.61977	5.58322	0.512828	-0.96345	-1.94997
Yipf5	NM_023311	0.000442	-10.639	8.72223	9.68665	0.512488	-0.96441	-1.95127
Sra1	NM_025291	0.000558	-10.0203	7.9275	8.89275	0.51219	-0.96525	-1.9524
Trp53	NM_001127233	0.002867	-6.51413	7.32209	8.28776	0.512041	-0.96567	-1.95297
Add3	NM_001164099	0.000303	-11.7187	7.95825	8.92473	0.511753	-0.96648	-1.95407
Mycbp2	NM_207215	0.001567	-7.65274	7.1247	8.0924	0.51132	-0.9677	-1.95572
Arid5b	NM_023598	0.000857	-8.96456	8.6256	9.59348	0.511256	-0.96788	-1.95597
Ifnar2	NM_010509	0.002194	-6.99857	7.74765	8.71895	0.510049	-0.97129	-1.9606
Sh3bp1	NM_009164	0.000402	-10.9026	5.76082	6.73214	0.51004	-0.97132	-1.96063
Gdf1	NM_001163282	0.002348	-6.87305	5.62287	6.59536	0.509625	-0.97249	-1.96223
Gmppb	NM_177910	0.004717	-5.68816	5.39448	6.36711	0.509577	-0.97263	-1.96241
Cdh18	NM_001081299	0.002227	-6.97031	4.10351	5.07637	0.509494	-0.97286	-1.96273
Osgep	NM_133676	0.000746	-9.29209	6.94666	7.92118	0.508907	-0.97453	-1.96499
	---	0.002683	-6.63106	4.58499	5.56143	0.508231	-0.97644	-1.96761

Bbox1	NM_130452	0.000617	-9.76327	3.53471	4.51245	0.507773	-0.97774	-1.96938
Gng10	NM_025277	0.004114	-5.90566	7.87324	8.85134	0.507648	-0.9781	-1.96987
Fzd8	NM_008058	0.000246	-12.3598	6.36923	7.34742	0.507616	-0.97819	-1.96999
Dnm3os	NR_002870	0.000655	-9.61313	5.90076	6.87964	0.507375	-0.97888	-1.97093
Rab9	NM_019773	0.004605	-5.72599	6.53874	7.51787	0.507284	-0.97913	-1.97128
Myd88	NM_010851	0.001587	-7.62655	7.82579	8.80655	0.506713	-0.98076	-1.9735
Dnajc27	NM_153082	0.001582	-7.63307	3.9217	4.90266	0.506641	-0.98096	-1.97378
Hebp2	NM_019487	0.000977	-8.66177	5.46858	6.44966	0.506601	-0.98108	-1.97394
Pkdcc	NM_134117	0.006298	-5.24987	5.38222	6.36367	0.506471	-0.98145	-1.97445
Uhrf2	NM_144873	0.00152	-7.71412	8.43358	9.41695	0.505797	-0.98337	-1.97708
Hnrnph3	NM_001079824	9.75E-05	-15.6457	7.46945	8.4529	0.505767	-0.98346	-1.9772
	---	0.000892	-8.86997	3.10076	4.08444	0.505689	-0.98368	-1.9775
	---	0.00117	-8.26445	4.63412	5.61996	0.504931	-0.98584	-1.98047
Rpl11	NM_025919	0.004647	-5.71159	10.4281	11.4177	0.503624	-0.98958	-1.98561
Arl5b	NM_029466	0.002723	-6.60487	7.01395	8.00405	0.503444	-0.9901	-1.98632
LOC100039753	NM_001017394	0.001113	-8.37238	3.61343	4.60382	0.503341	-0.99039	-1.98673
Rps2	NM_008503	0.000151	-14.0007	9.94864	10.9394	0.503207	-0.99078	-1.98725
Cog3	NM_177381	0.000741	-9.3097	7.37341	8.36509	0.502891	-0.99168	-1.9885
	---	0.005979	-5.32661	10.0595	11.0513	0.502862	-0.99176	-1.98862
Gdf10	NM_145741	0.006018	-5.31692	5.55628	6.5486	0.502669	-0.99232	-1.98938
Dchs1	NM_001162943	0.004803	-5.65999	4.64949	5.64372	0.502003	-0.99423	-1.99202
Mapk13	NM_011950	0.000765	-9.23134	5.33472	6.32959	0.501781	-0.99487	-1.9929
Nit2	NM_023175	0.002633	-6.66494	6.60578	7.60104	0.501643	-0.99527	-1.99345
Mfsd9	NM_172499	0.000855	-8.97041	4.95118	5.94953	0.500574	-0.99834	-1.99771
Litaf	NM_019980	0.003076	-6.39167	8.46206	9.4611	0.500335	-0.99903	-1.99866
Rplp1	BC091747	0.000148	-14.0798	9.65349	10.6528	0.500257	-0.99926	-1.99897
Ifitm2	NM_030694	0.004087	-5.91653	10.1341	11.1334	0.500251	-0.99928	-1.999
Papd4	NM_133905	2.78E-05	-21.4796	7.8928	8.89352	0.499748	-1.00073	-2.00101
Pdia5	NM_028295	0.000318	-11.5809	7.79895	8.8002	0.499566	-1.00125	-2.00174
Arl5c	NM_207231	0.002541	-6.72883	4.9993	6.00235	0.498943	-1.00305	-2.00424
	---	0.002462	-6.78642	5.83862	6.84287	0.498529	-1.00425	-2.0059

Adck4	NM_133770	0.002561	-6.71462	6.86778	7.87331	0.498089	-1.00552	-2.00767
Gspt2	NM_008179	0.002687	-6.62856	3.89096	4.89683	0.497972	-1.00586	-2.00814
Oat	NM_016978	0.000143	-14.1867	9.49681	10.5032	0.497786	-1.0064	-2.0089
2810405K02Rik	BC030453	0.000444	-10.629	4.68597	5.69287	0.497614	-1.0069	-2.00959
Gnpda2	NM_001038015	0.00128	-8.0716	6.40739	7.41569	0.497133	-1.0083	-2.01154
	---	0.002939	-6.47043	2.72025	3.72932	0.496867	-1.00907	-2.01261
Spin4	NM_178753	0.000532	-10.1441	5.86468	6.87634	0.495977	-1.01165	-2.01622
P4htm	NM_028944	0.002105	-7.07618	5.13185	6.14388	0.49585	-1.01203	-2.01674
Ddhd1	NM_001042719	6.61E-06	-30.813	7.66952	8.68366	0.495124	-1.01414	-2.0197
	---	0.005556	-5.4364	7.94349	8.95809	0.494967	-1.0146	-2.02034
Rcbtb2	NM_001170694	0.000645	-9.64953	6.66975	7.68497	0.494753	-1.01522	-2.02121
Capn1	NM_007600	4.39E-06	-34.1385	7.83994	8.85527	0.494715	-1.01533	-2.02137
Zfp874a	NM_177712	0.002791	-6.56105	4.48449	5.50205	0.49395	-1.01756	-2.0245
Rpia	NM_009075	7.09E-05	-16.9588	5.4804	6.4995	0.493423	-1.0191	-2.02666
Riok3	NM_024182	4.51E-05	-19.0082	7.88516	8.90609	0.492802	-1.02092	-2.02921
Dzip1	NM_025943	0.001149	-8.30391	5.26611	6.28874	0.492218	-1.02263	-2.03162
Spry4	NM_011898	0.003468	-6.18713	5.3333	6.35938	0.491041	-1.02609	-2.03649
	---	0.002801	-6.55479	4.00119	5.02895	0.490471	-1.02776	-2.03886
LOC100039753	NM_001017394	0.001861	-7.31142	3.77574	4.80402	0.490293	-1.02828	-2.0396
BC013529	NM_145418	0.0014	-7.88261	7.62926	8.65905	0.489781	-1.02979	-2.04173
Rgl3	NM_023622	0.000465	-10.5028	4.90279	5.93316	0.489584	-1.03037	-2.04255
1110032A13Rik	NM_199197	0.000138	-14.324	6.16477	7.19658	0.489097	-1.03181	-2.04458
Ank3	NM_146005	0.000948	-8.73153	5.91892	6.95141	0.488868	-1.03248	-2.04554
B3galnt2	NM_178640	0.003576	-6.13599	7.86163	8.89661	0.488025	-1.03497	-2.04907
Gas5	NR_002840	0.001152	-8.29732	9.63288	10.6689	0.487661	-1.03605	-2.05061
Chst11	NM_021439	0.004988	-5.60118	7.58617	8.62535	0.486605	-1.03918	-2.05506
Tmem38b	NM_028053	0.002165	-7.02326	5.11303	6.15346	0.48618	-1.04044	-2.05685
	---	0.005734	-5.38906	4.54087	5.5823	0.485848	-1.04142	-2.05826
Spry3	NM_001030293	0.004959	-5.61025	3.75696	4.79862	0.485767	-1.04166	-2.0586
Tmem159	NM_145586	0.002454	-6.79165	7.32051	8.3628	0.485556	-1.04229	-2.0595
Cpne8	NM_025815	0.007038	-5.0887	6.11114	7.154	0.485364	-1.04286	-2.06031

Cpa6	NM_177834	7.77E-05	-16.5723	3.41407	4.45719	0.485274	-1.04313	-2.06069
Prdx4	NM_016764	0.000379	-11.0695	7.9263	8.97077	0.484823	-1.04447	-2.06261
Tmem205	NM_178577	0.000795	-9.1395	6.68185	7.72883	0.483979	-1.04698	-2.06621
	---	0.004427	-5.78823	3.20428	4.25177	0.483812	-1.04748	-2.06692
Sh3bgrl3	NM_080559	0.001044	-8.51493	8.70582	9.75467	0.483352	-1.04885	-2.06888
Adora2b	NM_007413	0.000586	-9.89287	5.61706	6.66709	0.482958	-1.05003	-2.07057
Fam38a	NM_001037298	0.002568	-6.70964	7.63749	8.68781	0.482863	-1.05031	-2.07098
Rps19	NM_023133	0.006504	-5.20282	7.56623	8.61677	0.482789	-1.05053	-2.0713
Rps19	NM_023133	0.006504	-5.20282	7.56623	8.61677	0.482789	-1.05053	-2.0713
Rps27l	NM_026467	0.001846	-7.32737	7.60098	8.65352	0.482117	-1.05255	-2.07419
Zfp354c	NM_013922	0.002717	-6.60902	7.0805	8.13557	0.481273	-1.05507	-2.07782
Stat5a	NM_011488	0.004599	-5.72812	6.09014	7.1457	0.481109	-1.05556	-2.07853
Slc9a9	NM_177909	4.74E-06	-33.4891	6.55761	7.61351	0.480997	-1.0559	-2.07901
Gaa	NM_008064	0.005122	-5.5603	8.96874	10.025	0.480877	-1.05626	-2.07953
Slc7a7	NM_011405	0.004971	-5.60671	4.67144	5.72841	0.480641	-1.05697	-2.08055
A430110N23Rik	NM_173008	0.001257	-8.10907	6.95611	8.01372	0.480425	-1.05762	-2.08149
Rexo2	NM_024233	0.000148	-14.0711	9.00714	10.0648	0.480423	-1.05762	-2.0815
	---	0.003902	-5.99198	8.31825	9.37599	0.480385	-1.05774	-2.08166
Arsb	NM_009712	0.005318	-5.50293	7.72698	8.78498	0.480296	-1.058	-2.08205
Maf	NM_001025577	0.000447	-10.6052	6.93366	7.99416	0.479464	-1.06051	-2.08566
Fuca1	NM_024243	0.00694	-5.1088	8.03371	9.09618	0.47881	-1.06248	-2.08851
Alkbh2	NM_175016	0.005167	-5.5471	5.7054	6.76834	0.478656	-1.06294	-2.08918
Rev3l	NM_011264	0.000649	-9.63536	7.75874	8.82466	0.477668	-1.06592	-2.0935
	---	0.003004	-6.43237	3.62718	4.6932	0.477634	-1.06602	-2.09365
Eefsec	NM_023060	0.005582	-5.42932	6.59707	7.66364	0.477456	-1.06656	-2.09444
Zeb1	NM_011546	0.000701	-9.44504	8.25601	9.32269	0.477416	-1.06668	-2.09461
Ifitm2	NM_030694	0.004175	-5.88208	9.69935	10.7679	0.476812	-1.06851	-2.09726
Trmt61a	NM_177374	0.001369	-7.93013	5.75021	6.81958	0.476529	-1.06936	-2.09851
Wipf1	NM_153138	0.000154	-13.9251	5.80628	6.87598	0.476418	-1.0697	-2.099
Tgfbr1	NM_009370	0.000165	-13.6915	8.15416	9.22531	0.47594	-1.07115	-2.1011
Gpr88	NM_022427	0.001485	-7.76238	5.45815	6.52956	0.475854	-1.07141	-2.10149

9030617O03Rik	BC066161	0.000822	-9.06254	4.99075	6.06852	0.473762	-1.07777	-2.11076
Bmpr1b	NM_007560	2.03E-06	-41.4476	3.77498	4.85435	0.473235	-1.07937	-2.11312
Fcgrt	NM_010189	0.002237	-6.96224	9.27295	10.3528	0.473074	-1.07986	-2.11383
LOC100039147	XM_003085637	0.001026	-8.55259	3.92529	5.00545	0.472975	-1.08016	-2.11428
Cask	NM_009806	0.00456	-5.74141	8.05131	9.13268	0.472581	-1.08137	-2.11604
Zim1	NM_011769	0.006351	-5.2376	3.77478	4.8563	0.472532	-1.08152	-2.11626
Tspan4	NM_053082	8.79E-05	-16.0597	9.26213	10.3448	0.472162	-1.08265	-2.11792
Gpc4	NM_008150	0.000251	-12.2991	8.01274	9.09599	0.471963	-1.08325	-2.11881
Vaultrc5	NR_027885	0.001607	-7.60172	5.58105	6.66513	0.471694	-1.08408	-2.12002
Leprot	NM_175036	0.000128	-14.5901	7.63753	8.72216	0.471512	-1.08463	-2.12084
Sema6a	NM_018744	0.000885	-8.8886	4.74068	5.82664	0.471078	-1.08596	-2.12279
Zfpm2	NM_011766	0.004058	-5.928	5.60542	6.69327	0.470461	-1.08785	-2.12558
Cish	NM_009895	0.004046	-5.93261	4.97859	6.06683	0.470337	-1.08823	-2.12613
Chst12	NM_021528	0.002437	-6.80445	8.22691	9.3159	0.470092	-1.08899	-2.12724
Tifa	NM_145133	0.0035	-6.17185	6.72728	7.81809	0.469498	-1.09081	-2.12994
Prorsd1	NM_001163454	0.001247	-8.12662	5.89803	6.99011	0.469084	-1.09208	-2.13182
Gnpnat1	NM_019425	0.00035	-11.2964	7.33473	8.42684	0.469075	-1.09211	-2.13186
Higd1a	NM_019814	0.001607	-7.60206	5.75702	6.85118	0.468408	-1.09416	-2.13489
Kdelr3	NM_134090	0.001409	-7.86961	8.54438	9.63973	0.468021	-1.09535	-2.13665
Mmp14	NM_008608	0.000349	-11.3049	10.952	12.0476	0.467932	-1.09563	-2.13706
Mfsd10	NM_026660	0.000149	-14.0503	7.84753	8.94423	0.467582	-1.09671	-2.13866
Lrp1	NM_008512	0.00042	-10.7777	10.2086	11.3054	0.467526	-1.09688	-2.13892
Dsel	NM_001081316	0.002805	-6.55222	6.72983	7.82756	0.467253	-1.09772	-2.14017
	---	0.003755	-6.05495	3.6929	4.79334	0.466372	-1.10045	-2.14421
Rpl11	NM_025919	0.002629	-6.66786	9.96143	11.0627	0.466112	-1.10125	-2.14541
Fhl2	NM_010212	0.001964	-7.20755	6.24542	7.34697	0.466017	-1.10155	-2.14584
	---	0.000692	-9.47756	5.23812	6.33988	0.465947	-1.10176	-2.14617
Pkd2	NM_008861	0.000445	-10.6188	8.72667	9.82912	0.465723	-1.10246	-2.1472
	---	0.00097	-8.6792	5.25707	6.36108	0.46522	-1.10401	-2.14952
Gm1973	NM_029288	0.002071	-7.10724	3.20873	4.31306	0.465117	-1.10433	-2.15
Rbm47	NM_178446	0.004358	-5.81342	4.63179	5.73636	0.465041	-1.10457	-2.15035

Egfr	NM_207655	0.000903	-8.84254	8.66975	9.77558	0.464636	-1.10583	-2.15222
Mettl7a1	NM_027334	0.001931	-7.24047	7.29385	8.39986	0.464577	-1.10601	-2.1525
Wbp5	NM_011712	8.15E-05	-16.371	8.88711	9.99787	0.463049	-1.11076	-2.1596
Rps12	AF357393	0.001466	-7.7882	6.06425	7.17527	0.462968	-1.11102	-2.15998
1500011K16Rik	NR_015476	0.000761	-9.24349	5.74795	6.85916	0.462905	-1.11121	-2.16027
Podxl2	NM_176973	0.000821	-9.06551	4.61516	5.72645	0.46288	-1.11129	-2.16038
Frem1	NM_177863	0.004945	-5.61464	4.02472	5.1364	0.462755	-1.11168	-2.16097
Pcdh7	NM_018764	0.000544	-10.0826	7.68276	8.79471	0.462666	-1.11196	-2.16139
Lgals3bp	NM_011150	0.001504	-7.73584	8.9226	10.0386	0.461386	-1.11595	-2.16738
S100a13	NM_009113	0.000247	-12.3548	6.10687	7.223	0.46133	-1.11613	-2.16765
Tnfrsf1a	NM_011609	0.000606	-9.80767	9.242	10.3585	0.461226	-1.11646	-2.16814
Rassf8	NM_027760	3.66E-05	-20.041	7.06977	8.19286	0.45911	-1.12309	-2.17813
Armcx3	NM_027870	0.000788	-9.16128	9.49989	10.6232	0.459041	-1.12331	-2.17846
Pcdhga12	NM_033595	3.52E-05	-20.2309	5.39835	6.52211	0.458899	-1.12375	-2.17913
Rgma	NM_177740	0.001559	-7.66313	6.82886	7.95384	0.458508	-1.12498	-2.18099
Hif1a	NM_010431	0.000257	-12.2246	8.97993	10.1055	0.458313	-1.1256	-2.18192
Pappa2	NM_001085376	0.005633	-5.41569	4.30351	5.42932	0.458247	-1.1258	-2.18223
	---	0.00068	-9.51839	6.72402	7.85062	0.457994	-1.1266	-2.18344
Gnpnat1	NM_019425	0.000292	-11.8332	7.04328	8.1738	0.456754	-1.13051	-2.18936
Ptp4a3	NM_008975	0.000604	-9.81683	5.72038	6.85213	0.456362	-1.13175	-2.19124
Trim30d	NM_199146	0.004612	-5.72353	4.38402	5.51582	0.456348	-1.13179	-2.19131
Ldoc1l	NM_177630	0.00201	-7.16321	4.61477	5.74946	0.455434	-1.13468	-2.19571
Adcy9	NM_009624	0.001504	-7.73546	4.6877	5.82627	0.454209	-1.13857	-2.20163
Psen2	NM_011183	0.000224	-12.6613	6.27866	7.41781	0.454027	-1.13915	-2.20251
Armcx3	NM_027870	0.000539	-10.1097	9.03518	10.1765	0.453338	-1.14134	-2.20586
Il7	NM_008371	0.000577	-9.93133	3.2943	4.43962	0.452091	-1.14531	-2.21194
Marcks	NM_008538	0.001501	-7.73936	9.45469	10.6006	0.451918	-1.14587	-2.21279
Gla	NM_013463	0.001106	-8.38624	8.31883	9.46495	0.451838	-1.14612	-2.21318
Reck	NM_016678	0.000258	-12.2083	6.90529	8.0525	0.451496	-1.14722	-2.21486
Clybl	NM_029556	0.000431	-10.7081	6.87749	8.02578	0.45116	-1.14829	-2.21651
Ifitm1	NM_026820	0.004207	-5.86995	5.32349	6.4722	0.451029	-1.14871	-2.21715

Tmem200a	NM_029881	0.001947	-7.22415	5.05616	6.20613	0.450634	-1.14997	-2.2191
Ifih1	NM_027835	0.002187	-7.00449	3.91077	5.06123	0.450482	-1.15046	-2.21984
Ahi1	NM_026203	0.000984	-8.64773	6.40873	7.56048	0.450077	-1.15175	-2.22184
Mir541	NR_030263	0.002221	-6.97565	3.1695	4.32152	0.449996	-1.15202	-2.22224
Fam69b	NM_019833	0.000949	-8.72781	5.59918	6.75264	0.449545	-1.15346	-2.22447
D830030K20Rik	NM_177135	0.002953	-6.46208	3.1852	4.34117	0.448763	-1.15598	-2.22835
Satb1	NM_009122	0.005445	-5.46706	4.16037	5.31791	0.448277	-1.15754	-2.23076
Gypc	NM_001048207	0.000352	-11.2823	6.64398	7.80237	0.448013	-1.15839	-2.23208
Hoxb6	NM_008269	0.004294	-5.8369	6.55854	7.72388	0.445859	-1.16534	-2.24286
Atp2b4	NM_001167949	0.000397	-10.9398	4.54471	5.7137	0.444731	-1.169	-2.24855
Armcx3	NM_027870	0.001762	-7.41814	8.7583	9.92769	0.444612	-1.16938	-2.24915
Foxg1	NM_008241	0.001857	-7.31636	5.82378	6.9972	0.443368	-1.17342	-2.25546
Thoc1	NM_153552	0.005377	-5.48597	6.97616	8.15046	0.443101	-1.17429	-2.25682
B4galt4	NM_019804	0.00661	-5.17914	4.69825	5.87273	0.443042	-1.17449	-2.25712
Gas5	NR_002840	0.000739	-9.31641	8.25145	9.4274	0.442591	-1.17595	-2.25942
Trpc6	NM_013838	8.98E-05	-15.9737	4.19506	5.37216	0.442238	-1.1771	-2.26122
Crispld2	NM_030209	0.002231	-6.96715	5.18247	6.36103	0.441791	-1.17856	-2.26351
Nfix	NM_001081981	4.97E-05	-18.5495	9.06817	10.2469	0.441746	-1.17871	-2.26375
Dclk1	NM_019978	0.000443	-10.6306	7.32509	8.50485	0.441426	-1.17976	-2.26539
Rnf122	NM_175136	0.003821	-6.02602	6.64483	7.82565	0.441102	-1.18082	-2.26705
Msrb2	NM_029619	0.000264	-12.1399	4.76689	5.94955	0.440536	-1.18267	-2.26996
Glrx	NM_053108	0.000653	-9.61877	6.56207	7.74608	0.440127	-1.18401	-2.27207
Gm7265	AK087684	0.003711	-6.07455	4.81483	5.99955	0.43991	-1.18472	-2.27319
Dpep1	NM_007876	0.000282	-11.935	4.37467	5.56141	0.439295	-1.18674	-2.27638
Shisa5	NM_025858	0.00023	-12.5754	8.77956	9.96795	0.438794	-1.18838	-2.27897
Mcart1	NM_001009949	0.000114	-15.0317	6.83635	8.02912	0.43746	-1.19278	-2.28592
Mfsd7a	NM_172883	0.000203	-12.9861	5.32704	6.52064	0.43721	-1.1936	-2.28723
Ptger4	NM_001136079	0.000176	-13.4685	6.82761	8.02183	0.437023	-1.19422	-2.28821
Lepre1	NM_019783	0.000485	-10.3866	7.05295	8.24797	0.436781	-1.19502	-2.28947
Slc44a2	NM_152808	0.000493	-10.3423	8.04938	9.24597	0.436306	-1.19659	-2.29197
Bscl2	NM_008144	0.000686	-9.49617	6.16462	7.36539	0.435042	-1.20077	-2.29863

Cacna1c	NM_001159533	0.000341	-11.3755	5.80646	7.00791	0.434837	-1.20145	-2.29971
Papd4	NM_133905	0.000369	-11.1426	7.49582	8.69829	0.43453	-1.20247	-2.30134
H2-T24	NM_008207	0.0015	-7.74118	4.84752	6.0576	0.432245	-1.21008	-2.3135
Mum1l1	NM_001164630	0.001654	-7.54331	4.46563	5.67619	0.432103	-1.21055	-2.31426
Lclat1	NM_001081071	0.000606	-9.80781	8.00133	9.21347	0.431629	-1.21214	-2.31681
5730469M10Rik	BC056635	0.00199	-7.18281	6.39549	7.60875	0.431293	-1.21326	-2.31861
Zfp583	NM_001033249	0.000314	-11.6146	3.6512	4.86582	0.430887	-1.21462	-2.32079
Rarres1	NM_001164763	0.003672	-6.09191	3.65211	4.86854	0.430346	-1.21643	-2.32371
Rps19	NM_023133	0.00162	-7.58482	8.53885	9.7572	0.429775	-1.21835	-2.3268
Txnip	NM_001009935	0.007011	-5.09415	9.30952	10.528	0.429721	-1.21853	-2.32709
Setmar	NM_178391	0.005112	-5.56328	3.8834	5.10406	0.429085	-1.22066	-2.33054
Dtd1	NM_025314	0.000819	-9.07041	5.36385	6.58499	0.428944	-1.22114	-2.33131
Sec23a	NM_009147	0.000341	-11.3678	8.65496	9.87807	0.428357	-1.22311	-2.3345
Fli1	NM_008026	0.004312	-5.83039	5.0189	6.24646	0.427041	-1.22755	-2.3417
Rhou	NM_133955	0.00029	-11.8489	7.63678	8.86865	0.425764	-1.23187	-2.34872
Arhgap31	NM_020260	5.36E-05	-18.2003	6.28177	7.51921	0.424126	-1.23743	-2.35779
Nxn	NM_008750	0.002038	-7.13727	8.30873	9.55072	0.422789	-1.24199	-2.36525
Ggt7	NM_144786	0.000192	-13.1654	5.10648	6.3491	0.422606	-1.24262	-2.36627
Prickle2	NM_001134461	0.004088	-5.91622	5.87776	7.12267	0.421935	-1.24491	-2.37003
Gap43	NM_008083	0.000427	-10.7329	4.35013	5.59843	0.420942	-1.2483	-2.37562
Map3k5	NM_008580	0.001246	-8.12777	4.46942	5.71775	0.420934	-1.24833	-2.37567
Pbx1	NM_183355	0.000408	-10.8613	7.90886	9.15792	0.420723	-1.24906	-2.37686
Ptgir	NM_008967	0.002183	-7.0076	4.83815	6.08844	0.420364	-1.25029	-2.37889
Mtus1	NM_001005863	6.06E-06	-31.4862	5.37503	6.62658	0.419998	-1.25154	-2.38096
Crispld1	NM_031402	3.76E-05	-19.8969	4.36812	5.62	0.4199	-1.25188	-2.38152
3-Mar	NM_177115	0.006422	-5.22116	6.23962	7.49223	0.419691	-1.2526	-2.38271
Fam13c	NM_024244	0.000865	-8.94224	4.66932	5.92284	0.419424	-1.25352	-2.38422
Ptplad1	NM_021345	0.000846	-8.99394	7.88077	9.13493	0.419238	-1.25416	-2.38528
Crtap	NM_019922	7.81E-05	-16.5467	8.23175	9.48694	0.418937	-1.2552	-2.38699
Cd63	NM_001042580	0.005475	-5.45865	9.4529	10.7088	0.418723	-1.25593	-2.38822
Gm5458	NM_001024706	1.85E-06	-42.3855	4.29097	5.54772	0.418485	-1.25675	-2.38957

Samd5	NM_177271	0.001426	-7.84543	6.13545	7.39703	0.417087	-1.26158	-2.39758
Ick	NM_019987	2.38E-06	-39.8154	7.29072	8.55274	0.416959	-1.26202	-2.39832
Dnajc12	NM_013888	0.000251	-12.2982	4.97619	6.23931	0.416642	-1.26312	-2.40014
Slc35d2	NM_001001321	0.002611	-6.67974	3.72934	4.99612	0.415586	-1.26678	-2.40624
Basp1	NM_027395	0.000121	-14.8086	6.08894	7.35592	0.415529	-1.26698	-2.40657
Igfbp3	NM_008343	0.004681	-5.70039	6.03378	7.30357	0.414721	-1.26979	-2.41126
Rftn1	NM_181397	0.000801	-9.12399	4.90107	6.17093	0.4147	-1.26986	-2.41138
Ccng1	NM_009831	0.001894	-7.27804	9.43149	10.7015	0.414645	-1.27005	-2.4117
	---	0.00089	-8.87582	7.06651	8.33788	0.414268	-1.27136	-2.4139
Galnt12	NM_172693	0.001555	-7.66791	4.42122	5.69348	0.414011	-1.27226	-2.41539
Tacc1	NM_177089	0.00014	-14.2731	7.64247	8.91529	0.413851	-1.27282	-2.41633
Apba1	NM_177034	0.004177	-5.88142	4.30677	5.58503	0.412292	-1.27826	-2.42547
Gtdc1	NM_172662	0.004326	-5.82522	5.87909	7.16203	0.410957	-1.28294	-2.43334
Tspyl4	NM_030203	0.006876	-5.12204	4.59599	5.88076	0.410434	-1.28478	-2.43644
Stk40	NM_001145827	0.001602	-7.60813	6.20851	7.49578	0.409727	-1.28727	-2.44065
2510009E07Rik	NM_001001881	0.001213	-8.18516	5.54088	6.83004	0.409188	-1.28916	-2.44386
Ddr2	NM_022563	0.000713	-9.40168	9.14122	10.4316	0.40884	-1.29039	-2.44595
Lass4	NM_026058	0.000215	-12.7959	6.88936	8.18024	0.408703	-1.29088	-2.44677
Bnc2	NM_172870	0.00198	-7.19255	6.71859	8.01058	0.408386	-1.29199	-2.44866
Ttc38	NM_001033337	0.000394	-10.9589	4.98992	6.28202	0.408354	-1.29211	-2.44885
Zfp287	NM_133208	0.001712	-7.47592	3.9883	5.28193	0.407923	-1.29363	-2.45144
Klf15	NM_023184	0.003621	-6.11506	5.68851	6.98363	0.407501	-1.29512	-2.45398
Ifngr2	NM_008338	0.000138	-14.3252	8.41516	9.71051	0.407437	-1.29535	-2.45437
Icam1	NM_010493	0.001971	-7.20081	5.52793	6.82396	0.407247	-1.29602	-2.45551
Gria1	NM_001113325	0.001869	-7.30394	3.70858	5.00644	0.406729	-1.29786	-2.45864
Ifitm3	NM_025378	0.002186	-7.00533	10.7893	12.0881	0.406455	-1.29883	-2.4603
Trim47	NM_172570	0.001801	-7.37527	5.50655	6.80736	0.405899	-1.30081	-2.46366
Gal3st4	NM_001033416	0.006928	-5.11124	3.46895	4.77677	0.40393	-1.30782	-2.47567
Irx5	NM_018826	0.005093	-5.56915	5.82877	7.13781	0.40359	-1.30904	-2.47776
Ahnak2	AK138503	0.00059	-9.87489	8.20359	9.51307	0.403468	-1.30947	-2.47851
Klhl29	NM_001164493	0.000806	-9.10737	4.74288	6.05396	0.403016	-1.31109	-2.48129

Gabrb1	NM_008069	0.000223	-12.6714	3.47667	4.7889	0.402697	-1.31223	-2.48326
Mir485	NR_030253	0.003198	-6.32444	4.69872	6.01371	0.401927	-1.31499	-2.48801
Mn1	NM_001081235	0.00045	-10.5886	4.56427	5.89064	0.398769	-1.32638	-2.50772
Mir487b	NR_030271	0.006738	-5.15131	4.08461	5.41124	0.398699	-1.32663	-2.50816
Magee1	NM_053201	1.00E-05	-27.7735	6.27573	7.60669	0.397503	-1.33096	-2.5157
Loxl4	NM_001164311	0.002092	-7.08811	3.72696	5.05929	0.397128	-1.33233	-2.51808
Ugt1a9	NM_201644	0.000157	-13.863	6.62872	7.96195	0.396879	-1.33323	-2.51966
B4galt5	NM_019835	0.001484	-7.76291	7.06156	8.39759	0.396109	-1.33603	-2.52456
Tspan12	NM_173007	0.000175	-13.4768	3.72659	5.06314	0.395967	-1.33655	-2.52547
Rhbdf2	NM_172572	0.001541	-7.68642	4.81721	6.1539	0.395928	-1.33669	-2.52571
Aldh9a1	NM_019993	0.000254	-12.2581	7.49721	8.83771	0.394884	-1.3405	-2.53239
Cdkn1c	NM_001161624	0.005304	-5.50688	5.45451	6.7961	0.394586	-1.34159	-2.5343
Gnai1	NM_010305	0.003013	-6.42739	4.11629	5.45933	0.394188	-1.34304	-2.53686
Enah	NM_010135	1.53E-07	-79.0923	6.58137	7.92601	0.393752	-1.34464	-2.53967
1810014F10Rik	NM_026928	0.001717	-7.46979	4.64353	5.98866	0.393618	-1.34513	-2.54053
Col3a1	NM_009930	0.000215	-12.7917	11.2612	12.6094	0.39279	-1.34817	-2.54589
Mir154	NR_029564	0.000242	-12.4104	2.99182	4.34075	0.392584	-1.34893	-2.54722
Magt1	NM_001190409	0.000255	-12.2481	9.04671	10.3964	0.392384	-1.34966	-2.54853
Mir155	NR_029565	0.003402	-6.21975	4.06771	5.41942	0.391828	-1.35171	-2.55214
Kdelc2	NM_212445	0.000133	-14.4586	6.90174	8.25707	0.390846	-1.35533	-2.55855
AA388235	NR_033305	0.002841	-6.52975	3.81921	5.17533	0.390632	-1.35612	-2.55995
Hoxb9	NM_008270	0.006117	-5.2928	5.06009	6.41722	0.390357	-1.35713	-2.56176
Pde8b	NM_172263	0.00045	-10.5875	4.95682	6.3213	0.388374	-1.36448	-2.57484
Csf1	NM_007778	0.000338	-11.3956	8.10274	9.47249	0.386958	-1.36975	-2.58426
Jtb	NM_206924	0.00584	-5.3615	7.16272	8.53349	0.386685	-1.37077	-2.58609
Sdr39u1	NM_001082975	8.31E-07	-51.7993	4.8118	6.18432	0.386215	-1.37252	-2.58923
Tnfaip8	NM_134131	0.001894	-7.27827	6.88368	8.25872	0.38554	-1.37505	-2.59377
Cap2	NM_026056	0.00088	-8.90205	4.68196	6.05748	0.385416	-1.37551	-2.5946
Svil	NM_153153	1.16E-05	-26.7446	7.46644	8.84501	0.3846	-1.37857	-2.60011
Tiam1	NM_009384	0.000548	-10.0631	4.86239	6.24303	0.384051	-1.38063	-2.60382
Cdyl	NM_009881	0.000393	-10.9631	3.89675	5.28126	0.383019	-1.38451	-2.61084

Irx1	NM_010573	0.000662	-9.58654	5.15539	6.54221	0.382406	-1.38682	-2.61502
Gm1973	NM_029288	0.000344	-11.3424	3.26109	4.65275	0.381126	-1.39166	-2.62381
Hyi	NM_026601	6.99E-05	-17.0215	6.08535	7.47883	0.380645	-1.39348	-2.62712
BC034090	AK129406	0.002905	-6.49082	4.73506	6.12864	0.38062	-1.39358	-2.62729
Leprel2	NM_013534	0.000386	-11.0125	6.81129	8.20635	0.380228	-1.39506	-2.63
Mir377	NR_029878	0.000261	-12.1827	3.38904	4.78423	0.380196	-1.39519	-2.63022
Cyb5	NM_025797	0.000732	-9.33785	8.20826	9.60359	0.380157	-1.39533	-2.63049
Dse	NM_172508	0.000119	-14.888	8.58036	9.97783	0.379594	-1.39747	-2.63439
Rab20	NM_011227	0.000791	-9.15162	5.17068	6.56848	0.379505	-1.39781	-2.63501
Hoxc9	NM_008272	0.005299	-5.50841	4.8936	6.29155	0.379468	-1.39795	-2.63527
Ksr1	NM_013571	0.000824	-9.05491	5.92515	7.32378	0.379289	-1.39863	-2.63651
Gfra2	NM_008115	0.004622	-5.72004	4.59607	5.99572	0.379022	-1.39965	-2.63837
Snx30	NM_172468	0.000677	-9.52832	6.18326	7.58394	0.37875	-1.40068	-2.64026
Cebpd	NM_007679	0.002127	-7.05673	4.6353	6.03734	0.378394	-1.40204	-2.64275
Fndc3a	NM_207636	0.000406	-10.8745	9.06954	10.4716	0.378383	-1.40208	-2.64282
Idua	NM_008325	0.001743	-7.43953	4.92505	6.32861	0.377996	-1.40356	-2.64553
Cnp	NM_009923	0.000892	-8.87107	4.42442	5.82907	0.37771	-1.40465	-2.64753
	---	0.004985	-5.60209	7.64321	9.04903	0.377402	-1.40583	-2.6497
Nenf	NM_025424	0.001725	-7.46029	7.15421	8.56053	0.377273	-1.40632	-2.6506
Psmb8	NM_010724	0.001763	-7.41788	4.02015	5.42798	0.376878	-1.40783	-2.65338
Gm3579	AY140896	0.000459	-10.5376	3.96776	5.37677	0.376572	-1.409	-2.65554
Gm3579	AY140896	0.000459	-10.5376	3.96776	5.37677	0.376572	-1.409	-2.65554
Cyp4v3	NM_133969	5.75E-05	-17.8815	5.27869	6.68907	0.376212	-1.41038	-2.65808
Notch1	NM_008714	0.002717	-6.60896	5.46505	6.87618	0.376017	-1.41113	-2.65945
Cmtm6	NM_026036	8.77E-05	-16.0696	7.23354	8.64548	0.375805	-1.41194	-2.66095
Snai2	NM_011415	0.001611	-7.59674	7.32384	8.737	0.375488	-1.41316	-2.6632
Stxbp6	NM_144552	0.001218	-8.17664	4.65436	6.06853	0.375224	-1.41418	-2.66508
Slc43a1	NM_001081349	0.000401	-10.9054	4.25921	5.67359	0.375171	-1.41438	-2.66545
Tcf4	NM_013685	1.52E-05	-25.0182	8.45273	9.86767	0.375027	-1.41493	-2.66648
Arl5a	NM_182994	0.001368	-7.93191	8.16903	9.58425	0.374953	-1.41522	-2.667
Mir369	NR_030272	0.00079	-9.15507	2.98279	4.39845	0.374839	-1.41566	-2.66781

Ibtk	NM_001081282	0.000624	-9.73247	7.0783	8.49551	0.374433	-1.41722	-2.6707
Gm5458	NM_001024706	0.00016	-13.7856	3.75579	5.17347	0.374315	-1.41768	-2.67155
Fam198a	BC113767	4.35E-05	-19.1878	4.60434	6.03018	0.372204	-1.42584	-2.6867
Tmem223	NM_025791	0.000218	-12.7516	7.90365	9.33029	0.371996	-1.42664	-2.6882
Ntn1	NM_008744	0.000727	-9.35568	5.44953	6.87642	0.371932	-1.42689	-2.68866
Slc2a3	NM_011401	0.002962	-6.45688	4.35938	5.78642	0.371893	-1.42704	-2.68895
Paox	NM_153783	0.001941	-7.23037	5.56153	6.98963	0.371619	-1.4281	-2.69093
D10Ertd610e	NM_028027	2.03E-05	-23.2499	7.81542	9.24451	0.371366	-1.42909	-2.69276
Abcb8	NM_029020	0.000234	-12.5269	5.48792	6.91846	0.370993	-1.43054	-2.69547
Ak5	NM_001081277	0.00449	-5.76578	4.61506	6.04581	0.370938	-1.43075	-2.69587
Maob	NM_172778	9.08E-05	-15.9284	4.73892	6.17094	0.370613	-1.43202	-2.69824
Nbl1	NM_008675	0.000253	-12.2695	7.12237	8.55608	0.370176	-1.43372	-2.70142
2900062L11Rik	NM_029823	0.002405	-6.82893	2.84505	4.27998	0.369866	-1.43493	-2.70368
St6gal1	NM_145933	1.54E-05	-24.919	4.58569	6.02678	0.368289	-1.44109	-2.71526
Tmeff1	NM_021436	0.002575	-6.70473	6.51106	7.95233	0.368243	-1.44127	-2.7156
Leprel1	NM_173379	0.000678	-9.52583	4.1594	5.60487	0.367173	-1.44547	-2.72351
Rab39b	NM_175122	0.005206	-5.53542	4.46955	5.91635	0.366835	-1.4468	-2.72602
H60b	NM_001177775	0.000922	-8.79548	2.81425	4.26475	0.365896	-1.45049	-2.73302
Nmi	NM_001141949	0.000775	-9.2007	3.58886	5.04302	0.364966	-1.45417	-2.73998
Cblb	NM_001033238	5.40E-05	-18.1665	6.75789	8.21242	0.364873	-1.45453	-2.74068
Entpd5	NM_001026214	1.12E-05	-26.9953	6.68748	8.14428	0.364299	-1.4568	-2.745
Hoxb2	NM_134032	0.000163	-13.7208	5.36966	6.82827	0.363844	-1.45861	-2.74843
Fndc4	NM_022424	0.002426	-6.81277	4.59224	6.05322	0.363248	-1.46097	-2.75294
Ckb	NM_021273	0.000997	-8.61703	6.27555	7.74053	0.36224	-1.46498	-2.7606
Ctsb	NM_007798	0.000526	-10.1732	10.5546	12.0268	0.360415	-1.47227	-2.77458
Hoxd4	NM_010469	0.005076	-5.57429	4.03404	5.51162	0.359089	-1.47758	-2.78482
Ssh3	NM_198113	0.000137	-14.3451	5.6055	7.08397	0.358868	-1.47847	-2.78654
	---	0.000243	-12.3992	8.47271	9.95147	0.358797	-1.47876	-2.78709
Hs3st1	NM_010474	0.000285	-11.9072	4.22781	5.70762	0.358536	-1.47981	-2.78912
Ppl	NM_008909	0.000114	-15.0448	4.89219	6.37642	0.357439	-1.48423	-2.79768
Nsg1	NM_010942	0.000451	-10.5863	8.20536	9.68962	0.357431	-1.48426	-2.79774

Itga9	NM_133721	0.001434	-7.83368	4.6751	6.1602	0.357224	-1.4851	-2.79936
Slc19a2	NM_054087	0.000412	-10.8301	6.77232	8.25864	0.356923	-1.48631	-2.80172
Pard6g	NM_053117	0.000627	-9.72107	5.15364	6.6405	0.356789	-1.48686	-2.80278
Fah	NM_010176	0.000539	-10.1092	4.47589	5.96785	0.355529	-1.49196	-2.81271
Btg2	NM_007570	0.001257	-8.10906	5.58009	7.07291	0.355317	-1.49282	-2.81439
A4galt	NM_001170954	0.004132	-5.89862	6.04935	7.5428	0.355162	-1.49345	-2.81561
Nfil3	NM_017373	0.00056	-10.0102	7.20482	8.69934	0.354897	-1.49453	-2.81772
Ahnak2	BC138468	0.000264	-12.1456	8.0481	9.54727	0.353757	-1.49917	-2.8268
Lhfpl2	NM_172589	8.99E-05	-15.9708	8.83234	10.3353	0.352827	-1.50297	-2.83425
Esr1	NM_007956	0.005539	-5.44104	4.72984	6.23545	0.352181	-1.50561	-2.83945
	---	0.000107	-15.2665	3.94425	5.4502	0.352099	-1.50595	-2.84011
Naprt1	NM_172607	0.002924	-6.4793	5.37215	6.87817	0.35208	-1.50603	-2.84027
Tpst1	NM_001130476	6.61E-05	-17.2642	7.38087	8.88717	0.352012	-1.5063	-2.84081
Slc6a8	NM_133987	0.000493	-10.3457	6.62436	8.13696	0.35048	-1.5126	-2.85323
Zfp7	NM_145916	0.000241	-12.4303	3.64788	5.16171	0.350181	-1.51383	-2.85567
Tnik	NM_026910	0.000407	-10.8649	4.91383	6.428	0.350099	-1.51417	-2.85634
Gm13691	NM_001177432	0.000291	-11.8434	8.40674	9.92132	0.35	-1.51457	-2.85714
Gm13691	NM_001177432	0.000291	-11.8434	8.40674	9.92132	0.35	-1.51457	-2.85714
Gm13691	NM_001177432	0.000291	-11.8434	8.40674	9.92132	0.35	-1.51457	-2.85714
Gm13691	NM_001177432	0.000291	-11.8434	8.40674	9.92132	0.35	-1.51457	-2.85714
Gm13691	NM_001177432	0.000291	-11.8434	8.40674	9.92132	0.35	-1.51457	-2.85714
Gm13691	NM_001177432	0.000291	-11.8434	8.40674	9.92132	0.35	-1.51457	-2.85714
Gm13691	NM_001177432	0.000291	-11.8434	8.40674	9.92132	0.35	-1.51457	-2.85714
ND6	ENSMUST00000082419	0.006399	-5.22649	9.14835	10.6661	0.349232	-1.51774	-2.86342
Errfi1	NM_133753	0.002022	-7.15193	7.89852	9.41922	0.348518	-1.52069	-2.86929
Lxn	NM_016753	0.002782	-6.56693	7.31474	8.8422	0.346888	-1.52746	-2.88277
Hic1	NM_010430	0.003484	-6.17959	5.56518	7.09372	0.346629	-1.52854	-2.88493
Gng8	NM_010320	0.000987	-8.63886	4.81196	6.34399	0.345792	-1.53202	-2.89191
Ralgds	NM_001145835	0.001238	-8.14274	6.23476	7.76984	0.345062	-1.53507	-2.89803
Scara3	NM_172604	0.000507	-10.2683	4.46819	6.00341	0.345027	-1.53522	-2.89833

Gclc	NM_010295	0.000415	-10.81	7.23366	8.77002	0.344755	-1.53636	-2.90061
Dzip1	NM_025943	0.000539	-10.1101	6.2959	7.83462	0.344189	-1.53873	-2.90538
Ttyh3	NM_175274	2.98E-05	-21.1111	7.03804	8.57825	0.343836	-1.54021	-2.90836
Moxd1	NM_021509	0.00301	-6.42889	4.07155	5.61247	0.343668	-1.54091	-2.90979
Fosl2	NM_008037	0.000121	-14.8056	7.32238	8.87203	0.341593	-1.54965	-2.92746
Aoc3	NM_009675	0.000411	-10.8423	5.72243	7.2797	0.339795	-1.55727	-2.94295
Dnajc15	NM_025384	0.002398	-6.8344	5.80816	7.37092	0.338502	-1.56276	-2.95419
Sgpp1	NM_030750	4.75E-05	-18.7613	7.32997	8.89739	0.337412	-1.56742	-2.96374
Pip4k2a	NM_008845	0.000344	-11.3498	6.64246	8.21113	0.33712	-1.56866	-2.9663
Ctsz	NM_022325	0.000464	-10.5041	9.80226	11.3726	0.336725	-1.57036	-2.96979
Lhfp	NM_175386	9.33E-05	-15.8214	7.41675	8.98814	0.336483	-1.57139	-2.97192
Idh1	NM_010497	0.001088	-8.42163	9.27469	10.853	0.334868	-1.57834	-2.98625
Mageh1	NM_023788	0.003189	-6.32931	3.9852	5.57426	0.332387	-1.58907	-3.00854
Bcor	NM_029510	0.000211	-12.858	4.61471	6.20412	0.332307	-1.58941	-3.00926
Angptl2	NM_011923	0.002741	-6.59318	8.73638	10.336	0.329963	-1.59962	-3.03064
Eid2b	NM_001177427	0.000723	-9.36855	4.53519	6.13661	0.329552	-1.60142	-3.03443
Rnf144a	NM_001081977	2.58E-05	-21.878	5.298	6.9023	0.328895	-1.6043	-3.04048
Rnf113a2	NM_025525	0.004034	-5.93749	4.49915	6.10346	0.328894	-1.6043	-3.04049
Kctd12b	NM_175429	0.002721	-6.60624	4.22806	5.83863	0.327469	-1.61057	-3.05372
Klhl23	NM_177784	0.000614	-9.7746	3.77723	5.38974	0.327029	-1.61251	-3.05783
Csrp2	NM_007792	0.004503	-5.76133	7.16193	8.7769	0.326472	-1.61497	-3.06305
Ryk	NM_013649	3.09E-05	-20.9103	8.17329	9.78892	0.326323	-1.61563	-3.06445
Lbh	NM_029999	0.000152	-13.979	7.75173	9.3699	0.325747	-1.61818	-3.06987
Grasp	NM_019518	0.005966	-5.32972	6.43908	8.05743	0.325707	-1.61835	-3.07024
Mir543	NR_030261	0.000191	-13.1874	3.31604	4.9355	0.325458	-1.61946	-3.07259
	---	0.003003	-6.43337	4.05958	5.67945	0.325366	-1.61987	-3.07347
Rcn3	NM_026555	0.000238	-12.4674	9.69373	11.3145	0.325166	-1.62075	-3.07536
Cfb	NM_008198	0.002346	-6.87442	5.25531	6.8771	0.324932	-1.62179	-3.07757
Camk1d	NM_177343	0.000763	-9.23896	4.3107	5.93305	0.324806	-1.62235	-3.07876
Stox2	NM_175162	0.002891	-6.49915	3.949	5.57182	0.324701	-1.62282	-3.07976
Tshz1	NM_001081300	0.000319	-11.5655	6.27968	7.90371	0.324428	-1.62403	-3.08235

	---	0.003459	-6.1916	9.16736	10.7934	0.323987	-1.62599	-3.08655
Zfp105	NM_009544	0.000723	-9.36884	3.66613	5.29324	0.323735	-1.62712	-3.08895
Ece2	NM_139293	0.000482	-10.402	4.74622	6.37682	0.322954	-1.6306	-3.09642
Gm5458	NM_001024706	0.000798	-9.13239	3.73355	5.36818	0.322054	-1.63463	-3.10507
Trerf1	NM_001097623	0.000177	-13.4445	5.12625	6.76266	0.321656	-1.63641	-3.10891
Flrt2	NM_201518	0.003825	-6.0243	7.06701	8.70477	0.321355	-1.63776	-3.11182
Pcdhb16	NM_053141	0.001412	-7.86613	6.6038	8.24168	0.321328	-1.63788	-3.11209
Pir	NM_027153	4.61E-05	-18.9015	4.46561	6.10907	0.320088	-1.64346	-3.12415
Mapkapk3	NM_178907	0.001736	-7.44764	4.53986	6.18512	0.319688	-1.64527	-3.12805
Tmem154	NM_177260	0.000658	-9.60114	3.73016	5.38093	0.318471	-1.65077	-3.14001
Col4a5	NM_001163155	0.000232	-12.5462	5.13367	6.78997	0.317253	-1.6563	-3.15206
Rassf8	ENSMUST0000011170 4	0.000305	-11.7015	6.47059	8.127	0.317229	-1.6564	-3.15229
Gm10808	ENSMUST0000009972 8	0.001272	-8.0841	4.02088	5.68051	0.316519	-1.65964	-3.15937
B4galnt1	NM_008080	0.001008	-8.59314	5.10266	6.76378	0.316194	-1.66112	-3.16261
Six1	NM_009189	0.003729	-6.06649	3.68455	5.34736	0.315824	-1.66281	-3.16632
Slc36a4	NM_172289	0.000299	-11.7654	7.02258	8.68548	0.315804	-1.6629	-3.16652
Greb1l	NM_001083628	0.000469	-10.4803	4.79541	6.46255	0.314876	-1.66714	-3.17585
Dennd2c	NM_177857	0.006307	-5.24773	4.35935	6.02884	0.314364	-1.66949	-3.18103
Ano3	NM_001128103	0.005056	-5.58027	3.58902	5.25978	0.314088	-1.67076	-3.18382
Cebpa	NM_007678	0.001116	-8.36651	4.78772	6.46061	0.313625	-1.67289	-3.18852
Hebp1	NM_013546	0.000418	-10.7904	4.7398	6.41456	0.313219	-1.67476	-3.19266
Rnf24	NM_178607	0.003017	-6.4249	5.72052	7.3986	0.312498	-1.67808	-3.20002
	---	0.001324	-7.99948	4.0363	5.71739	0.311846	-1.68109	-3.20671
Mir679	NR_030445	0.003478	-6.18237	5.28765	6.9702	0.311532	-1.68255	-3.20994
	---	6.81E-05	-17.1302	4.53339	6.21988	0.310681	-1.68649	-3.21873
Capns2	NM_027112	0.000828	-9.04447	4.34827	6.03611	0.310393	-1.68783	-3.22172
Layn	NM_001033534	0.004641	-5.71378	7.34501	9.03297	0.310366	-1.68796	-3.22201
Fam78b	NM_001160262	4.02E-05	-19.5665	3.70625	5.40119	0.308867	-1.69494	-3.23764
Ica1	NM_010492	0.000199	-13.0505	3.56962	5.26761	0.308215	-1.69799	-3.24449
Pfkfb3	NM_001177753	0.003848	-6.01476	6.1756	7.87474	0.307971	-1.69914	-3.24706

BC005764	NR_033210	0.004053	-5.93012	5.50617	7.20803	0.307389	-1.70186	-3.2532
Inpp1	NM_008384	2.16E-05	-22.888	4.89573	6.59885	0.30712	-1.70313	-3.25606
	---	0.000376	-11.0914	5.51847	7.2225	0.306927	-1.70403	-3.2581
Pld2	NM_008876	4.88E-05	-18.6319	4.88164	6.58788	0.306457	-1.70624	-3.2631
Rasl11a	NM_026864	0.007097	-5.07676	5.5126	7.22107	0.305985	-1.70847	-3.26813
Mgll	NM_001166251	0.001661	-7.53599	5.13674	6.84573	0.305876	-1.70898	-3.2693
Chsy3	NM_001081328	0.004061	-5.92687	4.78338	6.49302	0.305736	-1.70964	-3.27079
Smarca1	NM_053123	0.002035	-7.14049	3.83539	5.54681	0.305358	-1.71142	-3.27484
Fkbp7	NM_010222	0.002234	-6.96455	7.54912	9.26523	0.304369	-1.71611	-3.28549
Cxcl16	NM_023158	0.000451	-10.5842	4.04488	5.76229	0.304094	-1.71741	-3.28845
Zfhx4	NM_030708	0.002997	-6.4365	5.74781	7.46654	0.303817	-1.71873	-3.29145
Enox1	NM_172813	0.001237	-8.14361	5.06599	6.78575	0.303599	-1.71976	-3.29382
Mmp23	NM_011985	0.001097	-8.40394	4.5132	6.23487	0.303197	-1.72167	-3.29818
Gm3002	NR_033388	0.002688	-6.628	3.91092	5.63336	0.303036	-1.72244	-3.29994
Phactr2	NM_001195096	0.000876	-8.91339	3.85237	5.57569	0.302851	-1.72332	-3.30195
Pcdhb14	NM_053139	0.001971	-7.20155	3.64545	5.36918	0.302764	-1.72373	-3.3029
Mmp27	NM_001030289	0.000147	-14.0878	3.89549	5.62336	0.301897	-1.72787	-3.31238
Fxyd1	NM_052992	0.000105	-15.3643	7.19884	8.92742	0.301747	-1.72859	-3.31403
Armcx1	NM_001166377	1.11E-05	-27.0635	5.90011	7.62948	0.301582	-1.72938	-3.31585
Gch1	NM_008102	6.65E-05	-17.2318	3.57442	5.30523	0.301283	-1.73081	-3.31914
Antxr1	NM_054041	1.38E-05	-25.6043	8.52643	10.2601	0.300694	-1.73363	-3.32564
Tmed3	NM_025360	0.000218	-12.7561	7.51219	9.24684	0.300482	-1.73465	-3.32799
Nr4a2	NM_001139509	0.005782	-5.37641	6.46643	8.20313	0.300056	-1.73669	-3.33271
2010002N04Rik	NM_134133	0.006537	-5.19541	6.705	8.44213	0.299966	-1.73713	-3.33371
Rad9b	NM_144912	0.005186	-5.54126	4.65206	6.39186	0.299412	-1.7398	-3.33988
Abcd2	NM_011994	0.000159	-13.8239	4.97842	6.71918	0.299211	-1.74077	-3.34212
Lhx8	NM_010713	0.004697	-5.69482	4.23141	5.97228	0.299189	-1.74087	-3.34236
Kif26b	NM_001161665	0.000733	-9.33629	5.68058	7.42251	0.298969	-1.74193	-3.34483
Mir134	NR_029548	0.000647	-9.64127	5.83584	7.58174	0.298149	-1.74589	-3.35403
Slc2a13	NM_001033633	9.32E-05	-15.8252	5.5014	7.24731	0.298146	-1.74591	-3.35406
Cercam	NM_207298	0.003407	-6.21697	5.2364	6.98308	0.297988	-1.74667	-3.35584

Kif26b	NM_001161665	0.000365	-11.1721	5.77469	7.5237	0.297507	-1.749	-3.36126
Nme4	NM_019731	0.000397	-10.9361	4.6299	6.38826	0.295582	-1.75837	-3.38315
Nudt12	NM_026497	0.000117	-14.9298	4.101	5.86486	0.29446	-1.76386	-3.39605
BC026585	NM_001033284	0.005882	-5.35098	4.59185	6.35715	0.294166	-1.7653	-3.39944
Tmem119	NM_146162	0.002179	-7.01081	6.42523	8.19265	0.293734	-1.76742	-3.40444
Mir380	NR_029881	2.17E-05	-22.8572	2.90388	4.67895	0.292181	-1.77506	-3.42253
Rhoj	NM_023275	3.32E-05	-20.5346	7.53152	9.31207	0.291072	-1.78055	-3.43557
Gstt1	NM_008185	2.34E-05	-22.4339	4.93104	6.71456	0.290474	-1.78352	-3.44265
Tbx3	NM_011535	0.000571	-9.96042	7.01745	8.80387	0.289891	-1.78642	-3.44958
Gm1973	NM_029288	0.006267	-5.25712	3.7827	5.57172	0.289369	-1.78902	-3.4558
Sirpa	NM_007547	1.10E-05	-27.0877	7.01014	8.80175	0.288849	-1.79161	-3.46202
Fam13a	NM_153574	0.000717	-9.38736	3.63344	5.42753	0.288352	-1.7941	-3.46798
Crip1	NM_007763	0.001355	-7.951	5.98958	7.78591	0.287907	-1.79632	-3.47334
Gm9927	AK034060	9.98E-05	-15.5514	2.84787	4.64569	0.287608	-1.79782	-3.47696
G630090E17Rik	NM_001173500	0.001495	-7.74832	3.05152	4.85415	0.28665	-1.80264	-3.48857
4930523C07Rik	NM_001162896	0.000348	-11.313	5.73578	7.54224	0.285893	-1.80646	-3.49782
Ctsh	NM_007801	0.00375	-6.05692	3.85341	5.66132	0.285605	-1.80791	-3.50134
	---	0.000127	-14.6375	4.36662	6.17913	0.284695	-1.81251	-3.51253
Tgfbr2	NM_009371	0.003979	-5.95991	7.88615	9.70099	0.284236	-1.81484	-3.5182
Gas7	NM_008088	0.002808	-6.55084	6.85846	8.67405	0.284087	-1.81559	-3.52005
Cyp2j9	NM_028979	0.000614	-9.77387	4.0402	5.85818	0.283618	-1.81798	-3.52586
Mgmt	NM_008598	0.000702	-9.44216	4.94614	6.76598	0.283253	-1.81984	-3.53042
Plce1	NM_019588	0.003237	-6.30412	4.02032	5.84142	0.283005	-1.8211	-3.53351
Hhipl1	NM_001044380	3.34E-05	-20.5045	5.35592	7.17858	0.2827	-1.82266	-3.53732
Xlr3a	NM_001110784	0.000573	-9.94802	3.70548	5.53164	0.282013	-1.82617	-3.54594
Arhgef5	NM_133674	1.21E-05	-26.4786	4.55137	6.3779	0.281943	-1.82653	-3.54682
Hoxb7	NM_010460	0.001178	-8.24994	5.30815	7.13473	0.281932	-1.82658	-3.54696
Xylt1	NM_175645	0.000956	-8.71309	4.92676	6.75569	0.281473	-1.82893	-3.55274
4930555G01Rik	NM_175393	0.00163	-7.57357	3.8143	5.64457	0.281212	-1.83027	-3.55604
4930555G01Rik	NM_175393	0.00163	-7.57357	3.8143	5.64457	0.281212	-1.83027	-3.55604
Tet2	NM_001040400	4.31E-05	-19.2253	4.17581	6.00816	0.280806	-1.83235	-3.56118

Fam43a	NM_177632	9.07E-06	-28.4606	5.36229	7.1949	0.280757	-1.83261	-3.5618
Slc8a1	NM_011406	0.000734	-9.33262	3.81742	5.65242	0.280291	-1.835	-3.56772
Flt1	NM_010228	0.000401	-10.91	5.0449	6.88402	0.279493	-1.83912	-3.57791
Letmd1	NM_134093	5.07E-05	-18.4566	5.1222	6.96167	0.279423	-1.83947	-3.5788
Zfp667	NM_001024928	0.000106	-15.2986	4.80266	6.65001	0.277903	-1.84735	-3.59838
Sparcl1	NM_010097	0.001462	-7.79326	4.34185	6.1908	0.277593	-1.84896	-3.6024
Plcb1	NM_019677	8.32E-05	-16.2862	5.57941	7.42863	0.277542	-1.84922	-3.60305
Xdh	NM_011723	0.001023	-8.55887	8.41368	10.2684	0.276482	-1.85474	-3.61687
Adcy2	NM_153534	0.001448	-7.81286	4.0396	5.89886	0.275617	-1.85926	-3.62822
Acsf2	NM_153807	0.000125	-14.6958	4.10865	5.96842	0.275521	-1.85977	-3.62949
Gli2	NM_001081125	0.002084	-7.09495	5.24277	7.10538	0.274979	-1.86261	-3.63665
Nol3	NM_030152	0.000198	-13.071	4.97445	6.83718	0.274955	-1.86273	-3.63696
Fzd6	NM_008056	0.003103	-6.37638	4.59774	6.463	0.274475	-1.86526	-3.64333
Fgd4	NM_139232	0.005448	-5.46617	3.46743	5.33579	0.273886	-1.86835	-3.65115
Acsf2	NM_153807	9.62E-06	-28.0446	4.33679	6.2118	0.272625	-1.87501	-3.66804
Enc1	NM_007930	0.000742	-9.306	7.40195	9.27879	0.27228	-1.87684	-3.67269
Trf	NM_133977	0.002969	-6.45318	4.86786	6.74695	0.271855	-1.87909	-3.67844
Scarf2	NM_153790	0.001305	-8.02951	5.65067	7.53046	0.271724	-1.87979	-3.68021
Bambi	NM_026505	0.000137	-14.3594	5.40217	7.28196	0.271724	-1.87979	-3.68021
Egr1	NM_007913	0.000176	-13.4652	5.37403	7.25918	0.270715	-1.88515	-3.69392
Gnptab	NM_001004164	8.36E-05	-16.2644	7.2329	9.13399	0.267742	-1.90109	-3.73494
Fat4	NM_183221	0.000815	-9.08178	5.54853	7.44965	0.267736	-1.90111	-3.73502
Arhgap6	NM_009707	9.42E-05	-15.7824	3.9184	5.82696	0.266359	-1.90856	-3.75434
Gpx7	NM_024198	0.000614	-9.77227	4.5193	6.42799	0.266334	-1.90869	-3.75468
Zfp518a	NM_028319	0.001096	-8.40637	3.22654	5.13909	0.265622	-1.91256	-3.76475
Fzd1	NM_021457	9.28E-05	-15.8416	8.30323	10.2166	0.26547	-1.91338	-3.7669
Ddit4l	NM_030143	0.000688	-9.48863	4.74321	6.66373	0.264158	-1.92053	-3.78561
Pde4b	NM_019840	0.005664	-5.40738	4.66619	6.59206	0.263182	-1.92587	-3.79965
Mir329	NR_029762	7.48E-05	-16.7287	3.98388	5.91274	0.262637	-1.92886	-3.80754
Tuba4a	NM_009447	0.000192	-13.1613	5.18628	7.11827	0.262068	-1.93198	-3.8158
Thbs2	NM_011581	0.001299	-8.03953	9.8544	11.7867	0.262005	-1.93233	-3.81671

Ghr	NM_010284	0.000114	-15.0513	9.1836	11.119	0.261454	-1.93537	-3.82476
Rbms3	NM_001172123	2.34E-05	-22.4398	7.05591	8.99139	0.261434	-1.93548	-3.82505
Fgfr2	NM_010207	0.003008	-6.43011	5.79403	7.73222	0.260943	-1.93819	-3.83225
Bace2	NM_019517	0.001018	-8.56989	4.29173	6.23787	0.259509	-1.94614	-3.85342
	---	0.000383	-11.0377	7.90174	9.85058	0.259025	-1.94884	-3.86063
Zrsr1	NM_011663	8.17E-05	-16.3601	4.03886	5.98931	0.258736	-1.95045	-3.86494
Pygl	NM_133198	0.002902	-6.49287	4.47397	6.42947	0.25783	-1.95551	-3.87852
Pion	NM_175437	0.000693	-9.47396	3.68791	5.64346	0.257823	-1.95555	-3.87864
H2-T23	NM_010398	0.000353	-11.2706	6.20271	8.16515	0.256595	-1.96243	-3.89719
Lrig3	NM_177152	0.000748	-9.28743	6.63003	8.59679	0.255827	-1.96676	-3.90889
Auts2	NM_177047	0.000144	-14.1625	4.34953	6.31799	0.255526	-1.96846	-3.9135
Ptk7	NM_175168	0.001423	-7.84955	5.32477	7.29476	0.255255	-1.96999	-3.91765
Fndc1	NM_001081416	0.000656	-9.60737	4.25821	6.23589	0.253899	-1.97768	-3.93858
Gstt3	NM_133994	0.000332	-11.4517	5.27145	7.25193	0.253405	-1.98048	-3.94626
Parm1	NM_145562	0.000267	-12.1122	5.04413	7.0283	0.252759	-1.98417	-3.95634
Mef2c	NM_001170537	0.001214	-8.18471	4.01849	6.00371	0.252574	-1.98522	-3.95923
Lpar1	NM_010336	8.87E-05	-16.0215	7.44658	9.43587	0.251863	-1.98929	-3.97041
Enpp5	NM_032003	0.000322	-11.5372	3.97507	5.96446	0.251846	-1.98939	-3.97069
Armcx6	NM_001007578	0.001328	-7.99396	4.79508	6.78496	0.25176	-1.98988	-3.97204
Map3k8	NM_007746	0.000144	-14.1695	5.43194	7.42764	0.250747	-1.99569	-3.98808
Kera	NM_008438	0.006252	-5.26062	3.86966	5.87176	0.249636	-2.0021	-4.00583
Sema7a	NM_011352	6.33E-06	-31.1541	5.29587	7.29933	0.249401	-2.00346	-4.00961
2610524H06Rik	NM_181075	0.000952	-8.72167	4.57214	6.57572	0.249379	-2.00359	-4.00996
Usp11	NM_145628	0.000116	-14.9647	4.53496	6.53913	0.249278	-2.00417	-4.01159
Pdgfrb	NM_001146268	0.000456	-10.5563	8.88502	10.8898	0.24918	-2.00474	-4.01316
Gas5	NR_002840	5.42E-05	-18.1512	9.658	11.6638	0.248992	-2.00583	-4.0162
Pde3a	NM_018779	0.003315	-6.26337	4.08075	6.08868	0.248629	-2.00793	-4.02205
Ccl7	NM_013654	0.002364	-6.86033	7.80147	9.81067	0.248411	-2.0092	-4.02559
	---	0.000481	-10.4078	4.67667	6.68617	0.248358	-2.00951	-4.02644
Atp1b1	NM_009721	0.004036	-5.93681	4.32604	6.33611	0.248261	-2.01007	-4.02802
Mir323	NR_029757	4.87E-05	-18.6425	4.08443	6.09936	0.247426	-2.01493	-4.04161

Bcl3	NM_033601	0.000341	-11.368	5.3048	7.32077	0.247247	-2.01597	-4.04454
Trim13	NM_001164220	2.85E-05	-21.351	3.04904	5.06585	0.247103	-2.01681	-4.04689
Greb1l	NM_001083628	8.40E-05	-16.2484	4.11886	6.14093	0.246205	-2.02207	-4.06166
Khdrbs3	NM_010158	0.002222	-6.97449	4.82104	6.84694	0.245552	-2.0259	-4.07245
Xlr3c	NM_011727	0.000277	-11.9918	3.48934	5.52481	0.243929	-2.03547	-4.09956
Lsp1	NM_019391	0.000154	-13.9196	7.57458	9.61022	0.243901	-2.03563	-4.10003
	---	0.002744	-6.59124	5.98022	8.01636	0.243815	-2.03614	-4.10148
C3	NM_009778	0.000184	-13.3177	10.2986	12.338	0.243275	-2.03934	-4.11058
Pnp2	NM_001123371	1.15E-05	-26.8107	9.15488	11.1958	0.243014	-2.04089	-4.11499
Sumo3	NM_019929	0.000185	-13.3041	6.67898	8.72142	0.242752	-2.04245	-4.11943
Plagl1	NM_009538	0.004488	-5.76649	4.442	6.48939	0.241922	-2.04739	-4.13357
Tbx15	NM_009323	2.66E-05	-21.7167	7.30299	9.35524	0.241108	-2.05225	-4.14752
Fmr1	NM_008031	1.59E-05	-24.7254	8.0256	10.0803	0.240706	-2.05466	-4.15445
Slc19a1	NM_031196	0.001187	-8.23265	5.39146	7.44762	0.240456	-2.05615	-4.15876
Aldh7a1	NM_001127338	1.39E-06	-45.5126	6.75594	8.81255	0.240381	-2.05661	-4.16006
Nox4	NM_015760	0.000138	-14.3251	3.91718	5.97691	0.239862	-2.05972	-4.16906
Gm3002	NR_033388	0.000829	-9.04025	4.14917	6.2306	0.23628	-2.08143	-4.23227
Pdk4	NM_013743	0.000557	-10.0243	4.07573	6.16318	0.235296	-2.08745	-4.24996
Fbxl7	NM_176959	0.001904	-7.26747	4.8397	6.92903	0.23499	-2.08933	-4.2555
Larp6	NM_026235	0.001046	-8.51053	4.9455	7.03549	0.234882	-2.08999	-4.25745
B3galtl	NM_001081204	0.000272	-12.0524	4.29635	6.38859	0.234516	-2.09224	-4.2641
Capn6	NM_007603	0.000292	-11.838	5.90621	8.00003	0.234259	-2.09382	-4.26877
Airn	NR_002853	0.000169	-13.6069	3.68664	5.78217	0.233982	-2.09553	-4.27383
Fkbp11	NM_024169	0.000581	-9.91489	5.01065	7.11138	0.23314	-2.10073	-4.28927
Fam84a	NM_029007	0.004385	-5.80366	4.37551	6.48063	0.232431	-2.10513	-4.30235
Irx2	NM_010574	1.75E-05	-24.1148	6.00416	8.11333	0.231781	-2.10917	-4.31442
Rhod	NM_007485	5.41E-07	-57.6867	5.26281	7.37433	0.231402	-2.11153	-4.32148
Dhx40	NM_026191	0.000197	-13.0902	5.6148	7.72751	0.231212	-2.11271	-4.32504
Tnxb	NM_031176	2.52E-05	-22.0122	5.28839	7.40418	0.23072	-2.11578	-4.33425
Arhgef5	NM_133674	3.92E-05	-19.6965	4.71048	6.82784	0.230468	-2.11736	-4.339
Jam3	NM_023277	0.000354	-11.2623	5.93662	8.06212	0.229171	-2.1255	-4.36355

Gm10406	NM_001164727	0.00199	-7.18273	4.17421	6.30143	0.228898	-2.12722	-4.36875
Gm2897	NM_001177714	0.00112	-8.35968	3.46955	5.59752	0.228779	-2.12797	-4.37102
Gm2897	NM_001177714	0.00112	-8.35968	3.46955	5.59752	0.228779	-2.12797	-4.37102
Osr2	NM_054049	0.000576	-9.93672	5.15343	7.28222	0.22865	-2.12879	-4.37351
Gm3002	NR_033388	0.000814	-9.08413	3.88863	6.01841	0.228493	-2.12978	-4.3765
Peli2	NM_033602	7.56E-05	-16.6845	5.28359	7.4146	0.228297	-2.13101	-4.38025
Adcy3	NM_001159537	0.000258	-12.2139	4.89265	7.026	0.227927	-2.13335	-4.38737
Pnp	NM_013632	5.47E-05	-18.1056	8.03239	10.1662	0.22785	-2.13384	-4.38885
C2	NM_013484	0.001216	-8.18013	4.28921	6.42847	0.226996	-2.13926	-4.40537
2210016F16Rik	NM_027335	2.54E-05	-21.9688	5.49765	7.65015	0.224922	-2.1525	-4.44598
Itgbl1	NM_145467	0.000263	-12.157	4.70219	6.85644	0.22465	-2.15425	-4.45136
Pcdhb19	NM_053144	0.001174	-8.25699	4.0453	6.19986	0.224601	-2.15456	-4.45234
Psrc1	NM_019976	0.003436	-6.20261	5.16408	7.32074	0.224276	-2.15665	-4.45879
Ggta1	NM_010283	0.000218	-12.7557	5.61159	7.76853	0.224231	-2.15694	-4.45969
Gm3002	NR_033388	0.000761	-9.24497	3.62484	5.78248	0.224122	-2.15764	-4.46185
Gpr176	NM_201367	0.004916	-5.62379	4.69999	6.86678	0.222705	-2.16679	-4.49024
Slc39a8	NM_001135149	0.000271	-12.0634	4.15692	6.32579	0.222385	-2.16887	-4.4967
Gpr124	NM_054044	4.30E-05	-19.2407	6.62609	8.79774	0.221957	-2.17165	-4.50538
Rgs16	NM_011267	0.002868	-6.51345	6.4672	8.64367	0.221216	-2.17647	-4.52047
Tmem173	NM_028261	0.004277	-5.84317	7.13105	9.30761	0.221203	-2.17656	-4.52074
Pdgfc	NM_019971	0.000255	-12.2541	5.93544	8.11442	0.220831	-2.17899	-4.52836
Eid2	NM_198425	0.000188	-13.2478	4.92897	7.11426	0.219868	-2.18529	-4.54818
Chic1	NM_009767	1.16E-05	-26.7693	3.43268	5.61984	0.219584	-2.18715	-4.55406
Gm10406	NM_001164727	0.001443	-7.82068	4.18251	6.37048	0.219461	-2.18796	-4.55662
Ctla2a	NM_007796	0.002374	-6.85234	5.71106	7.90006	0.219303	-2.189	-4.55991
Agtr2	NM_007429	6.50E-05	-17.3357	4.12271	6.31251	0.219181	-2.1898	-4.56243
H2afy2	NM_207000	0.000152	-13.9783	4.18168	6.37155	0.21917	-2.18988	-4.56266
Mmd	NM_026178	0.002313	-6.90062	8.21684	10.4071	0.219118	-2.19022	-4.56376
Gpr137b	NM_031999	0.000262	-12.1641	6.57227	8.78637	0.215522	-2.2141	-4.6399
Gm3002	NR_033388	0.001631	-7.57168	3.63563	5.85015	0.215458	-2.21452	-4.64127
Smox	NM_001177833	0.000139	-14.3048	5.79662	8.01185	0.215351	-2.21524	-4.64357

Galm	NM_176963	0.002516	-6.74649	4.37559	6.59639	0.214523	-2.2208	-4.66151
Hoxc5	NM_175730	0.001553	-7.66995	4.49912	6.7208	0.214392	-2.22168	-4.66435
Sash1	NM_175155	2.22E-05	-22.7152	6.85942	9.08292	0.214122	-2.22349	-4.67023
Adam19	NM_009616	0.000576	-9.93575	5.47474	7.69982	0.213887	-2.22508	-4.67537
Man2a1	NM_008549	3.24E-05	-20.6673	7.47791	9.70673	0.213333	-2.22882	-4.6875
Gucy1a2	NM_001033322	0.000926	-8.78425	3.46893	5.69827	0.213256	-2.22934	-4.6892
Spp1	NM_009263	0.004264	-5.84808	9.50799	11.7539	0.210819	-2.24592	-4.74341
Tgfbr3	NM_011578	0.000454	-10.5632	8.26804	10.5212	0.209769	-2.25312	-4.76714
Dkk2	NM_020265	0.001834	-7.34038	4.40231	6.65802	0.209394	-2.25571	-4.77569
Trp53inp1	NM_021897	0.000713	-9.40106	7.96354	10.2256	0.208471	-2.26208	-4.79684
Ptgr1	NM_025968	0.000617	-9.76347	5.87923	8.14328	0.208186	-2.26405	-4.80339
Figf	NM_010216	0.002763	-6.57891	7.43444	9.69879	0.208144	-2.26435	-4.80437
Man1a	NM_008548	0.000306	-11.6895	7.07194	9.34011	0.207593	-2.26817	-4.81712
Sema3a	NM_009152	9.71E-05	-15.6625	7.06649	9.33672	0.207296	-2.27023	-4.82401
Rab3il1	NM_144538	0.0012	-8.20958	5.53041	7.80264	0.207009	-2.27224	-4.83071
Slco2a1	NM_033314	0.004121	-5.90309	5.34688	7.62275	0.206489	-2.27587	-4.84288
Unc93b1	NM_019449	0.004266	-5.84763	4.43277	6.71031	0.20625	-2.27754	-4.8485
Hspb1	NM_013560	0.00011	-15.1805	5.1782	7.45976	0.205677	-2.28155	-4.862
Pappa	NM_021362	0.001225	-8.16537	4.1901	6.4742	0.205312	-2.28411	-4.87063
Mir300	NR_029651	0.000136	-14.3734	3.00632	5.30184	0.203695	-2.29552	-4.9093
Nfia	NM_010905	0.00034	-11.3829	5.9083	8.20887	0.202982	-2.30058	-4.92654
Pla2r1	NM_008867	0.002967	-6.45399	4.40956	6.71943	0.201679	-2.30987	-4.95838
Tnfsf13b	NM_033622	0.00109	-8.41771	4.38922	6.69942	0.201632	-2.31021	-4.95954
Tll1	NM_009390	0.000238	-12.4638	3.66135	5.98336	0.199988	-2.32202	-5.0003
Gm13139	NM_001083918	5.65E-05	-17.9584	5.58871	7.9111	0.199937	-2.32238	-5.00158
Folh1	NM_016770	1.43E-05	-25.3862	4.1425	6.46552	0.199849	-2.32302	-5.00378
Glt8d2	NM_029102	3.85E-05	-19.781	4.44439	6.76853	0.199694	-2.32414	-5.00767
Prickle1	NM_001033217	1.54E-05	-24.9226	4.85614	7.18463	0.199092	-2.3285	-5.02281
Hspb1	NM_013560	0.000187	-13.2626	5.13204	7.46744	0.198141	-2.3354	-5.04691
Gm3002	NR_033388	0.002524	-6.74089	3.69907	6.03502	0.198065	-2.33596	-5.04885
Adhfe1	NM_175236	0.001312	-8.01845	4.66148	6.9982	0.19796	-2.33672	-5.05153

Cxx1c	NM_028375	0.000558	-10.0191	5.68598	8.02286	0.197938	-2.33688	-5.0521
Gstm5	NM_010360	0.002013	-7.161	6.08939	8.43257	0.197075	-2.34318	-5.0742
Cpz	NM_153107	0.003166	-6.34191	5.15271	7.49877	0.196682	-2.34606	-5.08434
Cd1d1	NM_007639	0.000805	-9.11088	5.84627	8.19412	0.196439	-2.34785	-5.09064
Kank1	NM_181404	6.88E-06	-30.5016	4.69756	7.05827	0.194696	-2.36071	-5.13622
Col6a1	NM_009933	0.000114	-15.0417	8.42222	10.7868	0.194174	-2.36458	-5.15002
Osr1	NM_011859	6.65E-05	-17.2332	4.49094	6.85615	0.194088	-2.36521	-5.15229
Papss2	NM_011864	4.53E-05	-18.9922	4.18217	6.55331	0.193293	-2.37114	-5.17349
Gm3002	NR_033388	0.001307	-8.02695	3.58182	5.95465	0.193066	-2.37284	-5.17958
Slc39a14	NM_001135151	0.001818	-7.35735	7.5839	9.95794	0.192905	-2.37404	-5.1839
Tnfaip6	NM_009398	0.001652	-7.54651	5.86595	8.24107	0.192762	-2.37511	-5.18775
2810055F11Rik	NM_026038	0.000127	-14.6152	4.29341	6.68452	0.190636	-2.39111	-5.24561
Cmtm7	NM_133978	6.16E-05	-17.5697	4.63786	7.03064	0.190414	-2.39279	-5.2517
Shc2	NM_001024539	0.000995	-8.62105	4.35033	6.74956	0.189566	-2.39923	-5.27521
Nqo1	NM_008706	2.55E-05	-21.9461	4.59376	6.99536	0.189255	-2.40159	-5.28386
Gm3002	NR_033388	0.001529	-7.70208	3.53021	5.93279	0.189125	-2.40258	-5.2875
F3	NM_010171	0.000733	-9.33565	7.94232	10.3496	0.188515	-2.40725	-5.30462
Spats2l	NM_144882	3.01E-05	-21.043	5.19184	7.60416	0.187854	-2.41232	-5.32329
Procr	NM_011171	0.004307	-5.83216	4.38998	6.80616	0.187352	-2.41618	-5.33756
Lrrn4cl	NM_001013019	0.000384	-11.0301	5.76665	8.1887	0.186591	-2.42205	-5.35931
Fam26e	NM_178908	0.002036	-7.13965	5.03553	7.469	0.185119	-2.43347	-5.40192
Abhd6	NM_025341	1.14E-05	-26.8683	5.00773	7.44568	0.184547	-2.43794	-5.41869
Negr1	NM_001039094	0.001319	-8.00788	5.26717	7.70583	0.184455	-2.43866	-5.42138
Gsdmd	NM_026960	0.000149	-14.0574	4.5357	6.9782	0.183964	-2.44251	-5.43585
Mid1	NM_010797	0.000689	-9.4869	5.88182	8.33267	0.182903	-2.45085	-5.46739
Gstm4	NM_026764	9.81E-05	-15.6192	4.87782	7.3397	0.18151	-2.46188	-5.50934
Tgfb2	NM_009367	0.001666	-7.52962	4.62623	7.1054	0.179348	-2.47917	-5.57576
Anpep	NM_008486	0.003379	-6.23093	4.33197	6.81803	0.178493	-2.48606	-5.60246
Gm3002	NR_033388	0.000518	-10.211	3.81705	6.30641	0.178086	-2.48936	-5.61527
Ror2	NM_013846	0.000405	-10.8817	5.60006	8.10088	0.176677	-2.50081	-5.66005
Bhlhb9	NM_198161	0.000217	-12.7725	3.53071	6.03171	0.176654	-2.501	-5.66078

Has1	NM_008215	0.005376	-5.48636	4.86783	7.37315	0.176126	-2.50532	-5.67775
Abca8b	NM_013851	0.001441	-7.82405	3.50019	6.01139	0.17541	-2.5112	-5.70092
Grb14	NM_016719	0.000831	-9.03693	4.67487	7.19196	0.174696	-2.51708	-5.72424
Hvcn1	NM_001042489	7.57E-05	-16.6803	4.00978	6.52917	0.174416	-2.5194	-5.73342
Col12a1	NM_007730	0.000884	-8.89052	5.91931	8.44443	0.173726	-2.52511	-5.75619
Cygb	NM_030206	8.19E-05	-16.3481	6.59011	9.14383	0.170315	-2.55372	-5.87148
	---	0.000182	-13.358	4.82036	7.37671	0.170005	-2.55635	-5.88217
Mtmr11	NM_181409	5.05E-05	-18.4777	5.68546	8.24589	0.169525	-2.56043	-5.89882
Egln3	NM_028133	9.10E-05	-15.9192	3.8392	6.40392	0.169022	-2.56472	-5.91639
Birc3	NM_007464	0.000215	-12.7891	4.81985	7.38625	0.168825	-2.5664	-5.92329
Gm3696	NM_001024712	0.000939	-8.75195	3.77718	6.34507	0.168651	-2.56789	-5.92941
Snai1	NM_011427	0.000145	-14.1551	5.70456	8.27511	0.16834	-2.57055	-5.94037
Nynrin	NM_001040072	2.86E-05	-21.3324	4.56174	7.13663	0.167834	-2.57489	-5.95827
Lyn	NM_001111096	0.002124	-7.05883	4.1698	6.74809	0.167439	-2.57829	-5.97231
Fam110c	NM_027828	0.000285	-11.903	4.26856	6.85177	0.16687	-2.5832	-5.99269
Timp1	NM_001044384	0.001329	-7.99165	8.87147	11.4591	0.166358	-2.58763	-6.01112
Tnfrsf23	NM_024290	0.001918	-7.25363	6.20218	8.79768	0.165454	-2.5955	-6.04398
Gas1	NM_008086	0.00025	-12.3105	8.37085	10.9664	0.165452	-2.59551	-6.04403
Ppap2a	NM_008247	0.001621	-7.5841	7.6787	10.2771	0.165119	-2.59842	-6.05624
S100a1	NM_011309	0.000182	-13.3582	4.6585	7.2575	0.165053	-2.599	-6.05866
Fam115c	BC011487	7.68E-06	-29.6762	3.97169	6.58431	0.163502	-2.61262	-6.11613
Eif4g3	NM_172703	1.46E-05	-25.25	4.8122	7.43002	0.162914	-2.61782	-6.13821
Tnfrsf10b	NM_020275	0.00172	-7.46582	4.26334	6.88161	0.162863	-2.61827	-6.14013
Zfp521	NM_145492	0.000155	-13.9066	6.03219	8.65782	0.162034	-2.62563	-6.17153
Tpm2	NM_009416	0.00067	-9.55456	5.07107	7.70007	0.161656	-2.629	-6.18599
B3gnt9-ps	NM_178879	0.000112	-15.0931	5.86864	8.49947	0.161451	-2.63083	-6.19381
Unc5c	NM_009472	0.000194	-13.1383	5.49048	8.1269	0.160827	-2.63642	-6.21786
Fstl1	NM_008047	2.39E-05	-22.3209	9.31649	11.9529	0.160824	-2.63644	-6.21797
Ccdc122	NM_175369	0.000111	-15.1271	2.9098	5.56414	0.158841	-2.65434	-6.29559
Cyp2d22	NM_001163472	0.000117	-14.9525	5.21712	7.87401	0.15856	-2.6569	-6.30675
Nid2	NM_008695	0.000342	-11.3656	5.34894	8.00726	0.158403	-2.65832	-6.313

Nfia	NM_001122952	0.000142	-14.2207	5.19869	7.86137	0.157926	-2.66268	-6.33208
Cgnl1	NM_026599	0.00013	-14.5424	4.95069	7.61401	0.157855	-2.66332	-6.33491
Rasl11b	NM_026878	0.000113	-15.0663	5.54426	8.20803	0.157806	-2.66377	-6.33688
Trim12a	NM_023835	7.11E-05	-16.9451	3.65238	6.32232	0.157133	-2.66994	-6.36402
S1pr1	NM_007901	0.003459	-6.19164	4.79927	7.47293	0.156728	-2.67367	-6.38049
Rarres2	NM_027852	0.003904	-5.99115	4.8759	7.5554	0.156096	-2.6795	-6.40632
Pdgfd	NM_027924	9.95E-05	-15.5639	3.53687	6.21782	0.155938	-2.68095	-6.41279
Taf9b	NM_001167988	0.000363	-11.1942	3.61359	6.30064	0.155281	-2.68704	-6.43992
Tbc1d2b	NM_194334	9.60E-05	-15.7066	6.43535	9.13287	0.154158	-2.69752	-6.48685
Ppp1r3c	NM_016854	0.000829	-9.04158	3.74588	6.446	0.153879	-2.70013	-6.4986
Fmo1	NM_010231	0.000401	-10.9104	4.92277	7.62628	0.153519	-2.70351	-6.51383
Dbc1	NM_019967	0.000324	-11.5222	4.68297	7.38809	0.153348	-2.70512	-6.52111
Boc	NM_172506	0.000899	-8.85177	4.45148	7.16108	0.152872	-2.7096	-6.54141
Nsd1	NM_008739	0.000663	-9.58225	4.82159	7.53128	0.152863	-2.70969	-6.54181
Cyp2f2	NM_007817	0.000549	-10.0615	4.92067	7.63089	0.152807	-2.71022	-6.54421
9930013L23Rik	NM_030728	0.001811	-7.36437	4.56262	7.2981	0.150154	-2.73548	-6.65982
Top1mt	NM_028404	2.20E-05	-22.7784	4.58317	7.31992	0.150022	-2.73675	-6.66567
Cbr1	NM_007620	0.00028	-11.9649	5.51377	8.27045	0.147964	-2.75668	-6.7584
Cd24a	NM_009846	0.003767	-6.04975	3.50993	6.27428	0.14718	-2.76435	-6.79442
Smpdl3a	NM_020561	0.000788	-9.16082	3.91725	6.69169	0.146154	-2.77444	-6.8421
Ano3	NM_001128103	0.004117	-5.90447	2.93264	5.7162	0.145233	-2.78356	-6.88549
Ophn1	NM_052976	6.39E-06	-31.0758	4.45402	7.2401	0.14498	-2.78607	-6.89749
Stc1	NM_009285	0.001038	-8.52692	4.65042	7.43887	0.144741	-2.78845	-6.90889
Fap	NM_007986	0.002071	-7.10658	3.25277	6.04627	0.144236	-2.7935	-6.93309
Mmp19	NM_021412	0.000276	-11.9992	7.89812	10.6946	0.143936	-2.7965	-6.94753
Slc16a3	NM_001038653	0.00103	-8.54486	5.56573	8.36782	0.143379	-2.80209	-6.97451
Isyna1	NM_023627	0.002668	-6.64155	5.87075	8.67554	0.143112	-2.80479	-6.98755
Serping1	NM_009776	0.0026	-6.68744	6.25956	9.06514	0.143033	-2.80558	-6.99139
Nynrin	NM_001040072	0.000152	-13.9872	5.21871	8.02546	0.142918	-2.80674	-6.99703
Gm3002	NR_033388	0.001424	-7.8481	3.34123	6.15189	0.14253	-2.81066	-7.01608
Iah1	NM_026347	0.000374	-11.1058	5.93749	8.75032	0.142316	-2.81283	-7.02663

Xlr3b	NM_001081643	0.000297	-11.7838	3.19064	6.0119	0.141487	-2.82126	-7.0678
P4ha3	NM_177161	0.001396	-7.88868	4.42448	7.24776	0.141288	-2.82329	-7.07772
Ism1	NM_001126490	0.002306	-6.90628	5.14618	7.97186	0.141055	-2.82567	-7.08944
Runx1t1	NM_001111027	0.00066	-9.59247	4.16315	6.99114	0.140829	-2.82799	-7.10083
Ctsf	NM_019861	0.000697	-9.45774	4.86226	7.69649	0.140221	-2.83423	-7.13161
Ppap2b	NM_080555	6.61E-05	-17.261	8.73608	11.5746	0.1398	-2.83856	-7.15306
Eda2r	NM_001161432	0.000675	-9.53768	5.8377	8.68432	0.139021	-2.84662	-7.19314
Adam23	NM_011780	2.62E-05	-21.7944	3.9796	6.84199	0.137511	-2.86239	-7.27217
Crabp1	NM_013496	7.29E-06	-30.0651	5.29941	8.16415	0.137287	-2.86474	-7.28403
Tceal1	NM_146236	0.000141	-14.2559	3.05411	5.91998	0.137179	-2.86587	-7.28976
Ace	NM_207624	0.000501	-10.299	4.64612	7.52181	0.136248	-2.87569	-7.33955
Plekhh2	NM_177606	0.000122	-14.7817	4.26789	7.1501	0.135634	-2.88221	-7.37278
Dhrs3	NM_011303	0.000283	-11.9321	5.40979	8.29405	0.135441	-2.88427	-7.38331
Cbr3	NM_173047	8.56E-05	-16.1674	4.17255	7.06002	0.13514	-2.88747	-7.39971
Plekha2	NM_031257	5.47E-06	-32.3172	5.45477	8.3436	0.135013	-2.88883	-7.4067
Kcnab1	NM_010597	0.001327	-7.99515	4.8975	7.7869	0.13496	-2.8894	-7.40961
Egfl6	NM_019397	0.002972	-6.45119	3.8099	6.70136	0.134768	-2.89145	-7.42018
Fgf10	NM_008002	0.000323	-11.5356	4.24637	7.14098	0.134473	-2.89461	-7.43645
Col6a3	AF064749	0.000399	-10.926	7.99092	10.8876	0.13428	-2.89669	-7.44715
	---	0.000219	-12.7351	3.01397	5.9132	0.134043	-2.89923	-7.46028
Gm3002	NR_033388	0.001831	-7.34354	4.41762	7.32156	0.133606	-2.90394	-7.48468
Trim25	NM_009546	8.62E-07	-51.3246	3.8125	6.71668	0.133584	-2.90418	-7.48592
Abca9	NM_147220	0.000364	-11.1805	4.14659	7.05546	0.133151	-2.90887	-7.5103
Htatip2	NM_001146049	5.09E-05	-18.4359	5.08108	8.00277	0.131972	-2.92169	-7.57734
Hoxd8	NM_008276	8.81E-05	-16.0496	3.75971	6.68383	0.13175	-2.92413	-7.59015
Dab2	NM_023118	0.000186	-13.2679	7.21491	10.1436	0.131337	-2.92865	-7.61398
	---	0.000135	-14.4179	3.40088	6.32982	0.131311	-2.92895	-7.61553
Aox3	NM_023617	0.003635	-6.10887	5.34344	8.28388	0.130268	-2.94044	-7.67647
Ndrg2	NM_013864	0.000343	-11.3588	4.93784	7.88684	0.129498	-2.94899	-7.7221
Dact1	NM_001190466	8.97E-05	-15.977	3.85114	6.80234	0.1293	-2.95121	-7.73395
Enpep	NM_007934	0.000744	-9.2994	4.30961	7.29141	0.126587	-2.9818	-7.8997

Echdc2	NM_026728	0.000272	-12.0537	4.86641	7.8494	0.126482	-2.983	-7.90626
Chic1	NM_009767	9.18E-06	-28.3764	3.61306	6.60224	0.125941	-2.98918	-7.9402
Mark1	NM_145515	1.04E-05	-27.471	4.45567	7.46224	0.124432	-3.00658	-8.03655
Adamts1	NM_009621	0.001618	-7.58793	8.00885	11.0219	0.12387	-3.0131	-8.07295
Lphn2	NM_001081298	0.000409	-10.8504	4.63592	7.65278	0.123547	-3.01686	-8.09406
Manba	NM_027288	7.26E-05	-16.8576	5.31428	8.3341	0.123294	-3.01982	-8.11067
Mir382	NR_029883	0.000168	-13.6288	2.9204	5.96235	0.121418	-3.04195	-8.23601
Fam49a	NM_029758	0.000484	-10.3939	3.62044	6.66782	0.120961	-3.04738	-8.26711
Pde1a	NM_016744	4.91E-05	-18.6121	4.71852	7.76717	0.120855	-3.04865	-8.27437
B3galnt1	NM_020026	0.000581	-9.91253	4.00898	7.05871	0.120764	-3.04974	-8.28061
Hoxc4	NM_013553	0.000394	-10.9573	4.64059	7.69502	0.120372	-3.05442	-8.30756
Lphn2	NM_001081298	0.0001	-15.5327	3.83563	6.89578	0.119896	-3.06015	-8.34059
St3gal1	NM_009177	4.91E-05	-18.607	6.82574	9.89119	0.119456	-3.06545	-8.37128
Nynrin	NM_001040072	0.0002	-13.0338	4.56671	7.63277	0.119405	-3.06606	-8.37486
Vcam1	NM_011693	9.10E-05	-15.9217	8.32775	11.4057	0.118427	-3.07793	-8.44405
Ccnb1ip1	NM_001111119	5.21E-05	-18.3291	4.20406	7.29655	0.117238	-3.09249	-8.52967
C530030P08Rik	ENSMUST0000010138<br>1	0.000133	-14.4479	5.07095	8.16585	0.117042	-3.09491	-8.54397
Ptgis	NM_008968	5.14E-05	-18.3932	5.68496	8.78468	0.116652	-3.09972	-8.57252
Mafb	NM_010658	0.000601	-9.82608	4.77785	7.89945	0.114895	-3.12161	-8.70357
Rpl39l	NM_026594	0.000436	-10.6744	3.55127	6.6744	0.114774	-3.12313	-8.71276
Il11ra2	NM_010550	3.55E-05	-20.1882	5.63509	8.75836	0.114763	-3.12327	-8.71359
Il11ra2	NM_010550	3.55E-05	-20.1882	5.63509	8.75836	0.114763	-3.12327	-8.71359
Il1rap	NM_008364	5.45E-05	-18.1237	5.73117	8.86574	0.113867	-3.13457	-8.78214
Calcrl	NM_018782	0.00111	-8.37813	5.41386	8.56922	0.112239	-3.15536	-8.90958
Arsj	NM_173451	6.15E-05	-17.5756	4.66899	7.82507	0.112182	-3.15608	-8.91405
	---	3.90E-05	-19.7173	5.08086	8.24158	0.111822	-3.16072	-8.94278
Sema5a	NM_009154	7.50E-05	-16.7211	5.05845	8.22527	0.111351	-3.16682	-8.98064
Myl9	NM_172118	0.006339	-5.24018	5.22348	8.41208	0.109682	-3.1886	-9.11724
Fuca2	NM_025799	0.000432	-10.704	4.37916	7.5725	0.109323	-3.19333	-9.14722
Nfatc4	NM_023699	0.000231	-12.5695	4.86552	8.06029	0.109214	-3.19477	-9.15635

Rbpms	NM_019733	6.17E-05	-17.5643	4.19255	7.39072	0.108957	-3.19817	-9.17795
Rora	NM_013646	0.000111	-15.1329	4.07657	7.27702	0.108785	-3.20045	-9.19248
Vav3	NM_020505	0.001427	-7.84334	4.0051	7.23554	0.106547	-3.23044	-9.38555
Irak3	NM_028679	9.38E-05	-15.7996	3.94657	7.18001	0.106326	-3.23343	-9.40503
Fam198b	NM_133187	0.001976	-7.19641	5.56048	8.80134	0.10578	-3.24086	-9.4536
Kdm5d	NM_011419	2.24E-05	-22.6858	4.09336	7.33456	0.105755	-3.2412	-9.45583
Tbx5	NM_011537	2.82E-05	-21.3935	3.91107	7.15813	0.105326	-3.24706	-9.49432
Gsta4	NM_010357	0.000526	-10.1701	4.26313	7.51488	0.104985	-3.25175	-9.5252
Mir410	NR_029914	0.000102	-15.4731	3.31626	6.58404	0.103824	-3.26778	-9.63164
Flnc	NM_001081185	1.20E-05	-26.5267	5.48065	8.76929	0.102334	-3.28864	-9.77189
Adm	NM_009627	0.000645	-9.65016	4.26596	7.55635	0.102211	-3.29038	-9.78372
Adam12	NM_007400	0.000242	-12.4179	4.79855	8.08912	0.102197	-3.29057	-9.78498
Eya4	NM_010167	5.71E-06	-31.969	4.67825	7.99552	0.100323	-3.31728	-9.9678
Epha7	NM_010141	0.000213	-12.8283	3.90819	7.23042	0.099979	-3.32223	-10.0021
C530030P08Rik	ENSMUST0000010138 1	9.13E-05	-15.9077	5.07886	8.40154	0.099948	-3.32268	-10.0052
Ldb2	NM_001077398	4.64E-05	-18.8753	4.0152	7.34149	0.099698	-3.32629	-10.0303
Col6a2	NM_146007	0.00021	-12.8704	6.52818	9.8565	0.099558	-3.32832	-10.0444
Prl2c3	NM_011118	0.000796	-9.13614	3.73685	7.06966	0.099249	-3.33281	-10.0757
Ggt5	NM_011820	0.000111	-15.1222	5.22224	8.55538	0.099226	-3.33314	-10.078
Olr1	NM_138648	0.002411	-6.82417	3.94351	7.27882	0.099077	-3.33531	-10.0932
Clec2d	NM_053109	0.000407	-10.8664	3.80356	7.14127	0.098912	-3.33772	-10.11
Fads2	NM_019699	4.71E-05	-18.8044	5.14382	8.49734	0.097834	-3.35352	-10.2214
Bdkrb1	NM_007539	0.003125	-6.36408	4.98921	8.35693	0.096876	-3.36772	-10.3225
Fbln5	NM_011812	4.73E-05	-18.7846	5.3494	8.72164	0.096573	-3.37224	-10.3549
Plxdc1	NM_001163608	0.000641	-9.66579	4.65887	8.04148	0.095881	-3.38261	-10.4296
Lamb1	NM_008482	1.52E-05	-24.9926	7.34693	10.7357	0.095474	-3.38874	-10.474
Tspan11	NM_026743	0.000822	-9.06064	5.1688	8.56014	0.095302	-3.39134	-10.4929
Adh1	NM_007409	9.95E-05	-15.5633	7.15265	10.5482	0.095028	-3.3955	-10.5232
Postn	NM_015784	0.000219	-12.729	8.90871	12.3049	0.094982	-3.3962	-10.5283
Jam2	NM_023844	0.000459	-10.534	4.393	7.79125	0.094847	-3.39825	-10.5433

Serpinf1	NM_011340	4.86E-06	-33.2803	8.15589	11.5605	0.09443	-3.40461	-10.5899
Akap12	NM_031185	0.001198	-8.21335	5.23965	8.64468	0.094403	-3.40503	-10.5929
Bmper	NM_028472	0.000592	-9.86507	6.61391	10.0347	0.093377	-3.42079	-10.7093
Hsd11b1	NM_008288	0.006801	-5.13787	4.68929	8.11394	0.093128	-3.42465	-10.7379
Plau	NM_008873	0.000463	-10.5151	4.00928	7.44137	0.092648	-3.43209	-10.7935
Thbd	NM_009378	1.98E-05	-23.3809	4.85908	8.29462	0.092427	-3.43554	-10.8194
Gata6	NM_010258	0.000291	-11.8415	4.83614	8.27269	0.092362	-3.43656	-10.827
Il11ra1	NM_001172054	8.59E-06	-28.8496	5.54407	8.98854	0.091857	-3.44447	-10.8865
Islr	NM_012043	0.000321	-11.5452	6.46524	9.92435	0.090929	-3.45911	-10.9976
Mfap2	NM_008546	1.09E-05	-27.1942	5.97232	9.43389	0.090775	-3.46156	-11.0163
Csgalnact1	NM_172753	1.53E-05	-24.9719	5.95178	9.41338	0.090773	-3.4616	-11.0165
Setbp1	NM_053099	0.00013	-14.5439	4.70203	8.16581	0.090636	-3.46378	-11.0332
Prl2c5	NM_181852	0.000143	-14.1917	3.75189	7.21601	0.090614	-3.46412	-11.0358
Svep1	NM_022814	0.000109	-15.2007	4.65215	8.1318	0.089644	-3.47965	-11.1552
Tmem100	NM_026433	0.00085	-8.98399	4.01885	7.50682	0.089129	-3.48797	-11.2197
Galntl4	NM_173739	1.43E-05	-25.3835	4.44169	7.94162	0.088393	-3.49992	-11.3131
Hs6st2	NM_001077202	0.000297	-11.7808	4.35546	7.87504	0.087197	-3.51958	-11.4683
Htr2a	NM_172812	0.000384	-11.0317	4.37133	7.92319	0.085267	-3.55186	-11.7278
Itga11	NM_176922	1.84E-05	-23.8363	4.6194	8.20938	0.083044	-3.58997	-12.0418
Mrc2	NM_008626	8.40E-05	-16.2456	5.3918	8.98788	0.082694	-3.59608	-12.0928
Gstk1	NM_029555	4.13E-05	-19.4383	3.70174	7.29901	0.082625	-3.59727	-12.1028
Abca8a	NM_153145	0.003199	-6.32387	3.75796	7.35573	0.082597	-3.59777	-12.107
H2-D1	NM_010380	5.41E-05	-18.1538	5.69361	9.3079	0.081656	-3.61429	-12.2465
Prrx2	NM_009116	3.79E-05	-19.8678	5.49448	9.11401	0.08136	-3.61953	-12.291
	---	9.16E-05	-15.8948	4.03275	7.65739	0.081073	-3.62464	-12.3346
	---	0.000198	-13.0696	4.48982	8.11695	0.080933	-3.62714	-12.356
Spon2	NM_133903	0.000626	-9.7236	6.98726	10.6226	0.080473	-3.63535	-12.4265
Emilin2	NM_145158	2.07E-05	-23.1323	4.68668	8.33549	0.079726	-3.64881	-12.543
Rerg	NM_181988	0.000735	-9.32733	4.44315	8.0974	0.079426	-3.65425	-12.5904
Olfml3	NM_133859	7.69E-07	-52.8261	7.42628	11.0885	0.078989	-3.6622	-12.6599
Ppap2c	NM_015817	0.000186	-13.276	4.96723	8.63891	0.078472	-3.67168	-12.7434

Lgals3	NM_001145953	1.09E-06	-48.4171	5.58926	9.2705	0.077954	-3.68124	-12.8281
Cd248	NM_054042	5.13E-05	-18.3998	5.41462	9.10427	0.077501	-3.68965	-12.9031
Cd97	NM_011925	0.001098	-8.40174	4.47661	8.17215	0.077185	-3.69554	-12.9559
6330406I15Rik	BC116246	0.001007	-8.59414	4.16239	7.86685	0.076709	-3.70447	-13.0363
Igfbp6	NM_008344	9.45E-05	-15.7668	7.24247	10.9504	0.076523	-3.70797	-13.068
Itga8	NM_001001309	0.000602	-9.82408	3.87373	7.60399	0.07535	-3.73025	-13.2715
Slc7a2	NM_007514	0.0012	-8.20834	4.3829	8.13314	0.074313	-3.75024	-13.4566
Plxdc2	NM_026162	9.48E-05	-15.7538	4.25926	8.01896	0.073827	-3.7597	-13.5451
Dnm1	NM_010065	1.76E-06	-42.9012	5.41765	9.18863	0.073252	-3.77098	-13.6514
D930014E17Rik	NM_020616	8.95E-06	-28.5551	3.53036	7.30502	0.073066	-3.77465	-13.6862
Hoxc6	NM_010465	0.000151	-13.9913	4.59634	8.37562	0.072833	-3.77927	-13.7301
Prkg1	NM_001013833	1.60E-05	-24.6914	4.06023	7.84316	0.072648	-3.78294	-13.765
Fst	NM_008046	2.03E-07	-73.6828	4.88003	8.68007	0.071792	-3.80004	-13.9292
Ociad2	NM_026950	0.000648	-9.64014	3.7579	7.58841	0.070292	-3.83051	-14.2265
Sgcd	NM_011891	4.70E-05	-18.818	3.46166	7.29992	0.069915	-3.83826	-14.3031
Npy1r	NM_010934	6.36E-05	-17.4288	3.13438	6.97731	0.069689	-3.84293	-14.3495
Sned1	NM_172463	1.06E-05	-27.3686	5.22485	9.08619	0.068805	-3.86134	-14.5338
Lifr	NM_013584	5.50E-05	-18.0801	3.69933	7.56478	0.06861	-3.86545	-14.5752
Podn	NM_172874	0.000115	-15.0129	4.89906	8.76576	0.06855	-3.8667	-14.5879
Cd82	NM_007656	4.51E-06	-33.9114	5.9142	9.80476	0.067426	-3.89055	-14.8311
1110036O03Rik	NM_176830	7.59E-06	-29.7604	4.77492	8.66778	0.067318	-3.89285	-14.8548
Ripk3	NM_019955	3.00E-05	-21.0739	5.02725	8.92048	0.067301	-3.89323	-14.8586
Ltbp2	NM_013589	0.000248	-12.336	5.02895	8.93702	0.066612	-3.90807	-15.0123
Steap4	NM_054098	0.000993	-8.62597	4.54338	8.45273	0.066553	-3.90935	-15.0256
Serpine2	NM_009255	0.000306	-11.6908	4.51178	8.42716	0.066276	-3.91538	-15.0885
Tnc	NM_011607	0.00037	-11.1326	6.46753	10.4051	0.065263	-3.9376	-15.3227
Lphn2	NM_001081298	3.91E-05	-19.7048	4.68671	8.62967	0.065021	-3.94296	-15.3798
Fam129a	NM_022018	0.000476	-10.4384	4.56364	8.51732	0.064539	-3.95368	-15.4944
C4b	NM_009780	0.000284	-11.9125	4.47421	8.43203	0.064354	-3.95782	-15.539
Sfrp4	NM_016687	0.001201	-8.20813	3.78667	7.75162	0.064037	-3.96495	-15.616
Raet1d	NM_020030	0.002151	-7.03554	4.1048	8.11486	0.062066	-4.01006	-16.1119

Lphn2	NM_001081298	0.000227	-12.6263	3.34928	7.37188	0.061529	-4.0226	-16.2526
Stox2	NM_001114311	9.17E-06	-28.3856	2.94683	6.97402	0.061333	-4.02719	-16.3044
Apod	NM_007470	0.000601	-9.82729	4.8985	8.92855	0.061212	-4.03005	-16.3367
Hoxc8	NM_010466	0.000156	-13.8747	5.26295	9.3013	0.060861	-4.03835	-16.431
Rab34	NM_033475	3.75E-05	-19.9167	5.00902	9.05058	0.060725	-4.04157	-16.4677
Adamts9	NM_175314	0.002839	-6.53096	3.33354	7.37541	0.060712	-4.04187	-16.4711
Nid1	NM_010917	1.50E-05	-25.0669	7.50876	11.5526	0.060627	-4.04389	-16.4943
Tfpi	NM_011576	0.000165	-13.6945	4.84059	8.89431	0.060216	-4.05372	-16.607
Ar	NM_013476	0.000108	-15.2438	3.32113	7.38851	0.059648	-4.06738	-16.765
Lpl	NM_008509	7.58E-05	-16.6725	4.10474	8.17405	0.059568	-4.06931	-16.7875
Decr1	NM_026172	5.69E-06	-31.9895	3.89356	7.98423	0.058693	-4.09067	-17.0379
Ptgfr	NM_008966	0.000112	-15.1199	4.31507	8.419	0.058156	-4.10393	-17.1952
Gtpbp10	NM_153116	1.24E-05	-26.312	3.54379	7.65141	0.058007	-4.10762	-17.2392
Srpx2	NM_026838	0.000872	-8.92401	4.36032	8.47696	0.057646	-4.11664	-17.3473
Gtpbp10	NM_153116	2.84E-06	-38.0806	3.56619	7.70576	0.056737	-4.13957	-17.6253
Sulf2	NM_028072	6.12E-05	-17.6028	4.44992	8.59243	0.056621	-4.14251	-17.6612
Cyba	NM_007806	4.16E-05	-19.4031	5.11797	9.26591	0.056409	-4.14794	-17.7278
Ass1	NM_007494	0.000146	-14.1137	5.63652	9.80007	0.055801	-4.16355	-17.9207
Ass1	NM_007494	0.00014	-14.2714	5.52356	9.69074	0.055661	-4.16718	-17.9658
Adamts15	NM_001024139	4.20E-05	-19.3554	4.53409	8.70854	0.055382	-4.17444	-18.0565
D14Abb1e	NM_001114879	1.13E-06	-47.933	3.47297	7.64884	0.055327	-4.17587	-18.0744
Cyp1b1	NM_009994	0.00016	-13.7939	4.18022	8.35636	0.055317	-4.17614	-18.0777
Il1r1	NM_008362	1.98E-05	-23.3846	6.00101	10.1967	0.054572	-4.19569	-18.3243
Atp8b1	NM_001001488	5.68E-05	-17.9342	3.8773	8.07882	0.054352	-4.20152	-18.3985
Cpxm1	NM_019696	0.001338	-7.97699	4.72541	8.93058	0.054215	-4.20517	-18.4451
Igsf10	NM_001162884	2.33E-05	-22.4517	4.44123	8.65199	0.054005	-4.21077	-18.5169
Nt5e	NM_011851	3.90E-06	-35.1718	4.51859	8.78396	0.051999	-4.26537	-19.2311
Ptges	NM_022415	6.46E-05	-17.3599	4.90475	9.17019	0.051997	-4.26544	-19.2321
Uty	NM_009484	1.31E-05	-25.9614	3.11231	7.39429	0.051404	-4.28198	-19.4538
Fgl2	NM_008013	5.27E-07	-58.0717	3.71035	8.02036	0.050415	-4.31	-19.8353
Slc43a3	NM_021398	0.000126	-14.6608	4.70352	9.0225	0.050102	-4.31898	-19.9592

Dram1	NM_027878	3.12E-06	-37.1798	5.87372	10.2036	0.049724	-4.32991	-20.111
Lrrc32	NM_001113379	5.63E-05	-17.9732	4.60298	8.93589	0.049621	-4.33291	-20.1528
Pdgfrl	NM_026840	6.62E-05	-17.2537	4.33199	8.68803	0.048832	-4.35604	-20.4786
Il1rl1	NM_001025602	0.004476	-5.77071	4.41429	8.78193	0.048441	-4.36764	-20.6439
Grem2	NM_011825	3.47E-05	-20.3136	5.0736	9.45181	0.048087	-4.37821	-20.7956
Gfpt2	NM_013529	0.00039	-10.9853	4.44715	8.82908	0.047963	-4.38192	-20.8492
Car13	NM_024495	2.04E-06	-41.3965	3.65712	8.05364	0.047481	-4.39651	-21.0612
Myh10	NM_175260	8.80E-05	-16.0536	4.66394	9.0641	0.047361	-4.40016	-21.1145
Inhba	NM_008380	0.000722	-9.37109	4.38051	8.85112	0.045104	-4.47062	-22.1712
Abca1	NM_013454	0.000736	-9.32447	4.60373	9.0776	0.045002	-4.47387	-22.2213
Hsd17b11	NM_053262	4.04E-06	-34.8571	4.06677	8.54479	0.044873	-4.47802	-22.2853
Aldh1l2	NM_153543	0.00013	-14.5384	4.23569	8.72199	0.044616	-4.4863	-22.4135
Cck	NM_031161	3.37E-06	-36.4866	4.21468	8.71586	0.044158	-4.50118	-22.6459
Rbp1	NM_011254	5.96E-05	-17.7214	5.22203	9.72572	0.044082	-4.50368	-22.6852
Igsf10	NM_001162884	2.25E-05	-22.662	4.30562	8.81496	0.043909	-4.50933	-22.7743
Agt	NM_007428	2.42E-05	-22.2372	5.42012	9.93017	0.043888	-4.51005	-22.7855
Lrrc17	NM_028977	1.55E-05	-24.8746	5.03103	9.56532	0.043156	-4.53429	-23.1717
Gpnmb	NM_053110	0.000179	-13.4041	5.10381	9.67144	0.04217	-4.56764	-23.7135
Enpp1	NM_008813	0.001394	-7.89209	4.39612	8.97459	0.041855	-4.57847	-23.8923
Ccl8	NM_021443	3.31E-05	-20.5545	3.55152	8.14096	0.041538	-4.58944	-24.0745
9030625A04Rik	BC116748	1.94E-07	-74.5283	3.88568	8.48238	0.041329	-4.5967	-24.1961
Rgs2	NM_009061	1.07E-05	-27.2757	3.18053	7.78879	0.040999	-4.60826	-24.3907
Meg3	NR_003633	1.09E-05	-27.1988	5.18385	9.79735	0.040851	-4.6135	-24.4795
Cd302	NM_025422	9.07E-06	-28.4572	3.5375	8.16455	0.040469	-4.62704	-24.7103
S1pr3	NM_010101	0.000398	-10.9281	4.45599	9.08802	0.040329	-4.63203	-24.7959
Pamr1	NM_173749	0.000268	-12.1001	4.28598	8.94141	0.039681	-4.65543	-25.2013
Dkk3	NM_015814	4.66E-05	-18.8591	5.07348	9.73273	0.039575	-4.65925	-25.2682
Cxcl5	NM_009141	0.006767	-5.14509	4.00192	8.68177	0.039014	-4.67986	-25.6317
Gda	NM_010266	0.000245	-12.3772	4.06029	8.75915	0.038504	-4.69886	-25.9715
Ctsk	NM_007802	2.62E-05	-21.8034	5.2917	9.99082	0.038497	-4.69912	-25.9762
Cd55	NM_010016	0.000267	-12.1099	2.63969	7.34942	0.038215	-4.70973	-26.1679

Bicc1	ENSMUST00000143791	6.84E-06	-30.5447	4.25993	8.97654	0.038033	-4.71661	-26.293
Loxl2	NM_033325	4.76E-05	-18.7556	4.58182	9.31062	0.037713	-4.72881	-26.5163
Il1rl2	NM_133193	6.62E-06	-30.7995	3.4686	8.22975	0.036877	-4.76115	-27.1174
Loxl1	NM_010729	4.85E-07	-59.2804	4.92852	9.71064	0.036345	-4.78212	-27.5144
Enpp3	NM_134005	0.000135	-14.4072	3.70629	8.52374	0.035465	-4.81745	-28.1967
Ndn	NM_010882	6.41E-06	-31.0544	3.98694	8.80678	0.035407	-4.81984	-28.2433
Dapk1	NM_029653	1.55E-05	-24.8682	4.46562	9.30808	0.034856	-4.84246	-28.6898
Cd34	NM_001111059	0.000156	-13.8783	3.85083	8.7207	0.0342	-4.86986	-29.2398
Plac8	NM_139198	2.00E-05	-23.3215	3.97457	8.85866	0.033864	-4.88409	-29.5296
Fbln1	NM_010180	0.000355	-11.2539	5.17859	10.1236	0.032464	-4.94503	-30.8037
Rnase4	NM_021472	6.22E-06	-31.2904	4.84009	9.79385	0.032268	-4.95376	-30.9906
Lphn2	NM_001081298	6.31E-06	-31.1685	4.21704	9.17218	0.032237	-4.95514	-31.0202
Mgp	NM_008597	0.000189	-13.2238	6.20066	11.1595	0.032154	-4.95886	-31.1005
Gria3	NM_016886	2.59E-05	-21.8586	3.43232	8.3914	0.032149	-4.95908	-31.105
Gm10554	ENSMUST00000097674	8.25E-06	-29.1484	2.96903	7.95751	0.031501	-4.98848	-31.7454
Fkbp10	NM_010221	5.83E-07	-56.6012	4.87181	9.86559	0.031385	-4.99378	-31.8623
Gpm6b	NM_001177956	5.73E-05	-17.8978	4.55267	9.56556	0.030972	-5.01289	-32.2872
Igsf10	NM_001162884	3.05E-05	-20.9754	3.80947	8.82638	0.030886	-5.01692	-32.3774
Grb10	NM_010345	0.000138	-14.3307	4.34158	9.36213	0.030808	-5.02055	-32.459
Slit3	NM_011412	2.91E-06	-37.8576	4.89236	9.92624	0.030525	-5.03388	-32.7604
Bicc1	NM_031397	1.21E-05	-26.4958	4.40165	9.50734	0.029043	-5.10569	-34.4323
Ednra	NM_010332	0.000142	-14.2275	4.5362	9.6452	0.028976	-5.109	-34.5113
Efemp1	NM_146015	0.000179	-13.4112	5.018	10.2194	0.027179	-5.20136	-36.7931
Bgn	NM_007542	5.38E-07	-57.751	7.16352	12.374	0.027008	-5.21047	-37.0261
Rgs4	NM_009062	4.52E-05	-18.9957	4.03814	9.26327	0.026735	-5.22513	-37.4042
Lphn2	NM_001081298	2.11E-06	-41.0469	3.99005	9.22236	0.026602	-5.23231	-37.5909
AF357355	NR_028433	4.27E-06	-34.3806	3.84803	9.086	0.026498	-5.23797	-37.7387
Matn2	NM_016762	9.85E-06	-27.8769	4.92097	10.1698	0.0263	-5.24879	-38.0228
Cxcl12	NM_001012477	0.000102	-15.4785	4.52963	9.7975	0.025954	-5.26788	-38.5291
Il33	NM_001164724	0.00086	-8.95438	4.16128	9.43478	0.025853	-5.2735	-38.6797

Pla2g4a	NM_008869	1.16E-06	-47.625	4.07512	9.38048	0.025289	-5.30535	-39.5431
Apoe	NM_009696	2.75E-06	-38.4033	6.8293	12.1701	0.024676	-5.34077	-40.5259
Eif2s3y	NM_012011	1.72E-06	-43.1785	3.13961	8.5366	0.023733	-5.39699	-42.1361
Tgfbi	NM_009369	1.61E-05	-24.6421	5.09706	10.5262	0.023209	-5.42915	-43.0862
Mmp3	NM_010809	0.000394	-10.9569	5.04891	10.5016	0.022833	-5.45273	-43.796
Fbn1	NM_007993	3.39E-06	-36.4186	4.62519	10.0801	0.022798	-5.45495	-43.8637
Adamts5	NM_011782	2.97E-05	-21.1306	3.69062	9.22945	0.02151	-5.53884	-46.4897
Mmp2	NM_008610	1.77E-06	-42.896	6.89646	12.4673	0.021038	-5.57085	-47.5326
Kcne4	NM_021342	2.66E-05	-21.725	4.32018	9.90044	0.020901	-5.58027	-47.844
Thy1	NM_009382	9.12E-06	-28.4228	5.35114	10.9552	0.02056	-5.60402	-48.6383
Fibin	NM_026271	1.79E-05	-23.9817	4.28771	9.89573	0.020503	-5.60802	-48.7733
Scp2	NM_011327	3.21E-05	-20.7106	3.92651	9.54717	0.020324	-5.62066	-49.2025
Cyp7b1	NM_007825	1.91E-05	-23.5922	3.80515	9.43186	0.020239	-5.62671	-49.4094
Cdh11	NM_009866	2.09E-06	-41.1017	4.39748	10.0305	0.02015	-5.63305	-49.6268
Sulf1	NM_001198565	5.75E-05	-17.8793	4.12815	9.80835	0.019503	-5.6802	-51.2756
Nrn1	NM_153529	2.03E-06	-41.4027	4.04859	9.77714	0.01886	-5.72855	-53.0231
Ccdc80	NM_026439	1.25E-05	-26.2581	5.0406	10.8025	0.018429	-5.76191	-54.2636
Gas6	NM_019521	1.62E-06	-43.8047	5.29211	11.0602	0.01835	-5.76805	-54.4951
Cav1	NM_007616	1.42E-07	-80.5333	3.79276	9.57102	0.018221	-5.77826	-54.8821
Igf1	NM_010512	1.00E-05	-27.7489	4.27461	10.1116	0.017494	-5.83701	-57.1629
Has2	NM_008216	3.91E-05	-19.7021	4.0408	9.92159	0.016971	-5.88079	-58.9244
Il13ra1	NM_133990	8.35E-05	-16.2697	3.43319	9.36041	0.016434	-5.92722	-60.8513
Tmem45a	NM_019631	1.54E-05	-24.9143	3.63748	9.57398	0.016328	-5.9365	-61.2444
Prrx1	NM_175686	3.39E-06	-36.423	4.44543	10.501	0.015034	-6.05561	-66.5152
Lama4	NM_010681	1.01E-06	-49.2923	4.17311	10.2368	0.01495	-6.0637	-66.8891
Ddx3y	NM_012008	2.00E-07	-73.9511	3.74352	9.80847	0.014937	-6.06495	-66.9473
9030425E11Rik	NM_133733	4.49E-07	-60.4461	3.9171	9.98987	0.014856	-6.07277	-67.3111
Pdpn	NM_010329	0.000114	-15.0399	4.81231	10.9744	0.013965	-6.16205	-71.6083
Pi15	NM_053191	6.47E-05	-17.3542	3.53525	9.79539	0.013047	-6.26014	-76.6458
A430107O13Rik	BC151018	8.79E-07	-51.0831	4.1562	10.4306	0.012919	-6.27442	-77.4083
Lox	NM_010728	1.86E-07	-75.3571	3.77906	10.1441	0.012132	-6.36501	-82.425

Fgf7	NM_008008	2.46E-05	-22.1473	4.9176	11.3487	0.011589	-6.43115	-86.2915
Hgf	NM_010427	2.73E-05	-21.5717	3.73417	10.1825	0.011451	-6.44835	-87.3267
Abi3bp	NM_001014423	1.96E-06	-41.8048	3.76887	10.2219	0.011414	-6.45307	-87.6128
Gem	NM_010276	3.81E-07	-62.9843	4.55526	11.0189	0.011331	-6.4636	-88.2545
Pros1	NM_011173	4.62E-05	-18.8988	3.53465	10.0614	0.010846	-6.52675	-92.2033
Colec12	NM_130449	1.21E-05	-26.4977	4.45377	11.0495	0.010339	-6.5957	-96.7173
Pde7b	NM_013875	4.65E-06	-33.6501	4.01712	10.6545	0.010045	-6.63735	-99.5497
Vcan	NM_001081249	8.68E-08	-91.1507	4.42159	11.0864	0.009856	-6.66481	-101.463
Aspn	NM_025711	3.34E-05	-20.4991	3.4791	10.1871	0.009565	-6.70801	-104.547
Grem1	NM_011824	1.62E-07	-78.02	4.78704	11.5117	0.009455	-6.72464	-105.759
Pdgfra	NM_011058	3.03E-06	-37.4651	4.42145	11.2009	0.009103	-6.77946	-109.855
Osmr	NM_011019	4.15E-06	-34.6346	3.65241	10.5014	0.008675	-6.84895	-115.276
Lum	NM_008524	2.62E-06	-38.8457	3.18866	10.2186	0.007652	-7.02995	-130.685
Emb	NM_010330	7.13E-05	-16.931	3.72146	10.8922	0.00694	-7.17076	-144.083
Sfrp1	NM_013834	2.45E-06	-39.5166	4.03213	11.557	0.00543	-7.52487	-184.167
Saa3	NM_011315	6.35E-06	-31.1196	4.4165	12.0401	0.005071	-7.62362	-197.215
Dpt	NM_019759	2.32E-06	-40.0563	3.9402	12.1019	0.003492	-8.16171	-286.364
Ptn	NM_008973	1.90E-06	-42.1307	3.78246	11.9542	0.003468	-8.17179	-288.373
Serpina3n	NM_009252	1.15E-07	-85.0159	4.05706	12.5826	0.002714	-8.5255	-368.495
Dcn	NM_007833	3.48E-09	-203.779	4.31823	13.0228	0.002397	-8.70462	-417.207
